# Effects of *Trichoderma harzianum* combined with *Phanerochaete chrysosporium* on lignin degradation and humification during chicken manure and rice husk composting

**DOI:** 10.3389/fmicb.2025.1515931

**Published:** 2025-02-28

**Authors:** Senmao Zhai, Kuang Wang, Fengcun Yu, Zhenlu Gao, Xu Yang, Xiuqing Cao, Hiba Shaghaleh, Yousef Alhaj Hamoud

**Affiliations:** ^1^Anhui and Huaihe River Institute of Hydraulic Research, Anhui Provincial Key Laboratory of Water Science and Intelligent Water Conservancy, Hefei, China; ^2^College of Hydrology and Water Recourses, Hohai University, Nanjing, Jiangsu, China; ^3^The Key Lab of Integrated Regulation of Resource Development on Shallow Lakes, Ministry of Education, College of Environment, Nanjing, Jiangsu, China

**Keywords:** chicken manure, rice husk, lignin, humification, *Trichoderma harzianum*, *Phanerochaete chrysosporium*, fungal community

## Abstract

The purpose of this study was to investigate the effects of combined treatment of *Trichoderma harzianum* and *Phanerochaete chrysosporium* on lignin degradation and humification during aerobic composting. Chicken manure (CM) and rice husk (RH) were used as organic raw materials for composting. The basic physicochemical analysis indicated that the combined addition of *Trichoderma harzianum* and *Phanerochaete chrysosporium* effectively improved lignin degradation rate (16.60%), increased humic acid (HA) content (22.70 g/kg), and the germination index (GI) reached 110.99%. Fungal community revealed that the relative abundance of *Ascomycota* was 37.46–68.85%, 9.57–60.35%, 58.02–91.76%, 0.98–91.60% in CK, T1, T2, T3 and *Basidiomycota* was 7.81–36.03%, 7.84–3.55%, 4.42–9.60%, 0.06–8.05% in CK, T1, T2, T3 (in phylum); the relative abundance of *Kazachstania* was 0.001–68.48%, 0.62–14.60%, 7.06–25.45%, 0.001–38.16% in CK, T1, T2, T3 and *Diutina* was 2.67–7.97%, 1.11–34.42%, 15.79–64.41%, 0.25–35.34% in CK, T1, T2, T3 (in genus) during the composting. Especially, the combined addition of *Trichoderma harzianum* and *Phanerochaete chrysosporium* had more negative impact on the activity of *Basidiomycota* compared with CK and other treatments and *Trichoderma harzianum* treatment had the strongest inhibitory effect on *Tausonia* abundance compared with CK and other treatments. Correlation analysis indicated that moisture content influenced fungal community structure (*r* = −0.740, *p* < 0.01) which affected lignin degradation (*r* = −0.952, *p* < 0.01) and compost maturity level in the composting process. Fungi Functional Guild (FUNGuild) and correlation heatmap demonstrated that T3 could enhance the relative abundance of endophyte which may had the potential to improve the degradation of lignin. This study confirmed the positive effects of the combination of *Trichoderma harzianum* and *Phanerochaete chrysosporium* in enhancing lignin degradation and promoting compost maturity, providing a foundation for a deeper understanding of the mechanisms involved in lignin degradation and humification processes influenced by the fungal community during composting, ultimately contributing to the efficient utilization of agricultural waste resources.

## 1 Introduction

The rapid development of intensive agriculture in China has resulted in large amounts of livestock manure and crop straw (Ma et al., 2018). The annual amounts of livestock and poultry manure in China is 4.2 billion tons (Awasthi et al., 2021). It contains about 400 million tons of chicken manure (Li et al., 2013), resulting in serious environmental problems. Aerobic composting is an effective way to convert organic matter into humus with various functional groups to realize efficient utilization of livestock and poultry manure resource (Xie et al., 2021).

In the initial phase of composting, the lignin content in the pile increased due to the use of straw, wood chips, and other conditioners. The degradation of lignin is a vital factor influencing the humification process of composting (Yu et al., 2022). In recent years, lignin degradation in the composting process has been studied by many scholars. At present, the main methods to accelerate the lignin degradation in the composting process are the pretreatment of composting raw materials and the inoculation of microbiotics. Zhu et al., (2021a) found that thermal pretreatment can accelerate the lignin degradation during the composting process of dairy manure. Zhang et al. (2021) found that the inoculation of *Bacillus* could promote the degradation of lignin during composting. Moreover, Yu et al. (2022) found that the bacteria that degraded lignin were mainly mesmophilic bacteria and thermophilic bacteria. In particular, *Fibrobacter*, *Clostridium sensustricto* and *Geofilm* are the main bacteria genera of bacteria community (Yu et al., 2022).

Composting is a complex process in which microbial activity plays a key role in the biotransformation of organic matter. Most studies only focus on the bacterial community of compost (Storey et al., 2015; Tortosa et al., 2017), but neglect the mechanism of fungal community on organic matter such as lignin degradation. Fungal communities have different effects in the transformation from organic matter into stable and mature humus, thus resulting different influence on compost maturation (Belyaeva and Haynes, 2009; de Gannes et al., 2013). Arantes et al. (2012) found that *Phanerochaete chrysosporium* can produce hydroxyl free radicals and degrade lignin through Fenton chemical oxidation. To efficiently degrade lignin, white-rot fungi evolved unique ligninolytic peroxidases, such as manganese peroxidase, lignin peroxidase or the versatile peroxidase, showing unique characteristics, such as mediator utilization and surface-active sites to increase redox potential (Pham et al., 2018). The *Trichoderma* genus belongs to *Ascomycota* phylum of fungi which are found in almost all geographical climatic areas of various habitats, including agricultural felds, woodlands, marshes, and deserts (Asis et al., 2021). Some research found that *Trichoderma harzianum* posses potential for lignin degradation due to high ligninolytic enzyme production (Sijinamanoj et al., 2021). Although *Trichoderma harzianum* and *Phanerochaete chrysosporium* have been reported as incompatible (Alam et al., 2003), some studies demonstrate their combined effectiveness in large-scale solid-state bioconversion of domestic wastewater sludge (Molla et al., 2004). However, research on their combined effect on lignin degradation during composting remains limited.

Although there have been a lot of research about the succession of fungal communities during composting (Xie et al., 2021), while few research focus on the changes about functional classification of fungal communities during composting. Fungi functional guild (FUNGuild) can be used to analyze fungal sequences, independent of sequencing platforms or analytical pipelines, to predict the functional diversity of fungal communities (Nguyen et al., 2016). This method has been used to analyze functional groups structures in soil (Toju et al., 2016) and compost (Wang et al., 2018a). The comprehensive understanding and full characterization of the functional groups succession of fungal communities during composting can improve the composting efficiency and quality of compost.

Some recent studies investigated effects of bacterial communities on degradation of organic compounds (Storey et al., 2015), and the influence of additives on succession and abundance of fungi (Xie et al., 2021), but the relationships between the fungi community and the lignin degradation during composting remain unclear. Especially, how combined addition of *Trichoderma harzianum* and *Phanerochaete chrysosporium* impact the successive progression of the fungi to affect humification of compost remains to be understood. The objectives of this study were to: (i) reveal the effects of different treatments on lignin content and the humification process during composting, (ii) compare the effects of different treatments on fungal communities, and (iii) quantitatively reveal the relationship between fungal abundance, functional groups and compost maturity. This study can help us further understand the mechanisms about microbial community involvement in lignin degradation and maturation during the composting.

## 2 Materials and methods

### 2.1 Composting materials and experimental design

Composting was set at the Institute of Animal Husbandry and Poultry Science in Nanjing, China. Chicken manure and rice husks were used as raw materials, and the activated fungal liquid of *Trichoderma harzianum* (SHBCC-D82129) and *Phanerochaete chrysosporium* (NDM3-2) were used as additional agents. Before inoculated, the complex microorganisms were cultivated by malt extract agar (malt 15 g/L, agar 10 g/L), and the microorganisms were cultivated by potato dextrose agar (potato extract 200 g/L 1, agar 20 g/L). During cultivating process, the microbial colonies were counted using a standard dilution-plating procedure until to reach the desired concentration of 1 × 10^9^ CFU ml/L for composting inoculation. Chicken manure was mixed with rice husk, in a ratio of 4:1 (on a wet weight basis). The carbon nitrogen ratios was 20. The moisture content is adjusted to 65% by adding distilled water. Three treatments were set up in this experiment, which were CK: adding 0.1%(v/w) distilled water; T1: adding 0.1%(v/w) *Trichoderma harzianum*; T2: adding 0.1%(v/w) *Phanerochaete chrysosporium*; T3: adding 0.05%(v/w) *Trichoderma harzianum* + 0.05%(v/w) *Phanerochaete chrysosporium*, respectively. The total weight of each treatment was 20,000 g. This experiment was set up in a foam box (length: 1.2 m; width: 0.8 m; height: 0.8 m) with thickness of 0.03 m.

To mix the composting materials and provide aeration, a manual turning was performed at a 5 day interval until the compost was mature. This experiment lasted 35 days. All materials for this experiment were provided by Nanjing Institute of Animal Husbandry and Poultry Science. Samples were collected at 16:00 on days 0, 1, 3, 5, 7, 14, 21, and 35. Samples were collected from the upper, central and lower regions of each composting pile. The sub-samples were mixed thoroughly. Each sample was divided into two parts, one for physicochemical parameter determination and the other for high-throughput sequencing. Each treatment has triple replicates (Table 1).

**Table 1:** Physicochemical properties of raw composting materials.

Raw materials	TOC^a^(g/kg)	TN^a^(g/kg)	C/N^a^	pH^b^	MC^b^(%)
Chicken manure	350.62 ± 2.15	15.81 ± 0.14	22.18 ± 1.33	6.05 ± 0.16	68.26 ± 1.51
Rice husk	417.2 ± 2.31	10.17 ± 0.06	41.02 ± 0.99	–	8.03 ± 0.07

a represents dry weight.

b represents wet weight.

### 2.2 Analytical methods

The temperature was determined by an electronic thermometer (TP101, Innovation, Shenzhen, China). Moisture content (MC) was determined by drying method. Fresh compost samples were mixed with distilled water (solid–liquid ratio is 1:10) for 30 min of oscillating extraction, and then left for 30 min for filtration. The pH was measured by multi-parameter analyzer (DZS-706-A, Leimi, Shanghai, China). Chinese cucumber (*Cucumis sativus L.*) seed was used for the germination index (GI) measurement according to the following formula: GI (%) = seed germination numbers × root length of treatment × 100/(seed germination numbers × root length of control; Qiao et al., 2019). Total organic carbon (TOC) was measured by potassium dichromate oxidation method (Ren et al., 2019).

Humus components [humic substances (HS), humic acid (HA), fulvic acid (FA)] were measured based on the operation of Zhao et al. (2024). 1 g of sample was mixed with a 20-fold mixture of 0.1 M Na_4_P_2_O_7_·10H_2_O and 0.1 M NaOH (w/v, dry weight). The mixture was then incubated with continuous agitation at 200 rpm for 24 h at room temperature. After that, the mixture was centrifuged at 8000 rpm for 10 min. This process was repeated twice. The obtained supernatants were combined and filtered through a 0.45 μm filter membrane. The resulting solution was the compost HS solution. Thereafter, HS was divided into two parts. One part was stored at −20°C, and the other part was used for the separation of FA and HA. The HS was adjusted to pH 1.5 by using 6 M HCl and allowed to sit at 4°C for 12 h. After centrifuging at 8000 rpm for 10 min, the resulting supernatant was the FA, while the precipitate was HA. The HA precipitate was alternately washed three times with 0.1 M HCl and deionized water. The washed HA precipitate was completely dissolved in a 0.03 M Na_2_CO_3_ solution, resulting in a HA solution. The dried matter ratio of HA to FA was H/F.

The lignin content was measured based on the operation of Soest et al. (1991). The air-dried sample was crushed, and 0.2000 g was placed into a centrifuge tube. Following this, 10 mL of a 1% acetic acid solution was added, and the mixture was shaken and centrifuged. The precipitate was then washed with 5 mL of 1% acetic acid. Next, 3–4 mL of a mixture of ethanol and ether (in a 1:1 volume ratio) was added, and the mixture was soaked for 3 min. Afterward, the supernatant was discarded, and the precipitate was soaked two more times with the same ethanol and ether mixture. The remaining precipitate in the centrifuge tube was subjected to steam in a boiling water bath, then transferred to a new container. To this, 3 mL of 72% sulfuric acid was added, and the mixture was stirred well with a glass rod. It was allowed to stand at room temperature for 16 h to ensure complete dissolution of the cellulose. Following this, 10 mL of distilled water was added to the test tube, and the mixture was stirred again with a glass rod. The tube was placed in a boiling water bath for 5 min, then allowed to cool. Subsequently, 5 mL of distilled water and 0.5 mL of a 10% barium chloride solution were added, and the mixture was shaken well before centrifugation. The precipitate was then washed twice with distilled water. The rinsed lignin precipitate was treated with 10 mL of 10% sulfuric acid and 10 mL of a 0.1 mol/L potassium dichromate solution, and the test tube was placed in a boiling water bath for 15 min. After boiling, all contents of the cooled test tube were transferred to a beaker for titration, and any residual material was washed out with 15–20 mL of distilled water. Finally, 5 mL of 20% potassium iodide (KI) solution and 1 mL of 0.5% starch solution were added to the beaker, and the mixture was titrated with 0.2 mol/L sodium thiosulfate solution.

$\begin{array}{l} \mathrm{lignin degradation rate}\;\left(\%\right)\\ =\left(\mathrm{lignin content}\;\left(\mathrm{day0}\right)-\mathrm{lignin content}\;\left(\mathrm{day}35\right)\right)/\mathrm{lignin content}\;\left(\mathrm{day0}\right)\text{.} \end{array}$

### 2.3 Diversity succession of fungal communities

DNA was extracted with the TGuide S96 kit (DP812, TIANGEN BIOTECH CO., LTD.), and then the internal transcribed spacer 1 between 18S and 5.8S rRNA genes (ITS1 rRNA) was amplified by PCR using primers 5′-ACTTCGTACTTACGTAAT-3′ and 5′-TGCATTATTAGCATTAA-3′. PCR products were purified with the OMEGA DNA Kit and quantified using Qsep-400. The amplicon library was paired-end sequenced (2 × 250) on an illumina novaseq6000 (Beijing Biomarker Technologies Co., Ltd., Beijing, China).

The raw sequence data were processed in QIIME 1.8.0, Operational units (OTUs) were clustered with 97% similarity cutoff using UPARSE (USEARCH, version 10.0) and chimeric sequences were identified and removed using UCHIME v4.2. Fungal species were annotated with Unite (Release 8.0).^1^

### 2.4 Statistical analysis

Figures were performed by Origin 2018 (OriginLab, Northampton, United States). Significance analysis was performed by SPSS 24 (IBM, Chicago, United States). Alpha diversity, beta diversity and FunGuild were analyzed by TIANGEN BIOTECH (BEIJING) CO., LTD. Mantel test (Li et al., 2023) network analysis and heatmap (Gu et al., 2017) were performed by R language (Version 2.15.3).

## 3 Results

### 3.1 Changes in basic physicochemical parameters during composting

**Figure 1:**
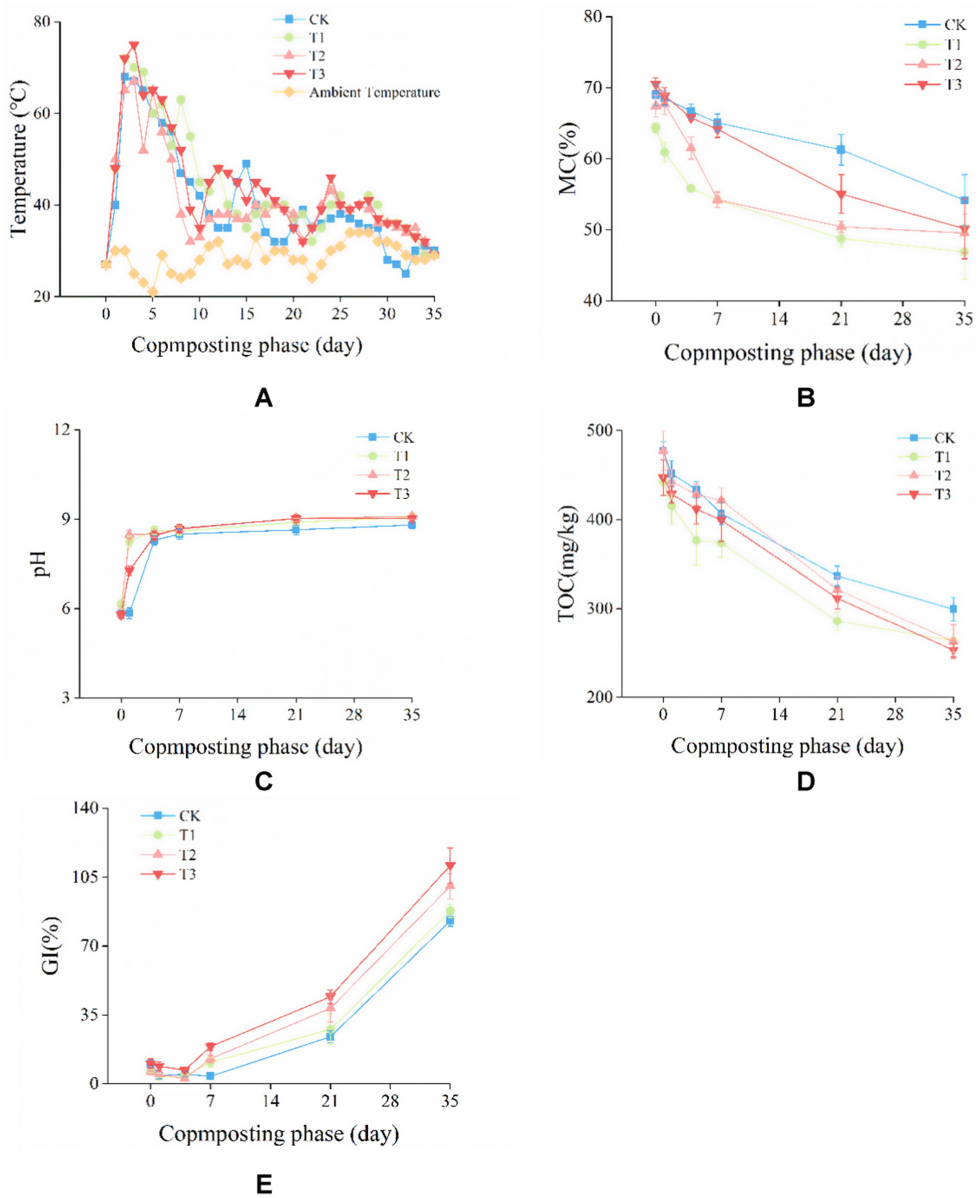
Changes in **(A)** temperature, **(B)** moisture content (MC), **(C)** pH, **(D)** total organic carbon (TOC), and **(E)** germination index (GI) during composting.

During composting, the temperatures increased were due to local microbes can decompose available organic matter and produce energy (Wong et al., 2009). As is shown in Figure 1A, the temperature of all treatments rapidly increased into the thermophilic phase (> 50°C) which was maintained for 6–9 days, and then the temperatures were decreased. To destroy pathogens and weed seeds in the compost, a temperature above 55°C which lasts for at least 3 days is necessary (Bernal et al., 2009). At the end of composting, with the temperature of all treatments dropping to ambient temperature, MC of CK, T1, T2 and T3 decreased from 68.99, 64.40, 67.43, 70.53% of the initial phase to 54.16, 46.88, 49.50, 50.14% (Figure 1B).

The pH of CK, T1, T2, and T3 in the initial phase was 5.84, 6.14, 5.85, and 5.80 (Figure 1C). In the first 7 days of composting, the pH value of each treatment increased rapidly, and then the pH increase rate slowed down until stable. At the end of composting, the pH of CK, T1, T2, and T3 were 8.81, 9.07, 9.10 and 9.02. The results showed that there was no significant difference in pH among all treatments, which met the requirement of weakly alkaline compost (General Administration of Quality Supervision, Inspection and Quarantine of the People's Republic of China, Standardization Administration of China, 2013).

The changes in TOC reflected the degree of mineralization and humification of compost, and significantly affected the quality of compost (Larney et al., 2006). As is shown in Figure 1D, the TOC content in CK, T1, T2 and T3 decreased from 477.35, 442.41, 477.35, 447.33 g/kg on day 0 to 299.29, 263.61, 262.72, 253.02 g/kg on day35. TOC was decreased by 37.30, 40.41, 44.96 and 43.44% in CK, T1, T2 and T3, respectively. Compared with CK, the decline range of TOC of T1, T2 and T3 were significantly (*p* < 0.05) higher. The results showed that the inoculation of microbial agent could improve the mineralization of TOC.

Seed germination index (GI) is the most commonly used index to evaluate the biological toxicity and maturity of compost (Larney et al., 2006). As is shown in Figure 1E, the GI value of each group increased in general. However, GI of CK, T1, T2, and T3 decreased from 10.20, 6.42, 6.06, 10.31 to 4.82%, 3.81, 2.58, 6.90% in the first 4 days, respectively. This was mainly due to the accumulation of substances such as fatty acids of low molecular, NH_3_ and toxic nitrogen compounds (Ajmal et al., 2021). At the end of composting, GI increased to 82.75, 87.91, 100.49 and 110.99%, respectively. The GI of T2 and T3 were significantly (*p* < 0.05) higher than those of T1 and CK. These results showed that the inoculation of *Phanerochaete chrysosporium* and the combined addition can effectively improve the humification degree of compost.

### 3.2 Changes in lignin content and humification during composting

**Figure 2:**
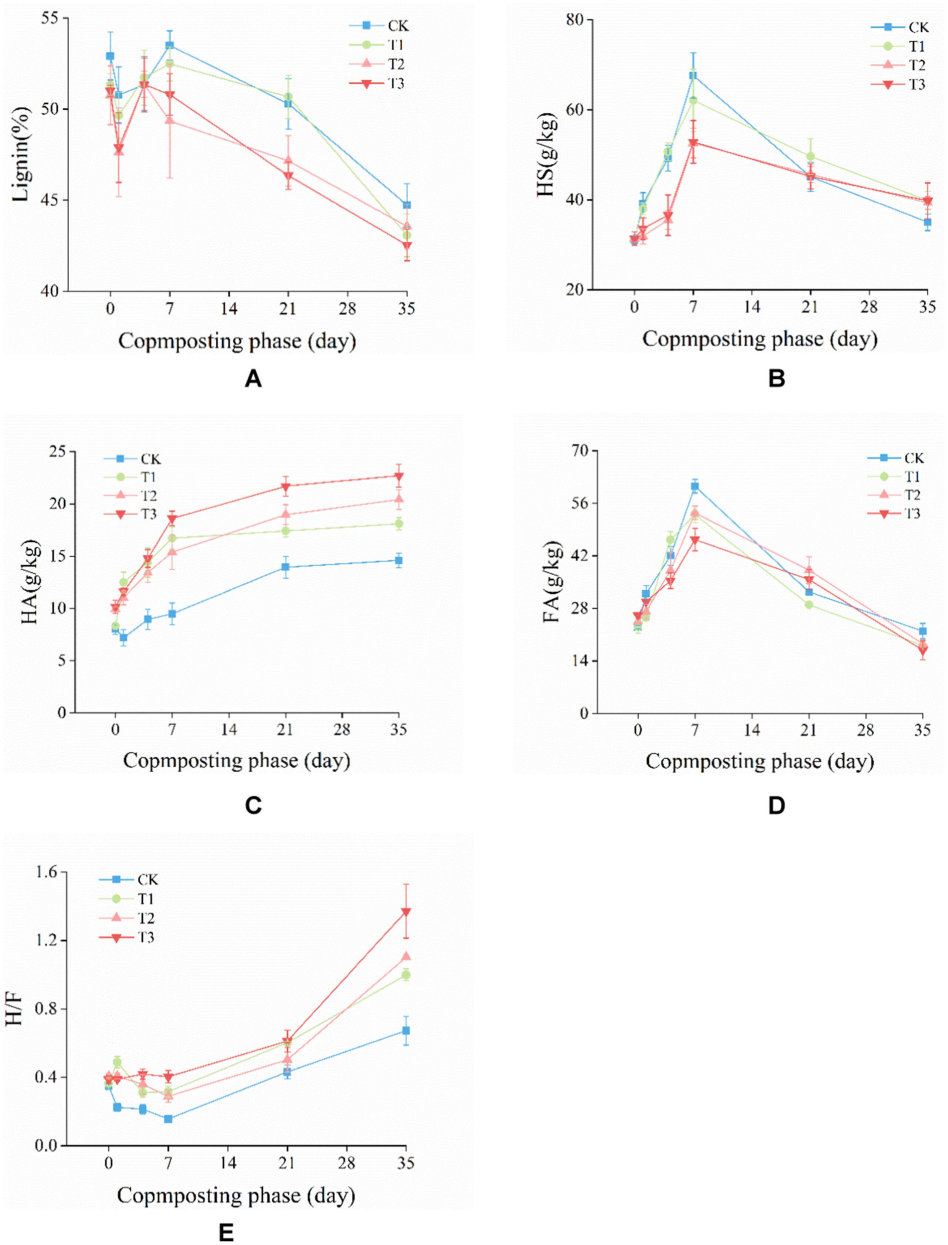
Changes in **(A)** lignin content, **(B)** humic substances (HS), **(C)** humic acid (HA), **(D)** fulvic acid (FA), and **(E)** ratio of HA to FA (H/F) during composting.

Lignin, a component of lignocellulosic material, is highly resistant to microbial degradation (Hoang et al., 2021). Additionally, the degradation of cellulose and hemicellulose occurs earlier than that of lignin, providing carbon sources for microbial metabolism and growth. This may lead to a decrease in the degradation rate of lignin during the thermophilic phase of composting (Ma et al., 2020). As depicted in Figure 2A, the lignin content initially decreased and then increased from day 0 to day 4. This was primarily due to the initial degradation of organic matter such as sugars, proteins, and amino acids by microorganisms in the compost pile, leading to a reduction in pile volume and an increasing in lignin content (Zhu et al., 2021b). As composting progressed, the lignin content gradually decreased. At the end of composting, the lignin content of the CK, T1, T2, T3 decreased from 52.91, 51.34, 50.76, 51.00% to 44.73, 43.08, 43.57, 42.54%, respectively, compared to day 0. The lignin degradation rate of CK, T1, T2, and T3 is 15.47, 16.10, 14.16, and 16.60%, respectively. Compared with CK, T1, T2, the lignin degradation rate of T3 was increased by 7.33, 3.12, 17.18%.

The composition of humus (HS, HA, and FA) is a crucial indicator for assessing compost maturity. As illustrated in Figure 2B, in the first 7 days of composting, the HS content exhibited an upward trend. However, as composting progressed, the HS content started to decrease. This was primarily due to the reduction in pile temperature, which accelerated microbial metabolism and improved the decomposition of HS (Barje et al., 2012). At the end of the composting process, the HS content of the CK, T1, T2, and T3 groups was 35.00, 39.88, 39.34, and 39.80 g/kg, respectively, with no significant difference between the treatment groups. The HS content of T1, T2 and T3 were significantly (*p* < 0.05) higher than CK. As the composting progressed, the HA content (Figure 2C) showed a gradual increasing trend, which was consistent with the findings reported by Bai et al. (2020) study. At the end of composting, the HA content was 14.60, 18.09, 20.42 and 22.70 g/kg for CK, T1, T2, and T3. Compared with CK, T1, T2, the HA content of T3 were significantly (*p* < 0.05) higher; the FA content decreased from 23.04 to 21.95, 18.12, 18.53, and 16.83 g/kg for CK, T1, T2, and T3, respectively (Figure 2D). Compared with CK, T1, T2, the HA content of T3 were significantly (*p* < 0.05) lower. The H/F initially decreased and then increased. Similar results were reported by Zhou et al. (2022). Moreover, the H/F increased to 0.67, 0.99, 1.10, and 1.37 for CK, T1, T2, and T3, respectively (Figure 2E). These findings suggested that the inoculation of microbial agent could improve HS content, and the combined addition could enhance HA content, reduce FA content.

### 3.3 Fungal community distribution and succession during composting

Alpha diversity represents the diversity of fungal communities within a single sample. It reflects the species abundance and species diversity in compost. Various indices can be used to measure alpha diversity, including Chao1, Ace, Shannon, Simpson, and Pielou_e. The Chao1 and Ace indices indicate species abundance, specifically the number of species present. The Shannon, Simpson, and Pielou_e were used to measure species diversity and were influenced by both specie abundance and evenness of the fungal community. When species abundance is the same, a higher evenness within the fungal community indicates greater diversity. A higher value for the Shannon and Simpson indicates greater species diversity of the fungal community (Grice et al., 2009). According to the Table 2, the species diversity of each treatment initially decreased and then increased, which suggested that high temperature during the thermophilic phase of composting reduced fungal community diversity.

**Table 2:** Alpha diversity of the fungi during composting.

Sample ID	ACE	Chao1	Simpson	Shannon	Pielou_e
CM0	461.9322	448.5541	0.8788	4.5914	0.5263
CKD1	209.908	202.7381	0.8271	3.5497	0.4666
T1D1	325.5221	324.1034	0.9175	4.8033	0.5797
T2D1	137.5631	135.8333	0.8229	3.172	0.4517
T3D1	134.504	130.6667	0.8803	3.6493	0.5248
CKD35	626.36	616.4304	0.953	6.3179	0.6837
T1D35	700.5415	703.2381	0.8978	5.5448	0.5897
T2D35	251.6739	251.0417	0.9756	6.1513	0.7717
T3D35	249.9393	250.24	0.7661	3.1948	0.4037

*CMO represents the raw materials.

**Figure 3:**
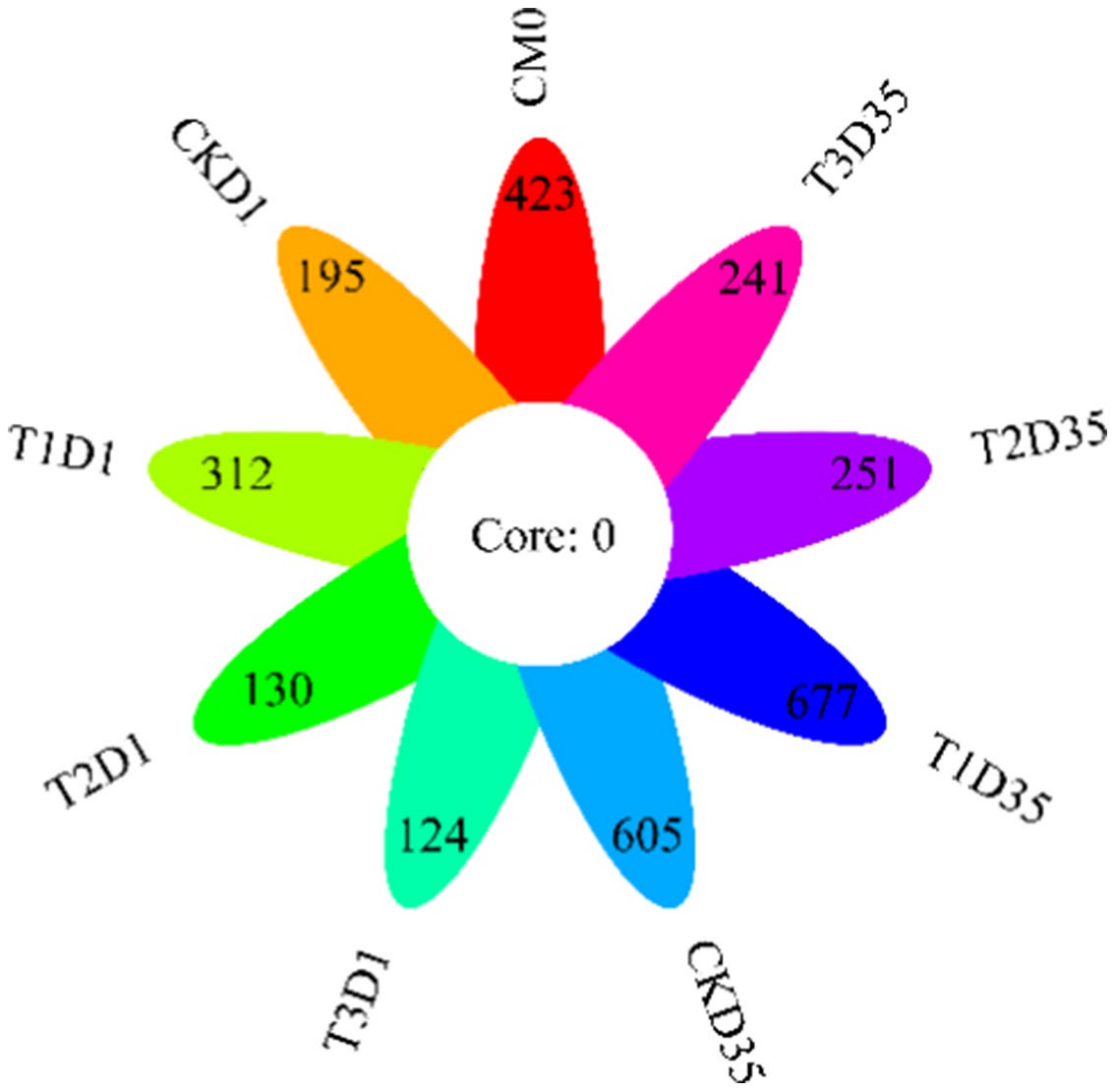
Changes in OTUs during composting (CM0: the raw materials).

The petal diagram in Figure 3 was based on the OTUs (Operational Taxonomic Units). In the diagram, different colors and petals represented different treatments and samples, respectively. The core value represented the number of shared OTUs among the treatments. None of the treatments had the same OTUs number, which was 0. In the raw materials, there were a total of 423 OTUs. By the end of composting, the number of OTUs in CK, T1, T2, and T3 increased from 195, 312, 130, 124 to 605, 677, 251, 241, respectively. The results indicated that T1 had the highest number of OTUs, which was higher than that of CK, T2, and T3.

**Figure 4:**
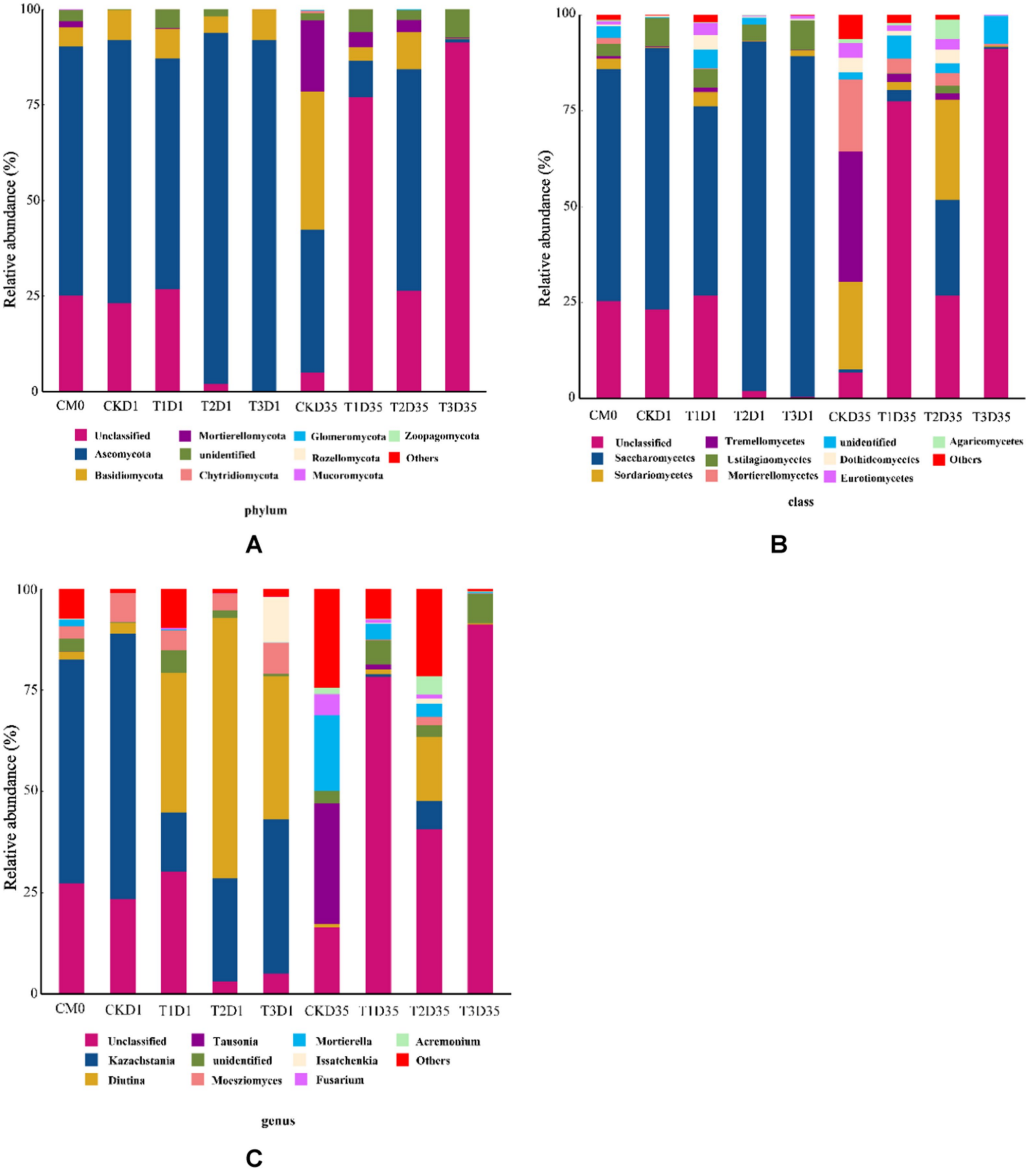
Changes in microbial community in phylum **(A)**; class **(B)**; genus **(C)** during composting (CM0: the raw materials).

As is shown in Figure 4A, the relative abundance of *Ascomycota* and *Basidiomycota* in raw material were 65.22 and 4.99%. As the temperature rising, in the day 1, *Ascomycota* was 68.85, 60.35, 91.76 and 91.60%, *Basidiomycota* was 7.81, 7.84, 4.42 and 8.05%, for CK, T1, T2, and T3, respectively. At the end of composting, *Ascomycota* was 37.46, 9.57, 58.02, and 0.98% for CK, T1, T2, and T3, respectively. Similarly, the relative abundance of *Basidiomycota* was 36.03, 3.55, 9.60 and 0.06% for CK, T1, T2, and T3, respectively. These results demonstrated variations in the fungal community structure among different treatments, which could be attributed to the presence of different additives during the initial phases of composting. The results indicated that T3 had more negative impact on the activity of *Basidiomycota* compared with CK and other treatments during composting.

In Figure 4B, the relative abundances of fungal communities at the class level for all treatments are displayed. In the *Ascomycota* phylum: in the raw material, the relative abundance of *Saccharomycetes* and *Sordariomycetes* were 60.43 and 2.75%. In the *Basidiomycota* phylum: in the raw material, the relative abundance of *Ustilaginomycetes* and *Tremellomycetes* were 3.12 and 0.63%. In Figure 4C, the relative abundance of fungal communities at the genus level for all treatments is presented. In the *Ascomycota* phylum: in the raw material, the relative abundance of *Kazachstania* and *Diutina* were 55.27 and 1.98%. As the temperature rising (in the day 1), the relative abundance of *Kazachstania* decreased to 14.60, 25.45, 38.16% in T1, T2, T3, but increased to 65.48% in CK; the relative abundance of *Diutina* increased to 2.68, 34.42, 64.41, 35.34% in CK, T1, T2, T3. At the end of composting (day 35), the relative abundance of *Kazachstania* decreased to 0.001, 0.61, 7.05, 0.001% in CK, T1, T2, T3; the relative abundance of *Diutina* decreased to 0.80, 1.1, 15.79, 0.25% in CK, T1, T2, T3. In the *Basidiomycota* phylum: in the raw material, the relative abundance of *Moesziomyces* and *Tausonia* were 3.19 and 0.02%. As the temperature rising (in the day 1), the relative abundance of *Moesziomyces* increased to 7.29, 4.91, 4.24, 7.75% in CK, T1, T2, T3; the relative abundance of *Tausonia* decreased to 0.004, 0.003, 0.004, 0.001% in CK, T1, T2, T3. At the end of composting (day 35), the relative abundance of *Moesziomyces* decreased to 0.01, 0.18, 2.05, 0.036% in CK, T1, T2, T3; the relative abundance of *Tausonia* increased to 29.86, 1.28, 0.01, 0.002% in CK, T1, T2, T3.

The results indicated that: in the *Ascomycota* phylum, compared with other treatments, T3 had the strongest inhibitory effect on *Kazachstania* abundance and the strongest promoting effect on *Diutina* abundance during composting; in the *Basidiomycota* phylum, compared with other treatments, T1 had the strongest inhibitory effect on *Tausonia* abundance during composting.

**Figure 5:**
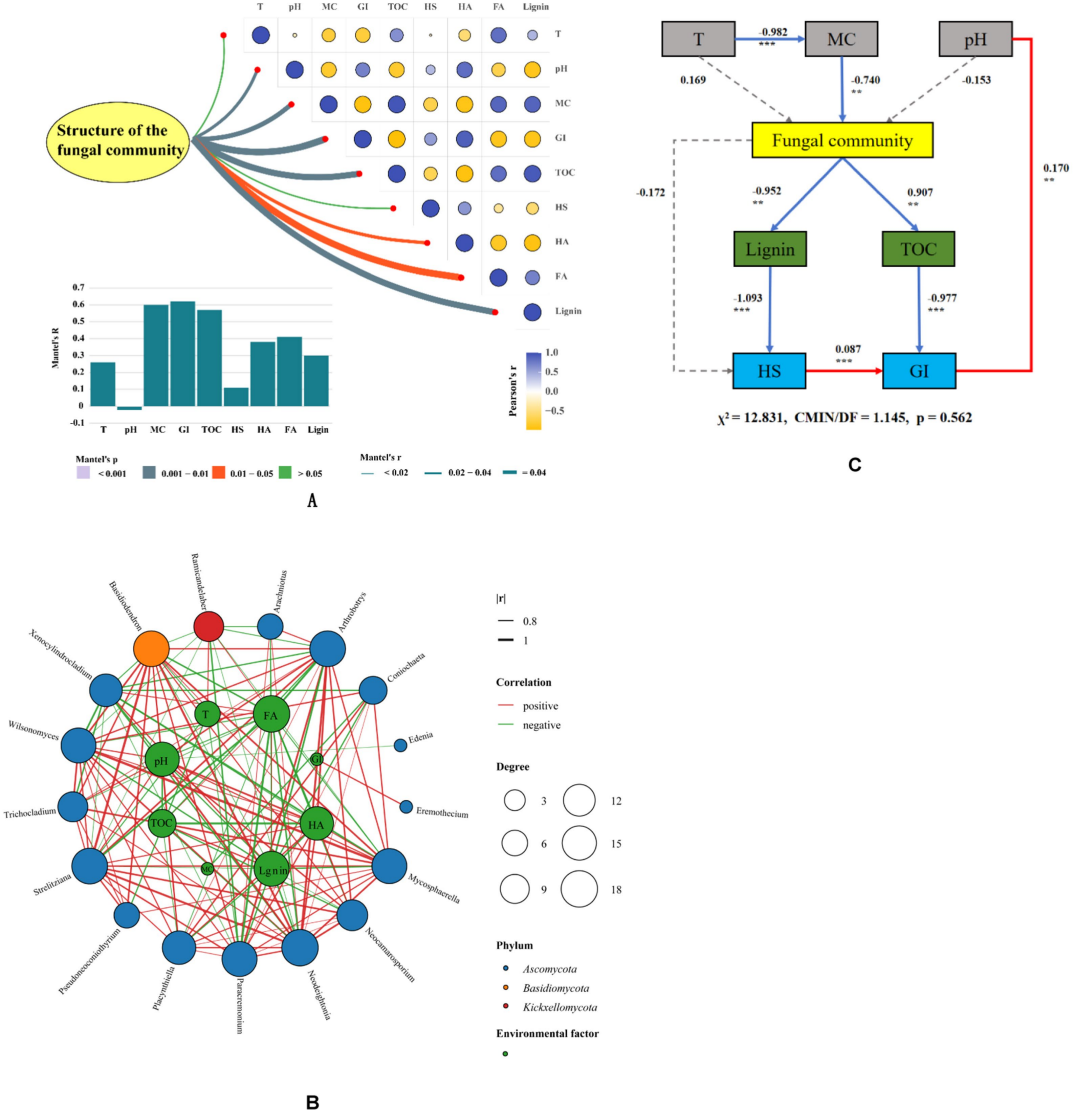
Correlation between different microbial groups, humification indexes and environmental factors in composting **(A)** Mantel test between humification, environmental factors and structure of fungal community. **(B)** Co-occurrences of fungal communities with humification and environmental factors during the composting process. **(C)** Structural equation model (SEM) analysis on relationships among environmental factors, fungal community and humification during composting. *p < 0.05, **p < 0.01, ***p < 0.001 (T, temperature; MC, moisture content; HS, humic substances; HA, humic acid; FA, fulvic acid; GI, germination index).

The Mantel test (Figure 5A) revealed that the fungal community had the extreme significant correlations with MC, GI, and TOC, had the significant correlations with lignin, HA, FA. These findings highlight the need to analyze the correlation between the dominant fungi and environmental factors, as well as the degree of decomposition, during the composting process.

Network analysis was employed to explore the relationship between microorganisms and environmental factors during composting. Figure 5B illustrates that *Ascomycota* played a crucial role in shaping the microbial habitat. At the genus level, *Arthrobotrys*, *Mycosphaerella*, *Paracremonium*, *Basidiodendron*, and *Ramicandelaber* had important effects on the microbial habitat. At the phylum level, *Ascomycota* is negative correlated with *Basidiomycota* and *Kickxellomycota*, while *Basidiomycota* exhibits a negative correlation with *Kickxellomycota*. At the genus level, *Arthrobotrys* is positively correlated with *Neodeightonia*, *Mycosphaerella* is positively correlated with *Wilsonomyces*, *Paracremonium* is positively correlated with *Basidiodendron*, Neocamarosporium is negatively correlated with *Ramicandelaber*, *Ramicandelaber* is negatively correlated with *Arachniotus*, and *Basidiodendron* is negatively correlated with *Xenocylindrocladium*. Importantly, temperature is also positively correlated with *Basidiodendron*. *Basidiodendron* is also positively correlated with HA but negatively correlated with FA. *Coniochaeta* exhibits a negative correlation with lignin content and MC. *Neocamarosporium* is positively correlated with HA, indicating its potential role in promoting humification. On the other hand, *Arthrobotrys* exhibits a negative correlation with lignin and TOC, indicating its involvement in the degradation of these components during composting. These findings indicate that the structure of the fungal community has varying effects on the composting process. Therefore, it becomes necessary to regulate the fungal community based on the specific conditions during composting, for enhancing the maturation of compost.

The relationship between environmental factors, fungal community and humification of compost was further investigated by SEM structural equation. As shown in Figure 5C, MC was negatively correlated with fungal community (−0.742, *p* < 0.01). Fungal community was negatively correlated with lignin content (−0.952, *p* < 0.01) and TOC (−0.907, *p* < 0.01). At the same time, it was found that the decrease of lignin and TOC could effectively increase HS and GI. These results indicate that reducing MC and pH of compost within the suitable range can effectively regulate the structure of fungal community, promote the degradation of lignin, and improve the maturity of compost.

### 3.4 Functional guild and correlations between functional groups, environmental factors and humification

**Figure 6:**
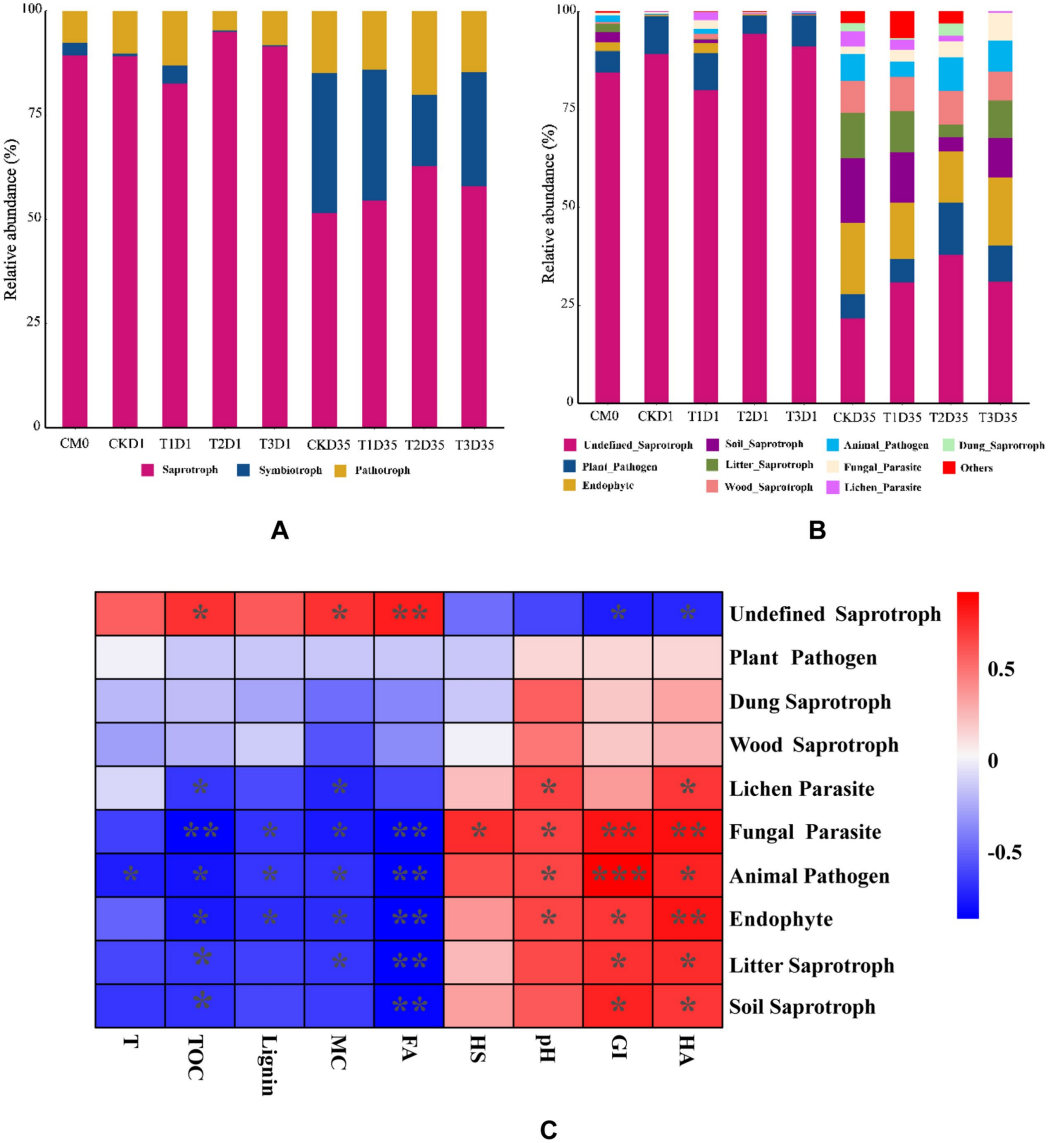
Relative abundance of **(A,B)** fungal functional guilds during composting. **(C)** correlation heatmap between functional groups, environmental factors and humification. *p < 0.05, **p < 0.01, ***p < 0.001 (CM0, the raw materials; T, temperature; MC, moisture content; HS, humic substances; HA, humic acid; FA, fulvic acid; GI, germination index).

Funguild, as an effective fungal community analysis method, is used to predict of nutrient and functional groups of fungal communities during different phases of composting. The results revealed that the fungal communities could be categorized into three nutrient groups: saprotroph, symbiotroph, and pathotroph. In Figure 6A, the relative abundance of saprophytic, symbiotic, and pathotrophic fungi in the raw materials was determined to be 89.25, 3.00, and 7.75%, respectively. In the day1, there was a general increase in the relative abundance of pathotrophic fungi, while the relative abundance of symbiotic fungi generally decreased. Additionally, we observed varying changes in the relative classification of saprotrophic fungi. Specifically, the relative abundance of saprotrophic fungi for CK, T1, T2, and T3 samples were 89.15, 82.52, 94.97, and 91.39%, respectively. The relative abundance of symbiotic trophic fungi was 0.61, 4.27, 0.25, and 0.38%, respectively. The relative abundance of pathotrophic fungi was 10.25, 13.21, 4.78, and 8.23%, respectively. At the end of composting (day 35), the relative abundance of saprotrophic and symbiotic fungi generally increased, while the relative abundance of pathotrophic fungi showed a decreasing trend. Specifically, the relative abundances for CK, T1, T2, and T3 samples were 51.42, 54.51, 62.78, and 57.83%, respectively, for saprotrophic fungi. The relative abundances of symbiotic trophic fungi were 33.67, 31.39, 17.05, and 27.35%, respectively. The relative abundance of pathotrophic fungi was 14.91, 14.10, 20.18, and 14.82%, respectively.

The saprotrophic types encompass soil saprotroph, wood saprotroph, litter saprotroph, dung saprotroph, and undefined saprotroph. The symbiotic trophic fungi category includes endophytes. Pathotrophic fungi are further classified as plant pathogens, animal pathogens, fungal parasites, and lichen parasites. As depicted in Figure 6B, in the raw materials, the dominant functional type of fungi was Undefined Saprophytic fungi, accounting for a relative abundance of up to 84.39%. During the thermophilic phase (day 1), the main types observed were defined saprophytic fungi. The relative abundances of undefined saprophytic fungi for CK, T1, T2, and T3 were 89.05, 79.98, 94.33, and 91.06%, respectively. At the end of composting (day 35), the fungal types were mainly categorized as defined saprophytic, endophytes, and soil Saprophytes. The relative abundances of undefined saprophytic for CK, T1, T2, and T3 were 21.72, 30.95, 37.87, and 31.11%, respectively. The relative abundances of endophytes for CK, T1, T2, and T3 were 18.34, 14.35, 13.12, and 17.47%, respectively. The relative abundances of soil saprophytes for CK, T1, T2, and T3 were 16.46, 12.77, 3.59, and 9.95%, respectively. The correlationship between functional groups, environmental factors and humification was further depicted by heatmap. As shown in Figure 6C, lignin content was negatively correlated with endophyte (*p* < 0.05), but positively correlated with undefined saprotroph. GI was positively correlated with animal pathogen (*p* < 0.001). These findings suggested that the enhancing the relative abundance of endophyte may had the potential to improve the degradation of lignin.

## 4 Discussion

### 4.1 Changes of basic physicochemical parameters during composting

In this research, a control group and three treatment groups were set up to investigate the effects. The results showed that additional agent effectively prolonged the heating period of the compost, resulting in lower water content compared to the control group at the end of composting (*p* < 0.05). The pH of the compost pile increased sharply in the first 7 days due to the mineralization and ammonification of nitrogen-containing organic matter into NH_3_ (Zhang, 2013). In day 7, the pH in both the control group and treatment group was significantly higher than that in day 0 (*p* < 0.05). During the thermophilic phase of composting, the pH increased. This was primarily due to the release of NH_3_ and CO_2_ triggered by high temperature, which accelerated the degradation of organic matter and the production of organic and inorganic acids (Zhang, 2013). At the end of the composting process, the pH stabilized within the range of 8.81 to 9.11. There was no significant difference in pH between CK and other treatments.

The variation in TOC content reflects the extent of mineralization and humification during composting, which greatly impacts the quality of the compost (Larney et al., 2006). In this study, CK and other treatments showed a decreasing trend in TOC content, which is consistent with the findings reported by Ye et al. (2021). Zhang et al. (2022) also observed similar results in the chicken manure composting, in which mineralization of TOC could provide carbon sources for the formation of HA and promotes an increase in HA content and GI (Li et al., 2022; Wang et al., 2022).

### 4.2 Changes of lignin content and humification during composting

Lignin is an aromatic biopolymer that is abundantly found in the vascular tissues of plants (Mankar et al., 2021). It is a three-dimensional macromolecular organic polymer consisting of gluconol, coniferol, and para-coumarol linked by C-O-C and C-C bonds (Mankar et al., 2021). During composting, lignin is known to be one of the most resistant aromatic compounds to degradation. The experiment revealed that in the initial phase of composting, the enrichment of lignin occurs due to the degradation of simple organic compounds such as sugars, amino acids, and aliphatic groups, leading to an increase in lignin content. As composting progresses and the pile’s temperature gradually decreases, the metabolism of *Ascomycota* becomes dominant. This results in an increased rate of lignin degradation (Song et al., 2022). The oxides produced during the breakdown of lignin commonly form the “core” of HS. These oxides serve as precursor for the formation of HA (Fukushima et al., 2009). Therefore, enhancing lignin degradation and promoting the synthesis of HS are highly significant for improving composting efficiency and compost quality.

HS primarily consist of HA and FA. The results of the study demonstrated that during the initial phase of composting, the HS content increased due to the degradation of organic components such as starch, glucose, and amino acids, as well as the accumulation of humus substances like quinones and phenols (Bai et al., 2020). However, from day 7 to day 35, the degradation of certain compounds within HS by microorganisms resulted in a decrease in HS content. Additionally, it was observed that the HA content exhibited a gradual increasing trend during the composting process, which aligns with the findings of Zhu et al. (2021c) research.

FA, as an important component of HS, is low molecular weight and instability (Dias et al., 2010). It was found that FA increased notably in the early phase of composting, particularly in CK. This increase was mainly attributed to the secretion of extracellular enzymes by *Mucoromycota*, which accelerated the degradation of cellulose and hemicellulose (Perkins et al., 2021), thereby promoting FA synthesis (Zhang et al., 2018). The FA content exhibited an increasing trend in the first 7 days and a decreasing trend in the later phase of composting, which is consistent with the findings of Li et al.’s research on pig manure composting (Li et al., 2021). This decrease in FA content resulted from enhanced microbial metabolism due to a decrease in pile temperature, which accelerated FA degradation (Ren et al., 2020).

Under the influence of HA and FA contents, H/F exhibited an initial decrease followed by an increase. At the end of composting, the H/F ratio in the T3 treatment group was significantly higher compared to CK and treatment groups T1 and T2 (*p* < 0.05). Duan et al. (2019a) discovered that the H/F reached its highest level (4.92) when 10% bamboo biochar was added. The results indicated that the combined addition improved the microenvironment of microbial metabolism in the compost, accelerated the degradation of organic matter, increased the content of humic acid, and then increased H/F, achieving the same effect as using biochar.

### 4.3 Fungal community distribution and succession during composting

The results indicated that the alpha diversity index of the samples was higher than that of the raw materials at the end of composting, and the beta diversity index suggested that the composition of fungal communities varied during different phases and treatments. Microorganisms play a vital role in the humus formation process during composting by participating in the degradation and conversion of organic matter (Wei et al., 2007). It was observed that the dominant fungi in the raw materials were *Ascomycota*, with *Kazachstania* and *Diutina* in genus. As temperature of compost rising, the dominant fungi changed to *Ascomycota* and *Basidiomycota*, which is consistent with the research of Huang et al. (2022) and Duan et al. (2019b) research. *Ascomycota* and *Basidiomycota* are vital microorganisms involved in the mineralization and degradation of lignin due to the spores they produce being highly resistant to high temperatures (Schmidt-Dannert, 2016). This may explain why the lignin degradation rate of the T3 treatment group was higher compared to CK and other treatment groups. However, Wang et al. (2018b) discovered that *Mycothermus* was the dominant fungus in the cow manure composting process, possibly due to the unique digestive system of beef cattle. At the end of composting (day 35), compared with day 1, there was an increase in the relative abundance of *Aspergillus* and the secretion of peroxidase, which accelerated lignin degradation (Schmidt-Dannert, 2016). It is worth noting that a significant proportion of the fungal communities in each phase consisted of unclassified fungi, consistent with the findings of Wang et al. (2020) research. These fungi might be associated with lignin degradation and the formation of HA at high temperatures.

The Mantel test, network analysis and SEM revealed the significant interaction between the fungal community and environmental factors, which play a vital role in the humification process of composting. It is important to note that this study found that a decrease in temperature and water content led to increased activity of *Neocamarosporium* and *Basidiodendron*, thereby accelerating lignin degradation and increasing HA content. Similar findings were reported by Jiang et al. (2020) who also observed the impact of temperature and water content on microbial activity and the humification process during cow manure composting. Temperature has been identified as a key factor affecting lignin degradation and compost maturity, as confirmed by Zhou et al. (2022). The water layer surrounding compost particles affects microbial activity and the humification process by influencing nutrient availability (Huhe Jiang et al., 2017). It has been discovered that most microorganisms perform similar functions, including scavenging reactive oxygen species, metabolic flux transfer, osmotic protection, production of extracellular polymeric substances, and biofilm recombination, all of which help them adapt to environment changing (Joshua et al., 2007). However, these biochemical processes require substantial carbon and nutrient reserves within microbial cells, which are highly dependent on the physiological characteristics and metabolic levels of the microorganisms (Joshua et al., 2007; Szekely and Langenheder, 2017). The carbon source and nutrients in compost are mainly provided by raw materials. This may be the reason why compost experiments with different raw materials exhibit same microbiome - environment correlationship.

### 4.4 Fungal OTUs assigned to functional guild

Through FUNguid analysis, it was discovered that saprotrophic fungi were the most abundant microbial types during the composting process. There was a general increase in the relative abundance of pathotrophic fungi in the day1. This was mainly because that most fungi respond to high temperature stress by increasing the production of heat-shock proteins and chaperones and initiating alternative metabolic pathways, which eventually leaded to the increase of the relative abundance of pathotrophic fungi (Abu Bakar et al., 2020). Fungi with undetermined functional types also accounted for a substantial proportion. Furthermore, some studies have shown that these fungi can accelerate nutrient mineralization (Schmidt et al., 2019). Some studies have found that the relative abundance of woody saprophytic fungi increases which improve the degradation of lignin during composting (Qiao et al., 2021). It was also found that the T3 treatment could enhance the relative abundance of endophyte which could improve the degradation of lignin. The differences in the above results may be caused by different addition. These results highlight the importance of further studying functional types of fungi to reduce the lignin content and enhance the maturation of compost.

## 5 Conclusion

Combined addition is more beneficial to promote compost maturation than single addition. Among them, lignin degradation rate (16.60%), HA (22.70 g/kg) and GI (110.99%) of combined addition were higher than those of other treatments. Different treatments had varying effects on microbial communities during composting. Correlation analysis exhibited that MC affected the fungal community structure (−0.740, *p* < 0.01). Fungal community structure played a vital role in the degradation of lignin (−0.952, *p* < 0.01) which affected the compost maturity (−1.093, *p* < 0.001). FUNGuild and correlation heatmap demonstrate that combined addition could enhance the relative abundance of endophyte which may had the potential to improve the degradation of lignin. Therefore, the combined addition of *Trichoderma harzianum* and *Phanerochaete chrysosporium* shows good performance on degradation of lignin, strengthen the humification process of composting, and increase the utilization efficiency of manure and the quality of compost. Future study will estimate whether there is antagonistic/cooperation interaction to better understand mechanisms underlying ecological roles of this dual fungal combination.

## Data Availability

The datasets presented in this study can be found in online repositories. The names of the repository/repositories and accession number(s) can be found in the article/Supplementary material.

## Footnotes

^1^ https://unite.ut.ee/
