# Supplementary figures and images for: Effects of Trichoderma harzianum combined with Phanerochaete chrysosporium on lignin degradation and humification during chicken manure and rice husk composting (part 2 of 2)
Source: Front Microbiol. 2025 Feb 28;16:1515931. doi: 10.3389/fmicb.2025.1515931 (PMC11906335; doi:10.3389/fmicb.2025.1515931)

PCoA bray\_curtis

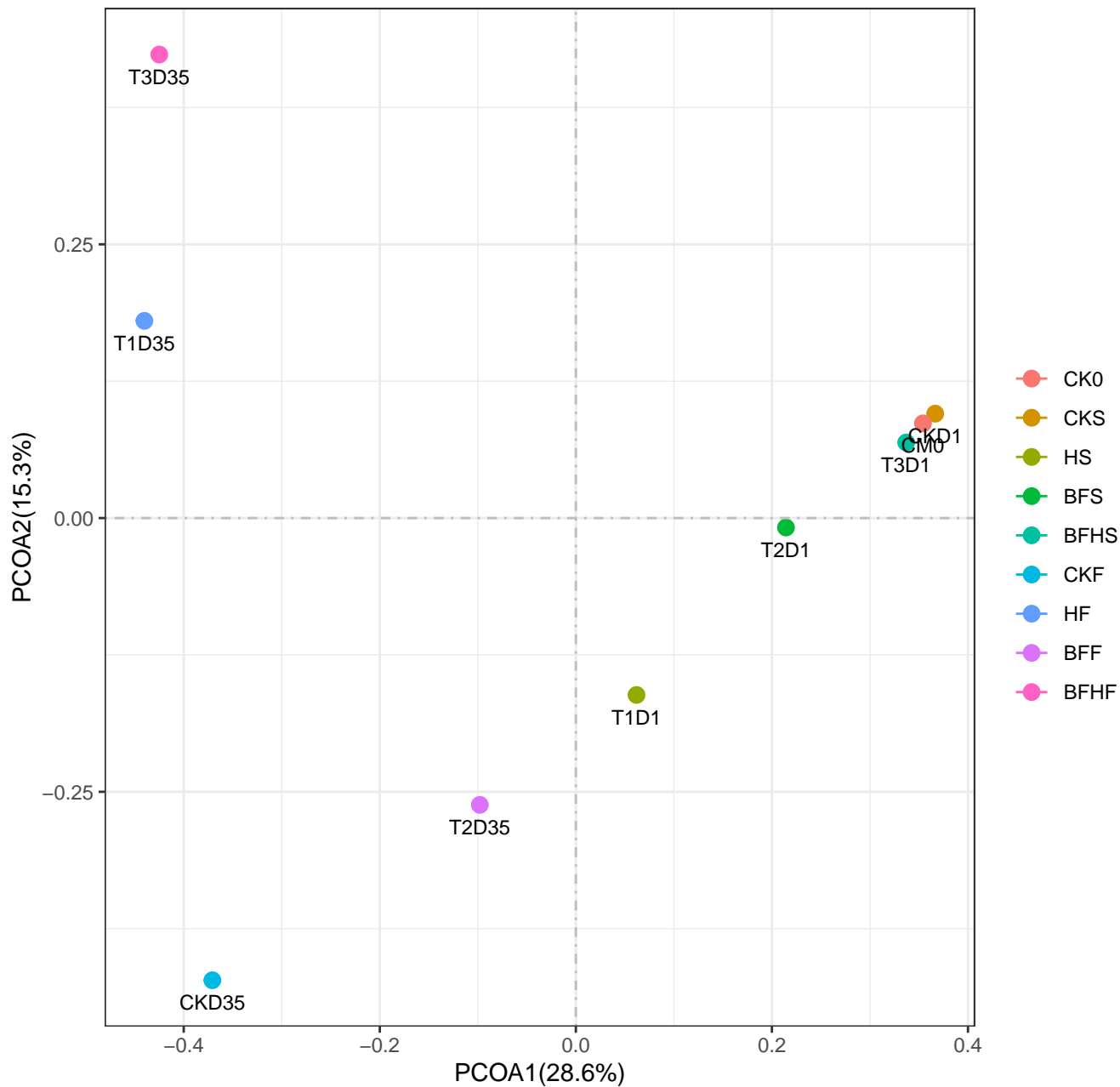

Supplement: Supplementary file 1 [file Data_Sheet_1.zip › 4.Beta_diversity/PCoA/bray_curtis_PCoA_name_cluster.pdf]

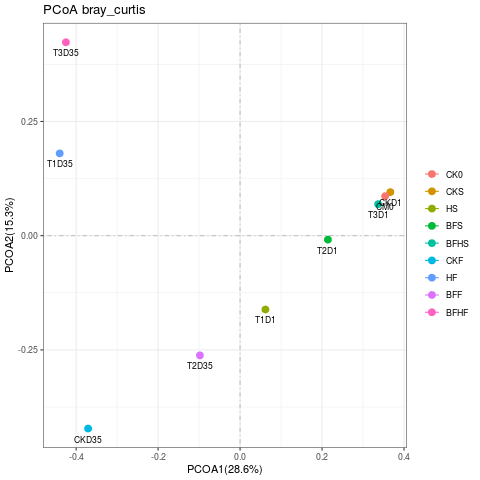

Supplement: Supplementary file 1 [file Data_Sheet_1.zip › 4.Beta_diversity/PCoA/bray_curtis_PCoA_name_cluster.png]

# jaccard\_PCoA1

Kruskal-Wallis,  $p = 0.4335$

group

|     |     |      |     |      |
|-----|-----|------|-----|------|
| CK0 | HS  | BFHS | HF  | BFHF |
| CKS | BFS | CKF  | BFF |      |

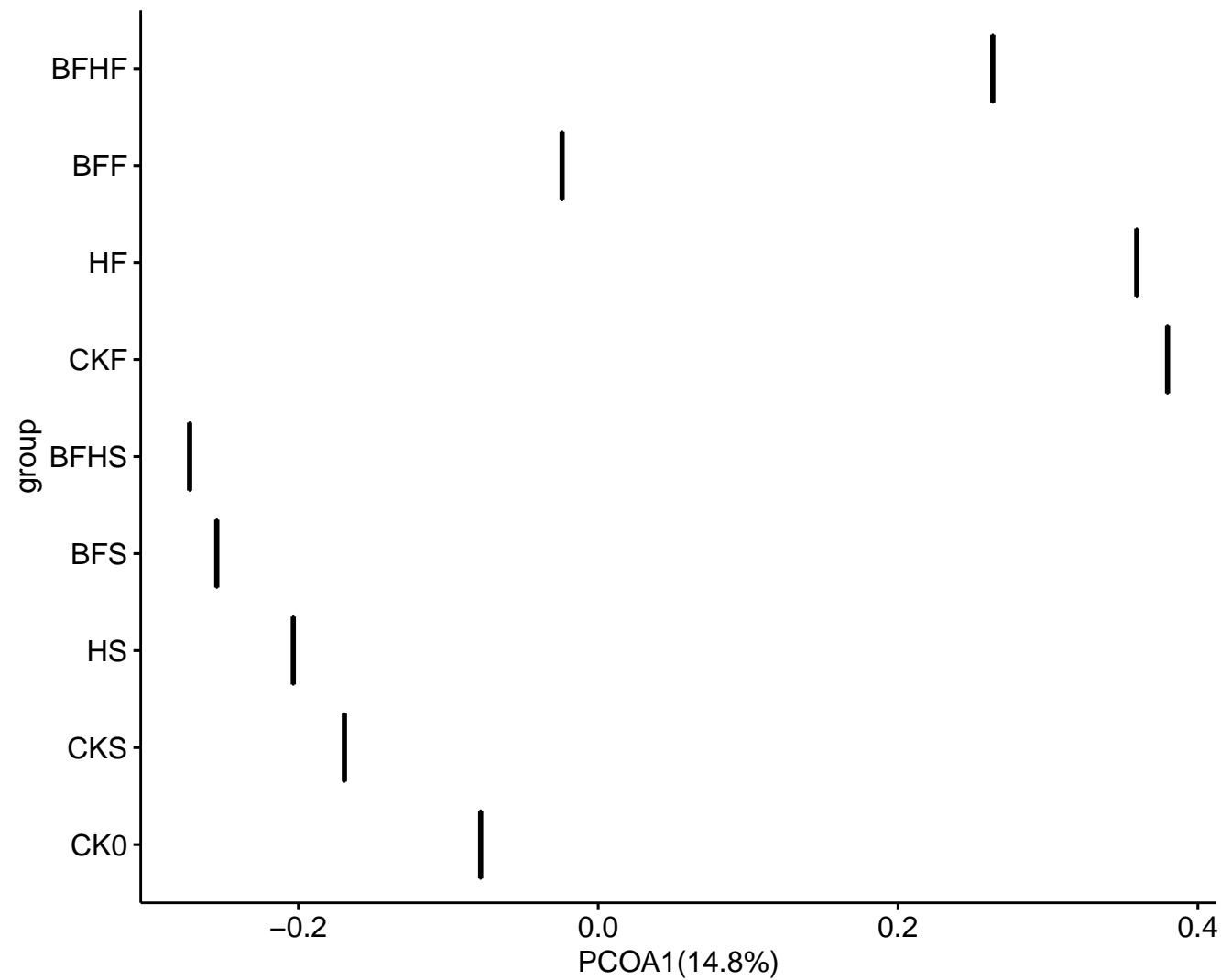

Supplement: Supplementary file 1 [file Data_Sheet_1.zip › 4.Beta_diversity/PCoA/jaccard.PCoA1.boxplot.pdf]

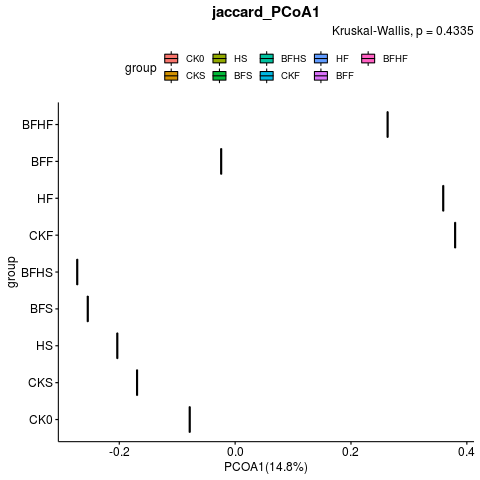

Supplement: Supplementary file 1 [file Data_Sheet_1.zip › 4.Beta_diversity/PCoA/jaccard.PCoA1.boxplot.png]

# jaccard\_PCoA2

Kruskal-Wallis,  $p = 0.4335$

group

|     |     |      |     |      |
|-----|-----|------|-----|------|
| CK0 | HS  | BFHS | HF  | BFHF |
| CKS | BFS | CKF  | BFF |      |

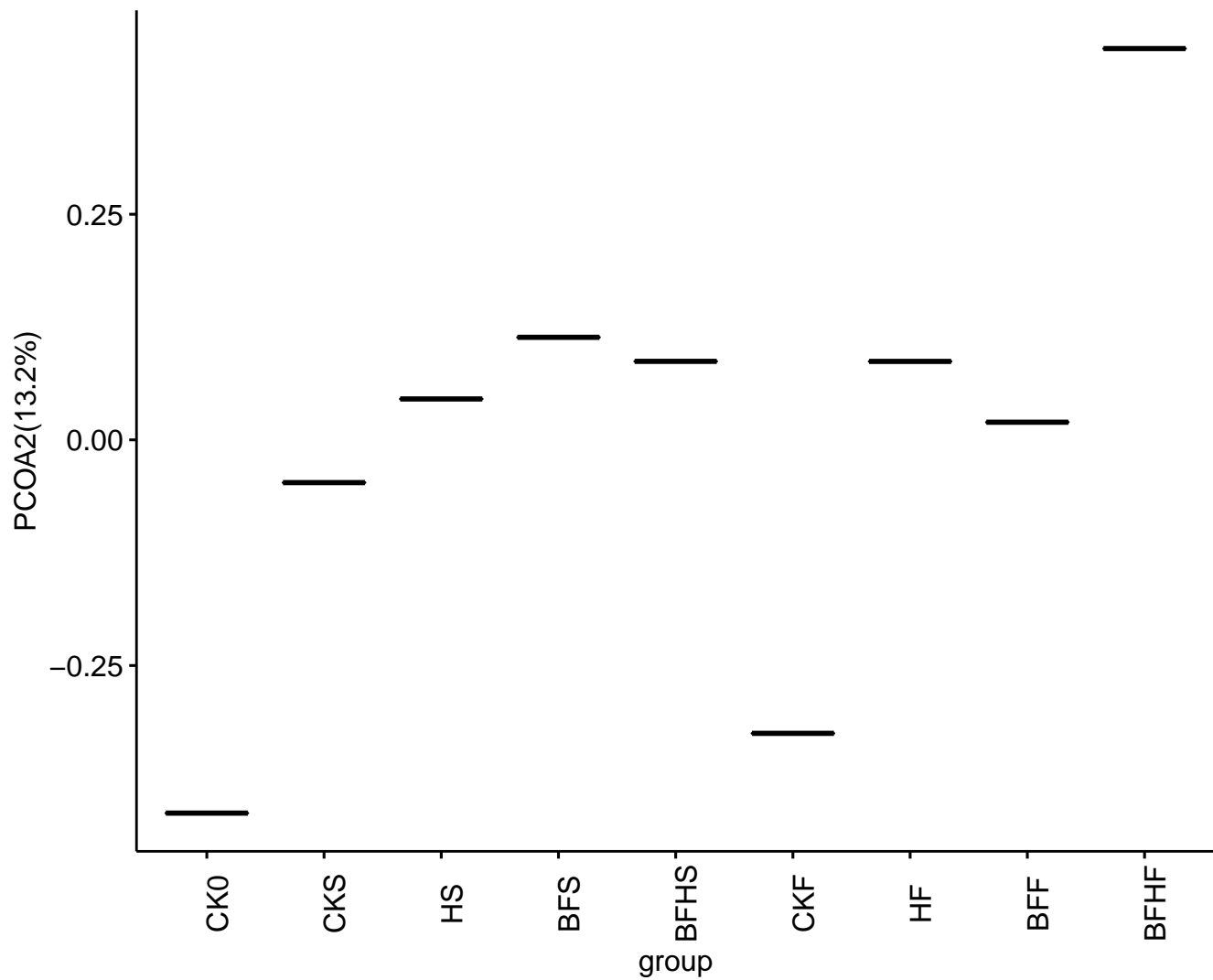

Supplement: Supplementary file 1 [file Data_Sheet_1.zip › 4.Beta_diversity/PCoA/jaccard.PCoA2.boxplot.pdf]

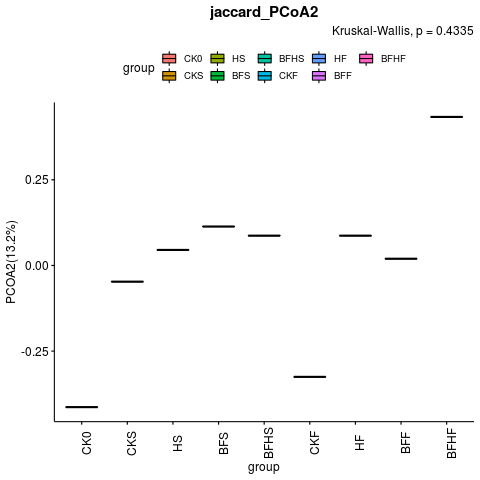

Supplement: Supplementary file 1 [file Data_Sheet_1.zip › 4.Beta_diversity/PCoA/jaccard.PCoA2.boxplot.png]

PCoA jaccard

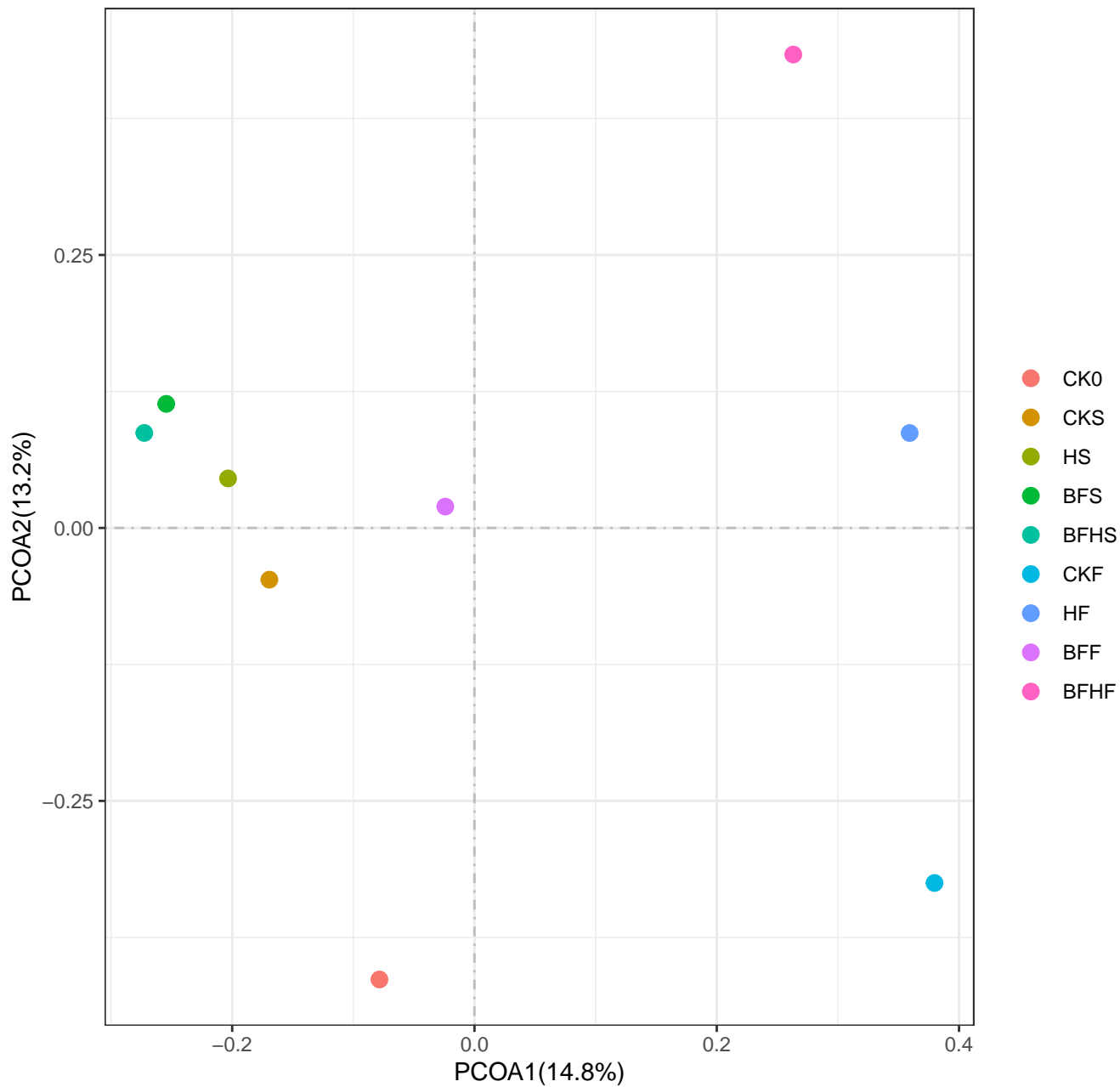

Supplement: Supplementary file 1 [file Data_Sheet_1.zip › 4.Beta_diversity/PCoA/jaccard_PCoA.pdf]

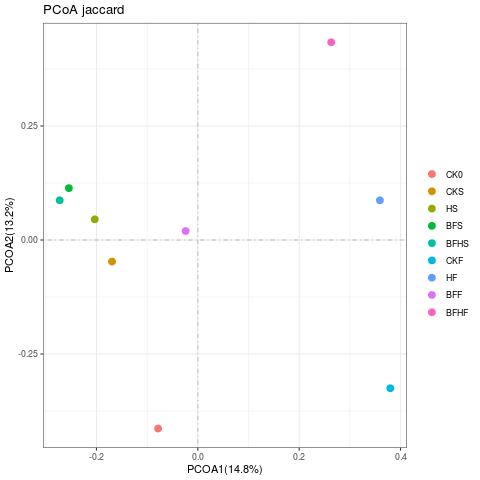

Supplement: Supplementary file 1 [file Data_Sheet_1.zip › 4.Beta_diversity/PCoA/jaccard_PCoA.png]

PCoA jaccard

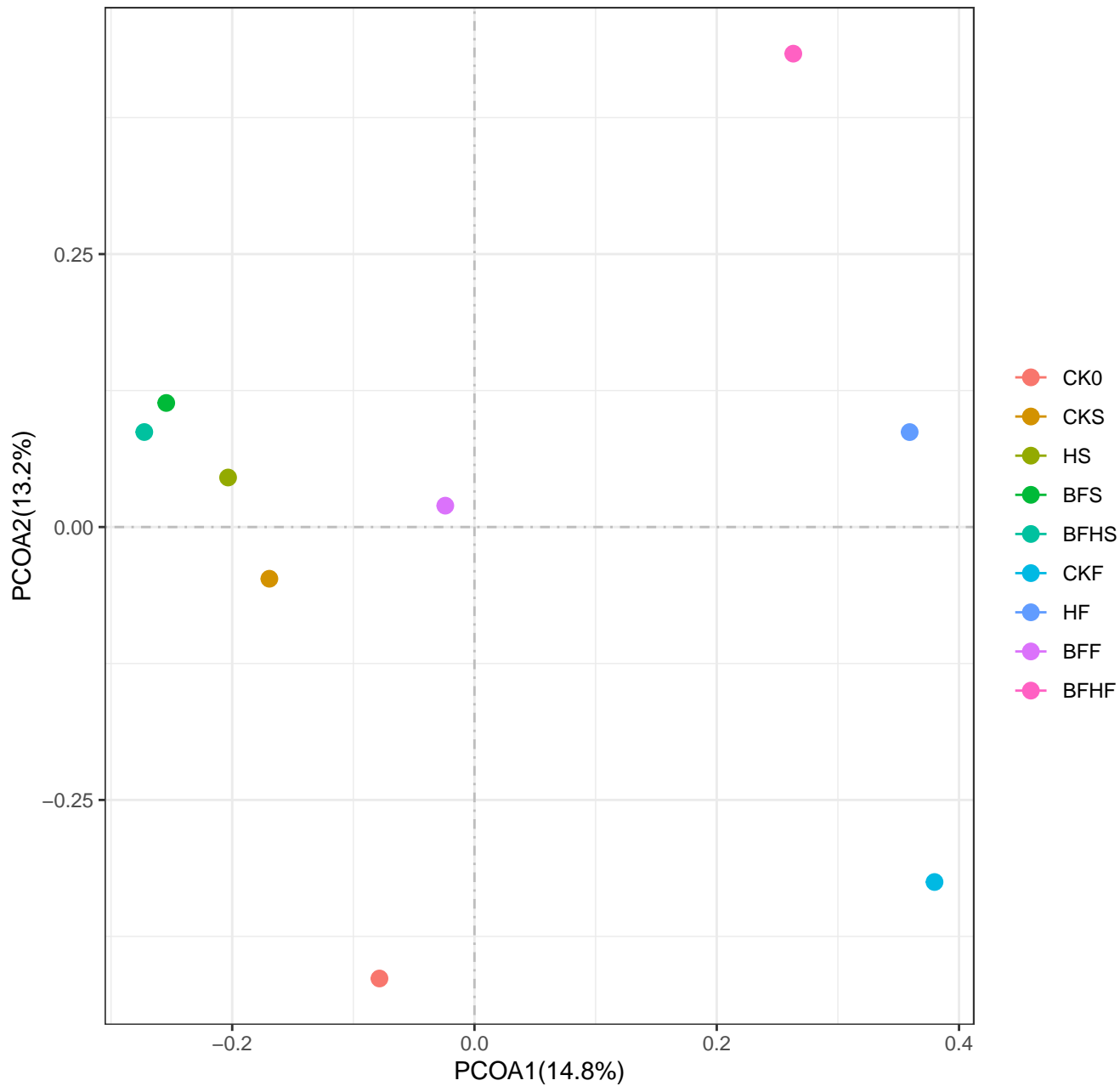

Supplement: Supplementary file 1 [file Data_Sheet_1.zip › 4.Beta_diversity/PCoA/jaccard_PCoA_cluster.pdf]

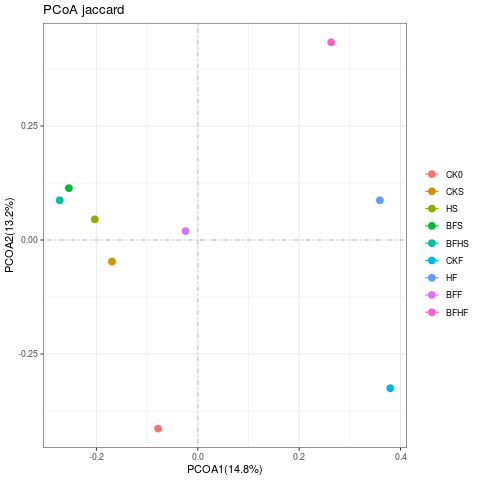

Supplement: Supplementary file 1 [file Data_Sheet_1.zip › 4.Beta_diversity/PCoA/jaccard_PCoA_cluster.png]

# PCoA jaccard

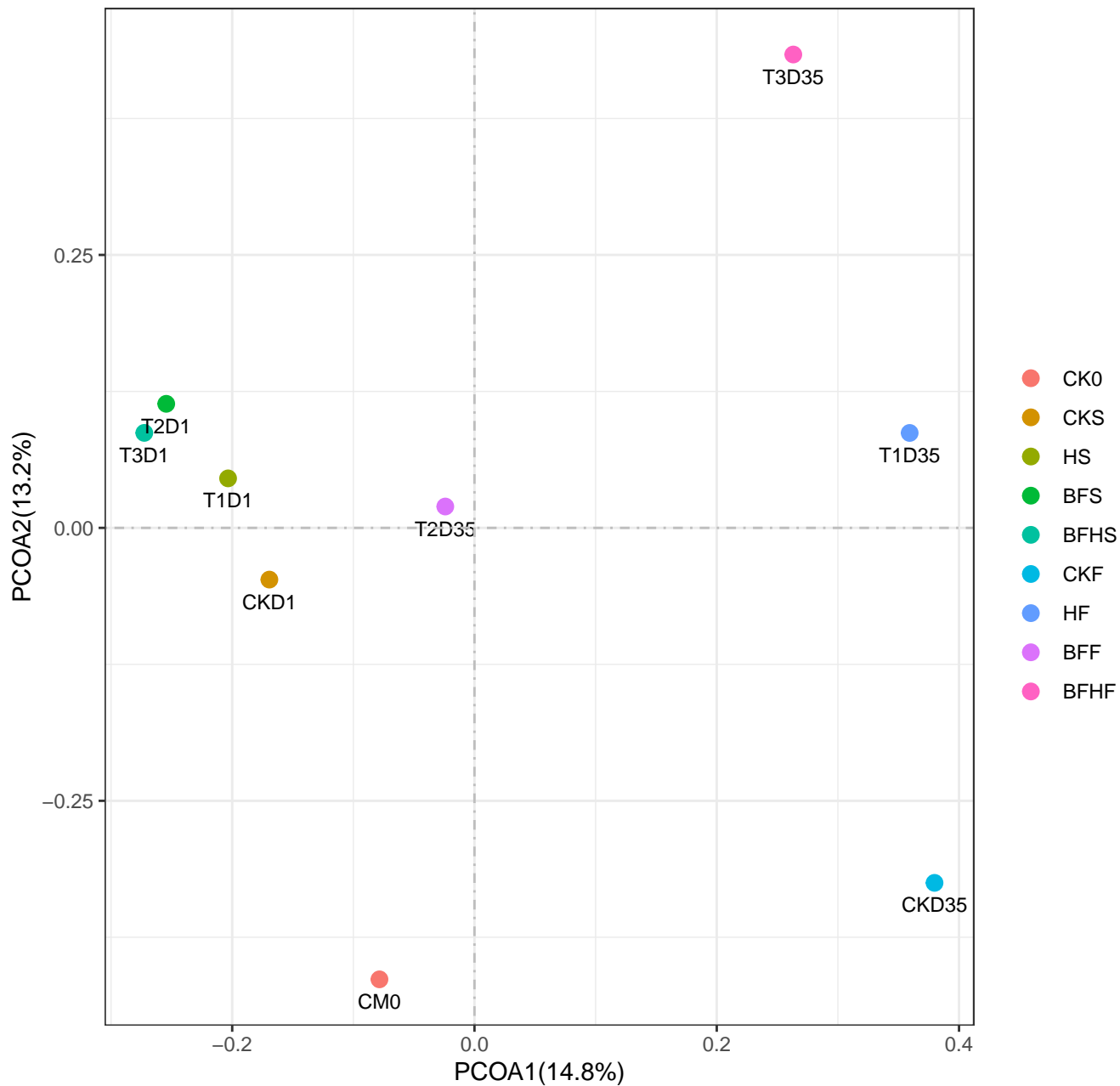

Supplement: Supplementary file 1 [file Data_Sheet_1.zip › 4.Beta_diversity/PCoA/jaccard_PCoA_name.pdf]

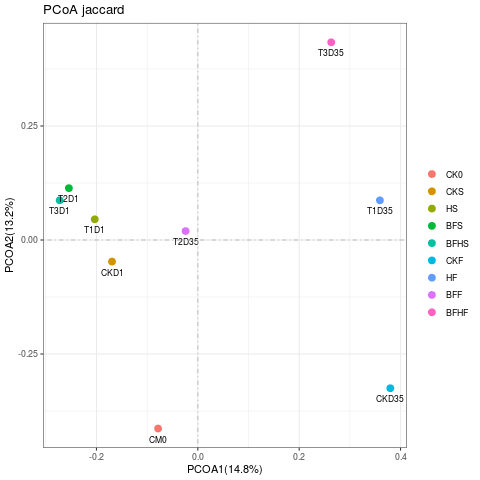

Supplement: Supplementary file 1 [file Data_Sheet_1.zip › 4.Beta_diversity/PCoA/jaccard_PCoA_name.png]

# PCoA jaccard

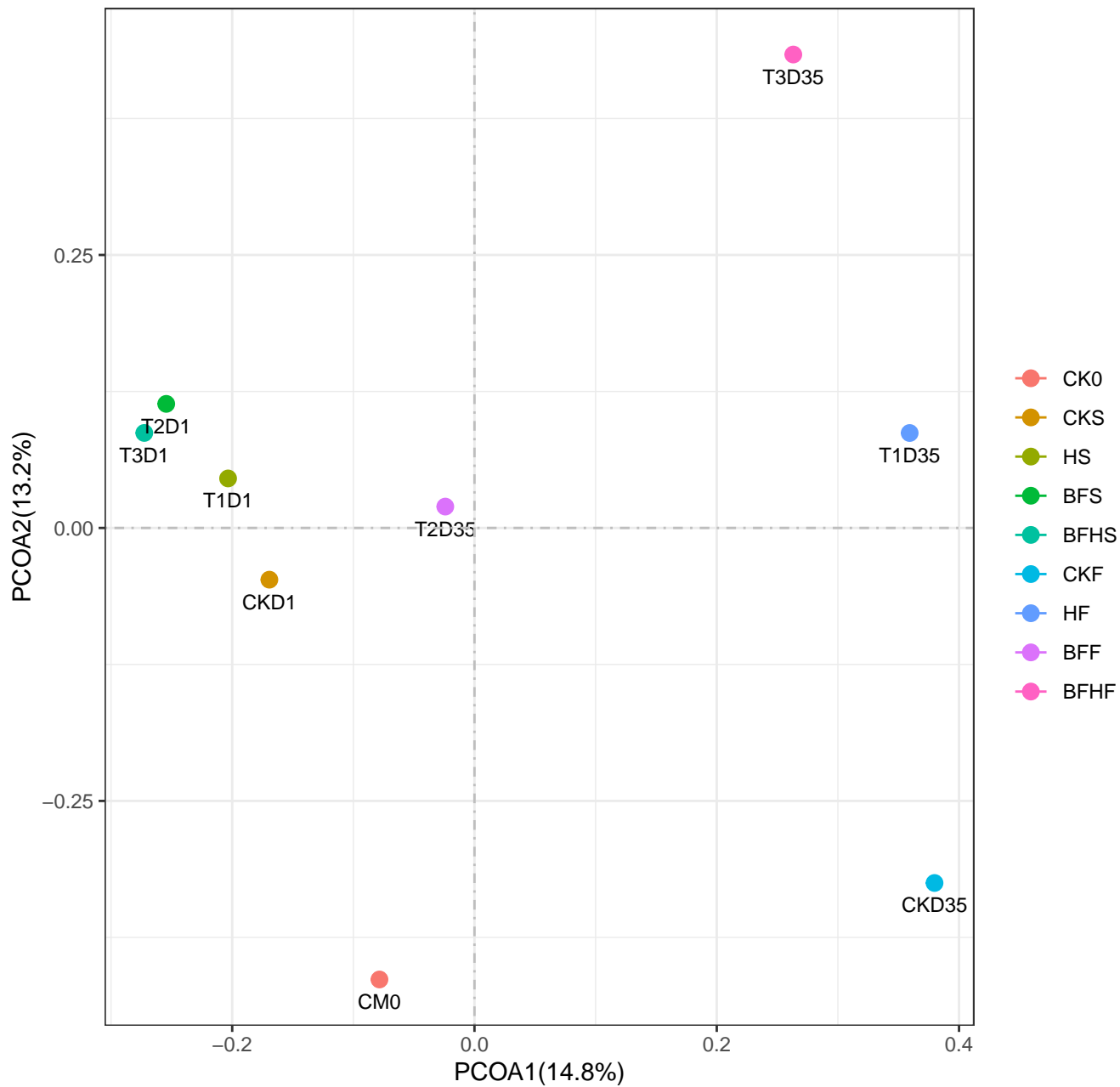

Supplement: Supplementary file 1 [file Data_Sheet_1.zip › 4.Beta_diversity/PCoA/jaccard_PCoA_name_cluster.pdf]

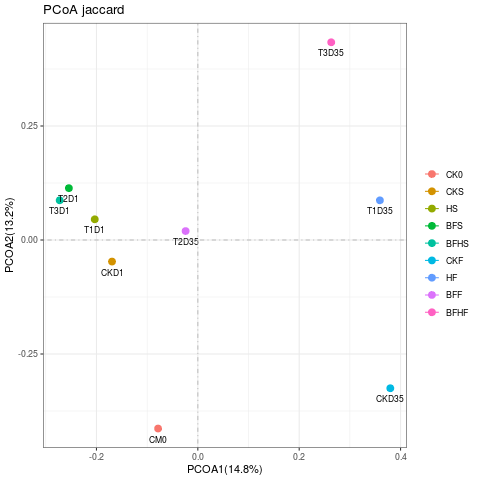

Supplement: Supplementary file 1 [file Data_Sheet_1.zip › 4.Beta_diversity/PCoA/jaccard_PCoA_name_cluster.png]

# unweighted\_unifrac\_PCoA1

Kruskal-Wallis,  $p = 0.4335$

group

|     |     |      |     |      |
|-----|-----|------|-----|------|
| CK0 | HS  | BFHS | HF  | BFHF |
| CKS | BFS | CKF  | BFF |      |

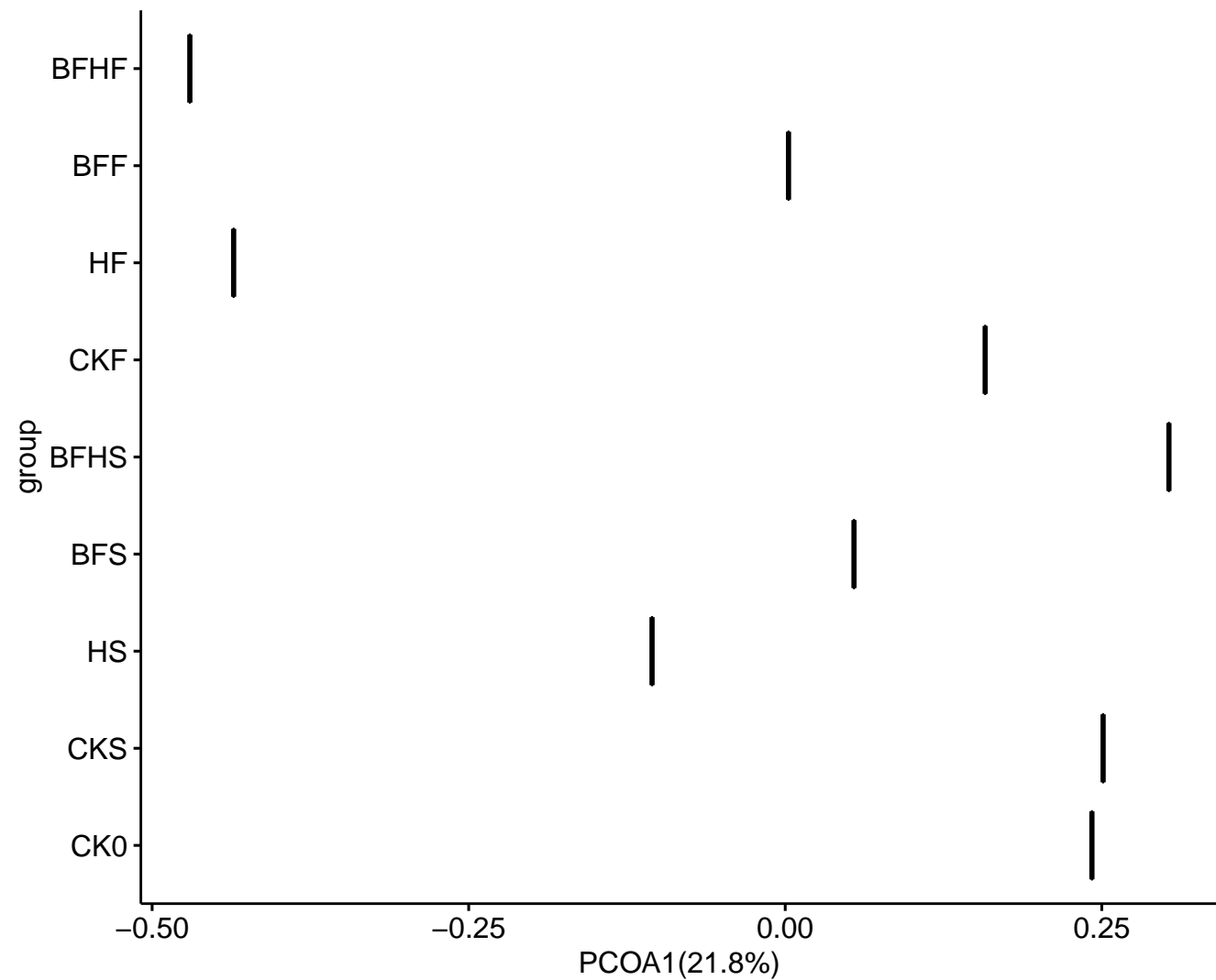

Supplement: Supplementary file 1 [file Data_Sheet_1.zip › 4.Beta_diversity/PCoA/unweighted_unifrac.PCoA1.boxplot.pdf]

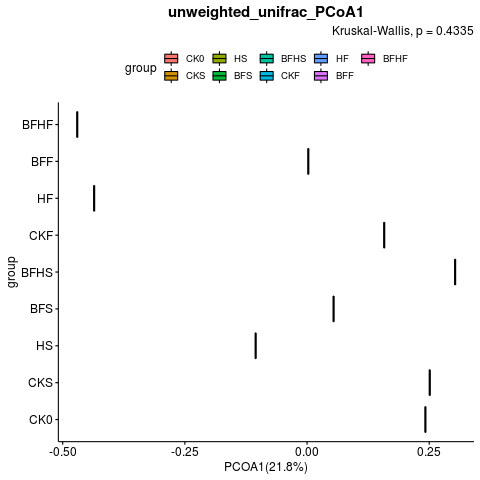

Supplement: Supplementary file 1 [file Data_Sheet_1.zip › 4.Beta_diversity/PCoA/unweighted_unifrac.PCoA1.boxplot.png]

# unweighted\_unifrac\_PCoA2

Kruskal-Wallis,  $p = 0.4335$

group

|     |     |      |     |      |
|-----|-----|------|-----|------|
| CK0 | HS  | BFHS | HF  | BFHF |
| CKS | BFS | CKF  | BFF |      |

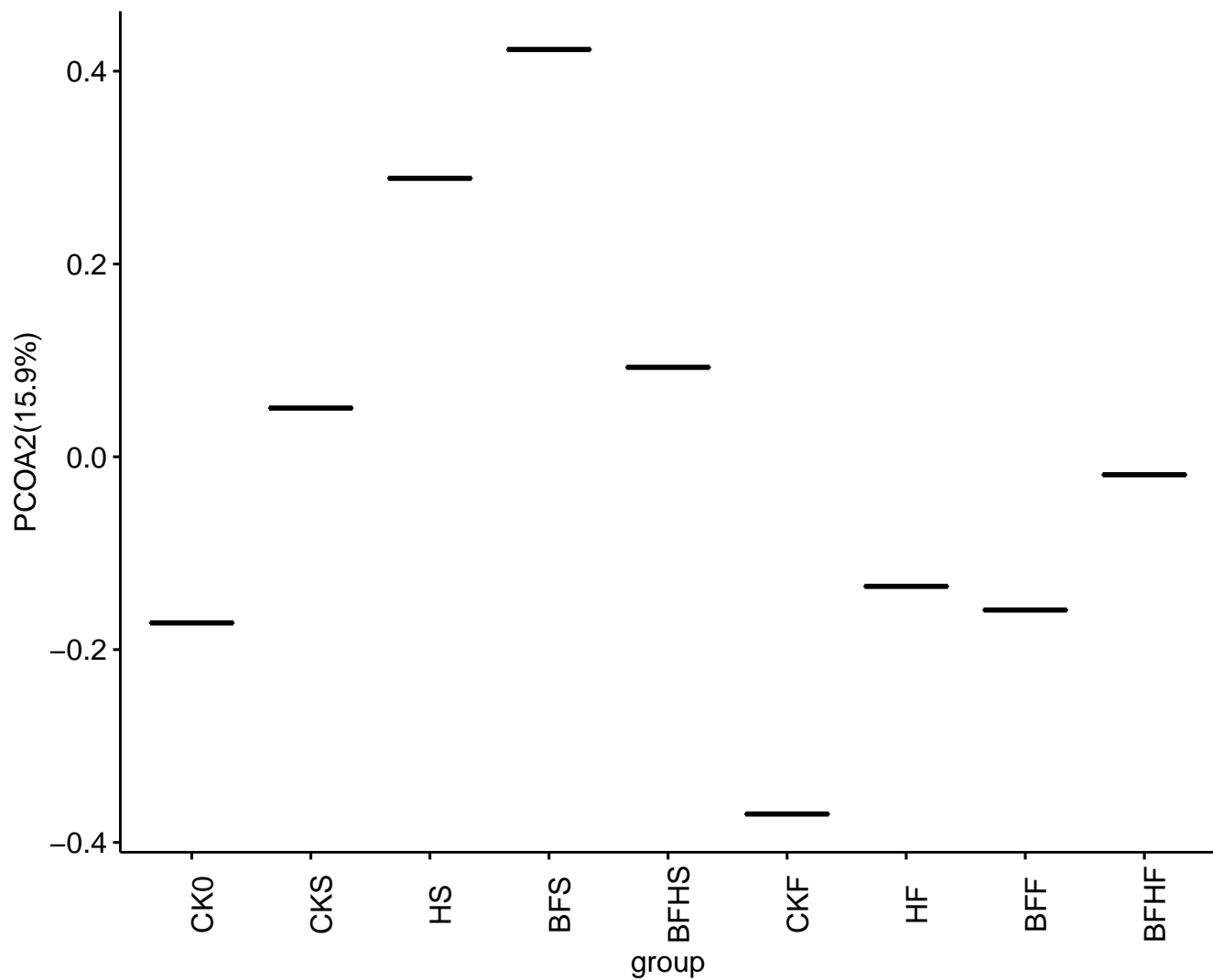

Supplement: Supplementary file 1 [file Data_Sheet_1.zip › 4.Beta_diversity/PCoA/unweighted_unifrac.PCoA2.boxplot.pdf]

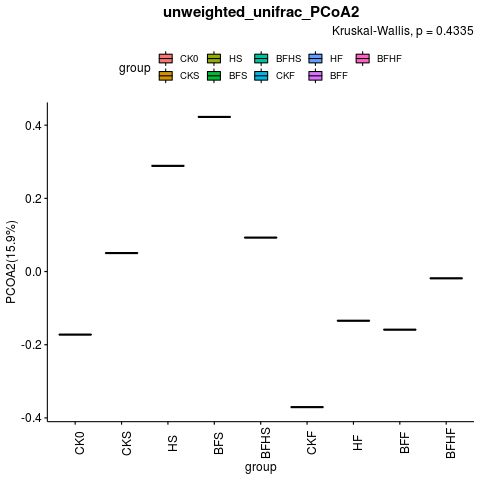

Supplement: Supplementary file 1 [file Data_Sheet_1.zip › 4.Beta_diversity/PCoA/unweighted_unifrac.PCoA2.boxplot.png]

PCoA unweighted\_unifrac

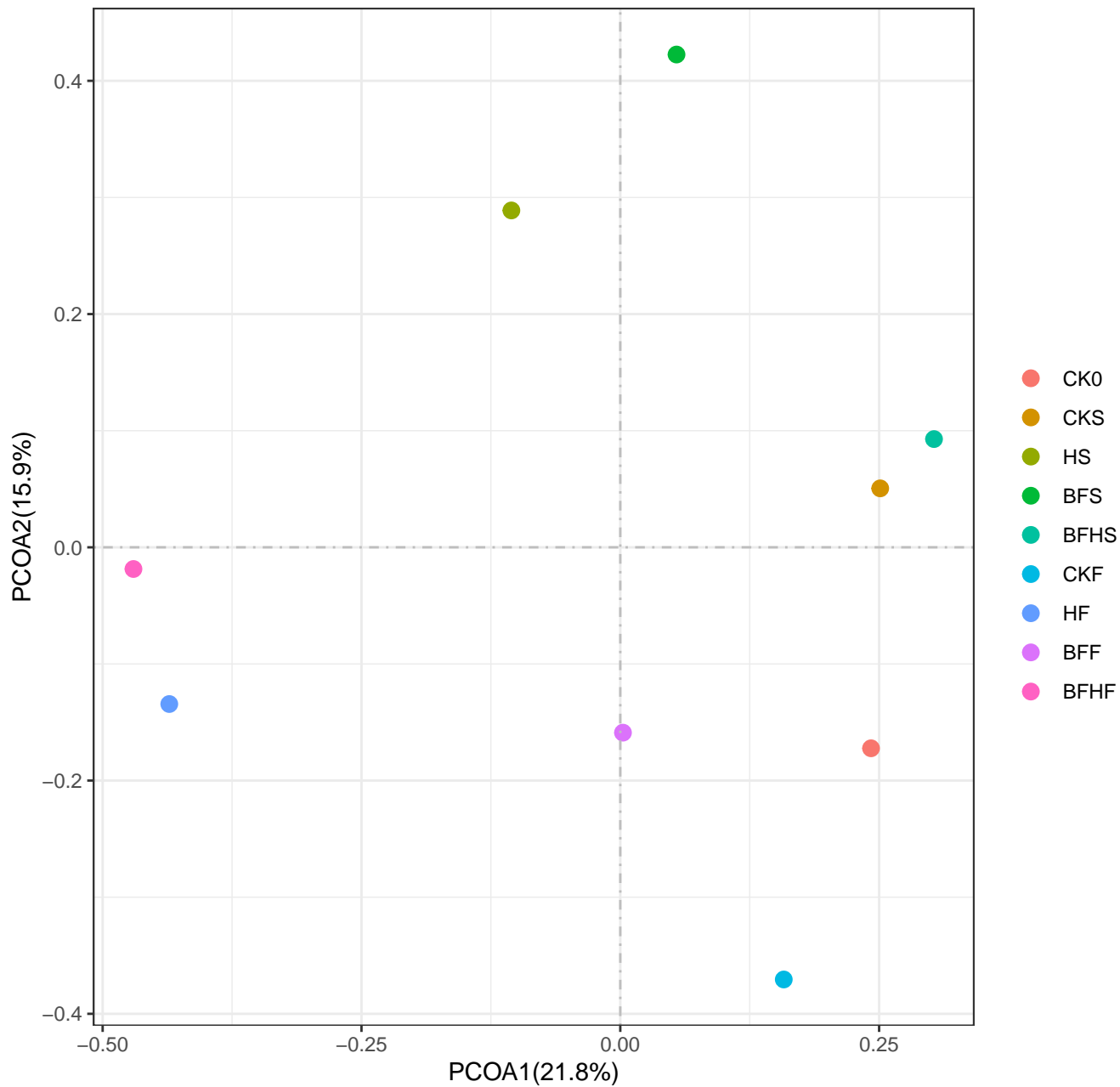

Supplement: Supplementary file 1 [file Data_Sheet_1.zip › 4.Beta_diversity/PCoA/unweighted_unifrac_PCoA.pdf]

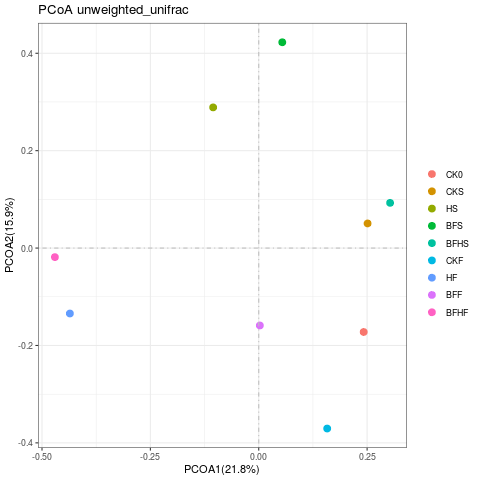

Supplement: Supplementary file 1 [file Data_Sheet_1.zip › 4.Beta_diversity/PCoA/unweighted_unifrac_PCoA.png]

PCoA unweighted\_unifrac

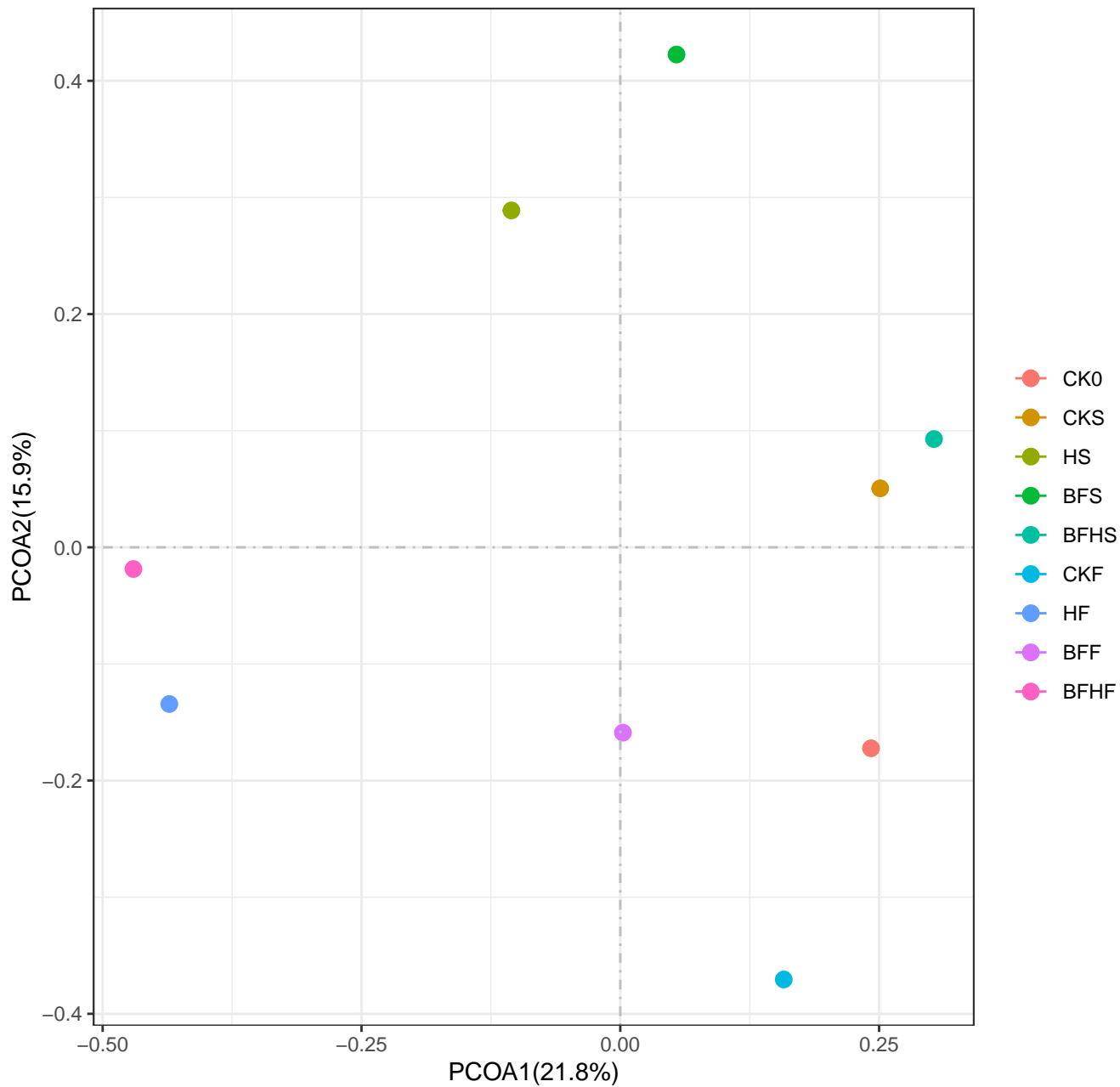

Supplement: Supplementary file 1 [file Data_Sheet_1.zip › 4.Beta_diversity/PCoA/unweighted_unifrac_PCoA_cluster.pdf]

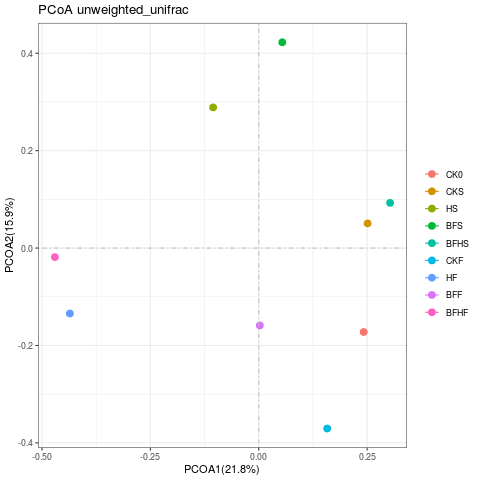

Supplement: Supplementary file 1 [file Data_Sheet_1.zip › 4.Beta_diversity/PCoA/unweighted_unifrac_PCoA_cluster.png]

PCoA unweighted\_unifrac

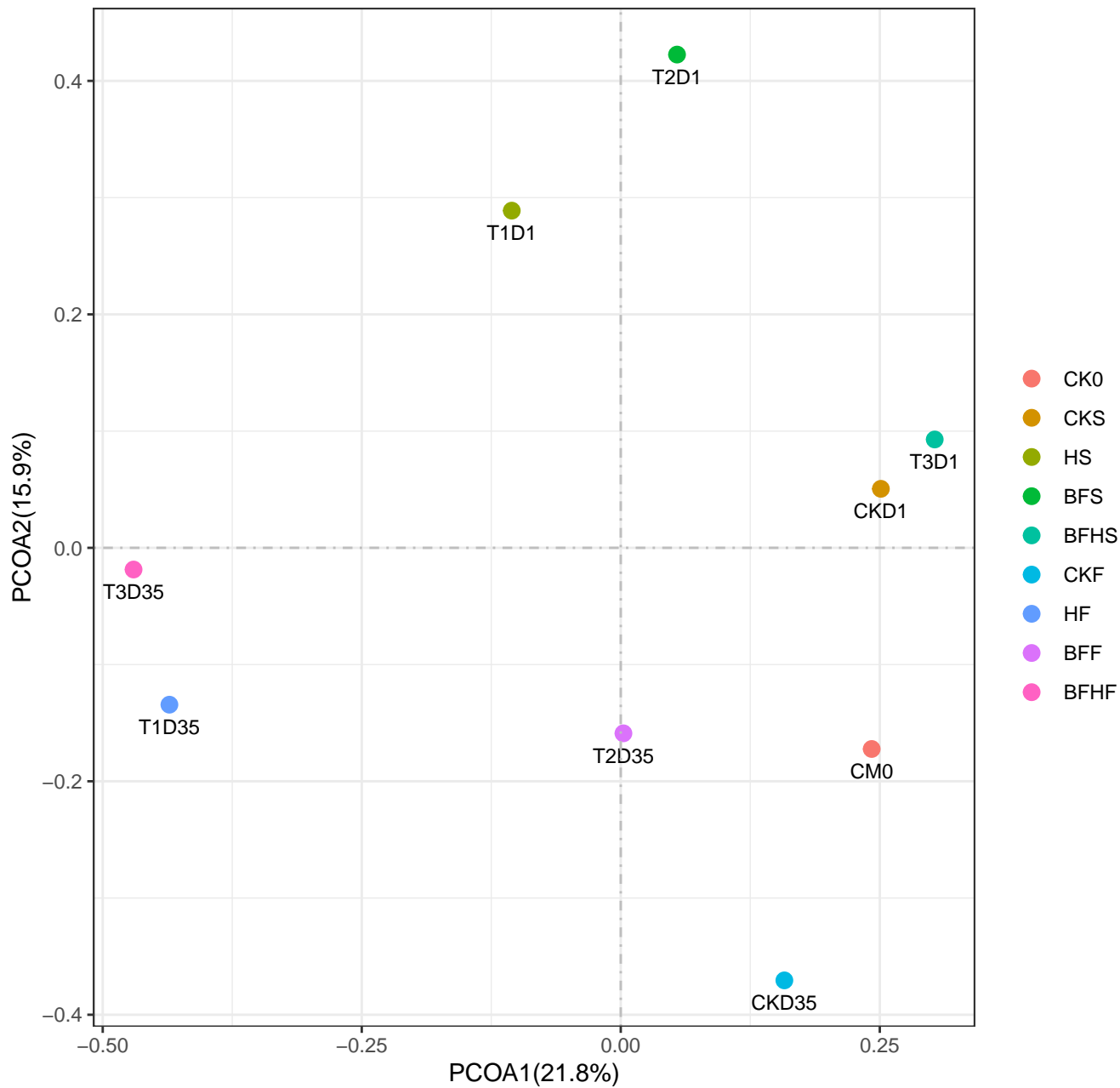

Supplement: Supplementary file 1 [file Data_Sheet_1.zip › 4.Beta_diversity/PCoA/unweighted_unifrac_PCoA_name.pdf]

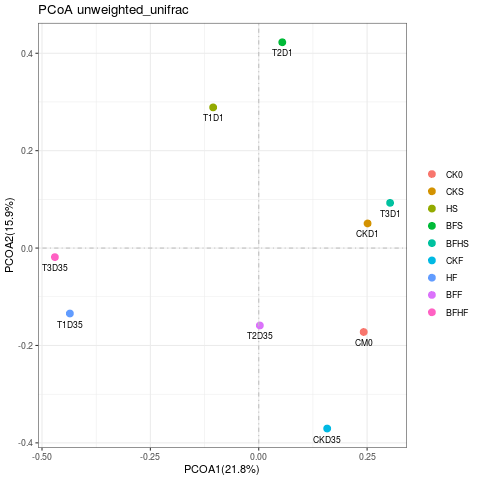

Supplement: Supplementary file 1 [file Data_Sheet_1.zip › 4.Beta_diversity/PCoA/unweighted_unifrac_PCoA_name.png]

PCoA unweighted\_unifrac

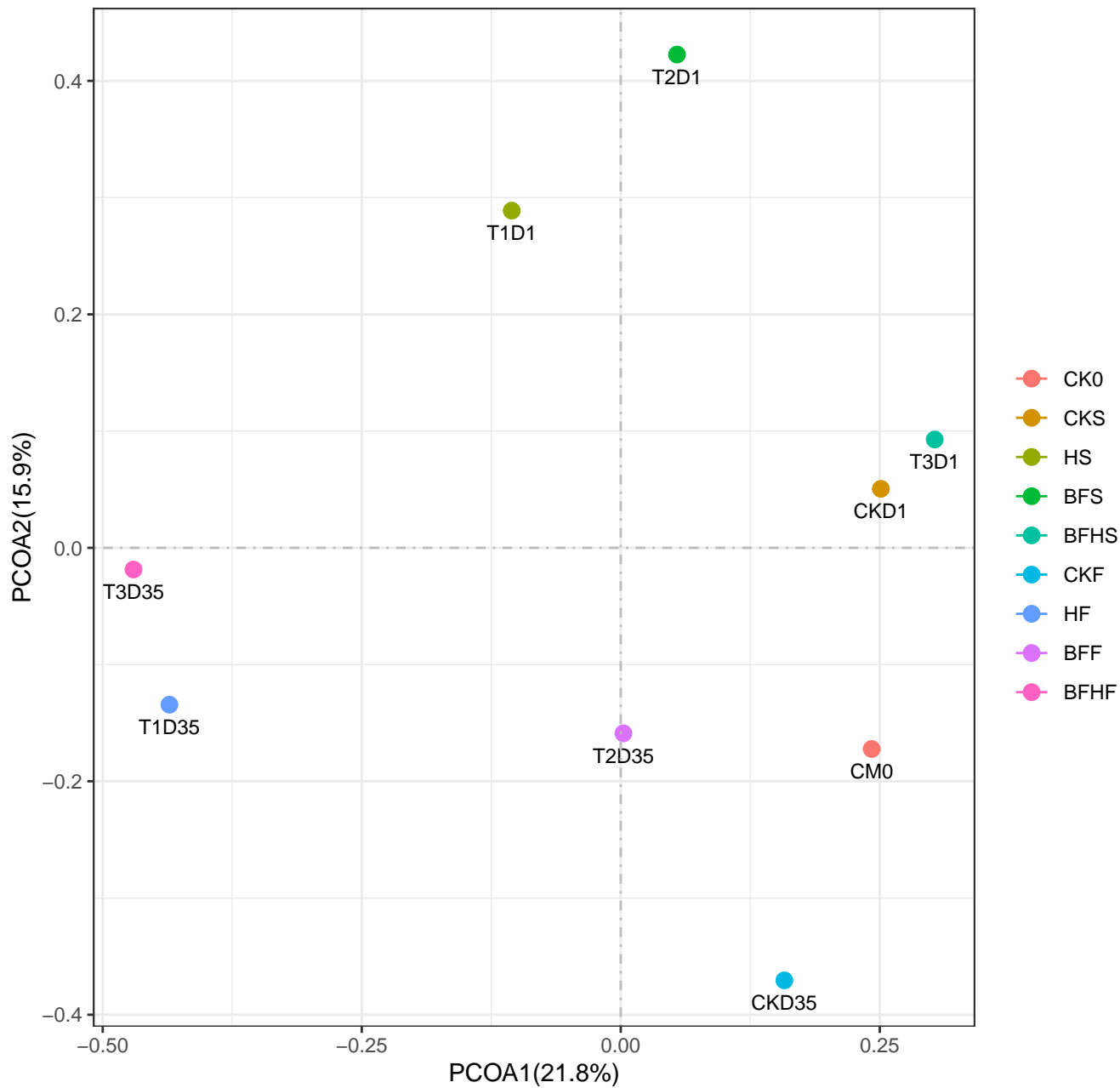

Supplement: Supplementary file 1 [file Data_Sheet_1.zip › 4.Beta_diversity/PCoA/unweighted_unifrac_PCoA_name_cluster.pdf]

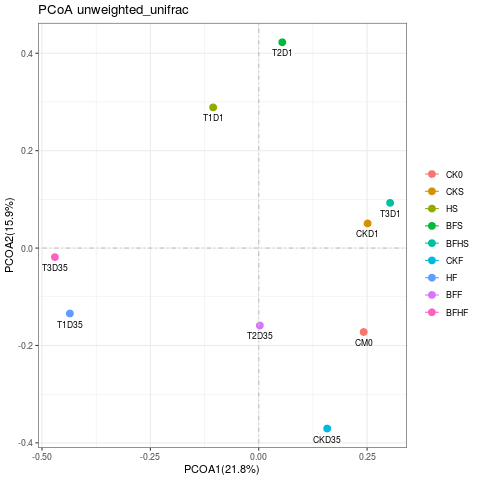

Supplement: Supplementary file 1 [file Data_Sheet_1.zip › 4.Beta_diversity/PCoA/unweighted_unifrac_PCoA_name_cluster.png]

# weighted\_unifrac\_PCoA1

Kruskal-Wallis,  $p = 0.4335$

group

|     |     |      |     |      |
|-----|-----|------|-----|------|
| CK0 | HS  | BFHS | HF  | BFHF |
| CKS | BFS | CKF  | BFF |      |

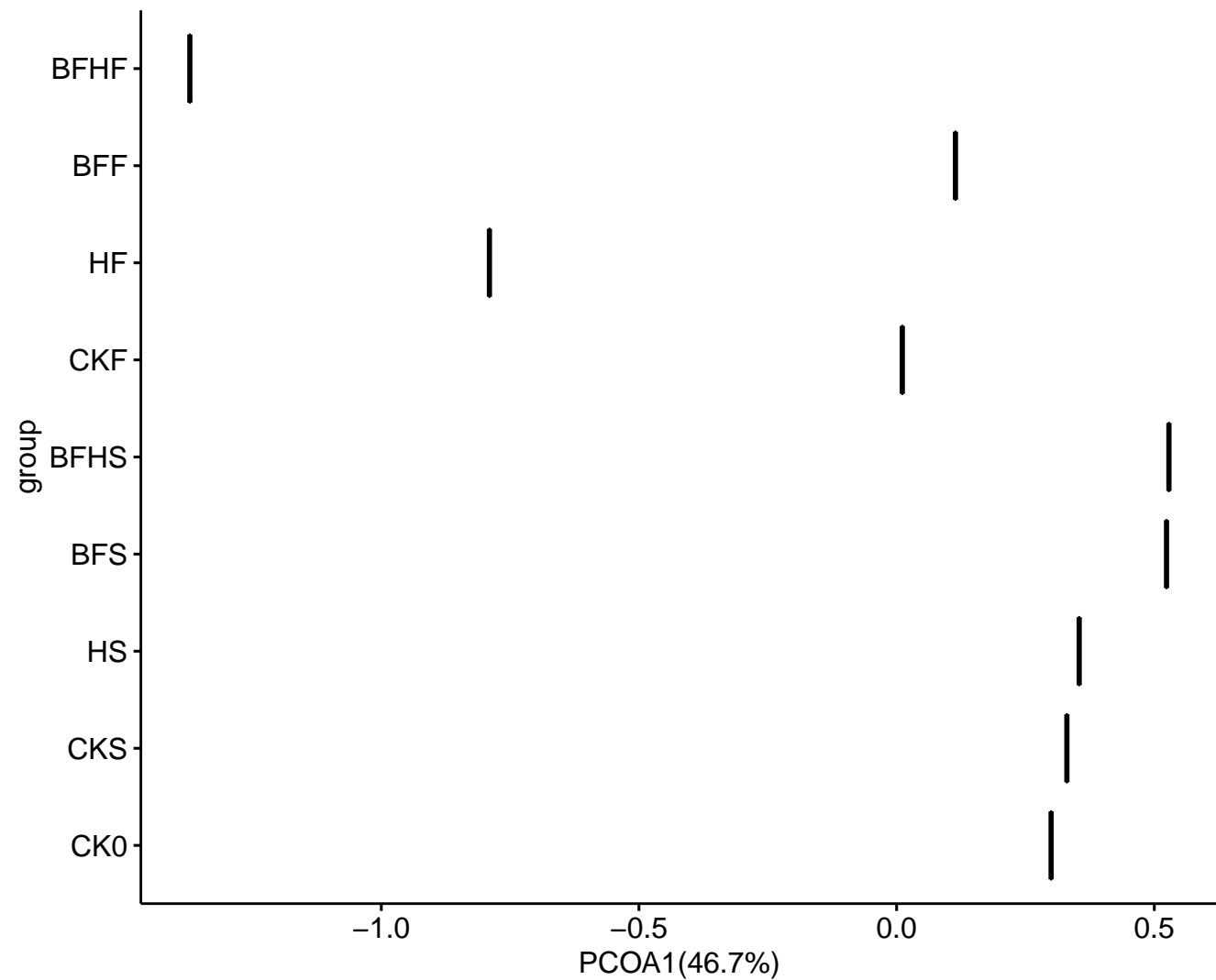

Supplement: Supplementary file 1 [file Data_Sheet_1.zip › 4.Beta_diversity/PCoA/weighted_unifrac.PCoA1.boxplot.pdf]

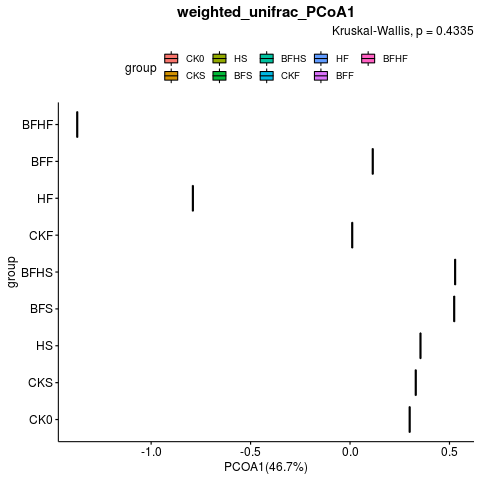

Supplement: Supplementary file 1 [file Data_Sheet_1.zip › 4.Beta_diversity/PCoA/weighted_unifrac.PCoA1.boxplot.png]

# weighted\_unifrac\_PCoA2

Kruskal-Wallis,  $p = 0.4335$

group

|     |     |      |     |      |
|-----|-----|------|-----|------|
| CK0 | HS  | BFHS | HF  | BFHF |
| CKS | BFS | CKF  | BFF |      |

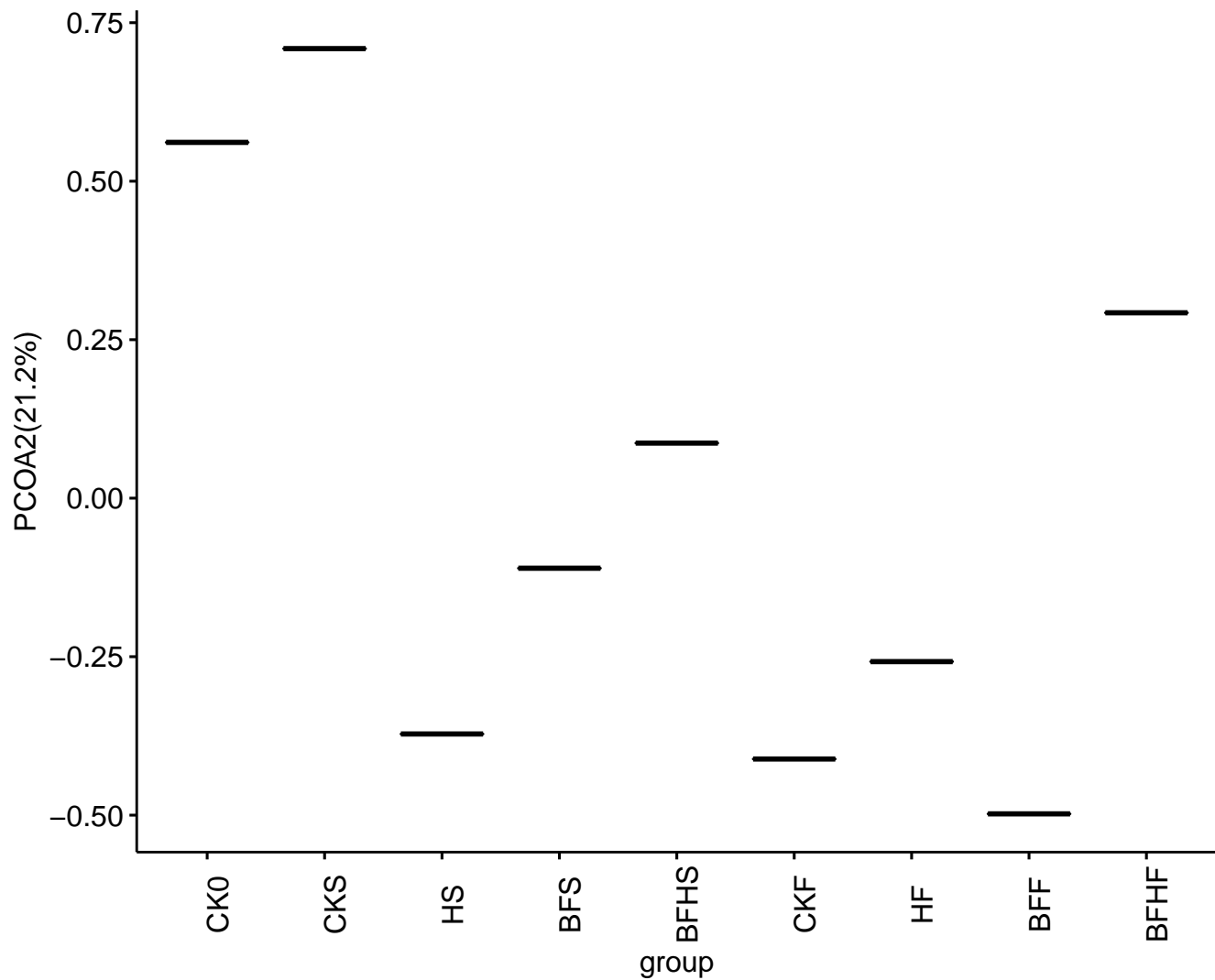

Supplement: Supplementary file 1 [file Data_Sheet_1.zip › 4.Beta_diversity/PCoA/weighted_unifrac.PCoA2.boxplot.pdf]

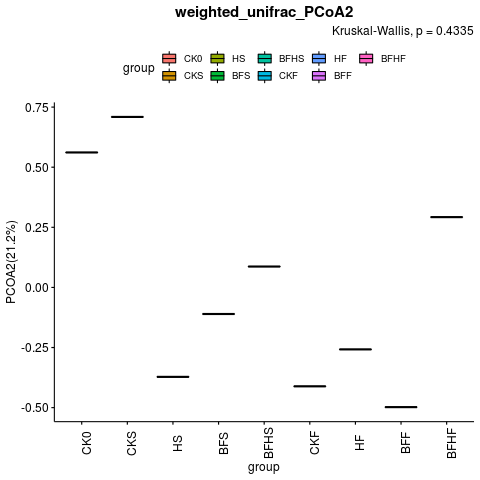

Supplement: Supplementary file 1 [file Data_Sheet_1.zip › 4.Beta_diversity/PCoA/weighted_unifrac.PCoA2.boxplot.png]

PCoA weighted\_unifrac

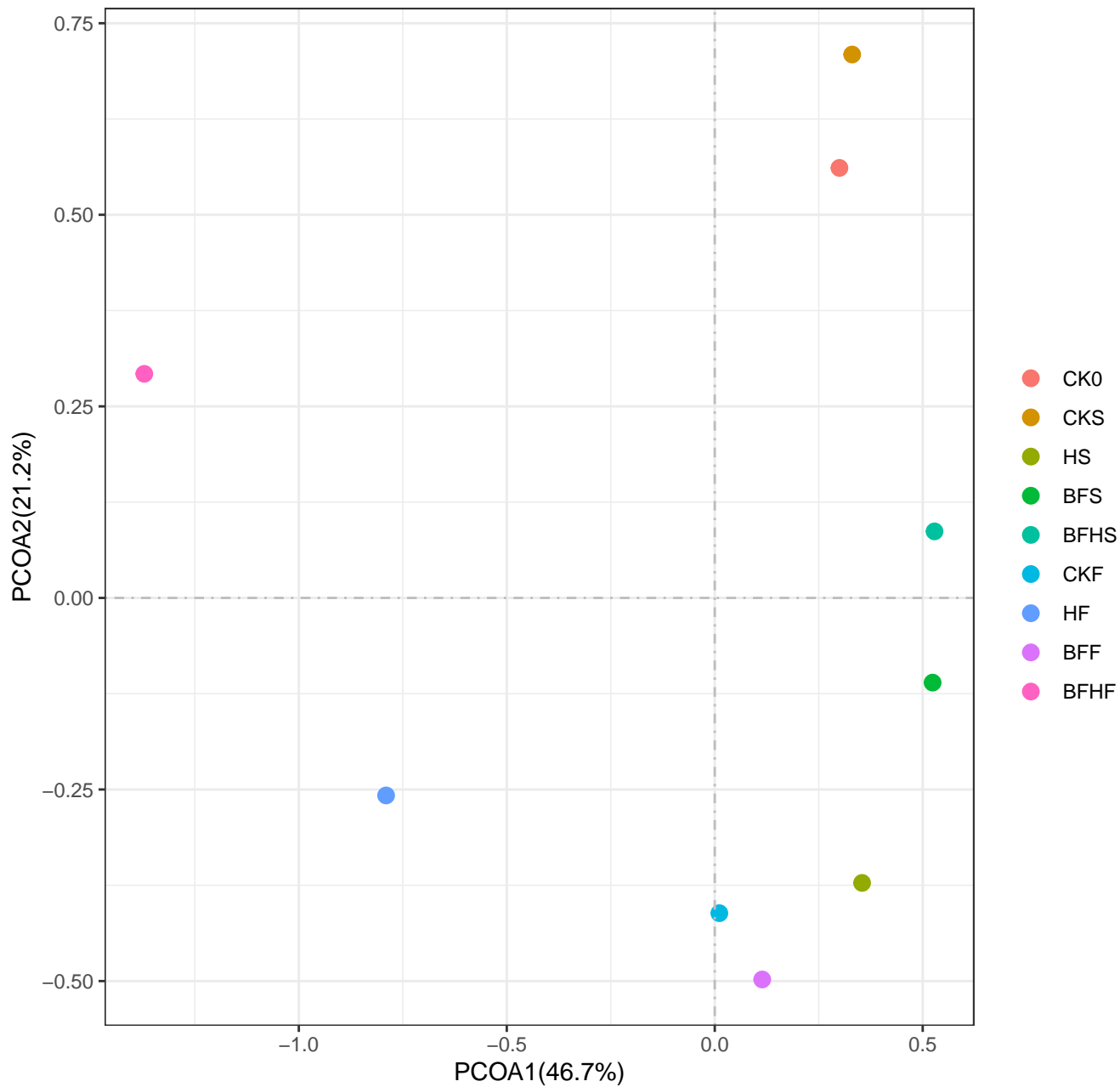

Supplement: Supplementary file 1 [file Data_Sheet_1.zip › 4.Beta_diversity/PCoA/weighted_unifrac_PCoA.pdf]

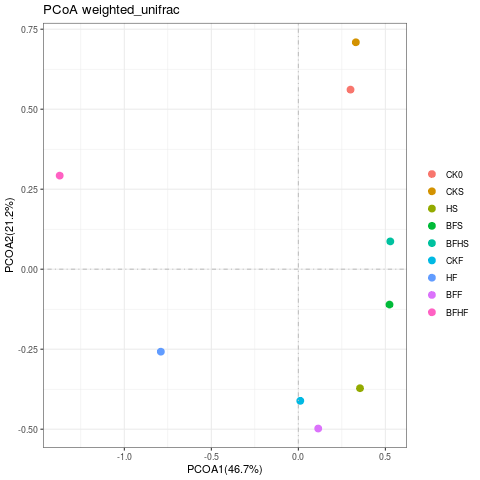

Supplement: Supplementary file 1 [file Data_Sheet_1.zip › 4.Beta_diversity/PCoA/weighted_unifrac_PCoA.png]

PCoA weighted\_unifrac

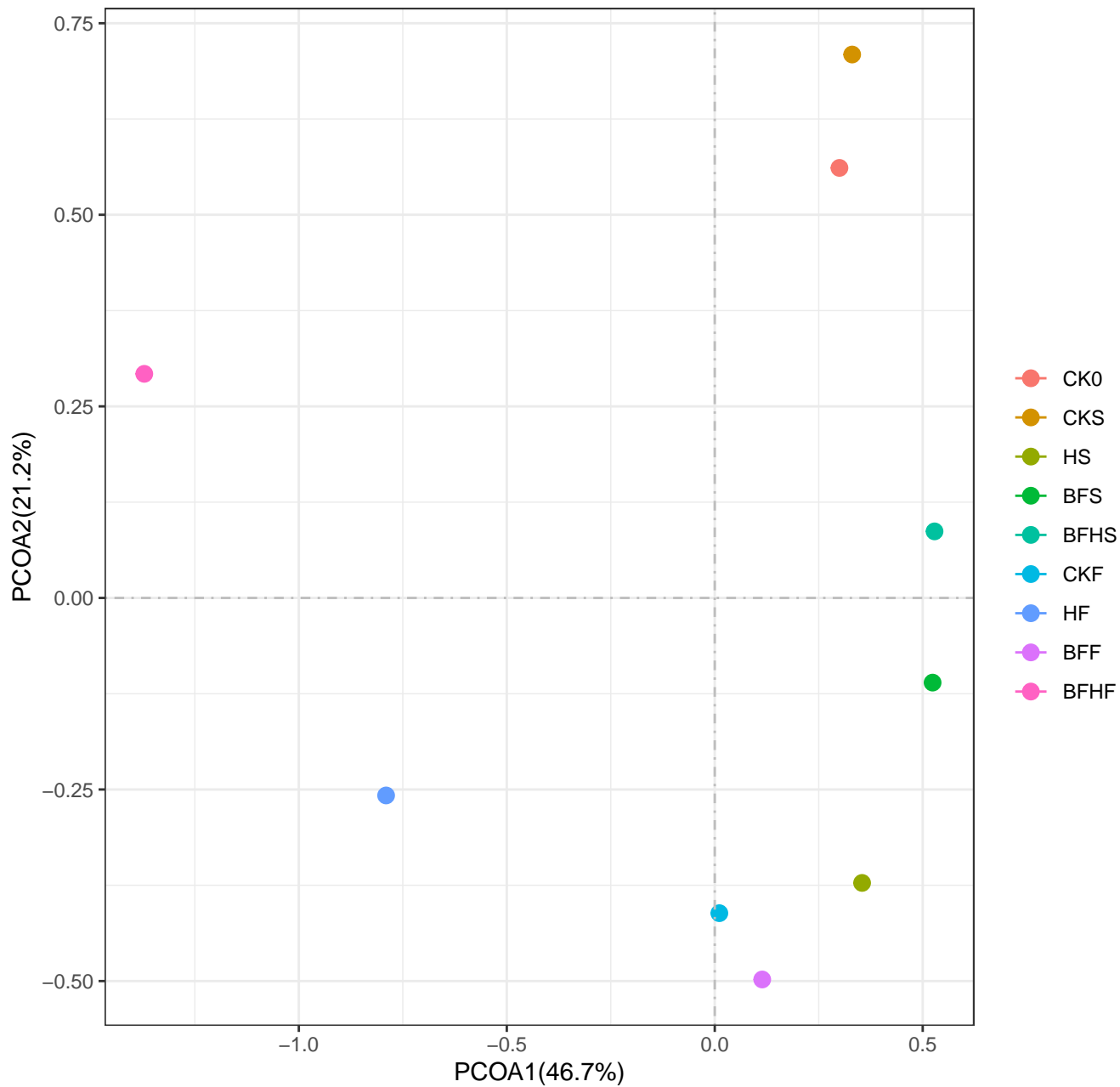

Supplement: Supplementary file 1 [file Data_Sheet_1.zip › 4.Beta_diversity/PCoA/weighted_unifrac_PCoA_cluster.pdf]

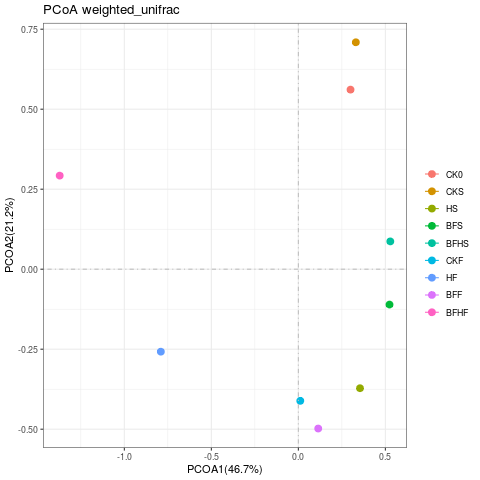

Supplement: Supplementary file 1 [file Data_Sheet_1.zip › 4.Beta_diversity/PCoA/weighted_unifrac_PCoA_cluster.png]

PCoA weighted\_unifrac

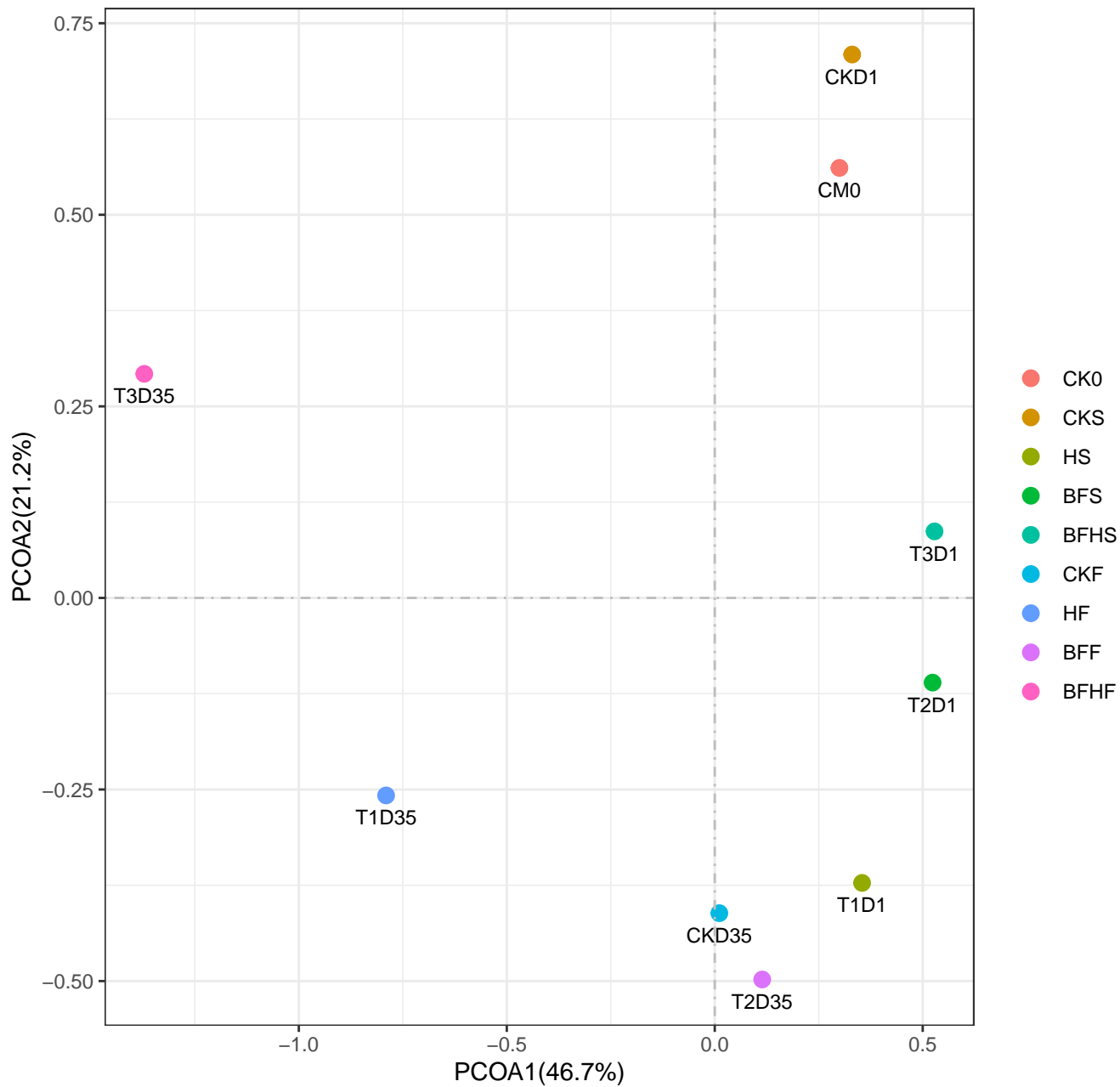

Supplement: Supplementary file 1 [file Data_Sheet_1.zip › 4.Beta_diversity/PCoA/weighted_unifrac_PCoA_name.pdf]

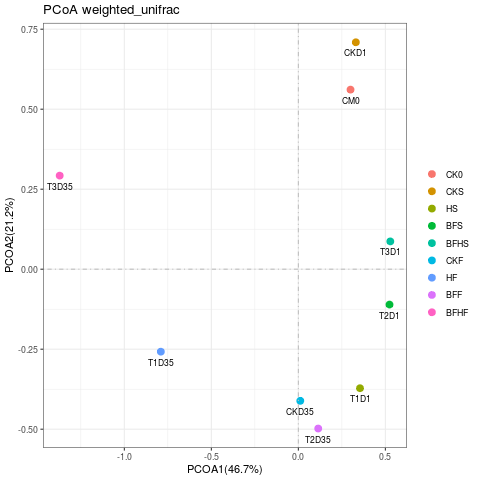

Supplement: Supplementary file 1 [file Data_Sheet_1.zip › 4.Beta_diversity/PCoA/weighted_unifrac_PCoA_name.png]

PCoA weighted\_unifrac

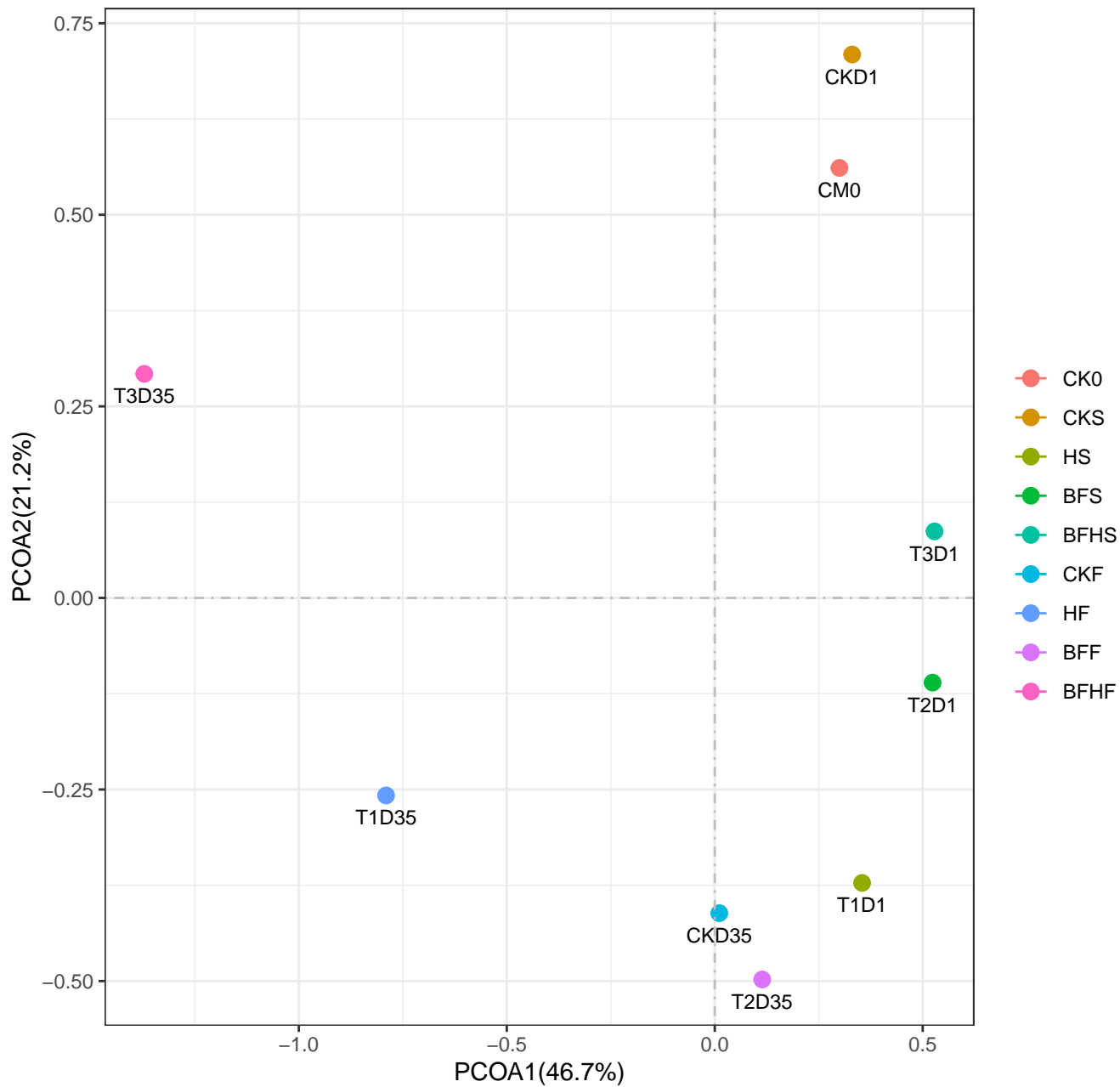

Supplement: Supplementary file 1 [file Data_Sheet_1.zip › 4.Beta_diversity/PCoA/weighted_unifrac_PCoA_name_cluster.pdf]

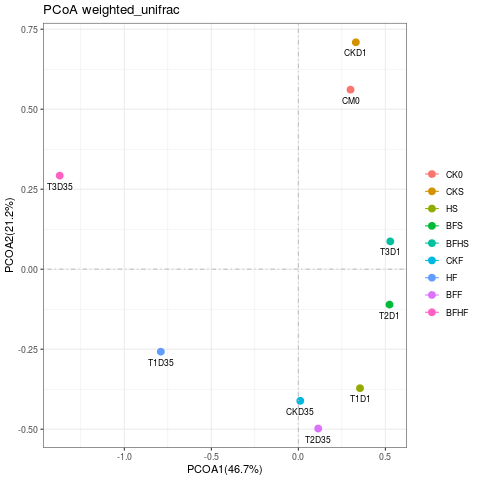

Supplement: Supplementary file 1 [file Data_Sheet_1.zip › 4.Beta_diversity/PCoA/weighted_unifrac_PCoA_name_cluster.png]

# UPGMA aitchison

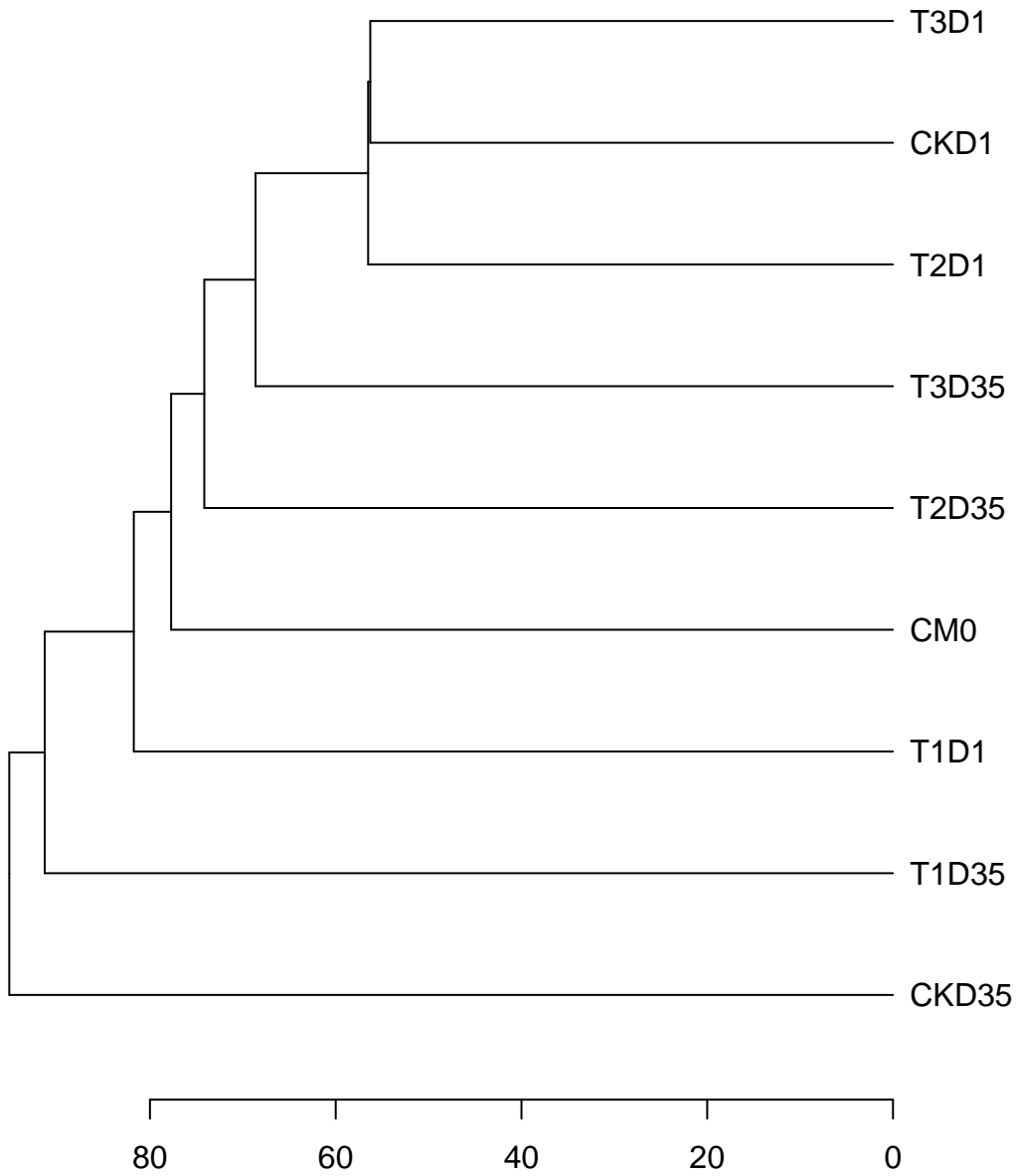

Supplement: Supplementary file 1 [file Data_Sheet_1.zip › 4.Beta_diversity/UPGMA/aitchison_UPGMA.pdf]

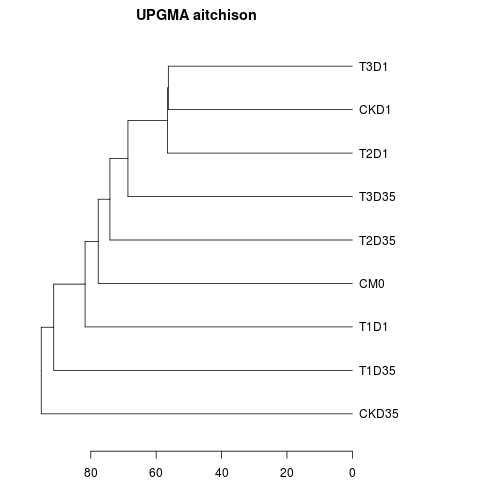

Supplement: Supplementary file 1 [file Data_Sheet_1.zip › 4.Beta_diversity/UPGMA/aitchison_UPGMA.png]

# UPGMA bray\_curtis

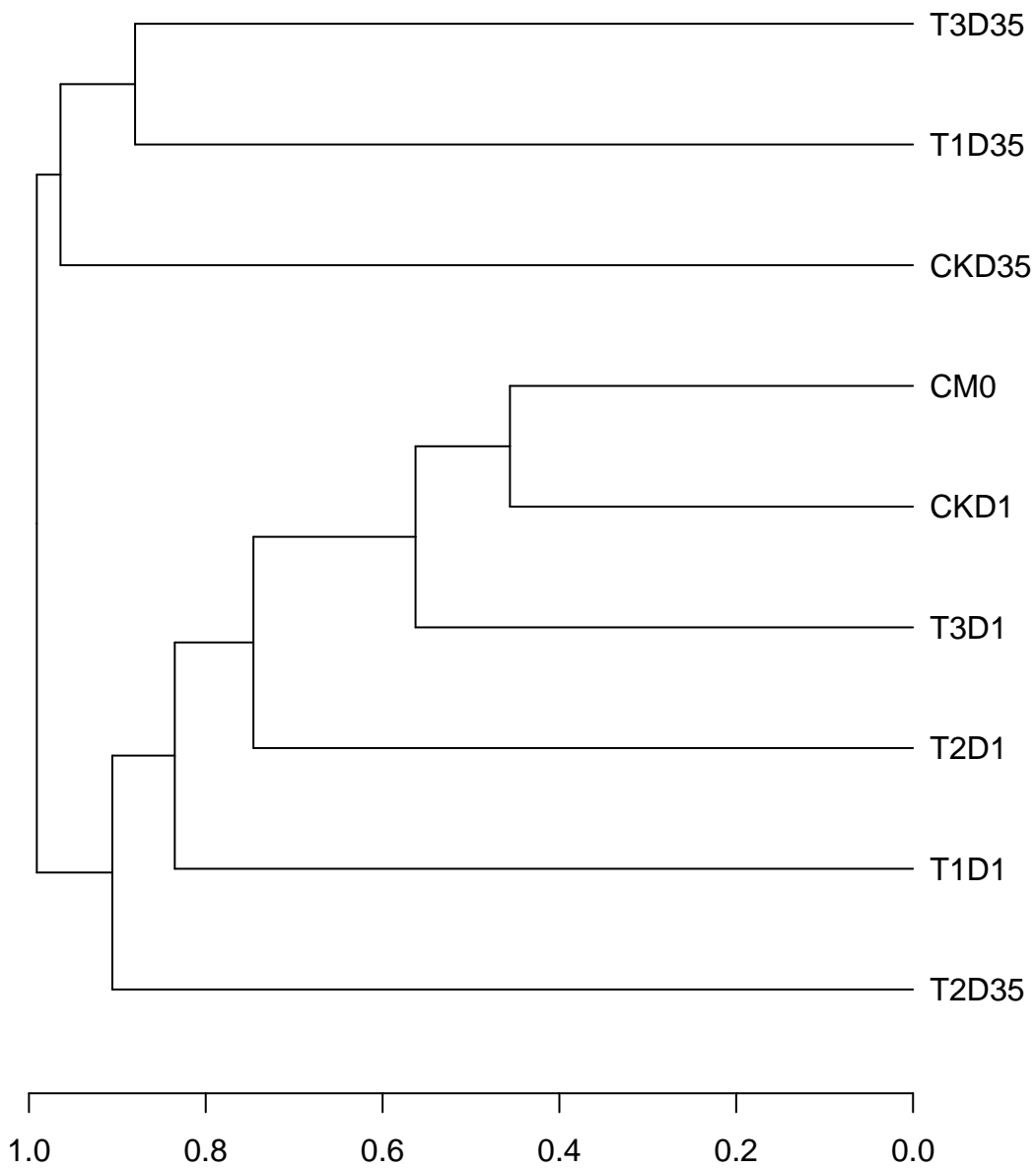

Supplement: Supplementary file 1 [file Data_Sheet_1.zip › 4.Beta_diversity/UPGMA/bray_curtis_UPGMA.pdf]

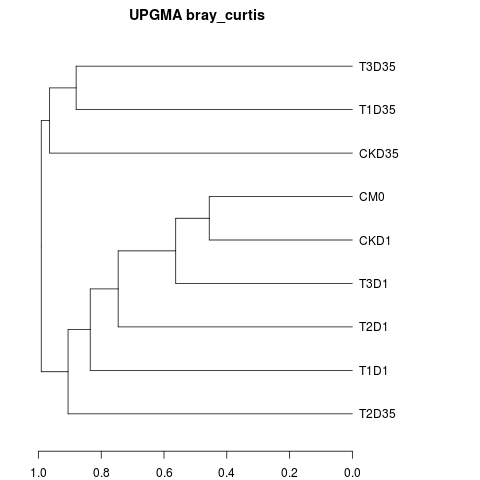

Supplement: Supplementary file 1 [file Data_Sheet_1.zip › 4.Beta_diversity/UPGMA/bray_curtis_UPGMA.png]

# UPGMA jaccard

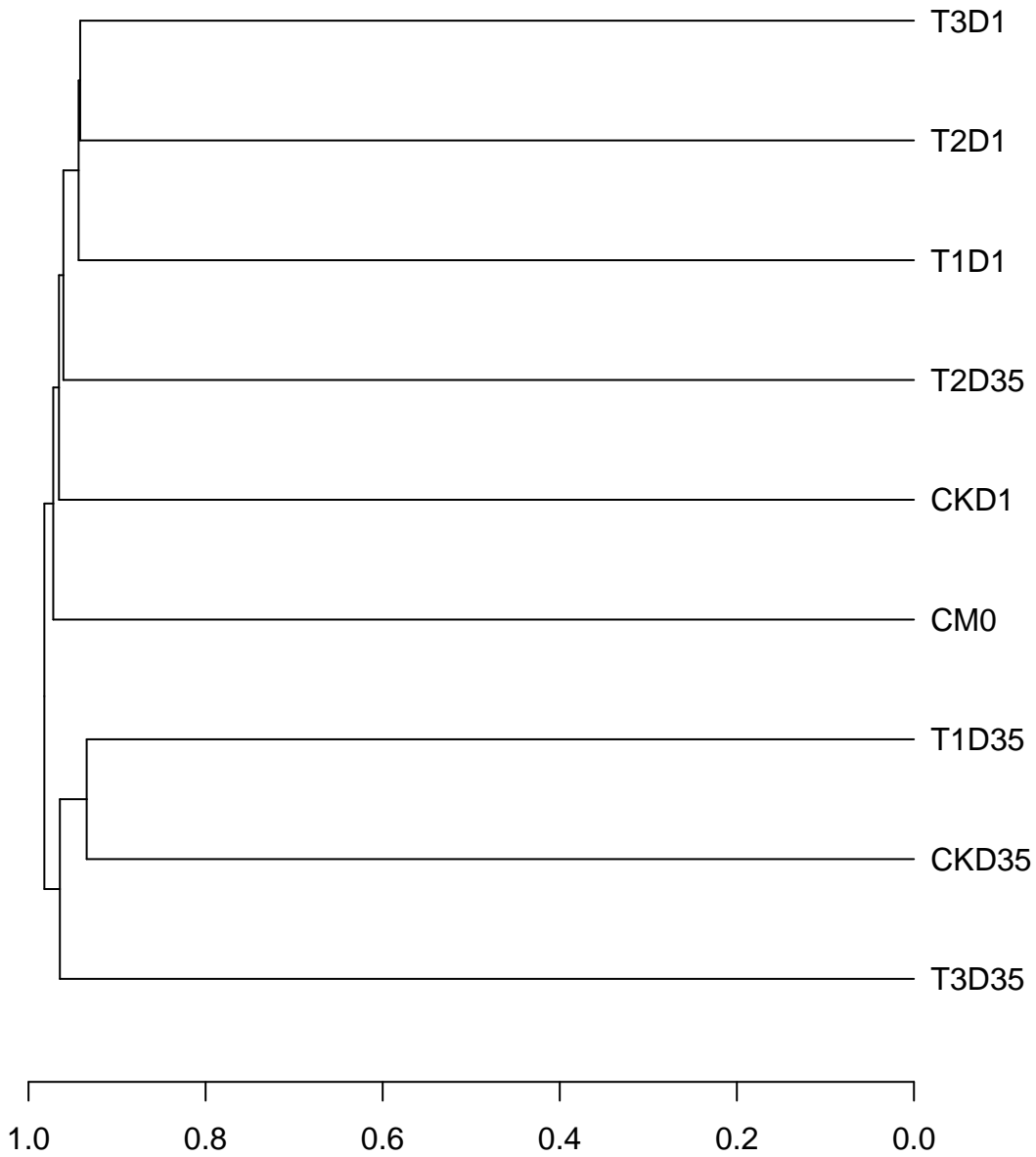

Supplement: Supplementary file 1 [file Data_Sheet_1.zip › 4.Beta_diversity/UPGMA/jaccard_UPGMA.pdf]

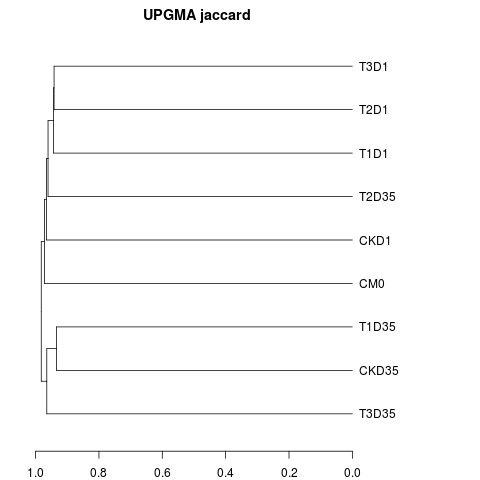

Supplement: Supplementary file 1 [file Data_Sheet_1.zip › 4.Beta_diversity/UPGMA/jaccard_UPGMA.png]

# UPGMA unweighted\_unifrac

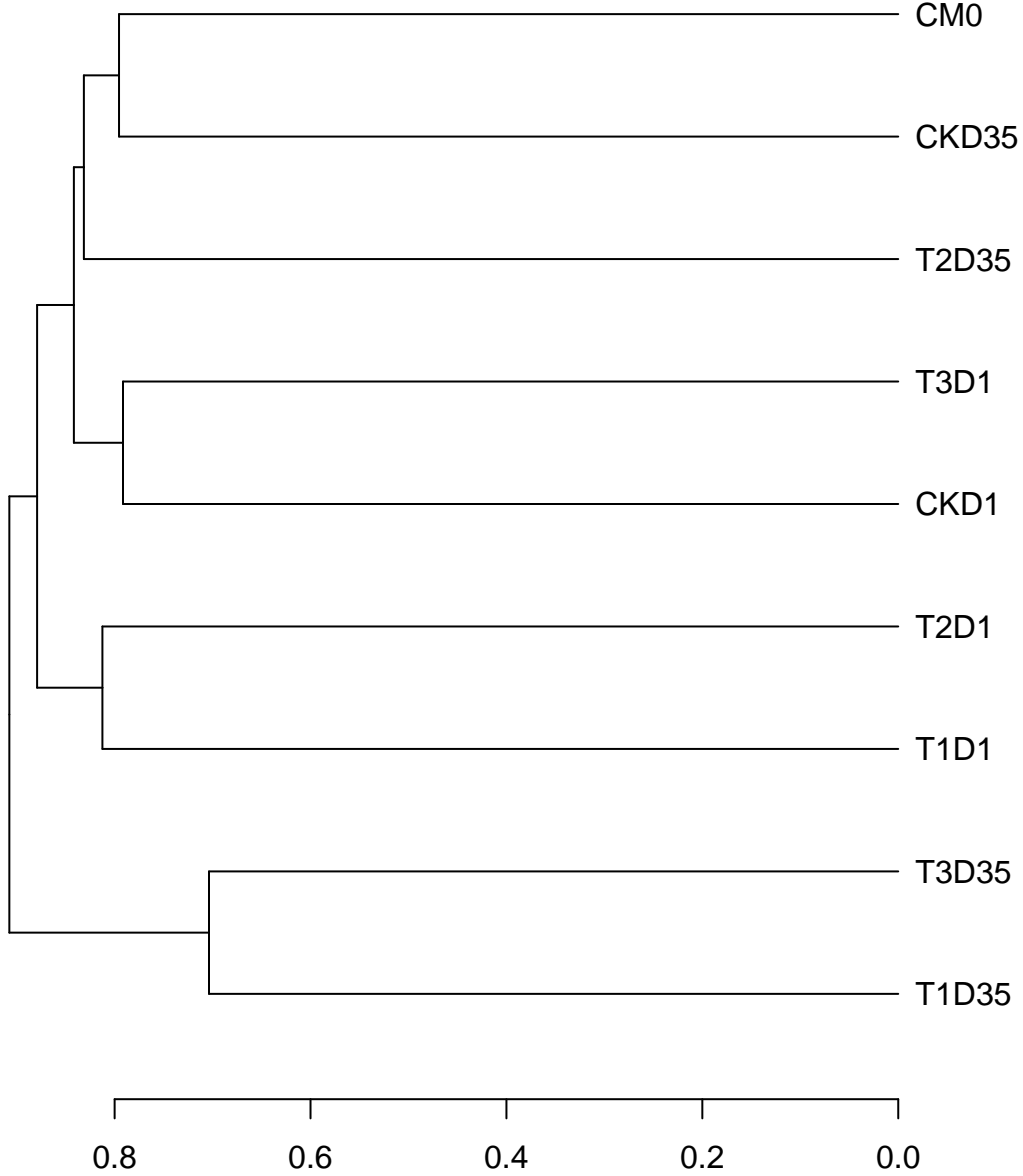

Supplement: Supplementary file 1 [file Data_Sheet_1.zip › 4.Beta_diversity/UPGMA/unweighted_unifrac_UPGMA.pdf]

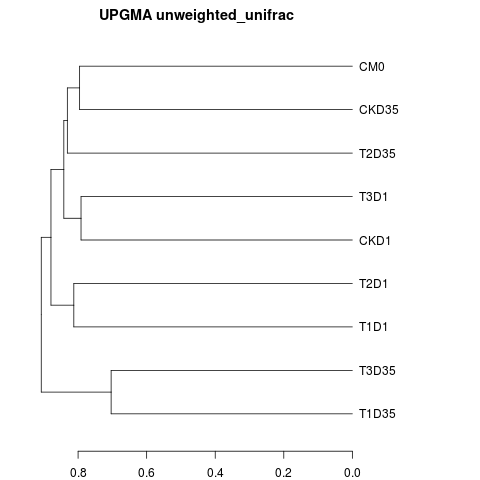

Supplement: Supplementary file 1 [file Data_Sheet_1.zip › 4.Beta_diversity/UPGMA/unweighted_unifrac_UPGMA.png]

# UPGMA weighted\_unifrac

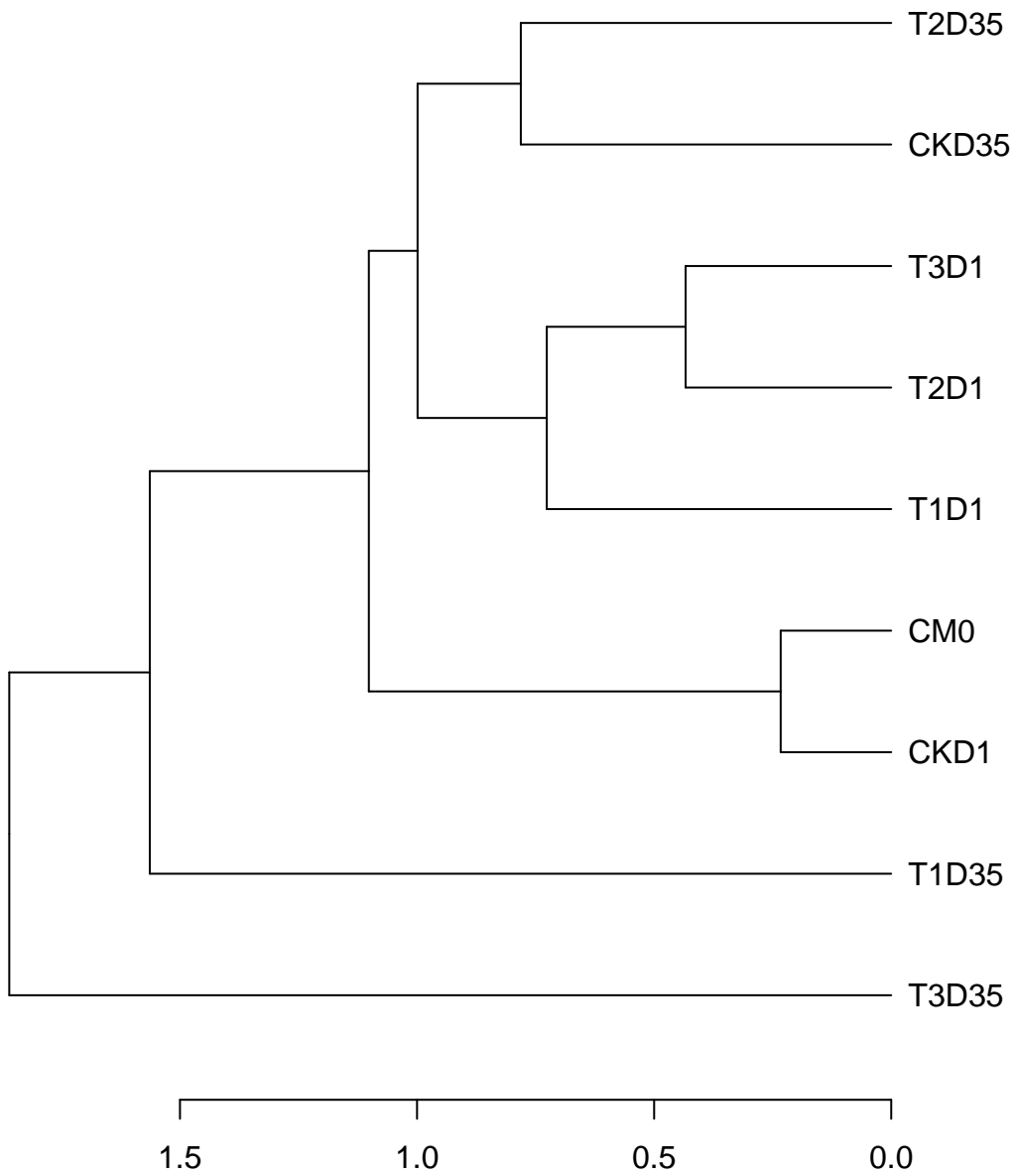

Supplement: Supplementary file 1 [file Data_Sheet_1.zip › 4.Beta_diversity/UPGMA/weighted_unifrac_UPGMA.pdf]

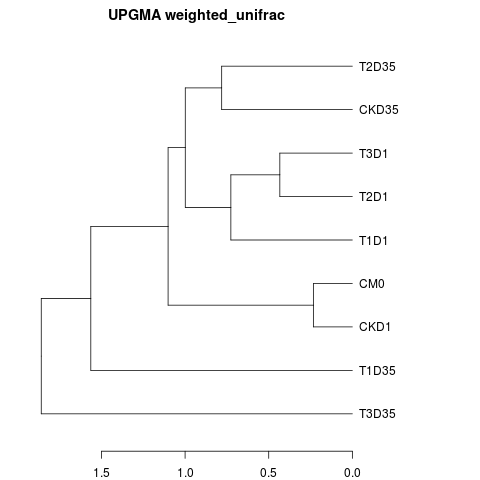

Supplement: Supplementary file 1 [file Data_Sheet_1.zip › 4.Beta_diversity/UPGMA/weighted_unifrac_UPGMA.png]

FunGuild\_11

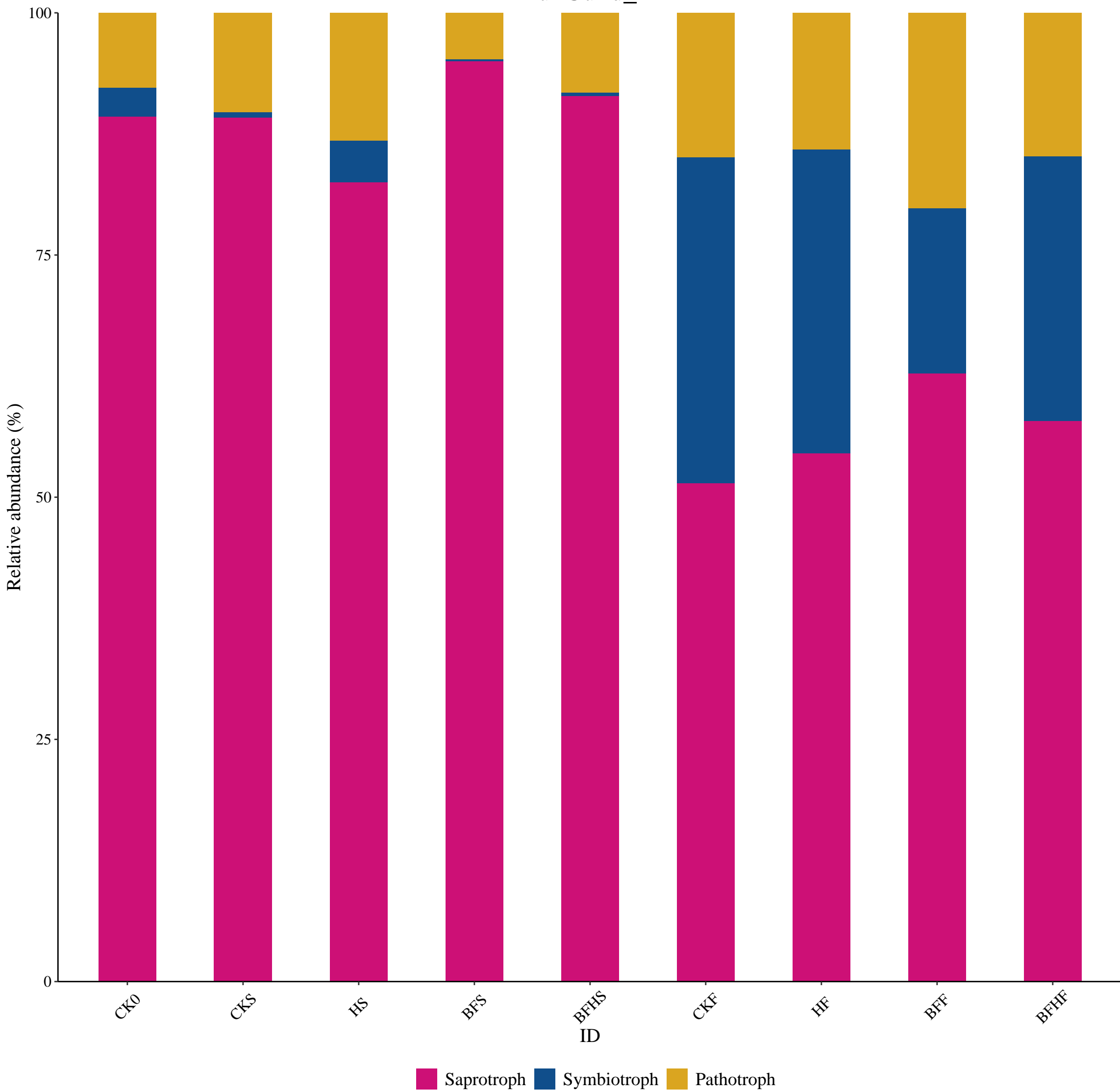

Supplement: Supplementary file 1 [file Data_Sheet_1.zip › 6.Function/All/FunGuild/l1/barplot_group.pdf]

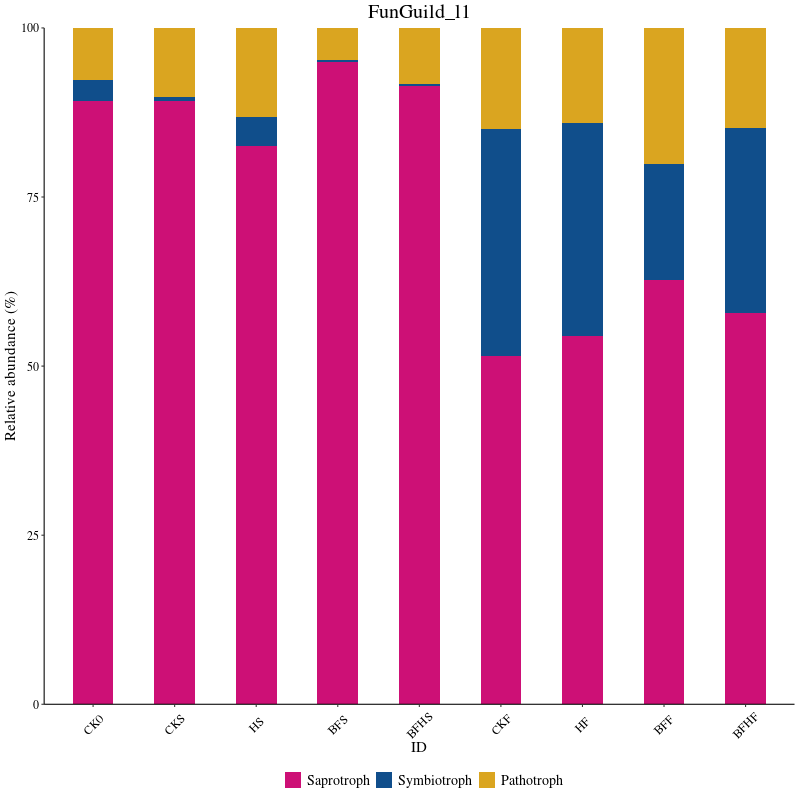

Supplement: Supplementary file 1 [file Data_Sheet_1.zip › 6.Function/All/FunGuild/l1/barplot_group.png]

FunGuild\_11

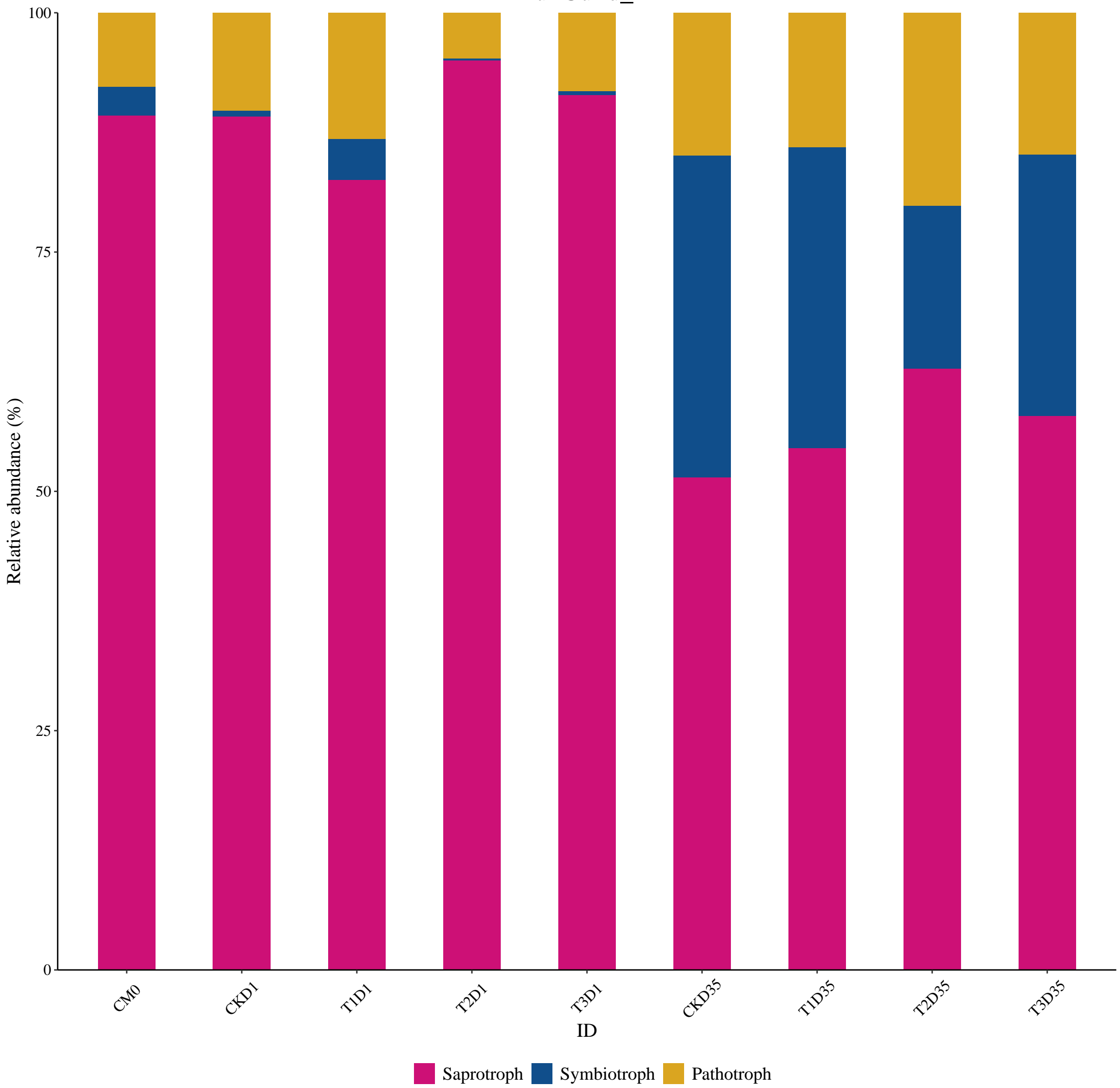

Supplement: Supplementary file 1 [file Data_Sheet_1.zip › 6.Function/All/FunGuild/l1/barplot_sample.pdf]

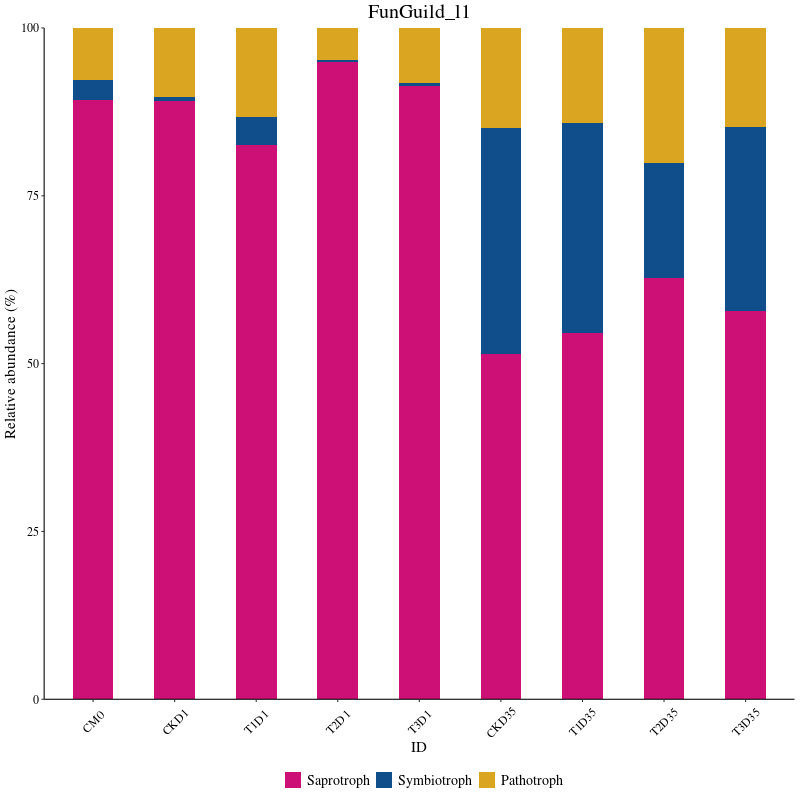

Supplement: Supplementary file 1 [file Data_Sheet_1.zip › 6.Function/All/FunGuild/l1/barplot_sample.png]

# FunGuild\_I1

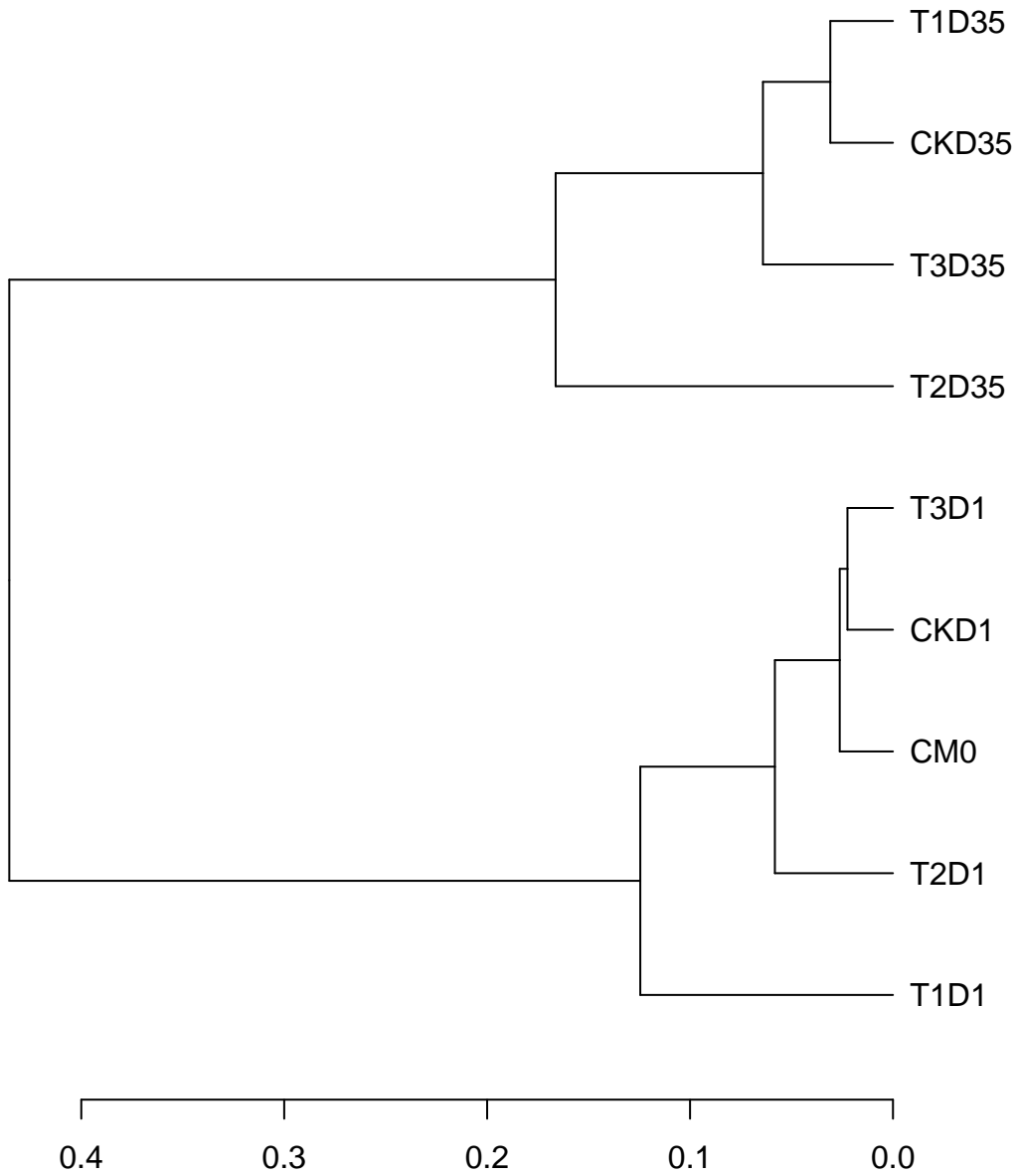

Supplement: Supplementary file 1 [file Data_Sheet_1.zip › 6.Function/All/FunGuild/l1/cluster.pdf]

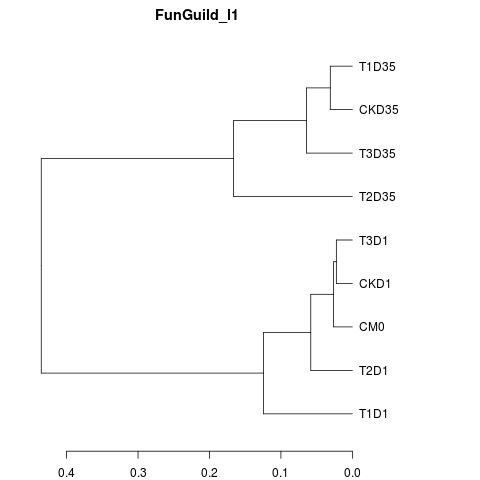

Supplement: Supplementary file 1 [file Data_Sheet_1.zip › 6.Function/All/FunGuild/l1/cluster.png]

FunGuild\_11

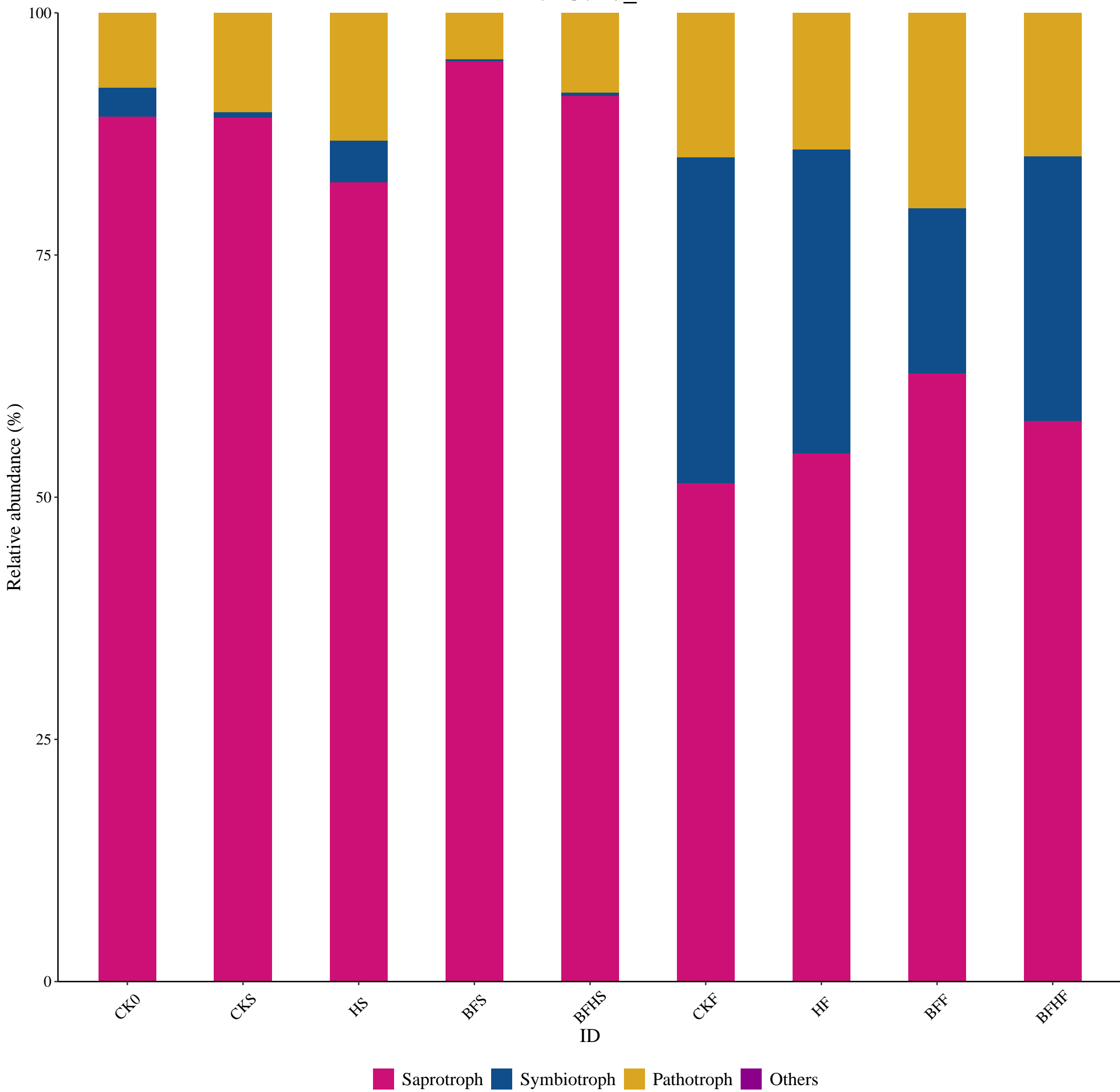

Supplement: Supplementary file 1 [file Data_Sheet_1.zip › 6.Function/All/FunGuild/l1/Freq.0.01.barplot_group.pdf]

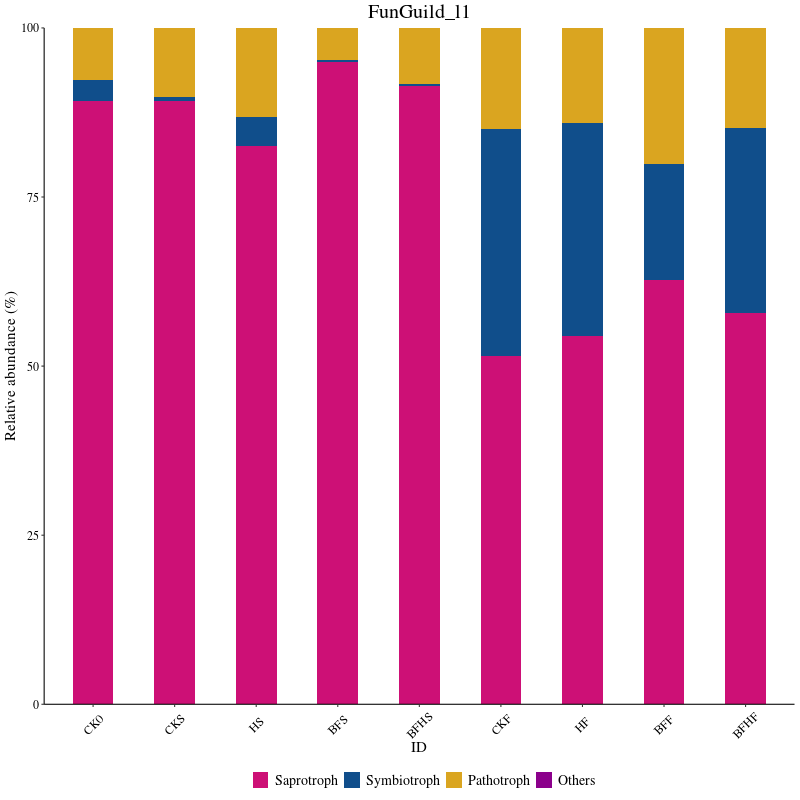

Supplement: Supplementary file 1 [file Data_Sheet_1.zip › 6.Function/All/FunGuild/l1/Freq.0.01.barplot_group.png]

FunGuild\_11

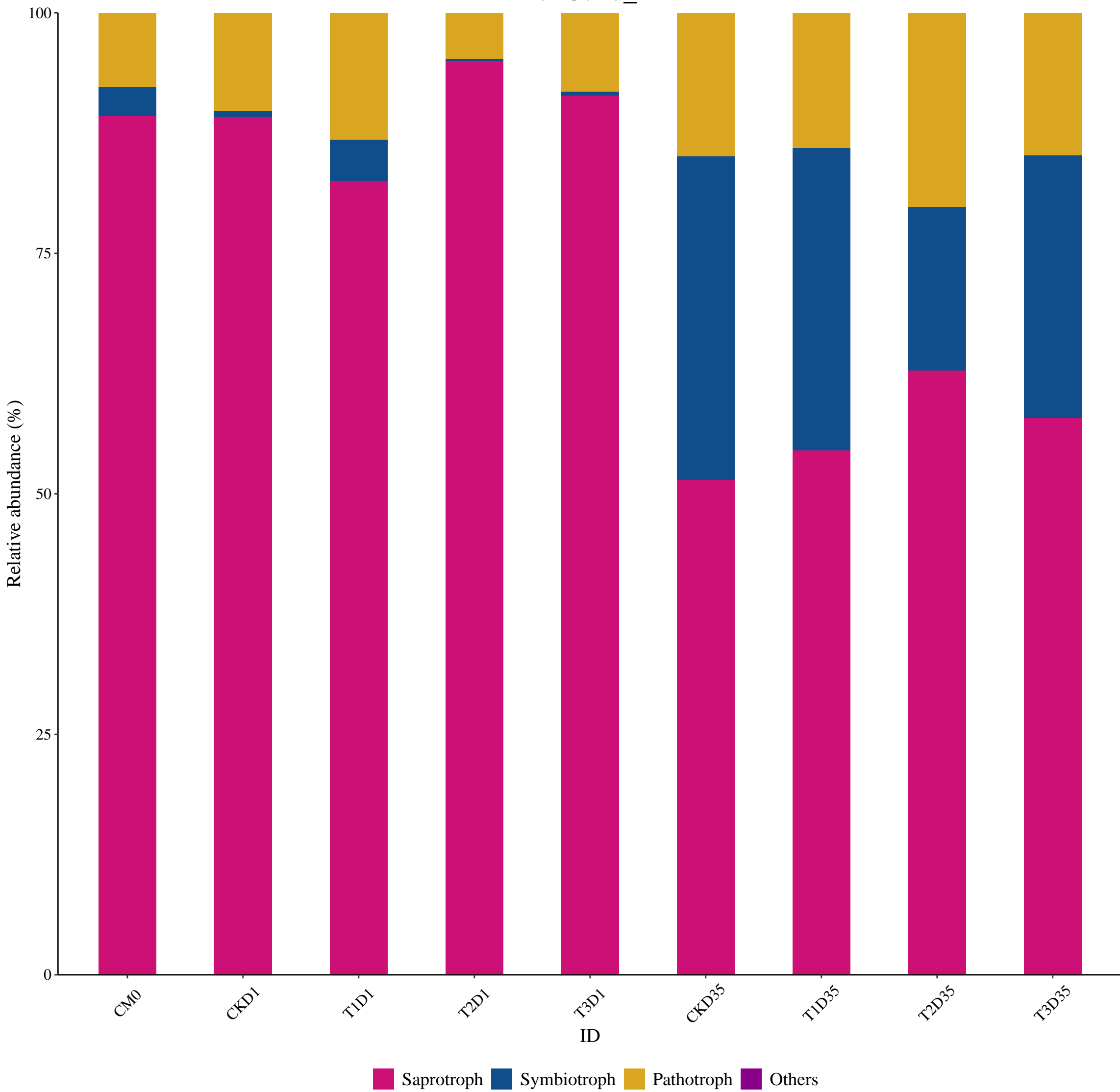

Supplement: Supplementary file 1 [file Data_Sheet_1.zip › 6.Function/All/FunGuild/l1/Freq.0.01.barplot_sample.pdf]

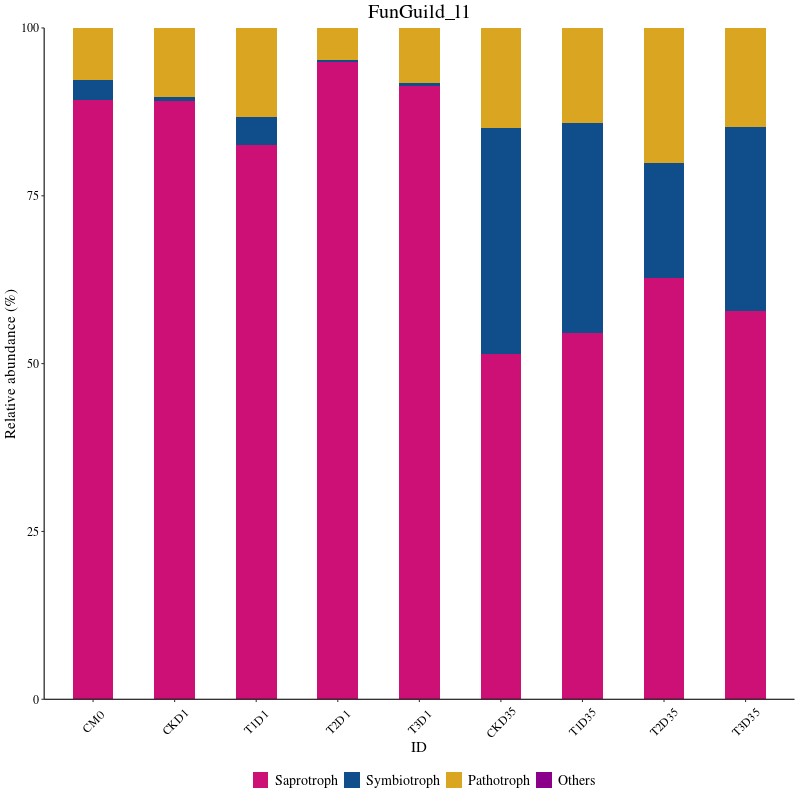

Supplement: Supplementary file 1 [file Data_Sheet_1.zip › 6.Function/All/FunGuild/l1/Freq.0.01.barplot_sample.png]
