# Supplementary figures and images for: Effects of Trichoderma harzianum combined with Phanerochaete chrysosporium on lignin degradation and humification during chicken manure and rice husk composting (part 1 of 2)
Source: Front Microbiol. 2025 Feb 28;16:1515931. doi: 10.3389/fmicb.2025.1515931 (PMC11906335; doi:10.3389/fmicb.2025.1515931)

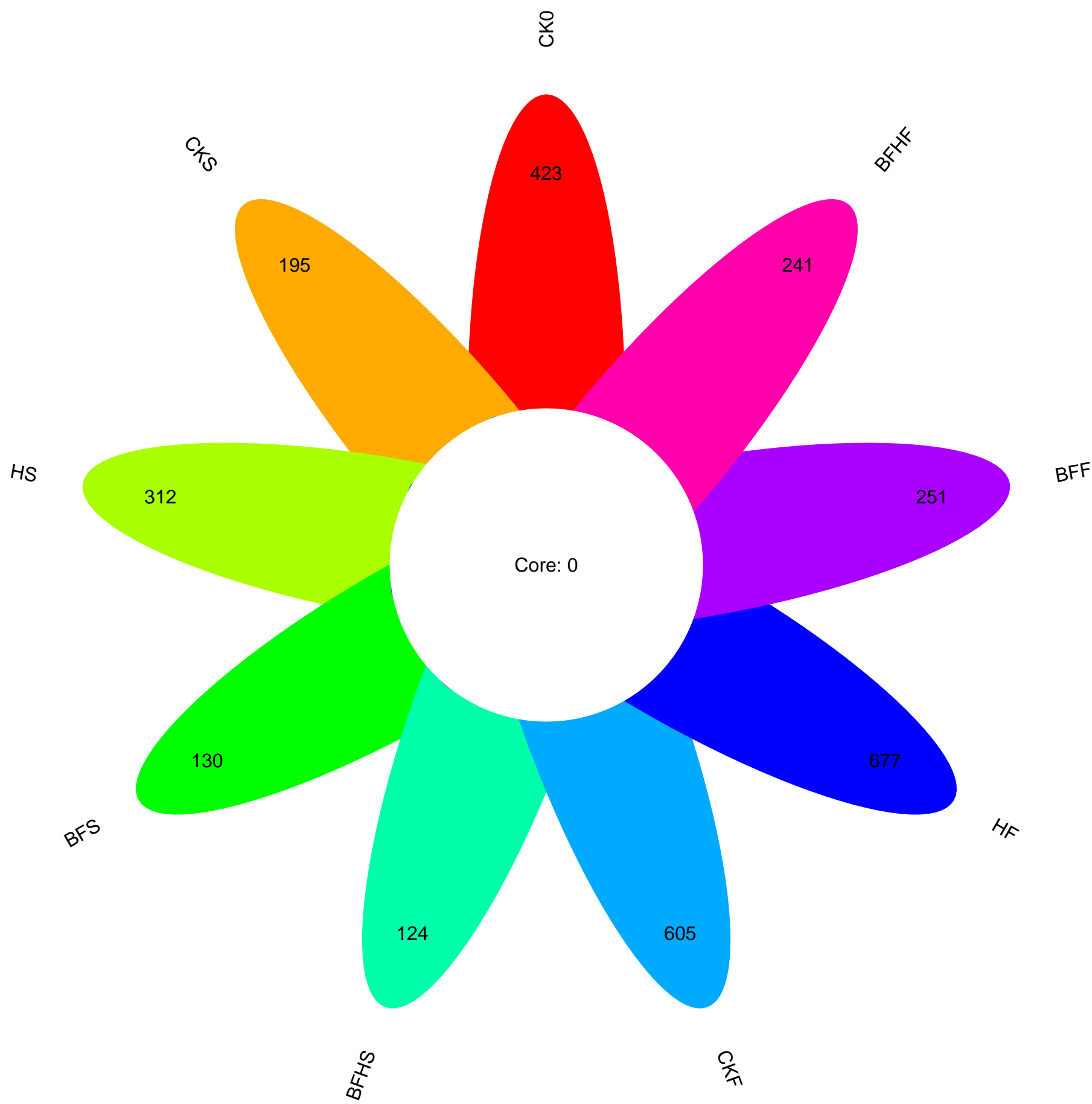

Supplement: Supplementary file 1 [file Data_Sheet_1.zip › 2.Feature_sequence/flower.pdf]

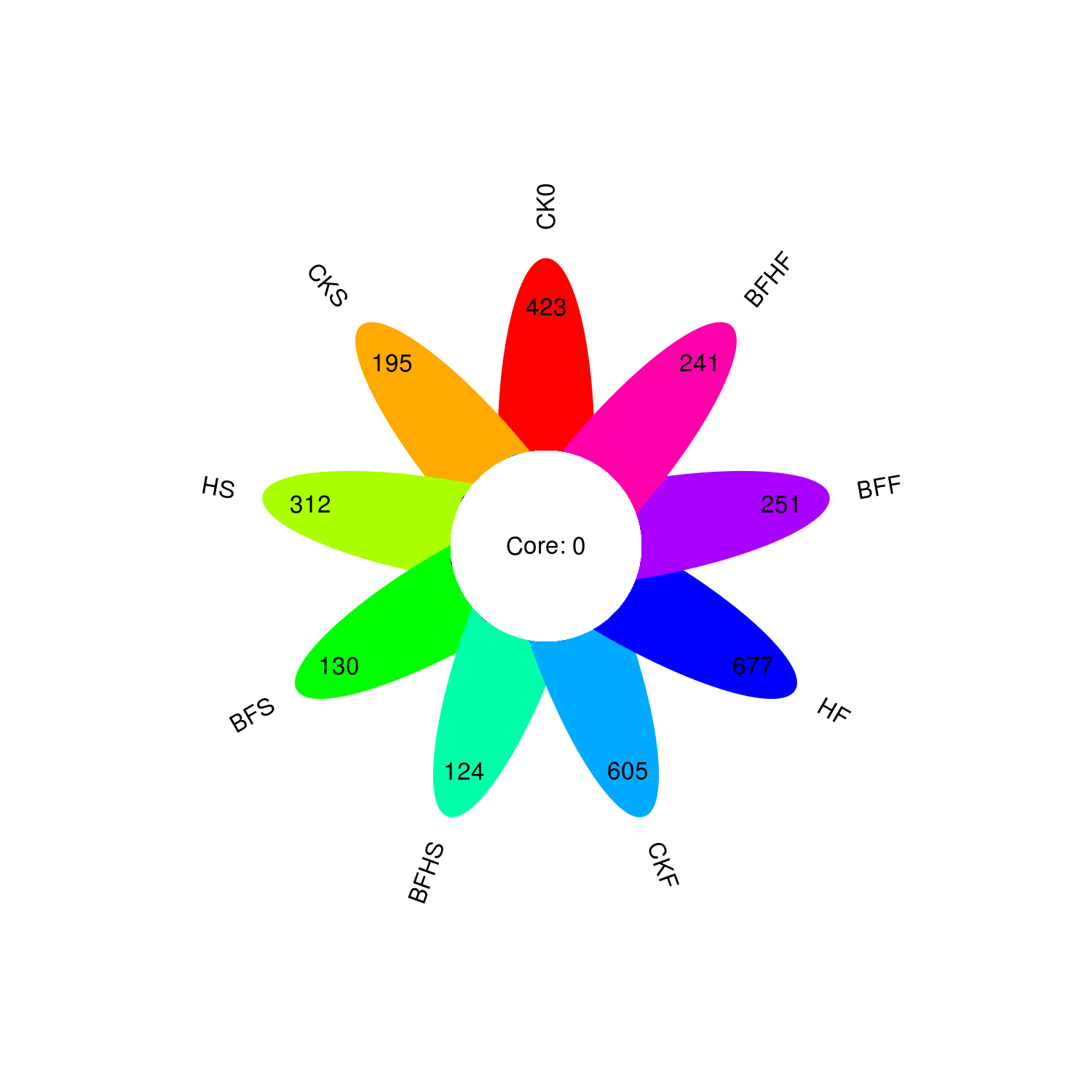

Supplement: Supplementary file 1 [file Data_Sheet_1.zip › 2.Feature_sequence/flower.png]

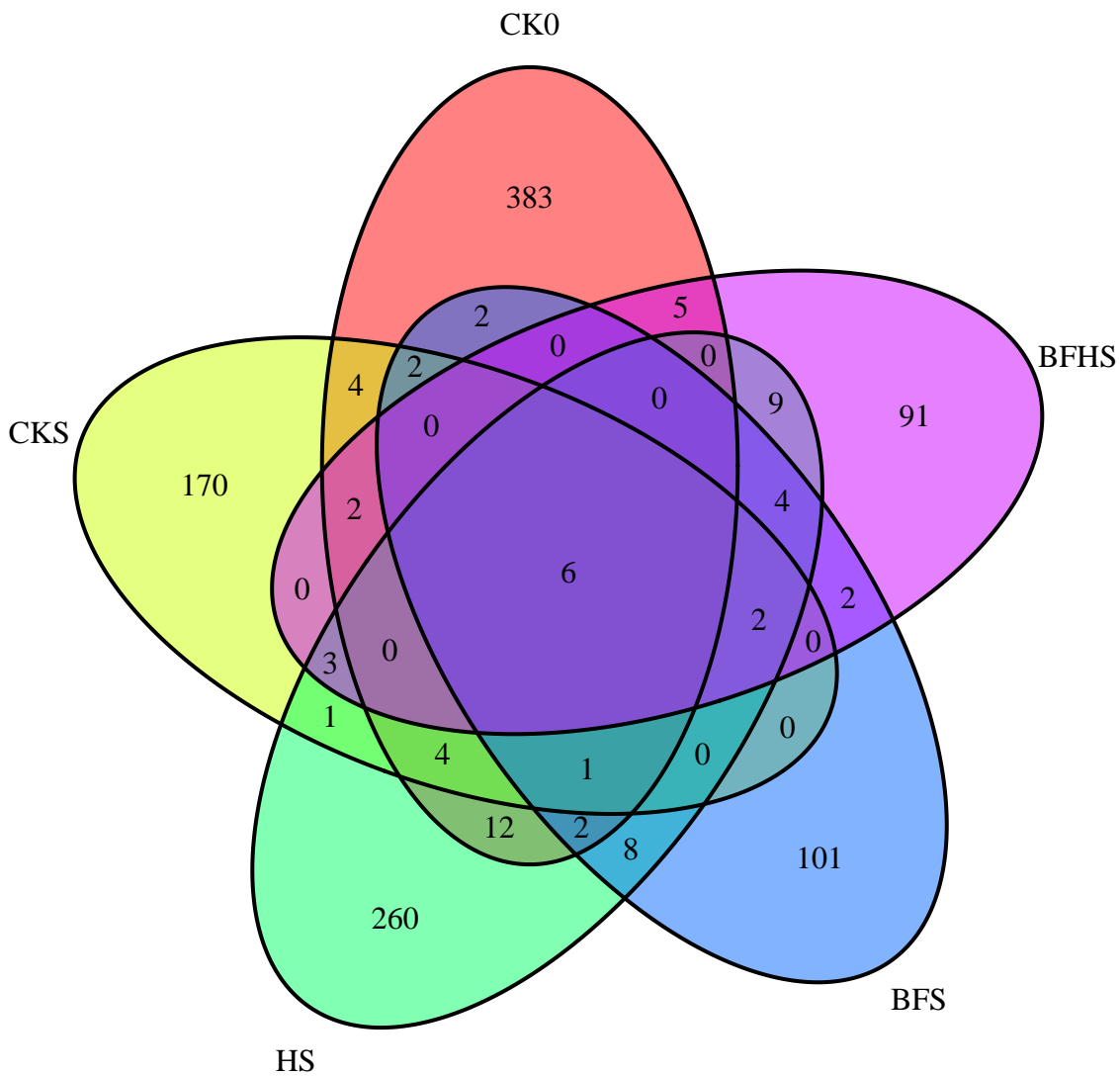

Supplement: Supplementary file 1 [file Data_Sheet_1.zip › 2.Feature_sequence/venn.pdf]

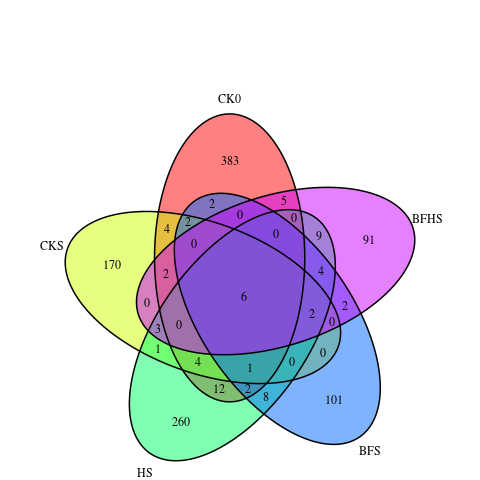

Supplement: Supplementary file 1 [file Data_Sheet_1.zip › 2.Feature_sequence/venn.png]

Kruskal-Wallis,  $p = 0.4335$

group

|     |     |      |     |      |
|-----|-----|------|-----|------|
| CK0 | HS  | BFHS | HF  | BFHF |
| CKS | BFS | CKF  | BFF |      |

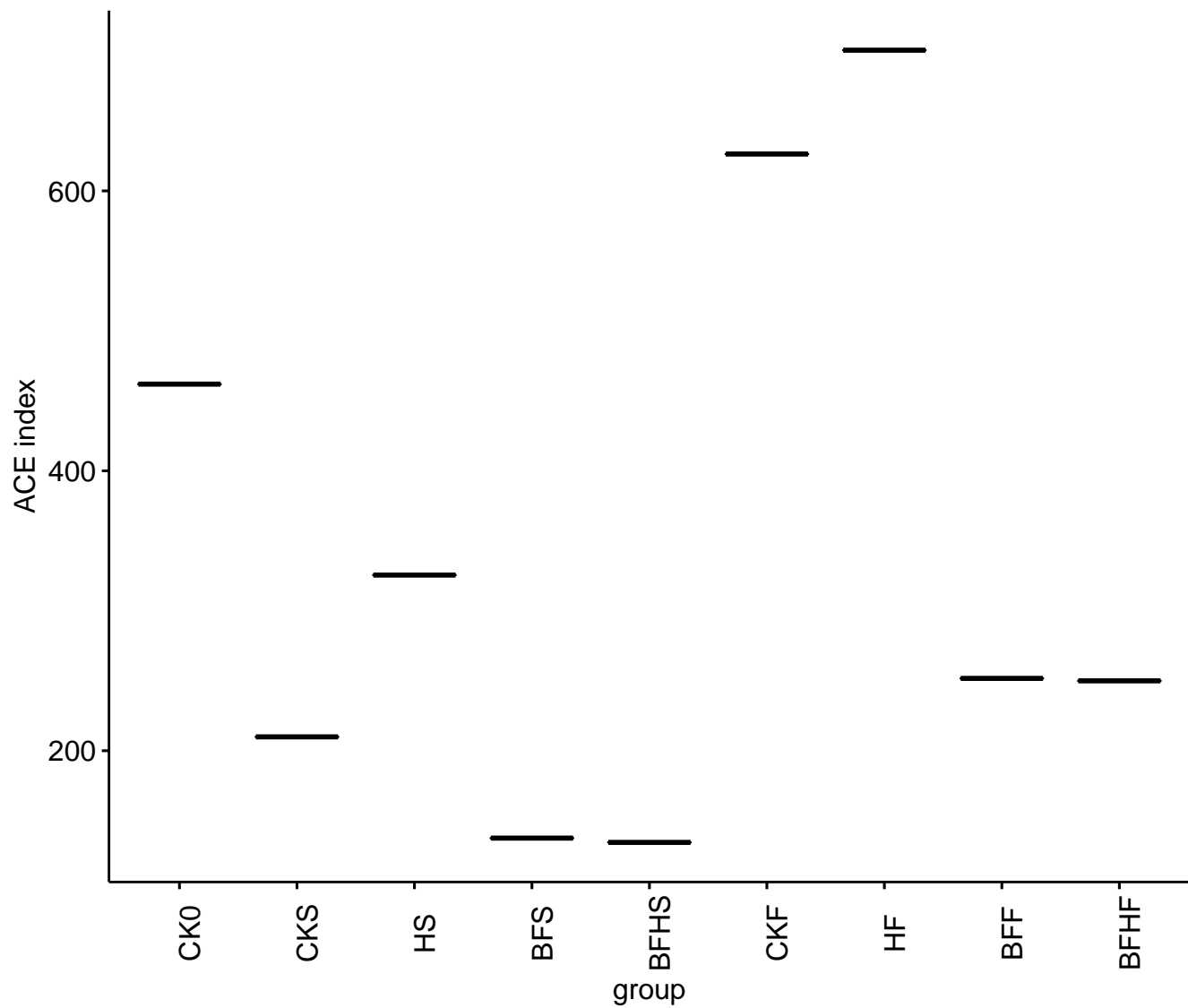

Supplement: Supplementary file 1 [file Data_Sheet_1.zip › 3.Alpha_diversity/ACE.boxplot.pdf]

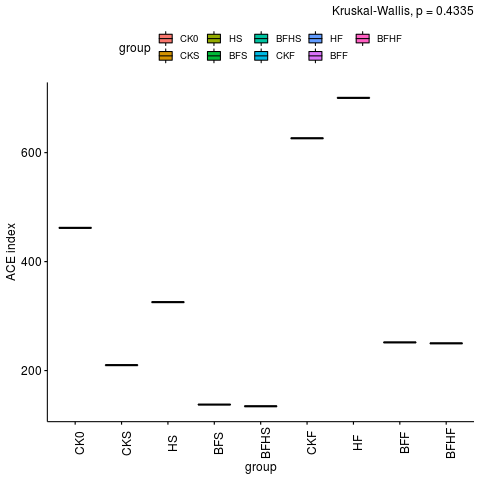

Supplement: Supplementary file 1 [file Data_Sheet_1.zip › 3.Alpha_diversity/ACE.boxplot.png]

Kruskal-Wallis,  $p = 0.4335$

group

|     |     |      |     |      |
|-----|-----|------|-----|------|
| CK0 | HS  | BFHS | HF  | BFHF |
| CKS | BFS | CKF  | BFF |      |

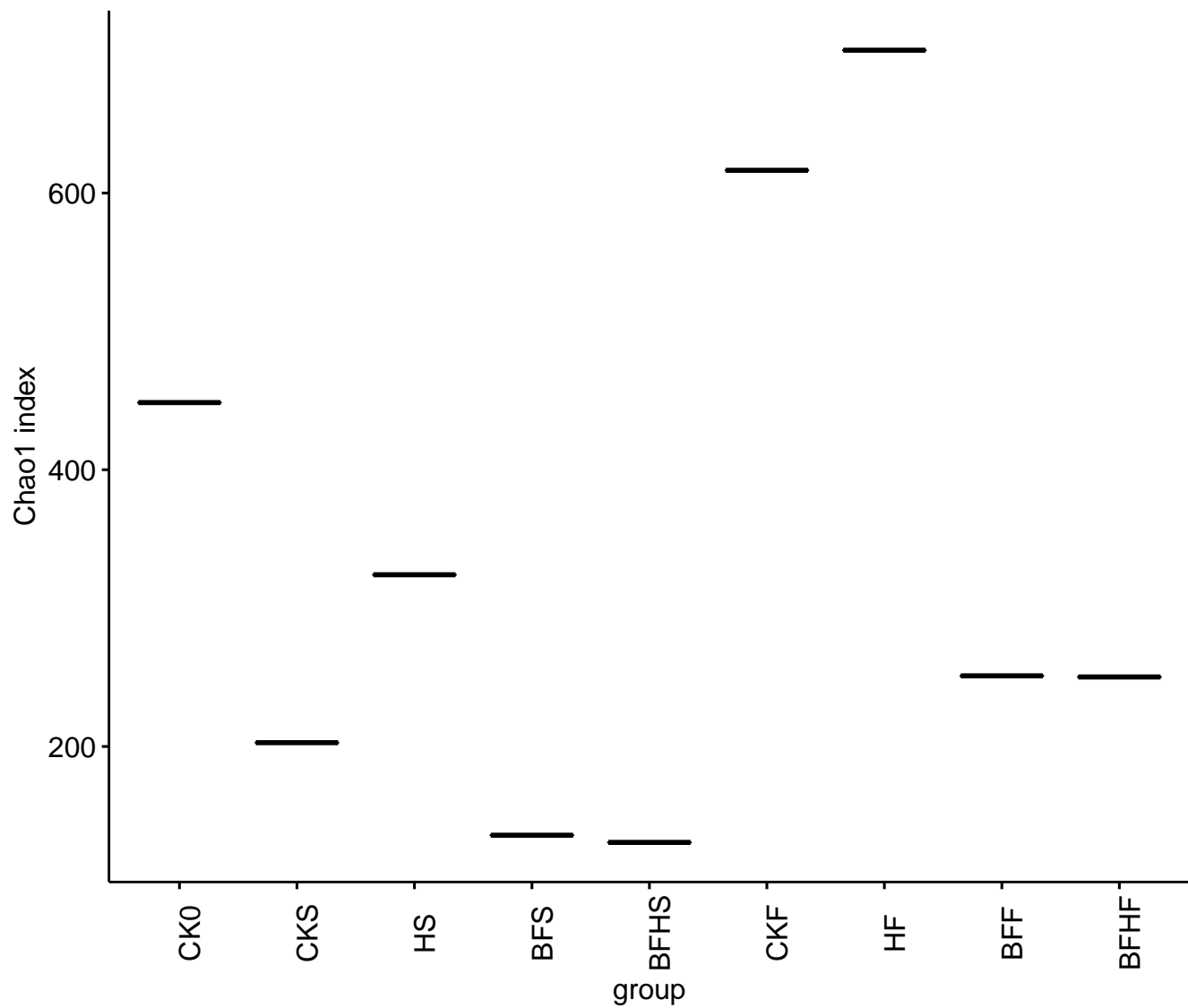

Supplement: Supplementary file 1 [file Data_Sheet_1.zip › 3.Alpha_diversity/Chao1.boxplot.pdf]

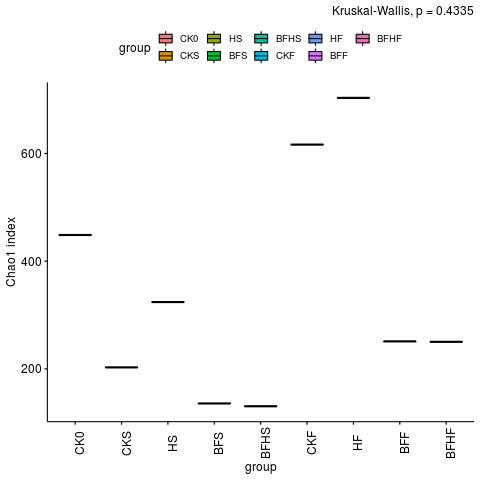

Supplement: Supplementary file 1 [file Data_Sheet_1.zip › 3.Alpha_diversity/Chao1.boxplot.png]

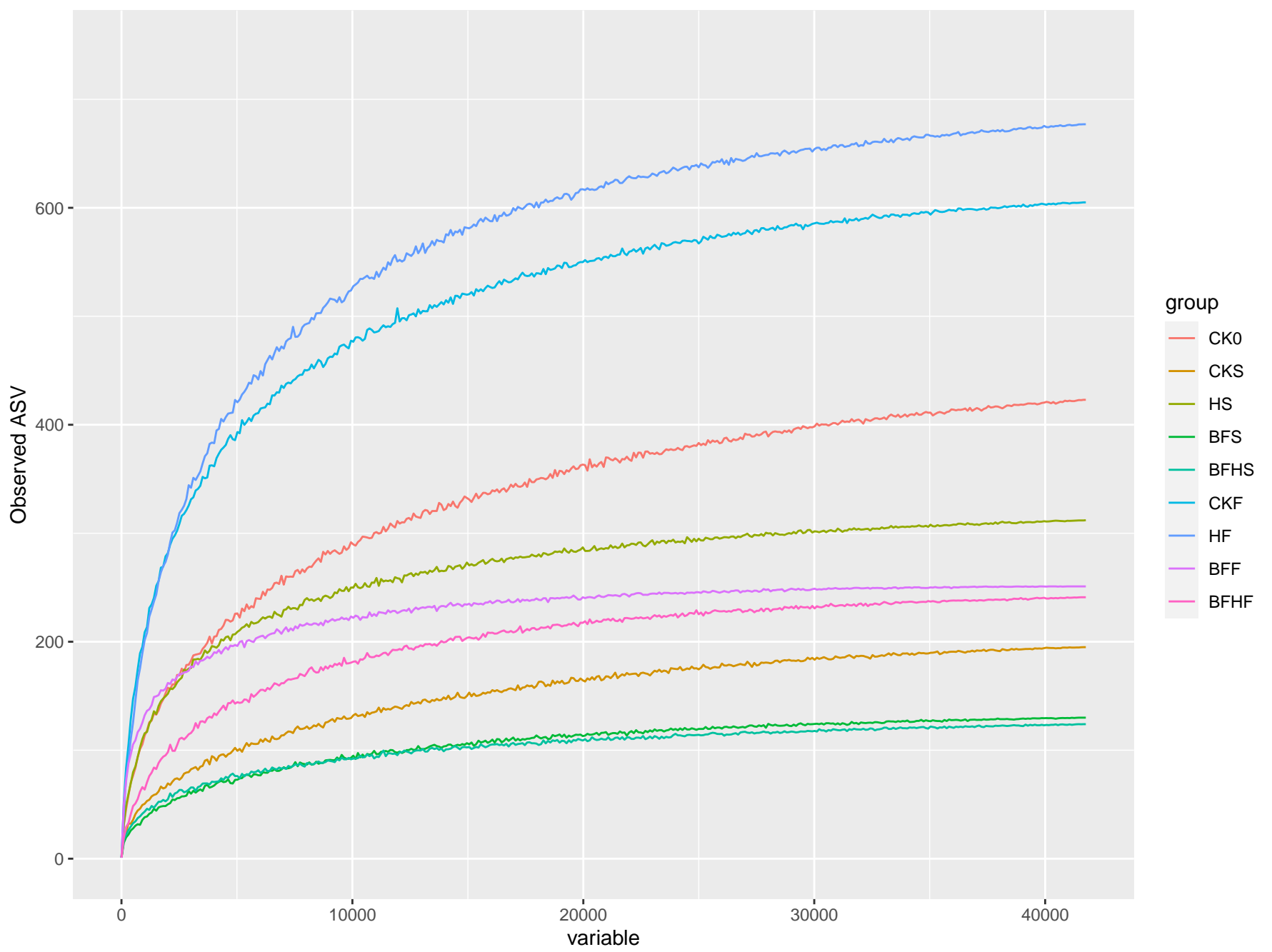

Supplement: Supplementary file 1 [file Data_Sheet_1.zip › 3.Alpha_diversity/observed_otus_group.pdf]

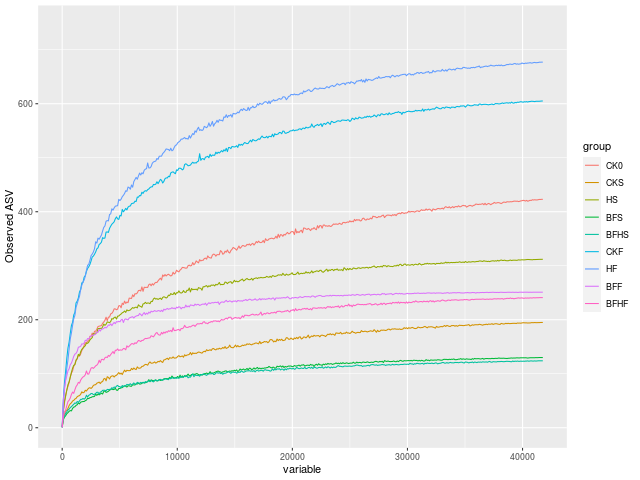

Supplement: Supplementary file 1 [file Data_Sheet_1.zip › 3.Alpha_diversity/observed_otus_group.png]

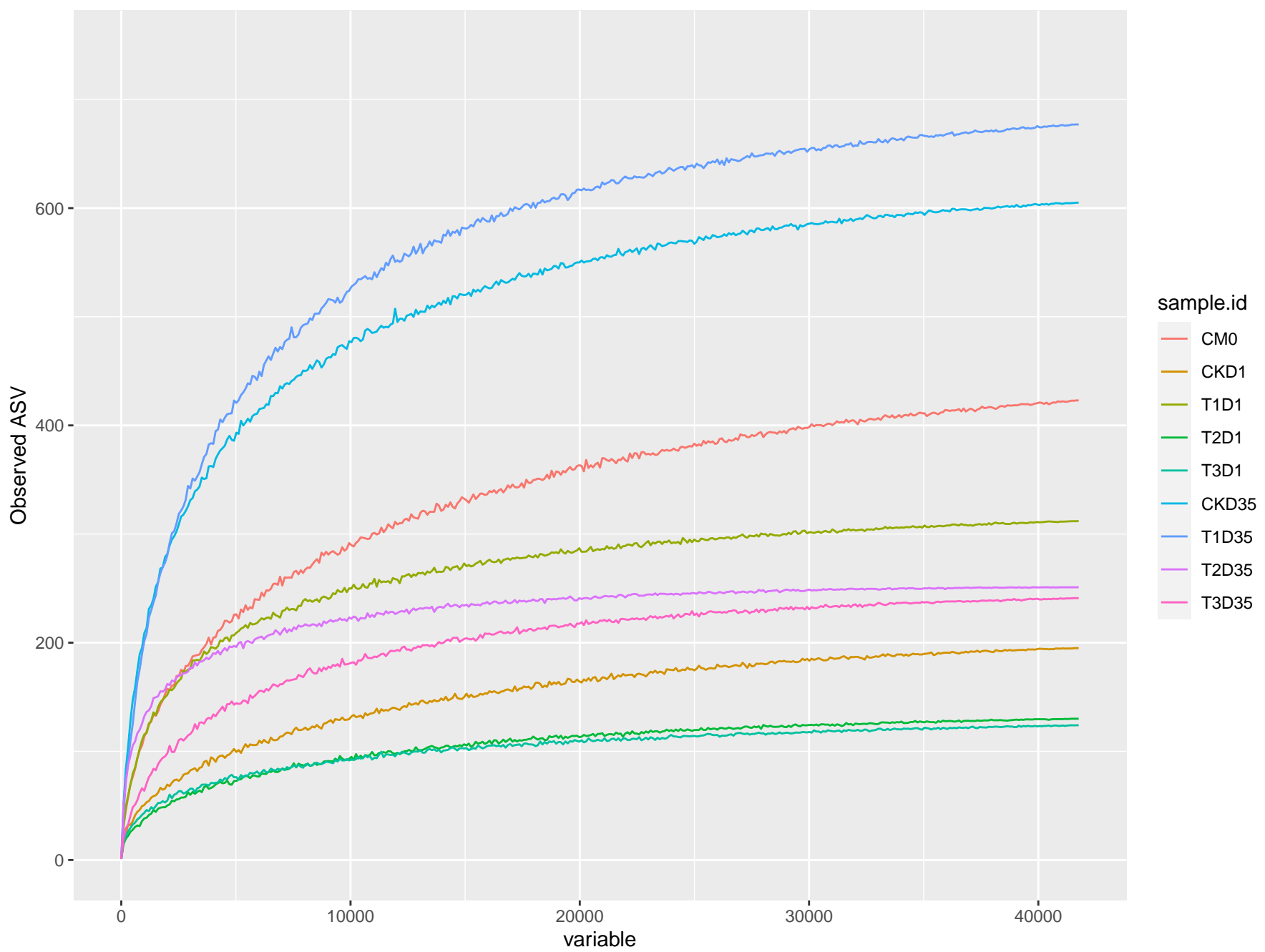

Supplement: Supplementary file 1 [file Data_Sheet_1.zip › 3.Alpha_diversity/observed_otus_sample.pdf]

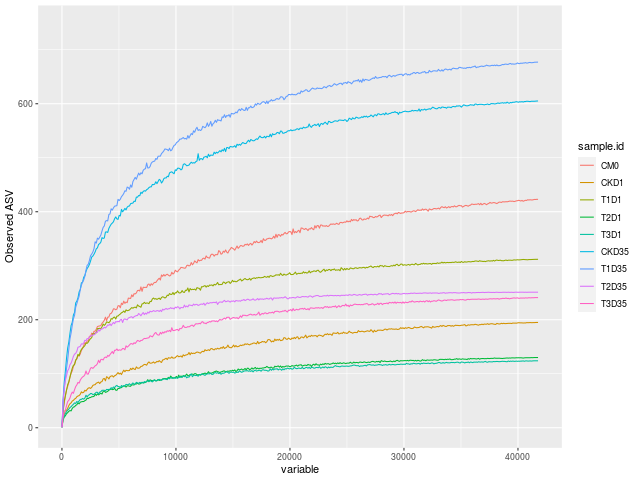

Supplement: Supplementary file 1 [file Data_Sheet_1.zip › 3.Alpha_diversity/observed_otus_sample.png]

Kruskal-Wallis,  $p = 0.4335$

group

|     |     |      |     |      |
|-----|-----|------|-----|------|
| CK0 | HS  | BFHS | HF  | BFHF |
| CKS | BFS | CKF  | BFF |      |

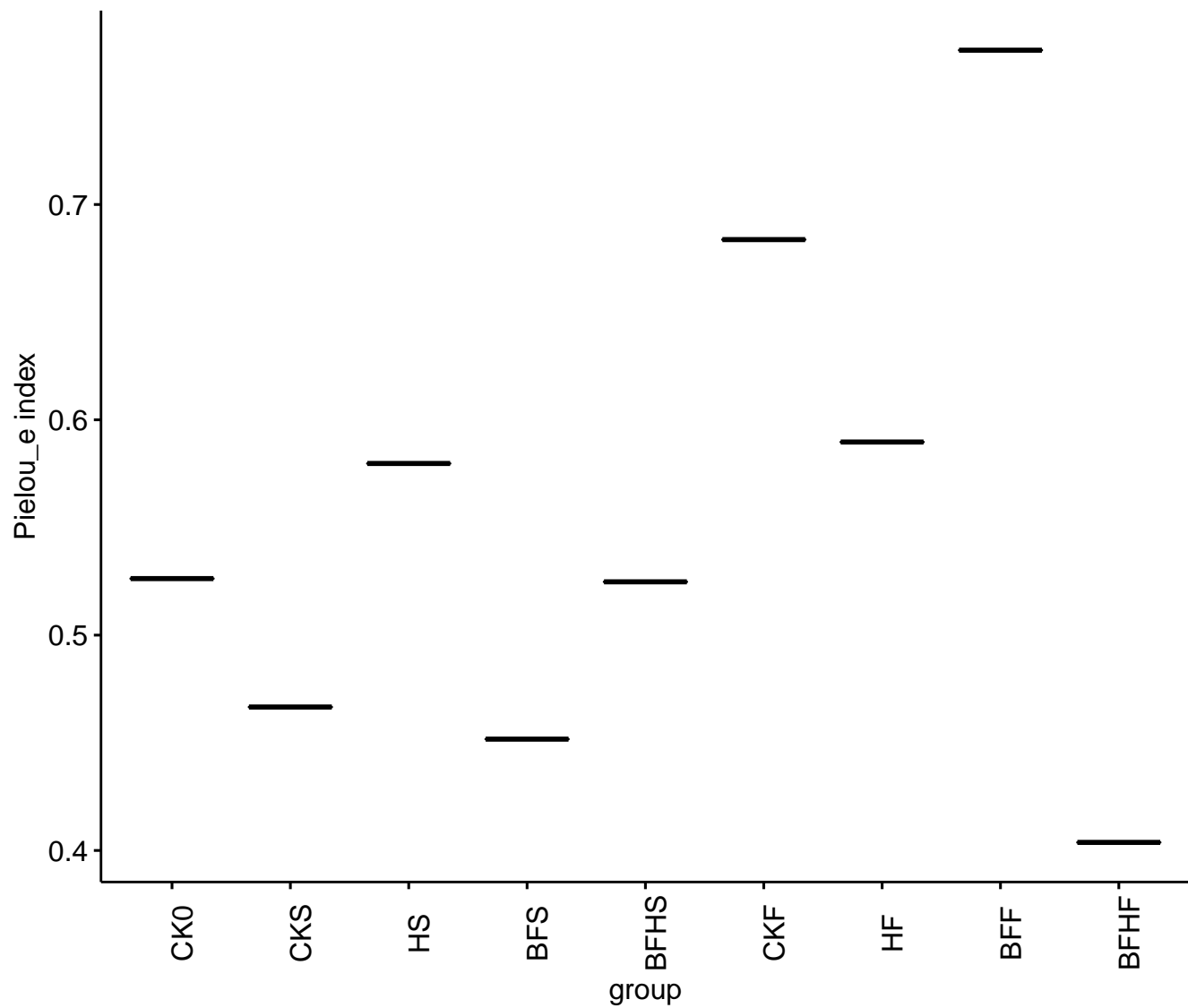

Supplement: Supplementary file 1 [file Data_Sheet_1.zip › 3.Alpha_diversity/Pielou_e.boxplot.pdf]

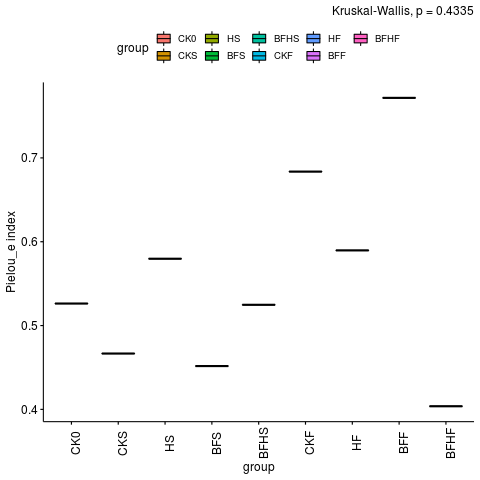

Supplement: Supplementary file 1 [file Data_Sheet_1.zip › 3.Alpha_diversity/Pielou_e.boxplot.png]

## Rank–Abundance Curves

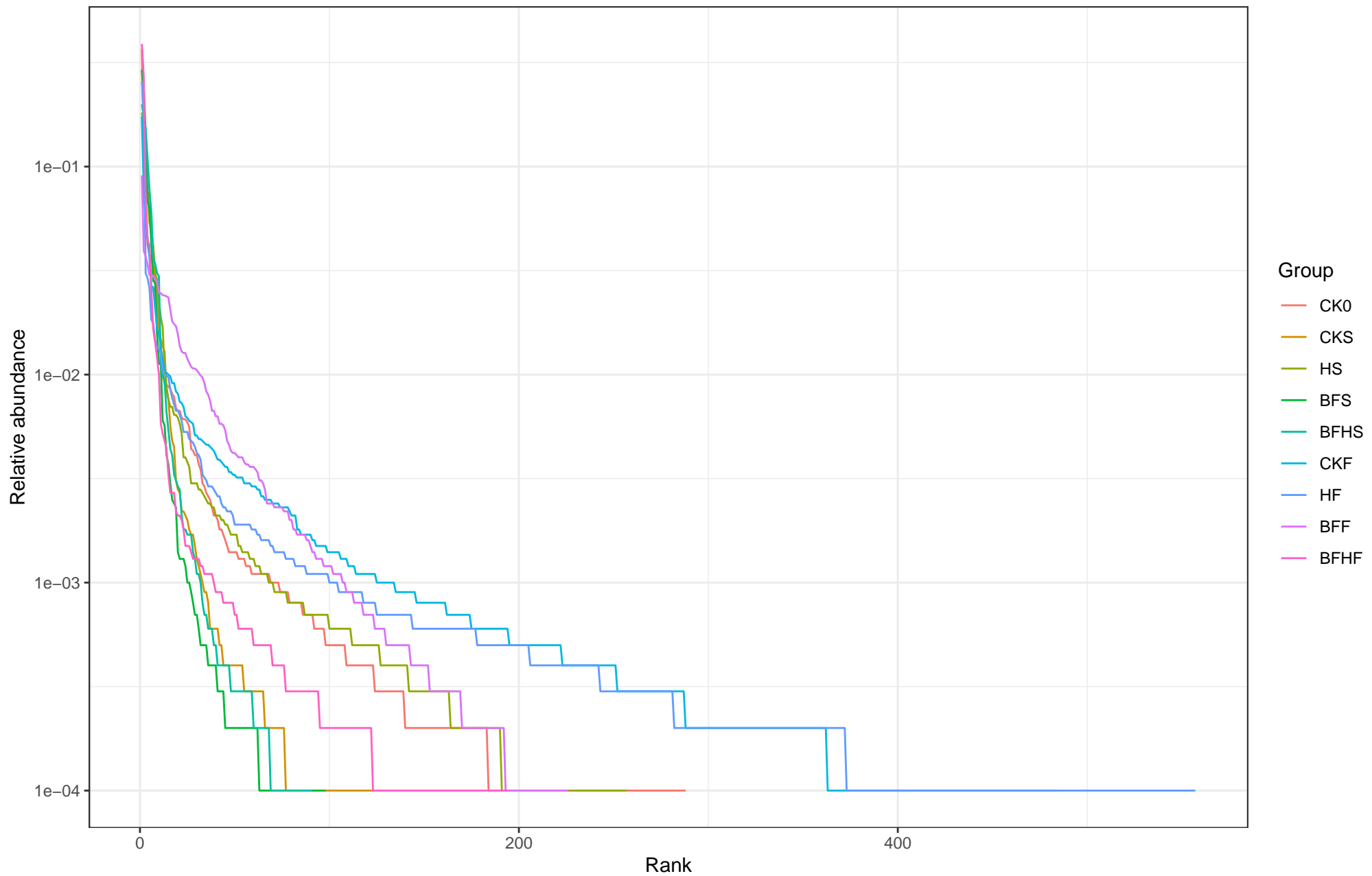

Supplement: Supplementary file 1 [file Data_Sheet_1.zip › 3.Alpha_diversity/rank_abundance_group.pdf]

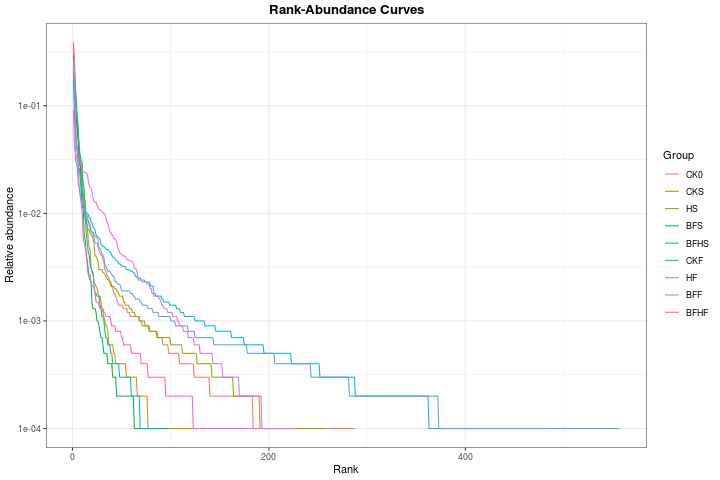

Supplement: Supplementary file 1 [file Data_Sheet_1.zip › 3.Alpha_diversity/rank_abundance_group.png]

## Rank-Abundance Curves

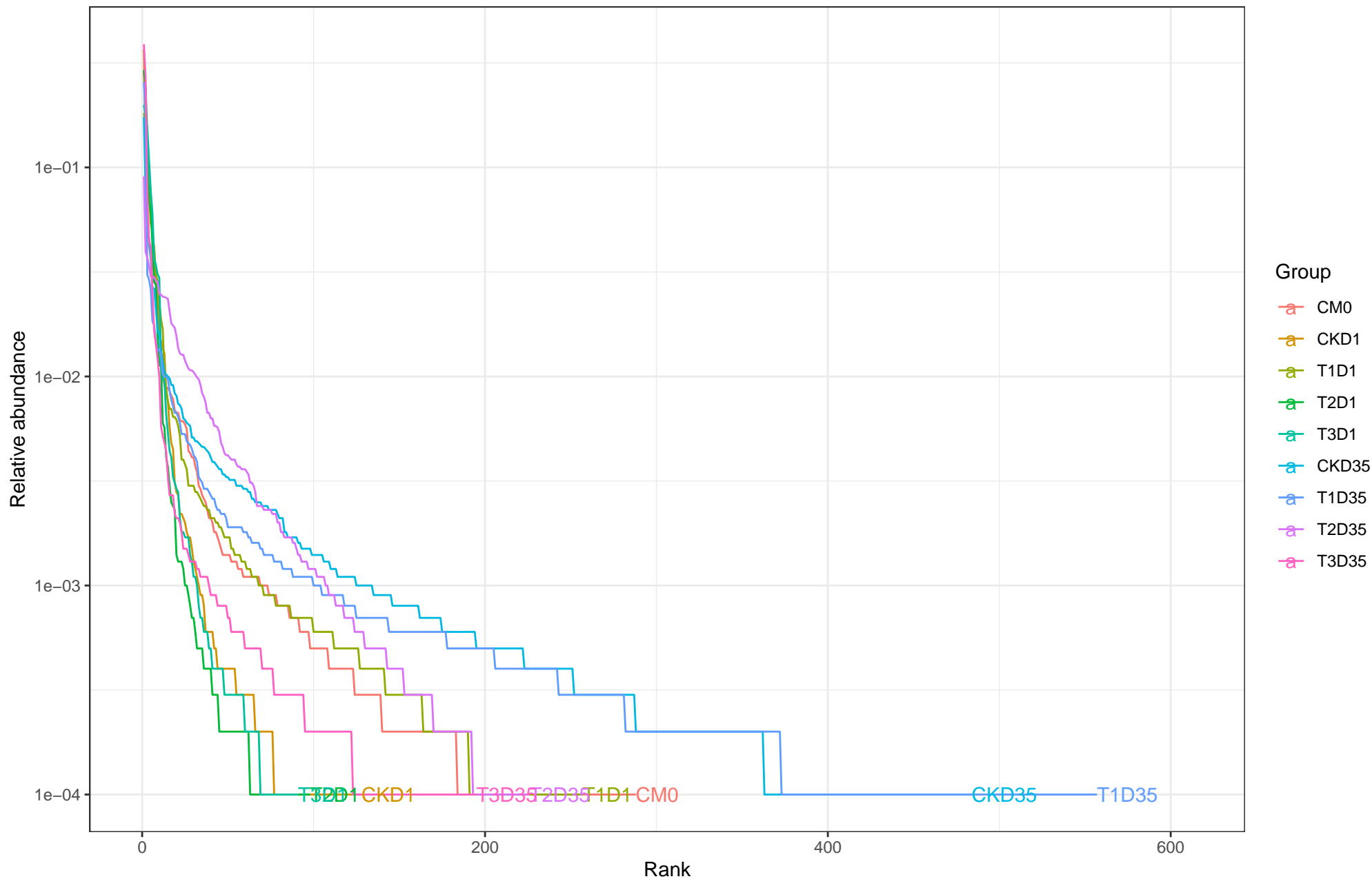

Supplement: Supplementary file 1 [file Data_Sheet_1.zip › 3.Alpha_diversity/rank_abundance_sample.pdf]

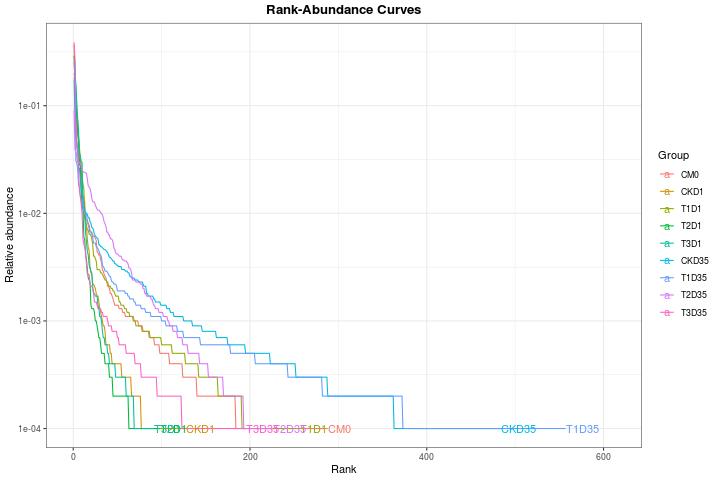

Supplement: Supplementary file 1 [file Data_Sheet_1.zip › 3.Alpha_diversity/rank_abundance_sample.png]

Kruskal-Wallis,  $p = 0.4335$

group

|     |     |      |     |      |
|-----|-----|------|-----|------|
| CK0 | HS  | BFHS | HF  | BFHF |
| CKS | BFS | CKF  | BFF |      |

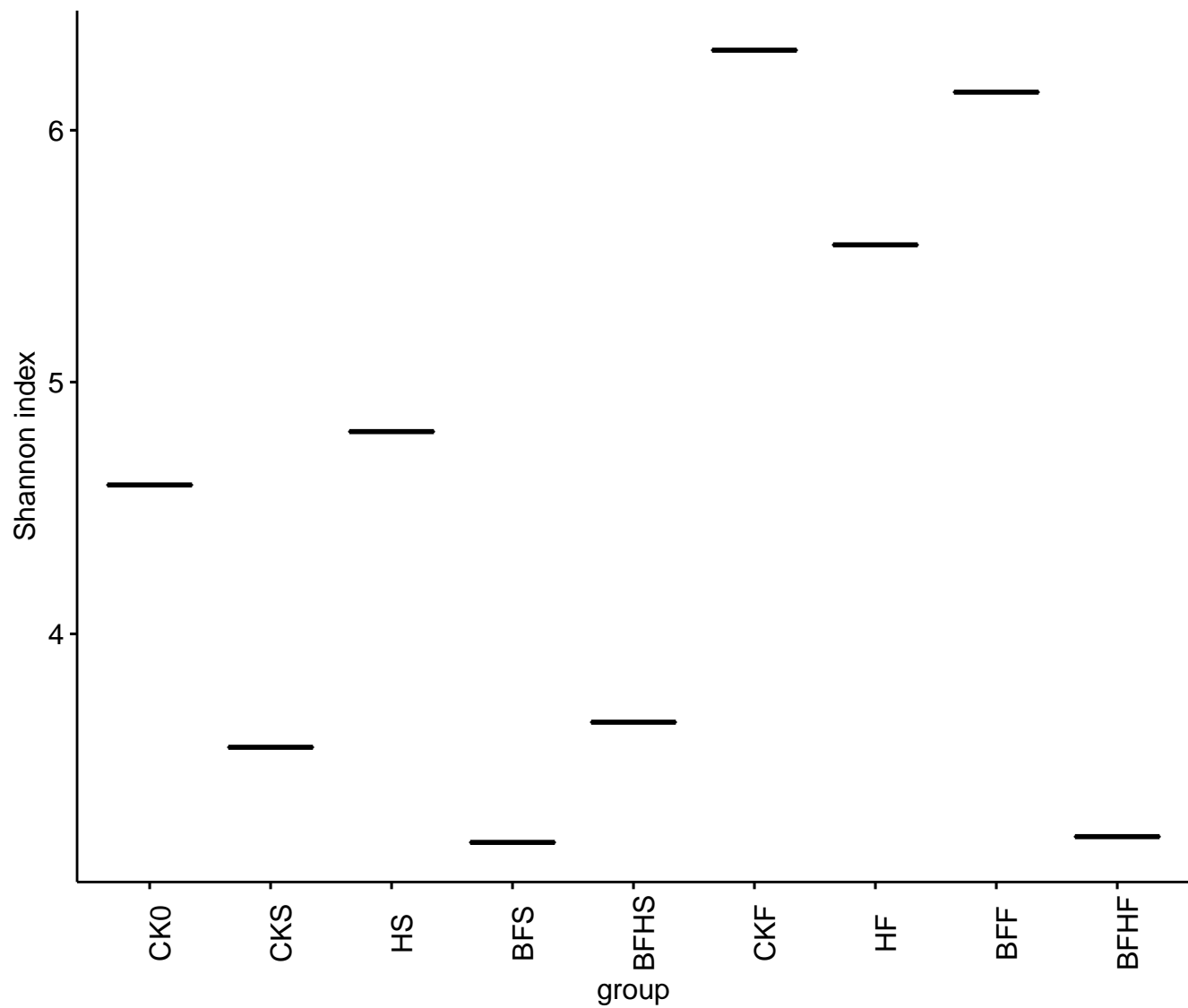

Supplement: Supplementary file 1 [file Data_Sheet_1.zip › 3.Alpha_diversity/Shannon.boxplot.pdf]

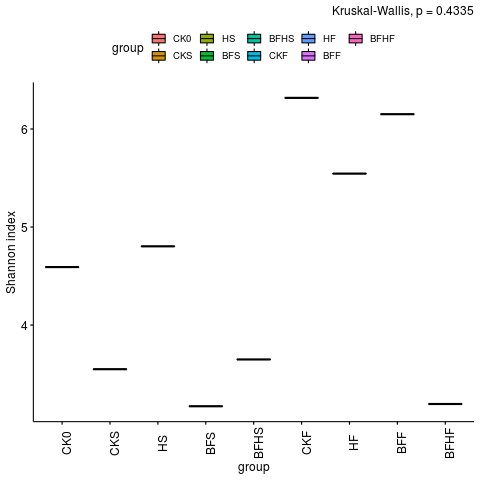

Supplement: Supplementary file 1 [file Data_Sheet_1.zip › 3.Alpha_diversity/Shannon.boxplot.png]

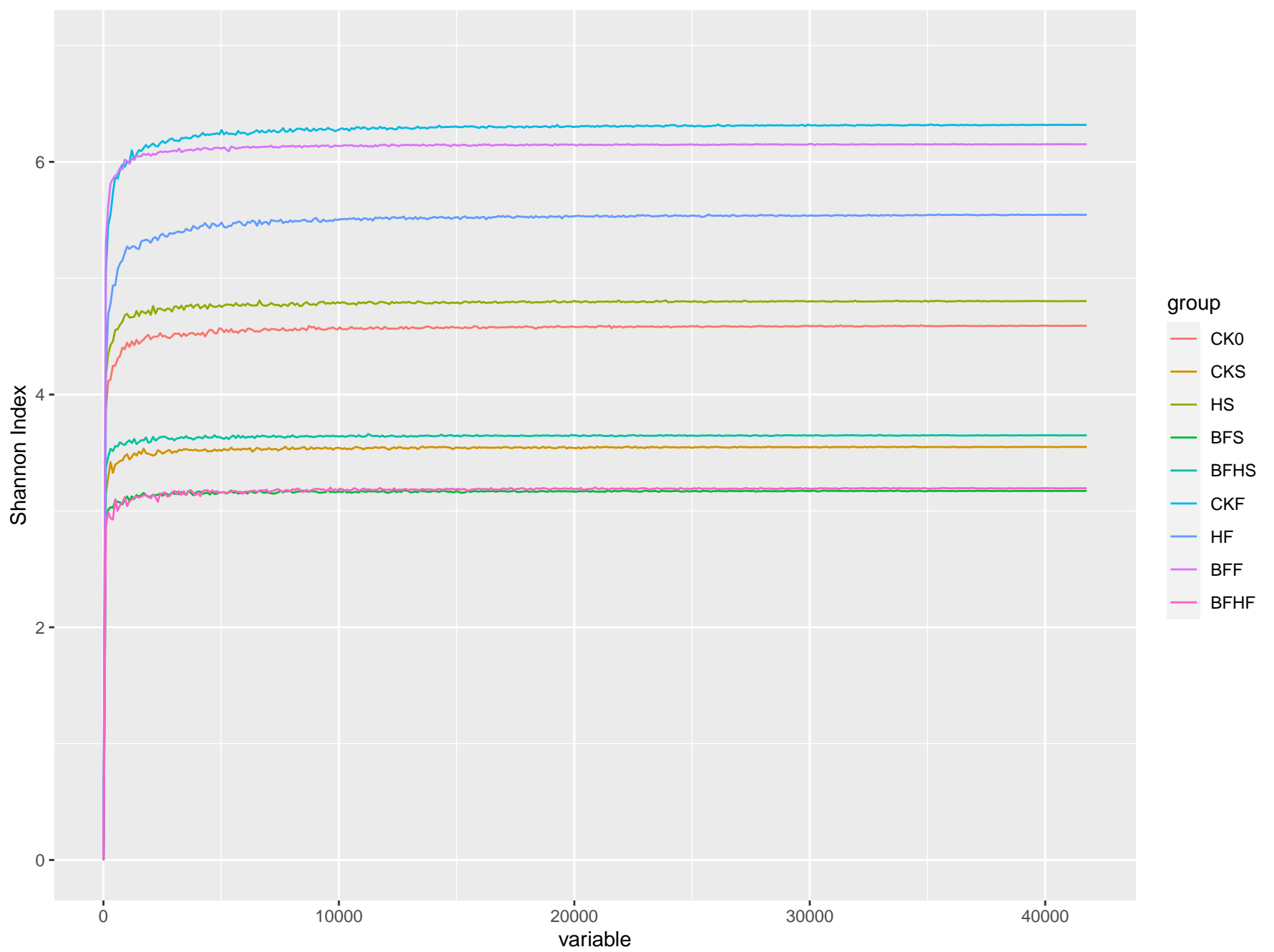

Supplement: Supplementary file 1 [file Data_Sheet_1.zip › 3.Alpha_diversity/shannon_group.pdf]

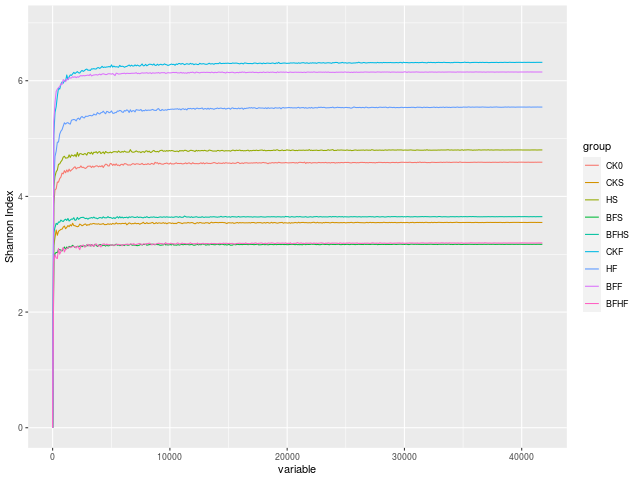

Supplement: Supplementary file 1 [file Data_Sheet_1.zip › 3.Alpha_diversity/shannon_group.png]

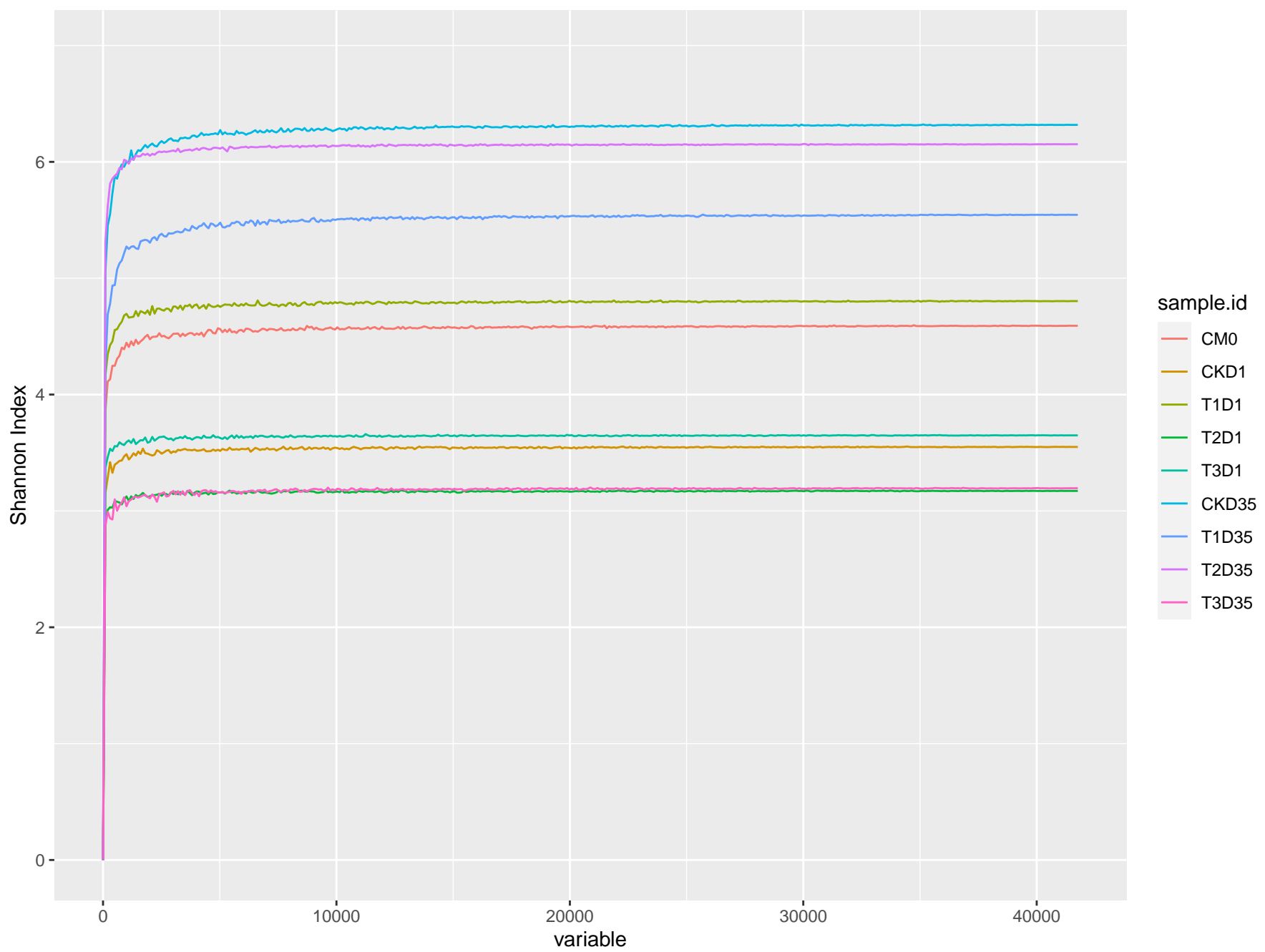

Supplement: Supplementary file 1 [file Data_Sheet_1.zip › 3.Alpha_diversity/shannon_sample.pdf]

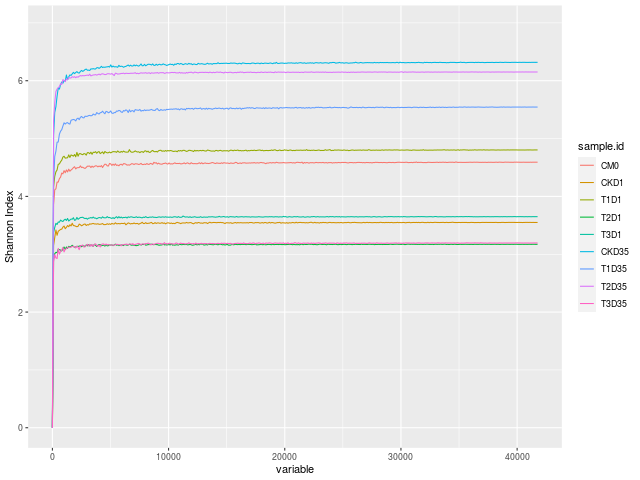

Supplement: Supplementary file 1 [file Data_Sheet_1.zip › 3.Alpha_diversity/shannon_sample.png]

Kruskal-Wallis,  $p = 0.4335$

group

|     |     |      |     |      |
|-----|-----|------|-----|------|
| CK0 | HS  | BFHS | HF  | BFHF |
| CKS | BFS | CKF  | BFF |      |

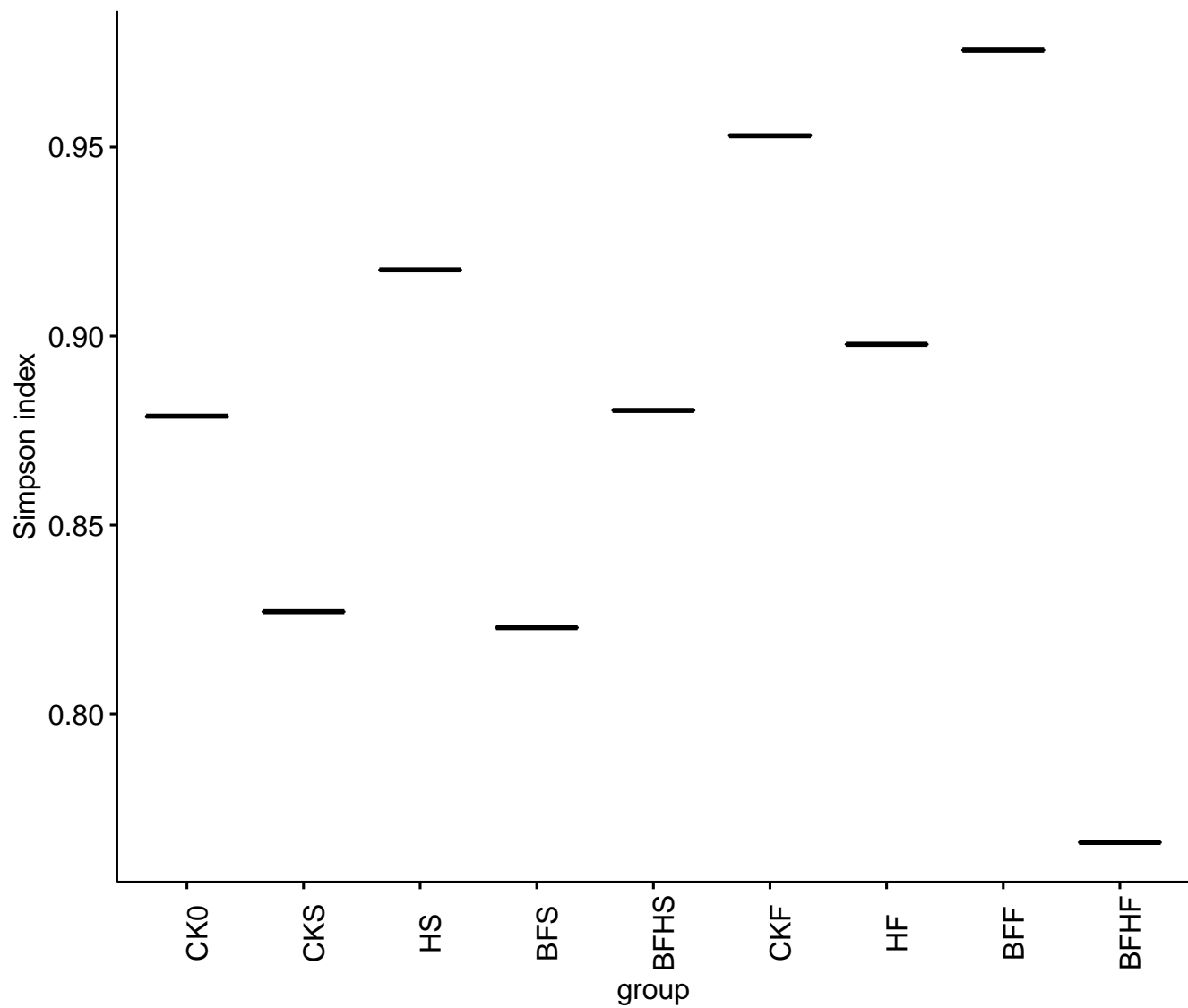

Supplement: Supplementary file 1 [file Data_Sheet_1.zip › 3.Alpha_diversity/Simpson.boxplot.pdf]

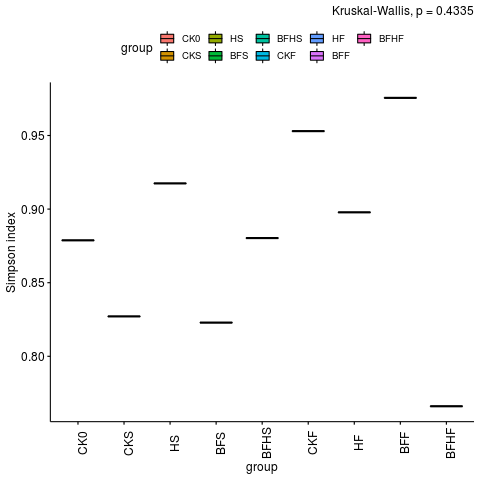

Supplement: Supplementary file 1 [file Data_Sheet_1.zip › 3.Alpha_diversity/Simpson.boxplot.png]

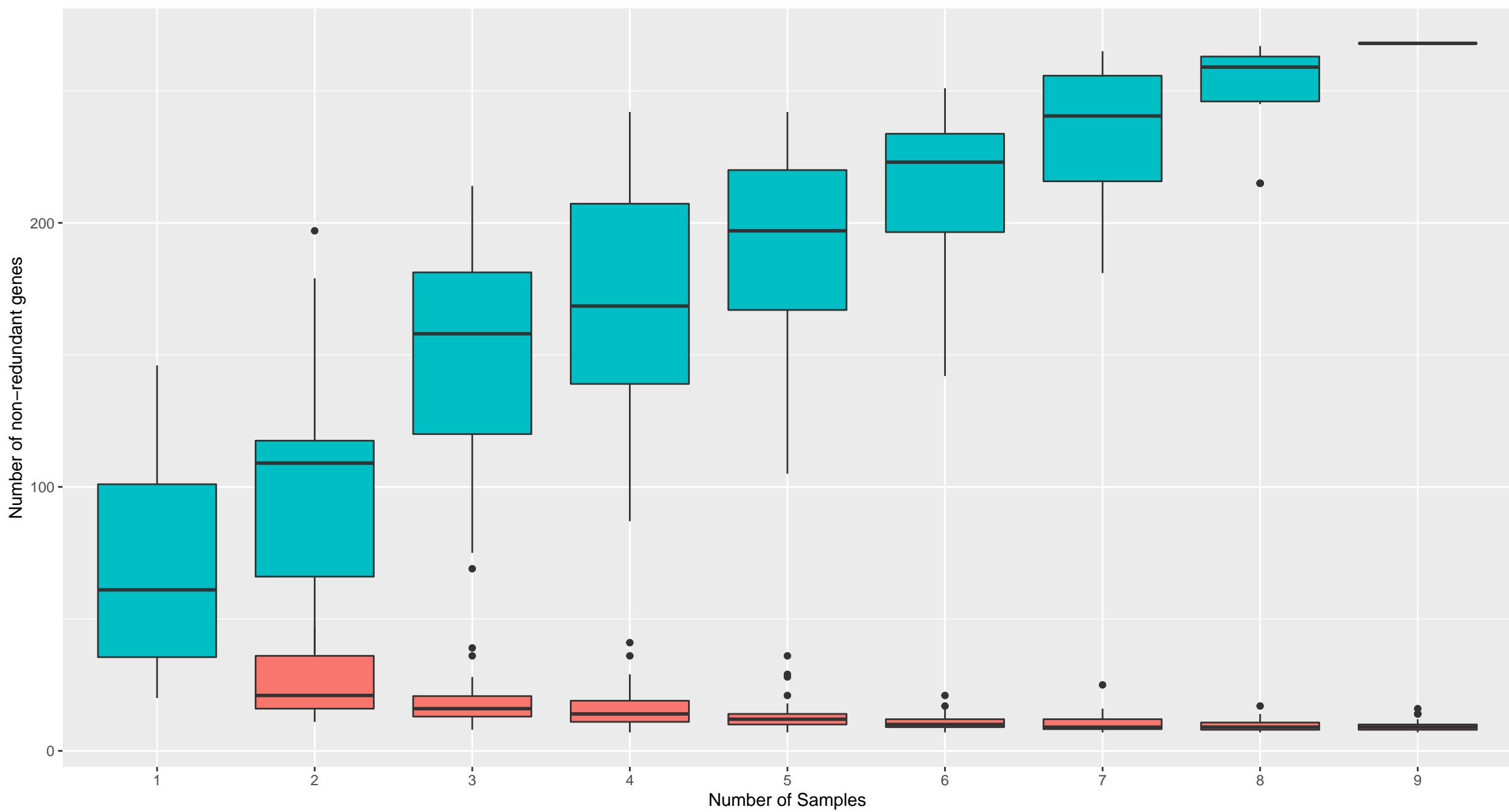

Supplement: Supplementary file 1 [file Data_Sheet_1.zip › 3.Alpha_diversity/specaccum.pdf]

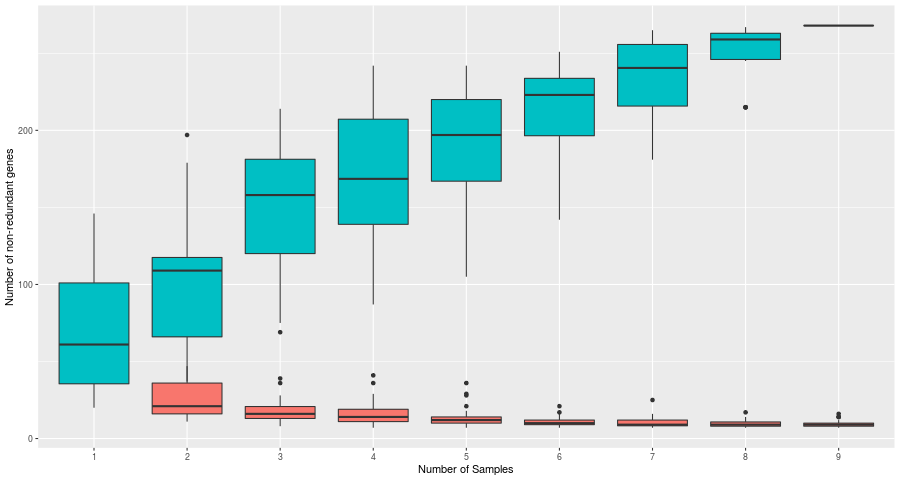

Supplement: Supplementary file 1 [file Data_Sheet_1.zip › 3.Alpha_diversity/specaccum.png]

Heatmap aitchison

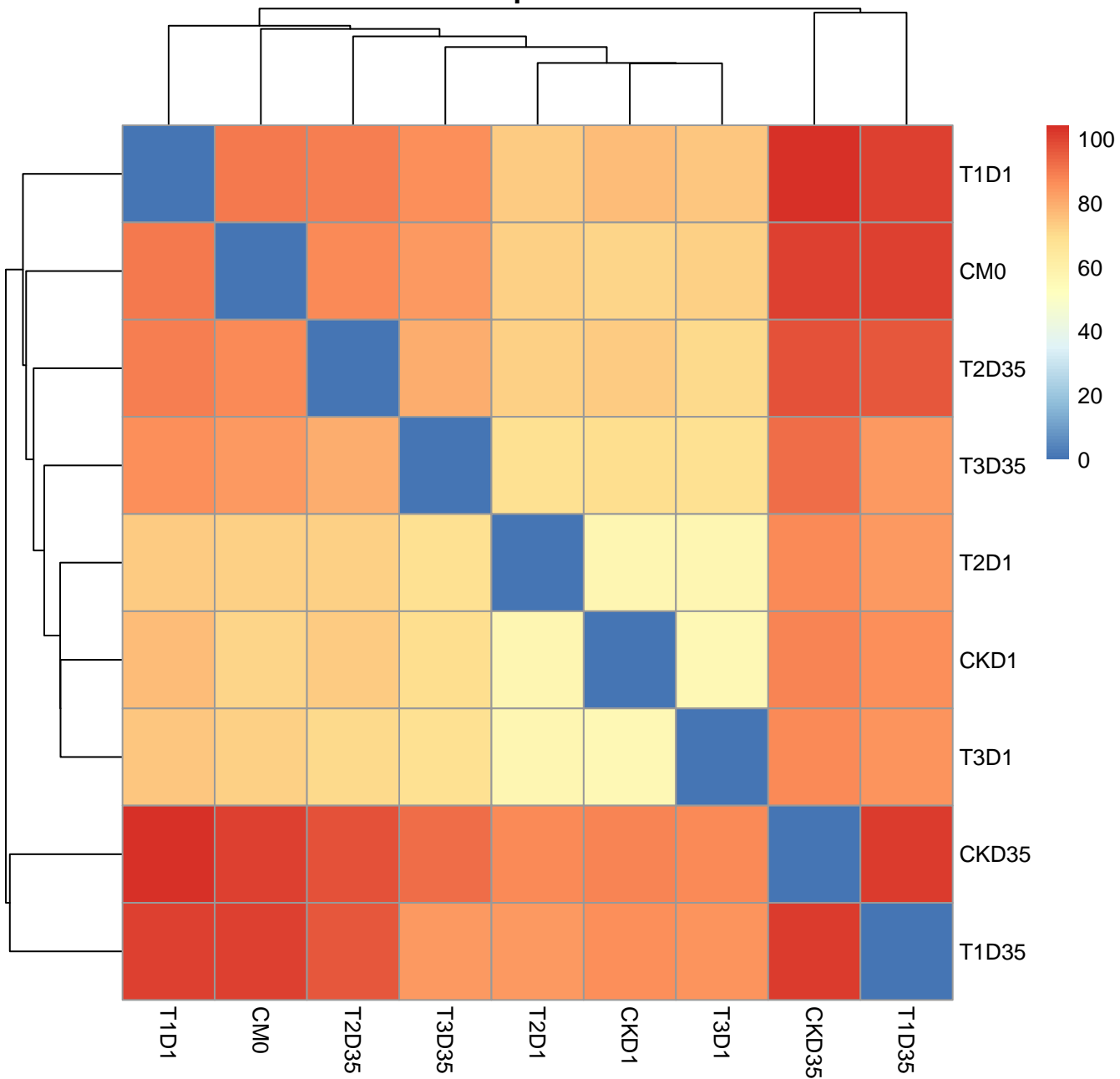

Supplement: Supplementary file 1 [file Data_Sheet_1.zip › 4.Beta_diversity/Heatmap/aitchison_heatmap.pdf]

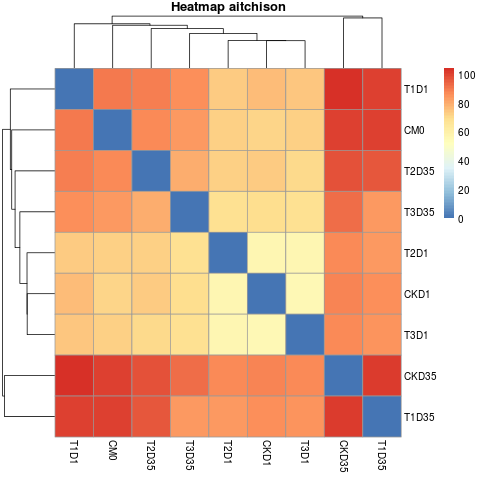

Supplement: Supplementary file 1 [file Data_Sheet_1.zip › 4.Beta_diversity/Heatmap/aitchison_heatmap.png]

Heatmap bray\_curtis

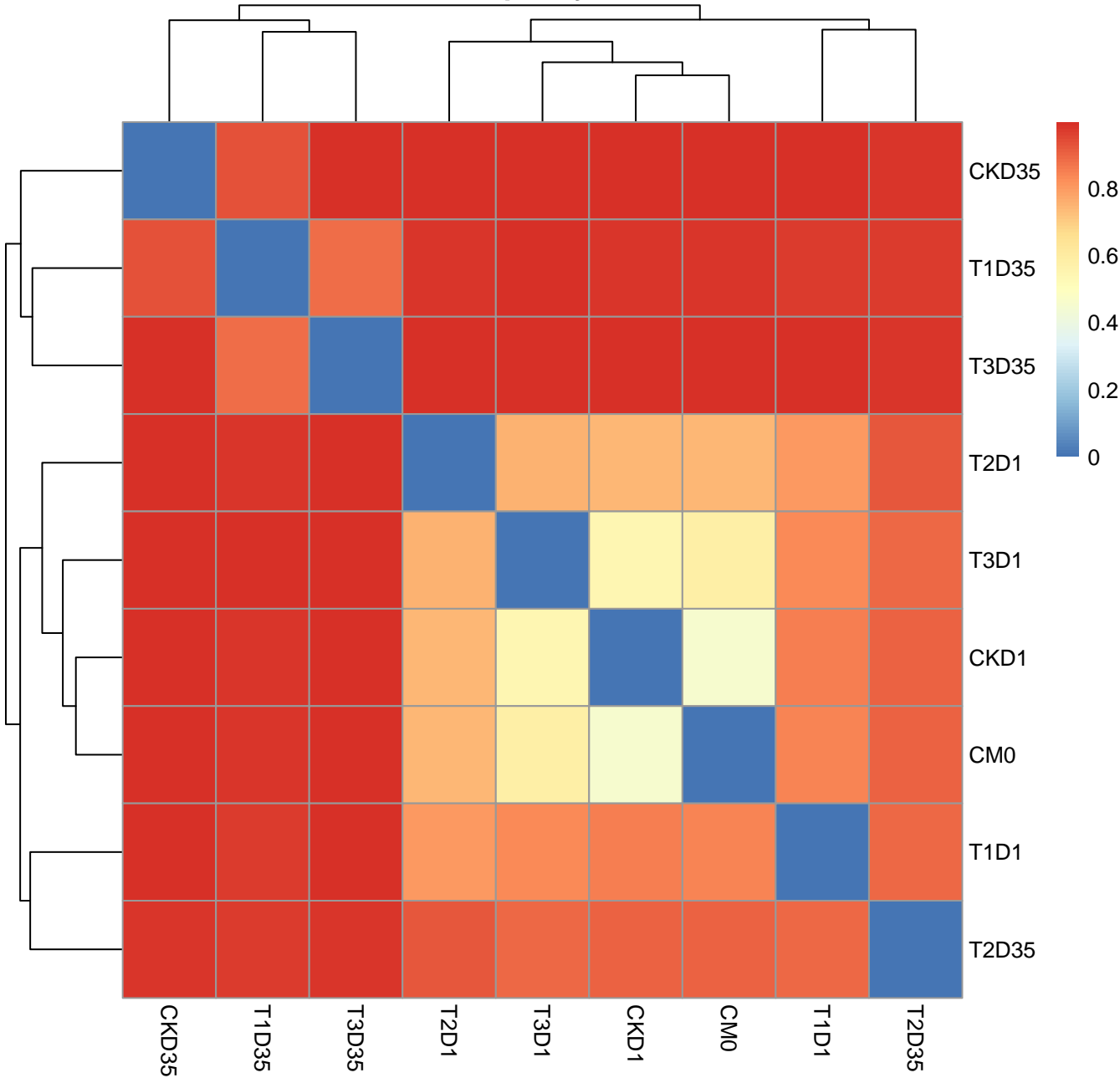

Supplement: Supplementary file 1 [file Data_Sheet_1.zip › 4.Beta_diversity/Heatmap/bray_curtis_heatmap.pdf]

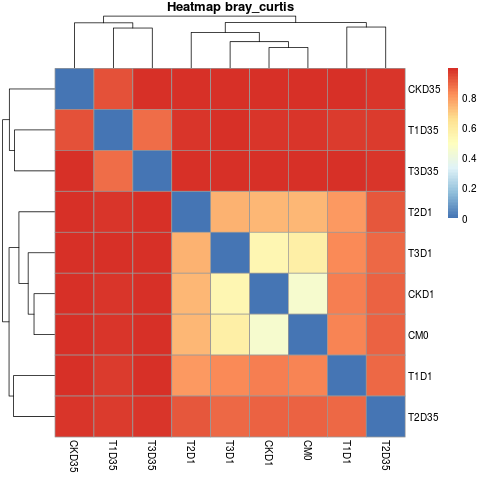

Supplement: Supplementary file 1 [file Data_Sheet_1.zip › 4.Beta_diversity/Heatmap/bray_curtis_heatmap.png]

[illegible]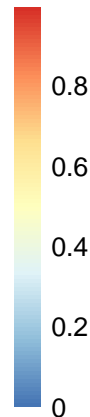

Supplement: Supplementary file 1 [file Data_Sheet_1.zip › 4.Beta_diversity/Heatmap/jaccard_heatmap.pdf]

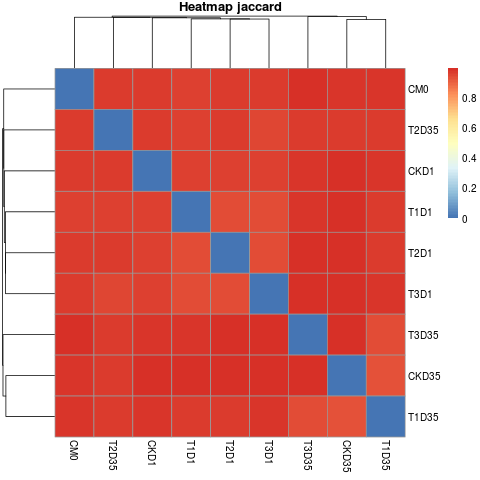

Supplement: Supplementary file 1 [file Data_Sheet_1.zip › 4.Beta_diversity/Heatmap/jaccard_heatmap.png]

Heatmap unweighted\_unifrac

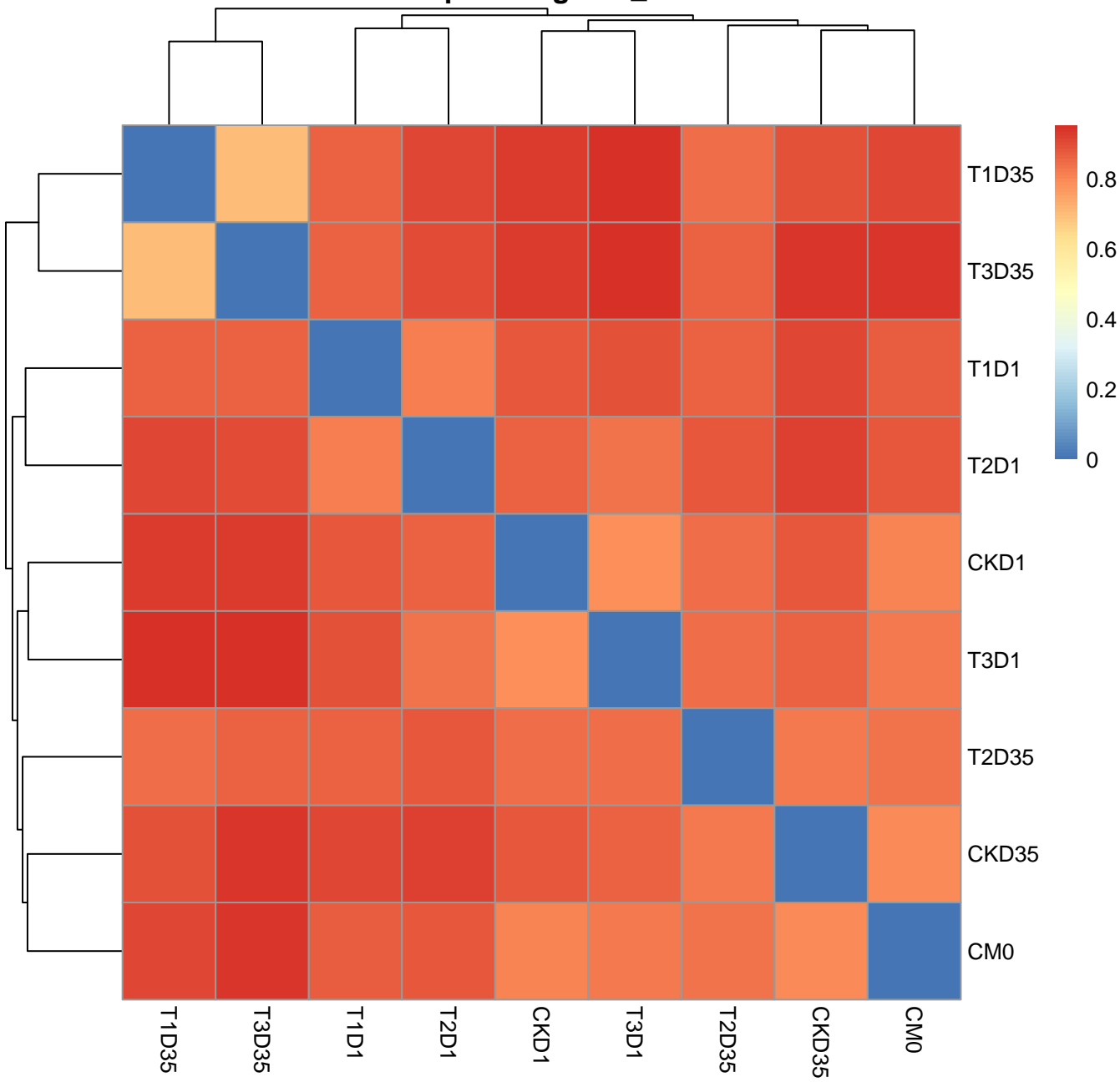

Supplement: Supplementary file 1 [file Data_Sheet_1.zip › 4.Beta_diversity/Heatmap/unweighted_unifrac_heatmap.pdf]

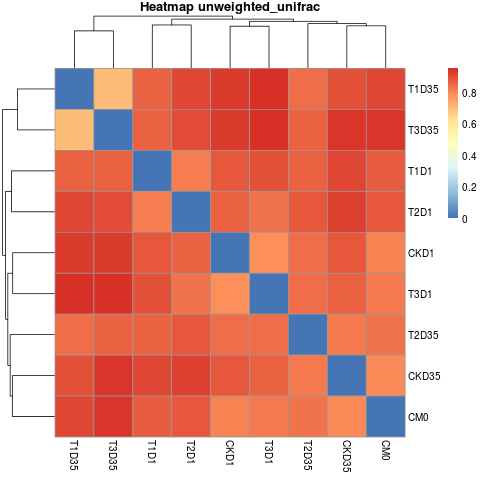

Supplement: Supplementary file 1 [file Data_Sheet_1.zip › 4.Beta_diversity/Heatmap/unweighted_unifrac_heatmap.png]

## Heatmap weighted\_unifrac

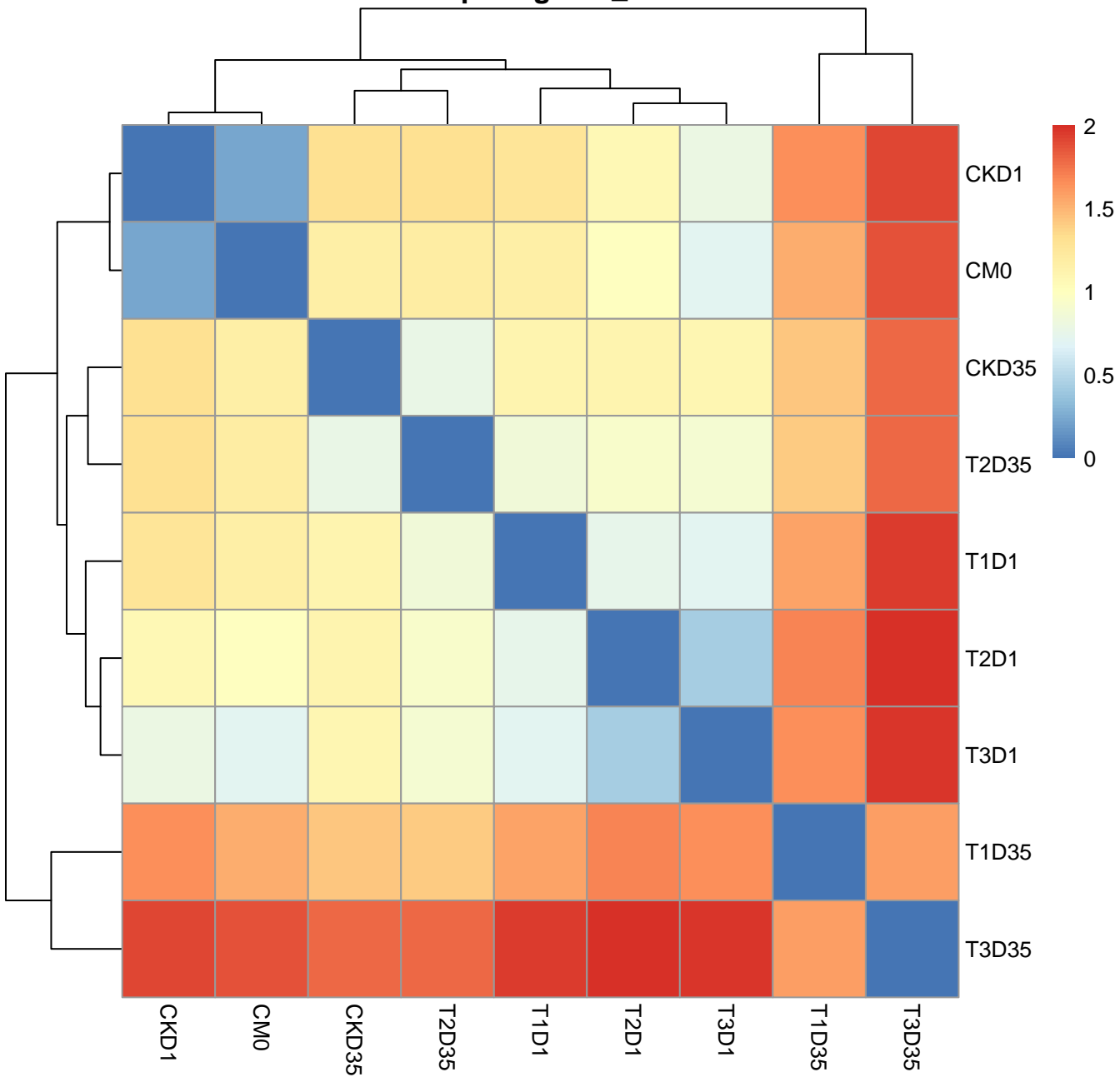

Supplement: Supplementary file 1 [file Data_Sheet_1.zip › 4.Beta_diversity/Heatmap/weighted_unifrac_heatmap.pdf]

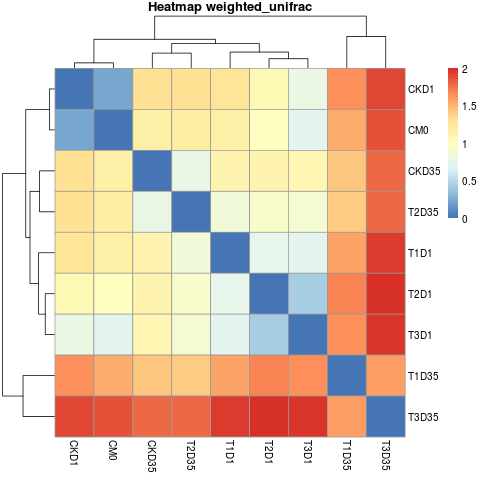

Supplement: Supplementary file 1 [file Data_Sheet_1.zip › 4.Beta_diversity/Heatmap/weighted_unifrac_heatmap.png]

# NMDS aitchison

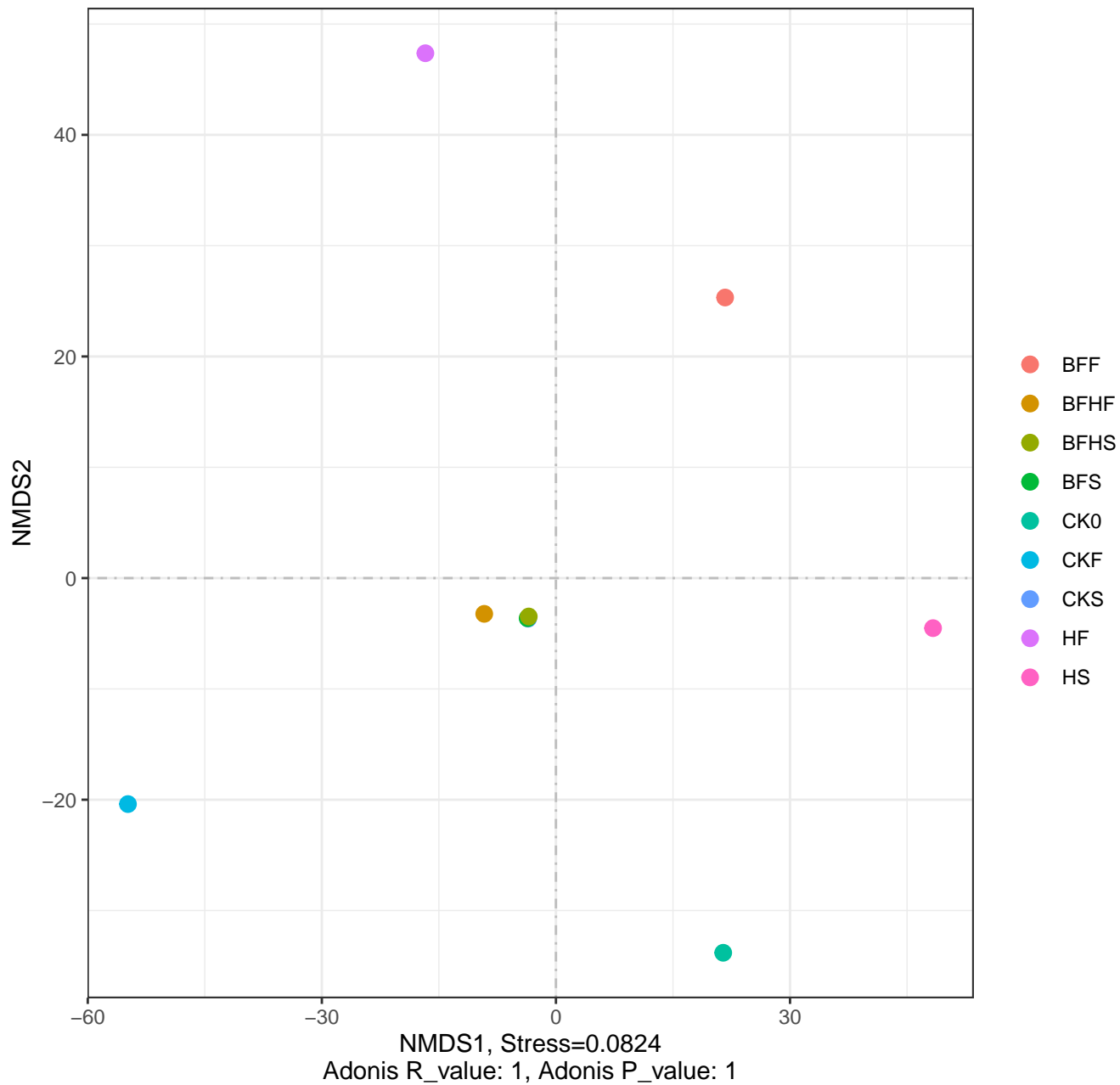

Supplement: Supplementary file 1 [file Data_Sheet_1.zip › 4.Beta_diversity/NMDS/aitchison_NMDS.pdf]

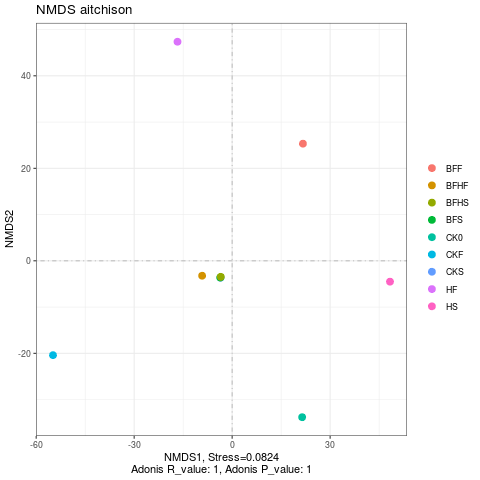

Supplement: Supplementary file 1 [file Data_Sheet_1.zip › 4.Beta_diversity/NMDS/aitchison_NMDS.png]

# NMDS aitchison

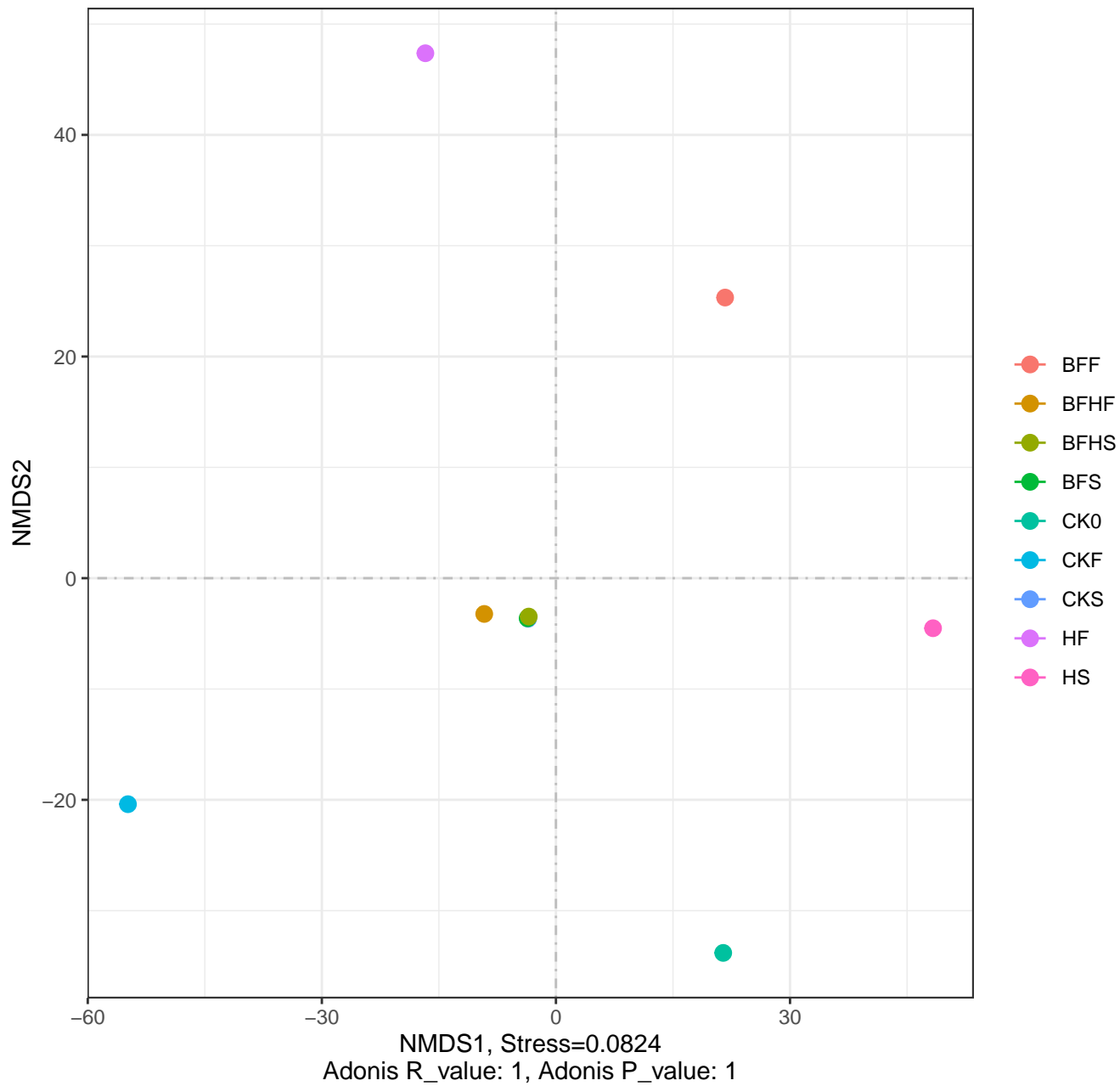

Supplement: Supplementary file 1 [file Data_Sheet_1.zip › 4.Beta_diversity/NMDS/aitchison_NMDS_cluster.pdf]

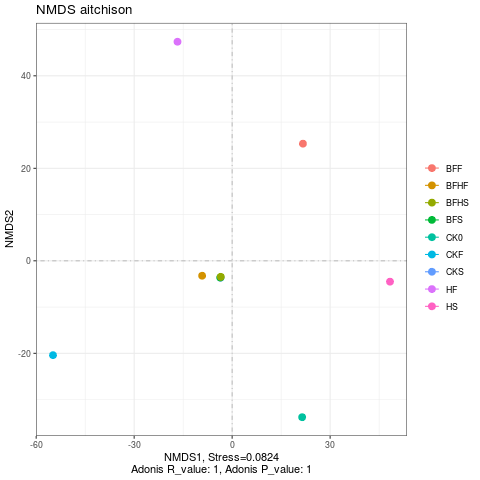

Supplement: Supplementary file 1 [file Data_Sheet_1.zip › 4.Beta_diversity/NMDS/aitchison_NMDS_cluster.png]

# NMDS aitchison

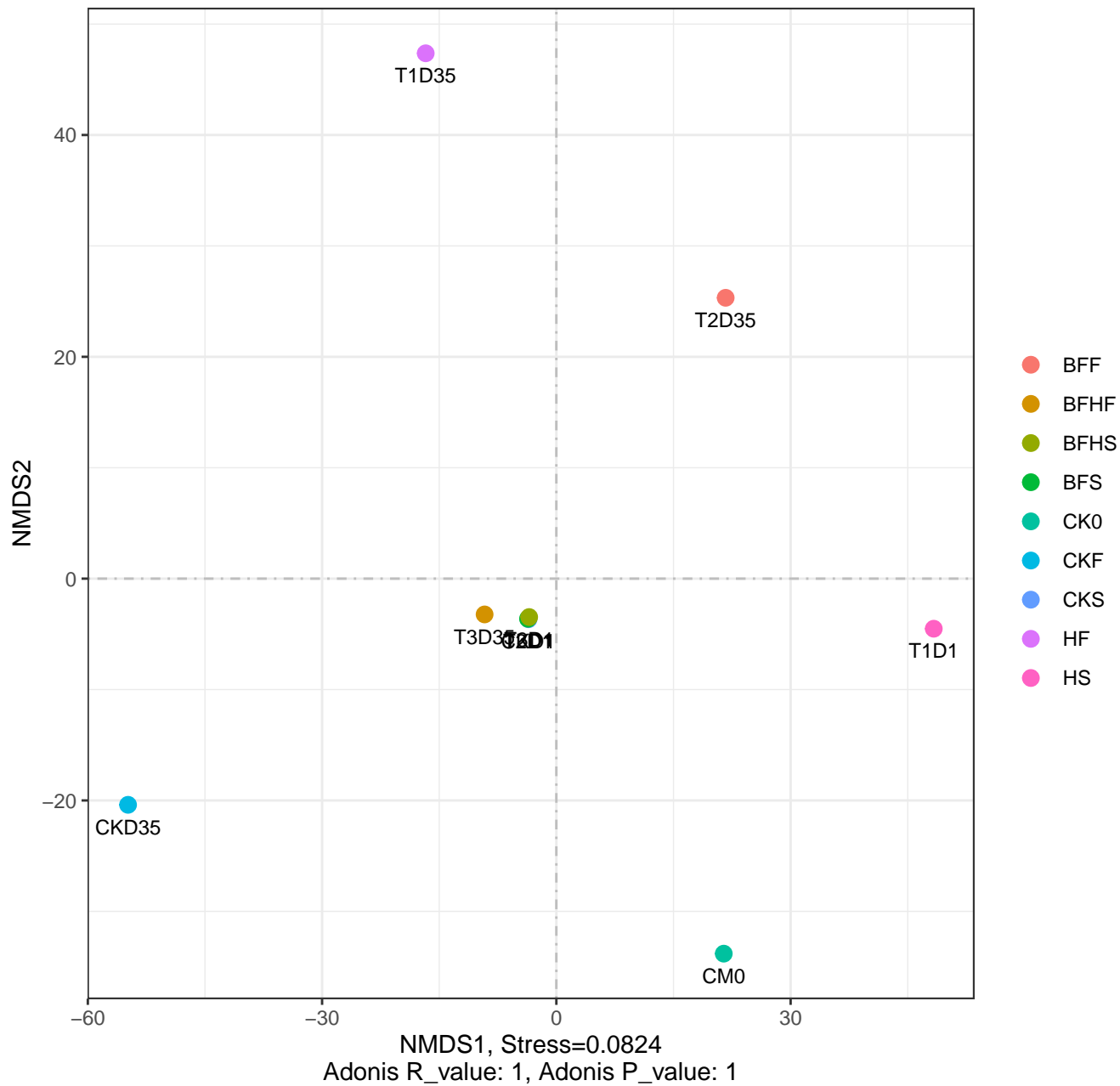

Supplement: Supplementary file 1 [file Data_Sheet_1.zip › 4.Beta_diversity/NMDS/aitchison_NMDS_name.pdf]

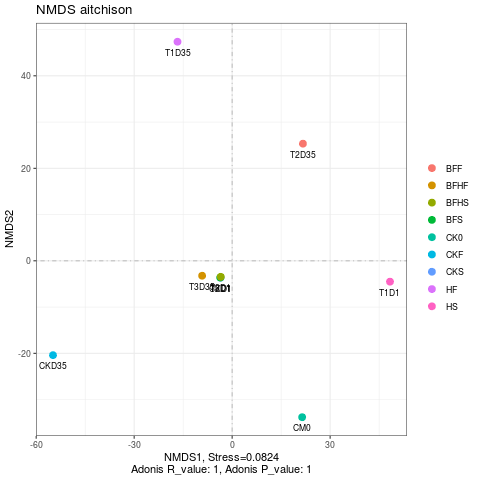

Supplement: Supplementary file 1 [file Data_Sheet_1.zip › 4.Beta_diversity/NMDS/aitchison_NMDS_name.png]

# NMDS aitchison

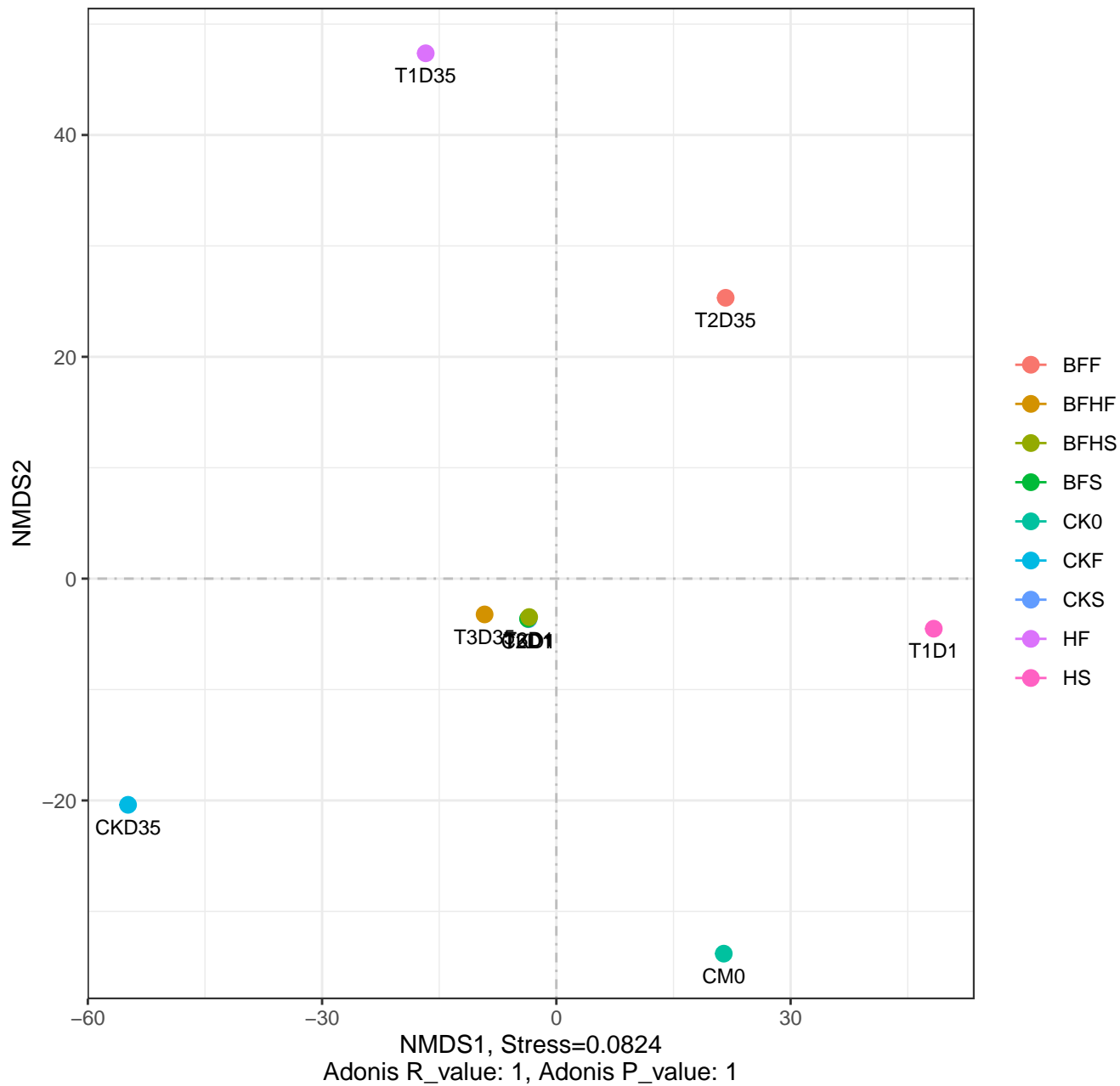

Supplement: Supplementary file 1 [file Data_Sheet_1.zip › 4.Beta_diversity/NMDS/aitchison_NMDS_name_cluster.pdf]

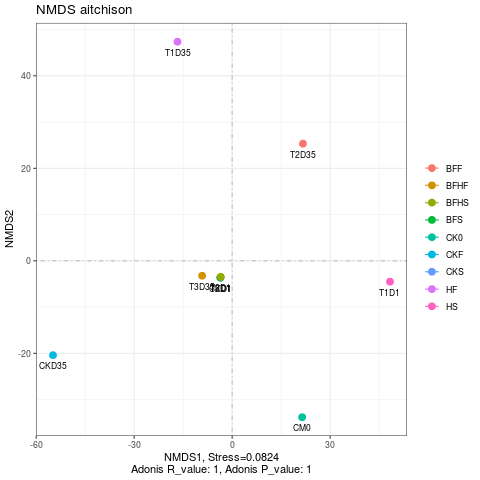

Supplement: Supplementary file 1 [file Data_Sheet_1.zip › 4.Beta_diversity/NMDS/aitchison_NMDS_name_cluster.png]

# NMDS bray\_curtis

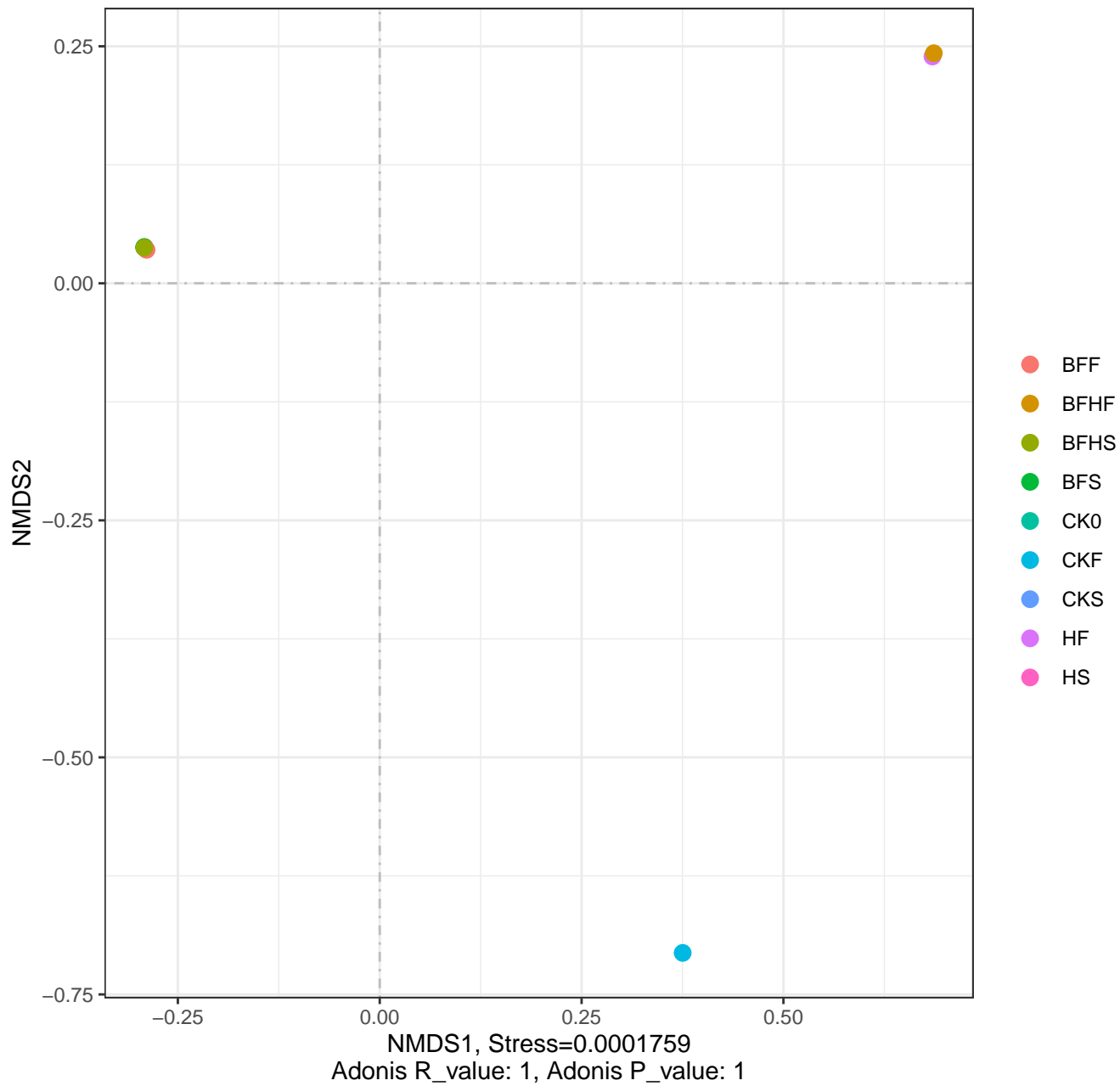

Supplement: Supplementary file 1 [file Data_Sheet_1.zip › 4.Beta_diversity/NMDS/bray_curtis_NMDS.pdf]

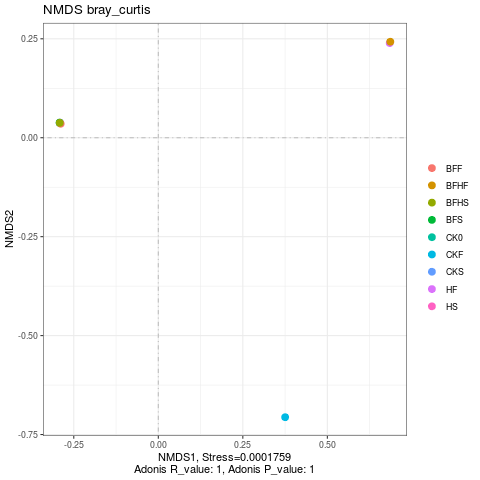

Supplement: Supplementary file 1 [file Data_Sheet_1.zip › 4.Beta_diversity/NMDS/bray_curtis_NMDS.png]

NMDS bray\_curtis

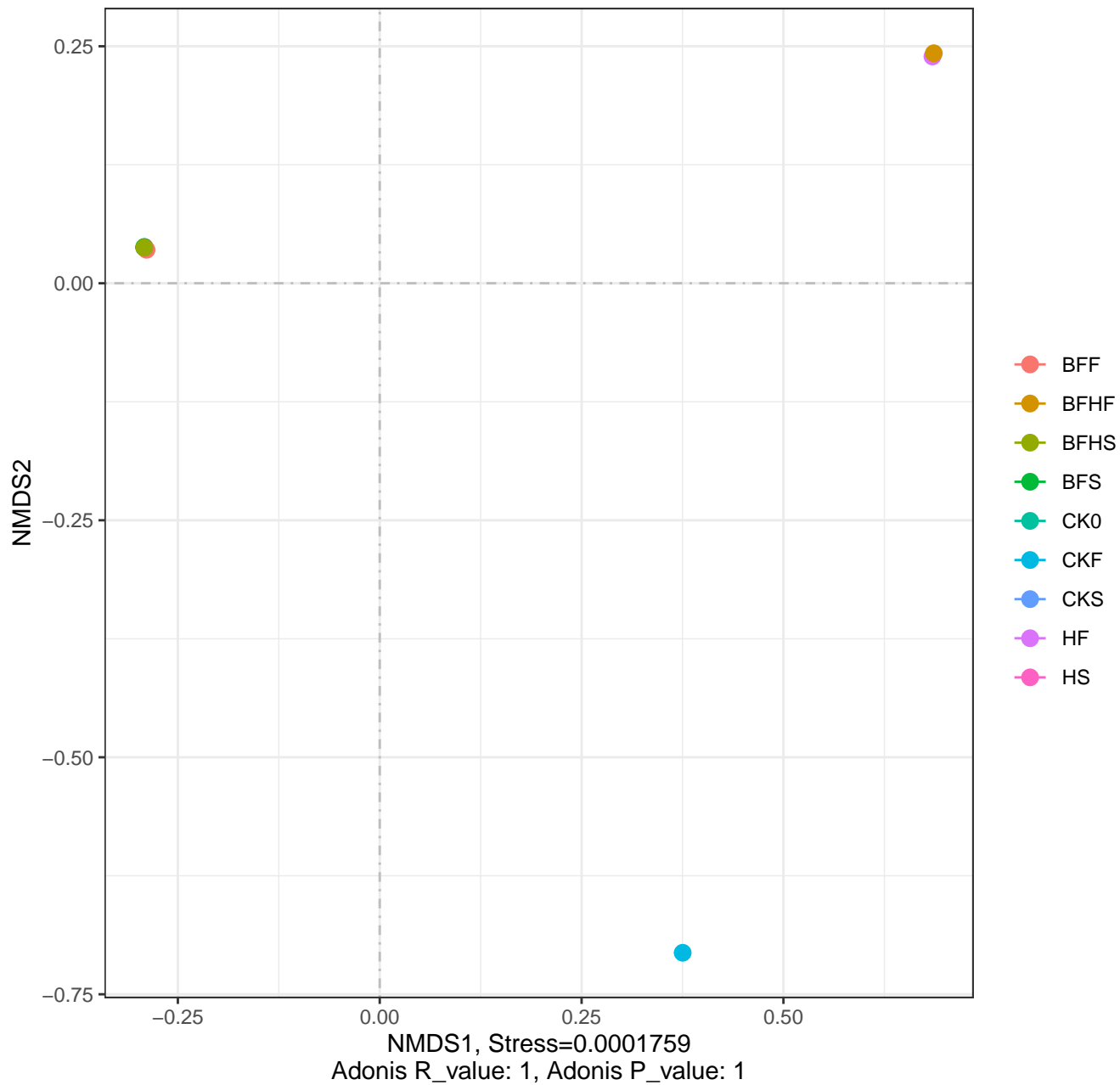

Supplement: Supplementary file 1 [file Data_Sheet_1.zip › 4.Beta_diversity/NMDS/bray_curtis_NMDS_cluster.pdf]

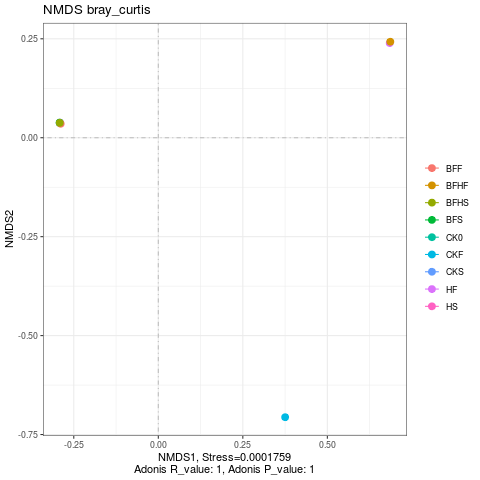

Supplement: Supplementary file 1 [file Data_Sheet_1.zip › 4.Beta_diversity/NMDS/bray_curtis_NMDS_cluster.png]

# NMDS bray\_curtis

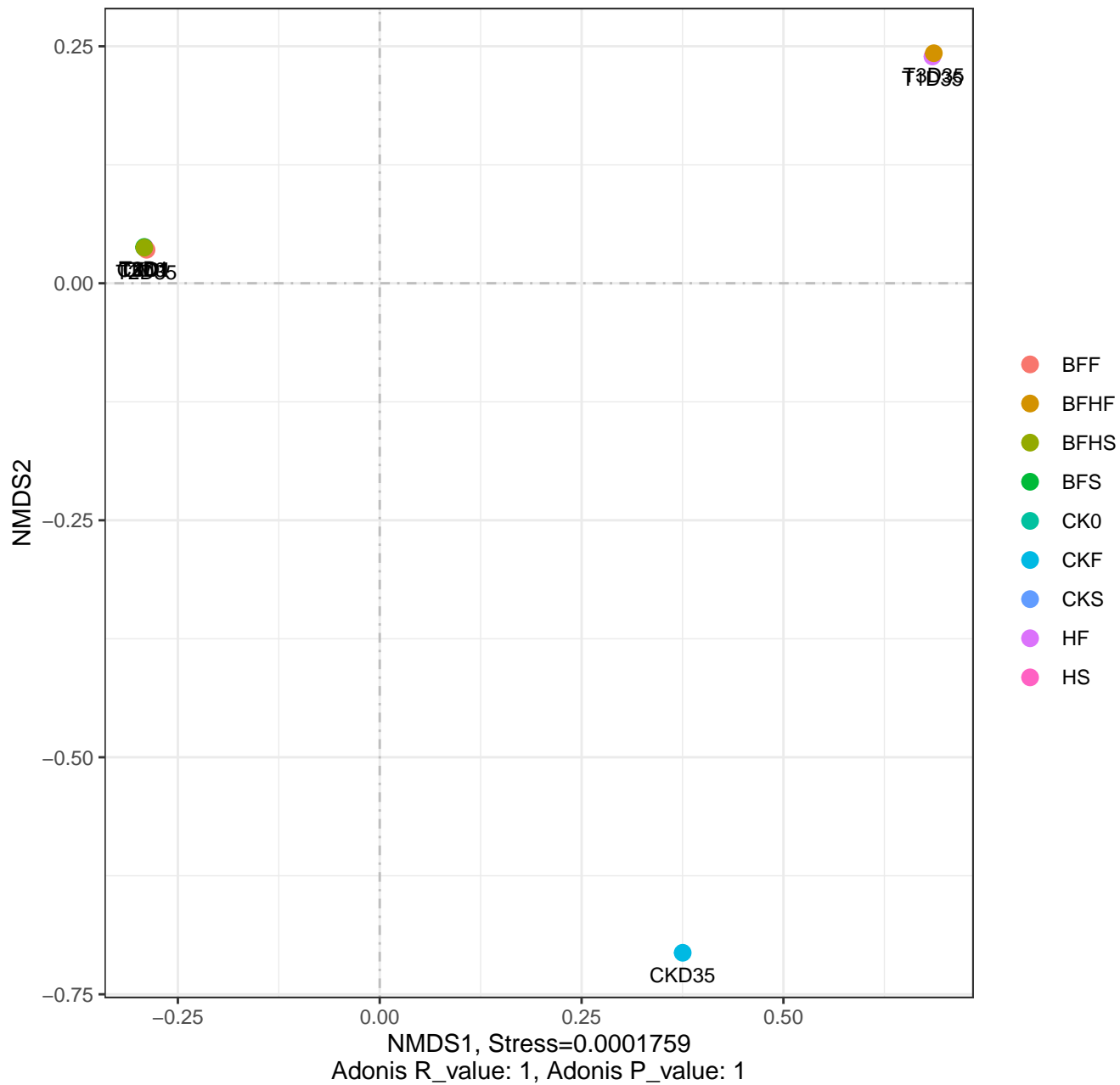

Supplement: Supplementary file 1 [file Data_Sheet_1.zip › 4.Beta_diversity/NMDS/bray_curtis_NMDS_name.pdf]

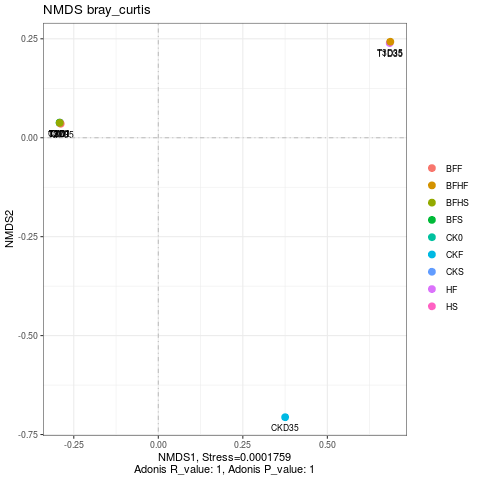

Supplement: Supplementary file 1 [file Data_Sheet_1.zip › 4.Beta_diversity/NMDS/bray_curtis_NMDS_name.png]

# NMDS bray\_curtis

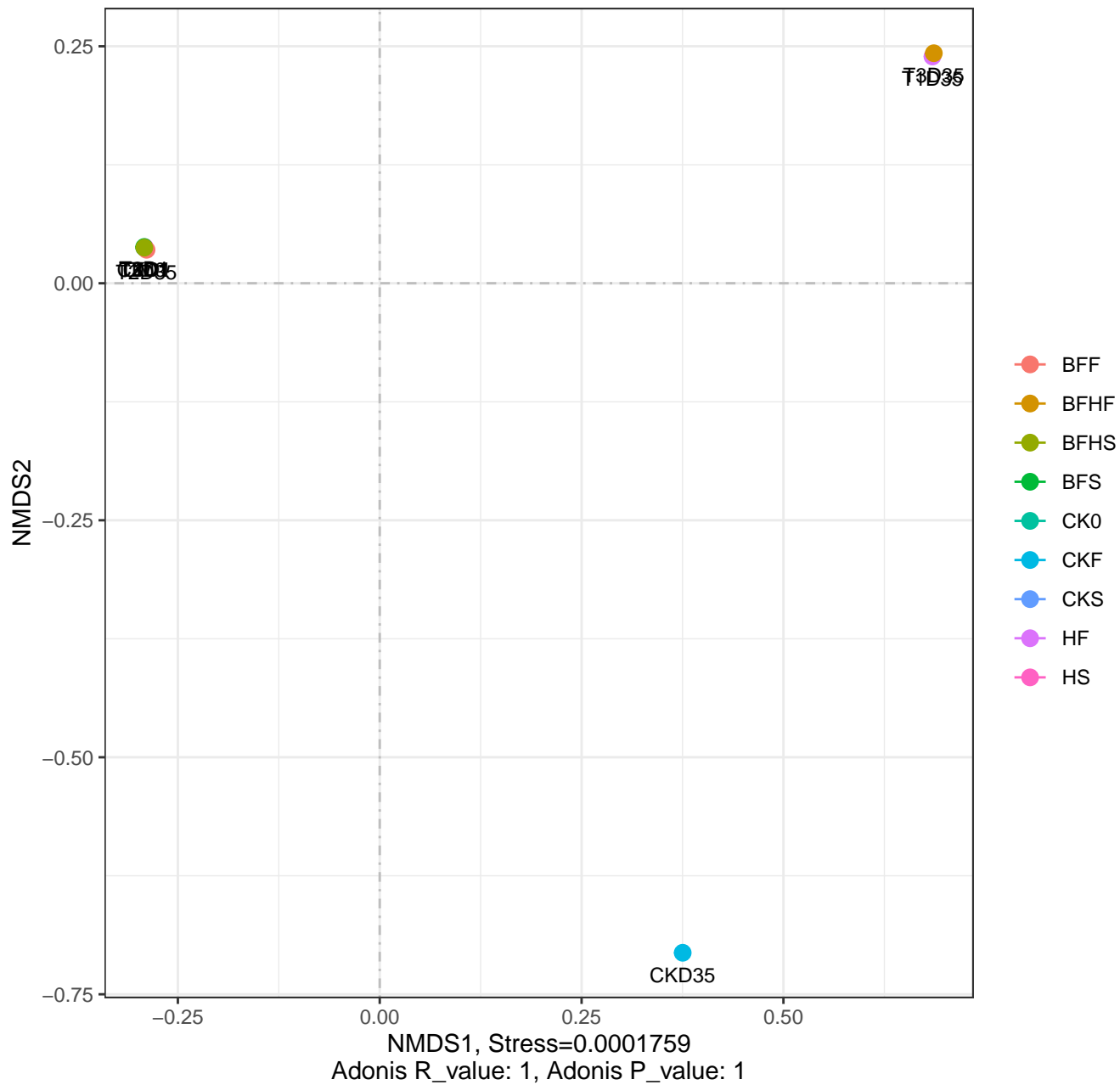

Supplement: Supplementary file 1 [file Data_Sheet_1.zip › 4.Beta_diversity/NMDS/bray_curtis_NMDS_name_cluster.pdf]

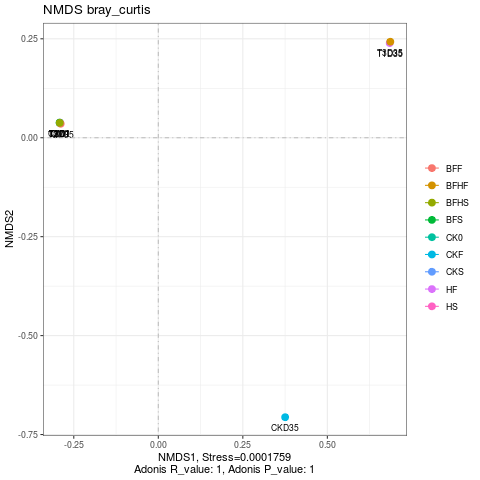

Supplement: Supplementary file 1 [file Data_Sheet_1.zip › 4.Beta_diversity/NMDS/bray_curtis_NMDS_name_cluster.png]

# NMDS jaccard

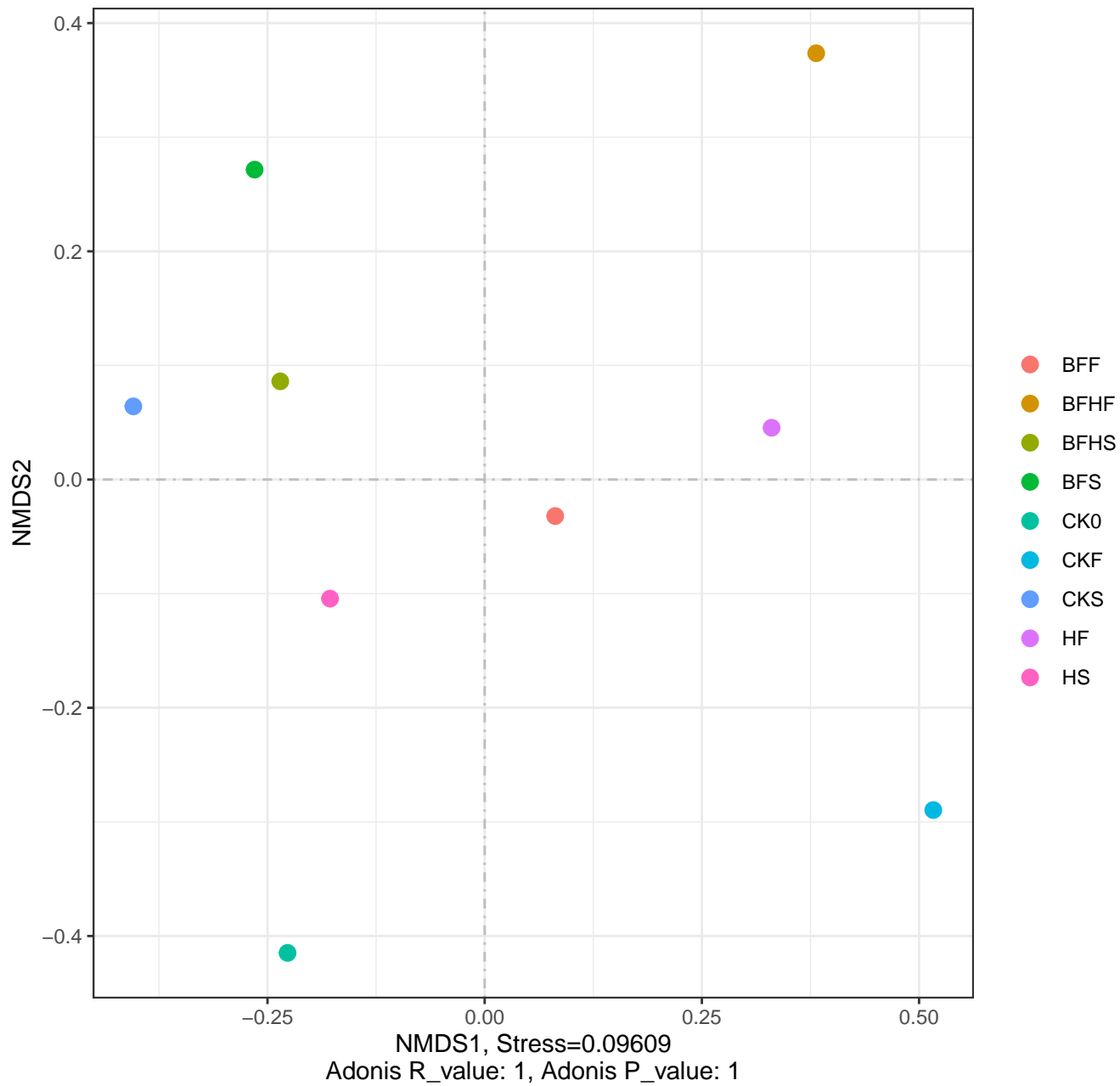

Supplement: Supplementary file 1 [file Data_Sheet_1.zip › 4.Beta_diversity/NMDS/jaccard_NMDS.pdf]

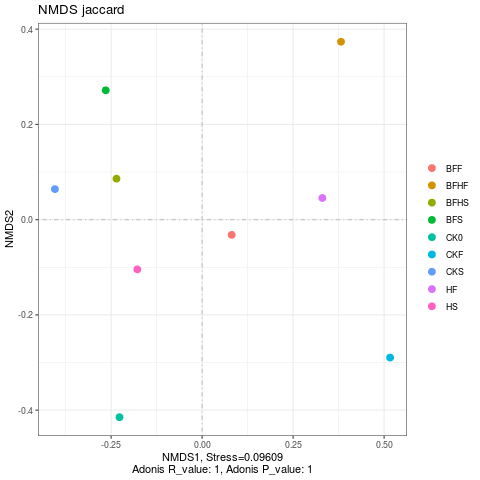

Supplement: Supplementary file 1 [file Data_Sheet_1.zip › 4.Beta_diversity/NMDS/jaccard_NMDS.png]

# NMDS jaccard

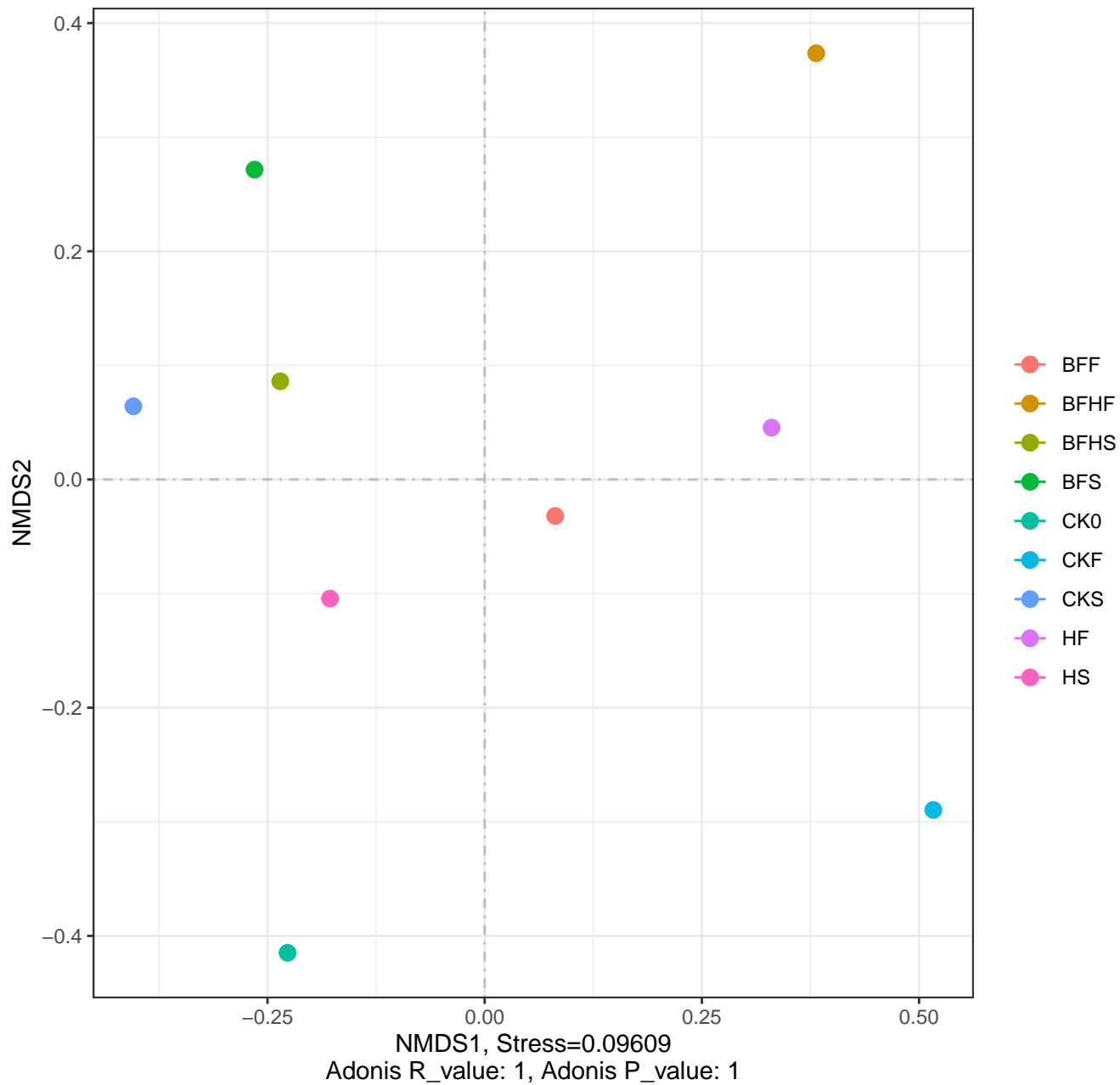

Supplement: Supplementary file 1 [file Data_Sheet_1.zip › 4.Beta_diversity/NMDS/jaccard_NMDS_cluster.pdf]

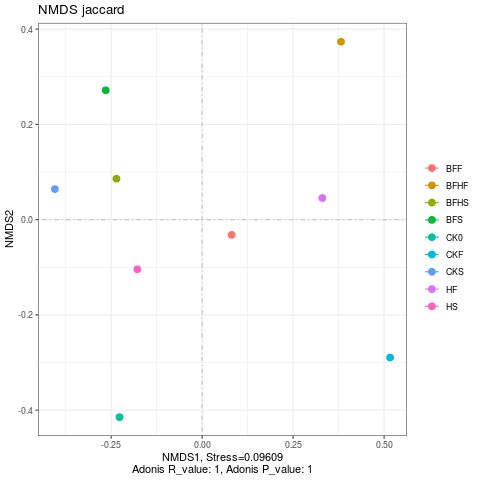

Supplement: Supplementary file 1 [file Data_Sheet_1.zip › 4.Beta_diversity/NMDS/jaccard_NMDS_cluster.png]

# NMDS jaccard

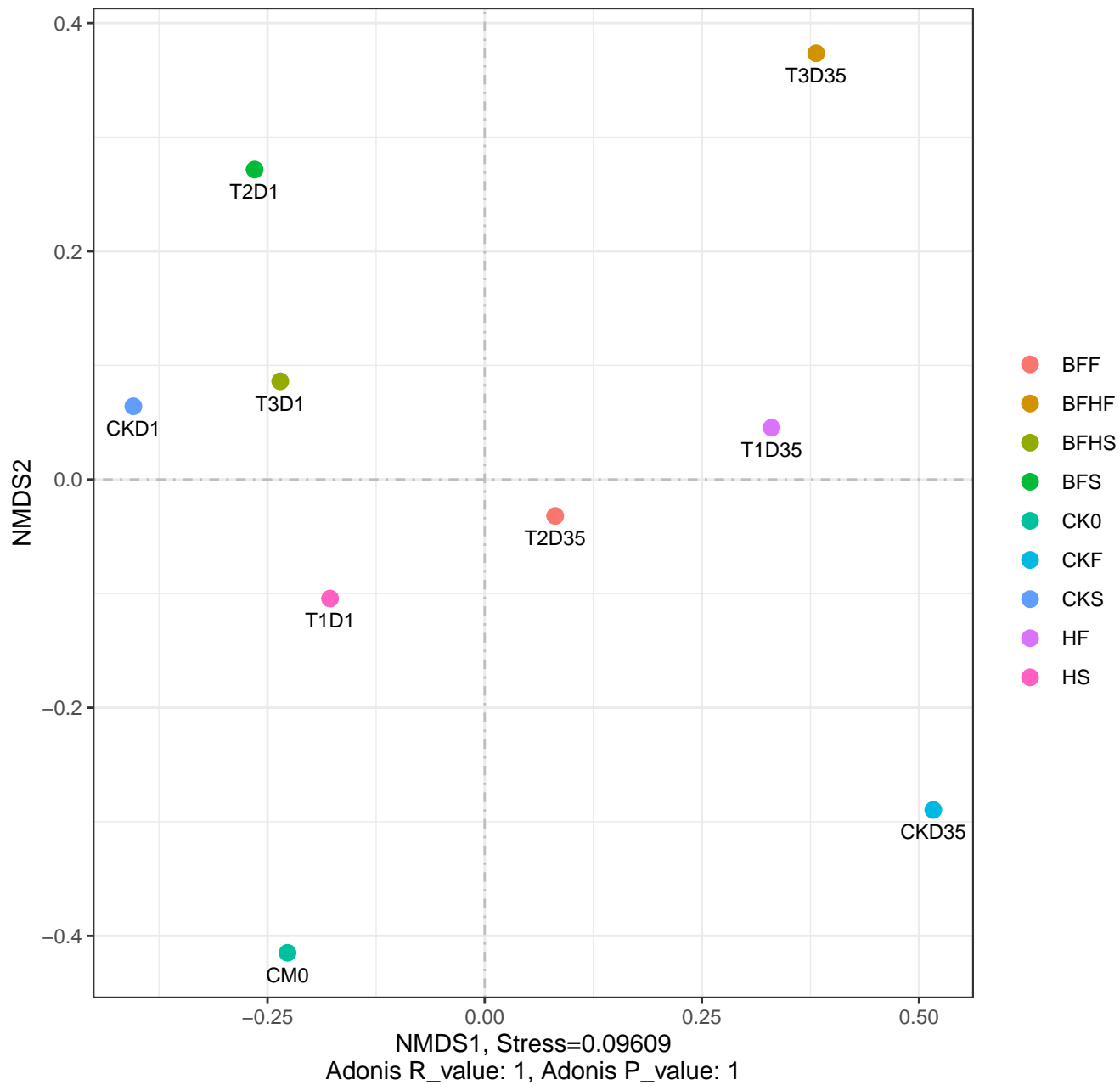

Supplement: Supplementary file 1 [file Data_Sheet_1.zip › 4.Beta_diversity/NMDS/jaccard_NMDS_name.pdf]

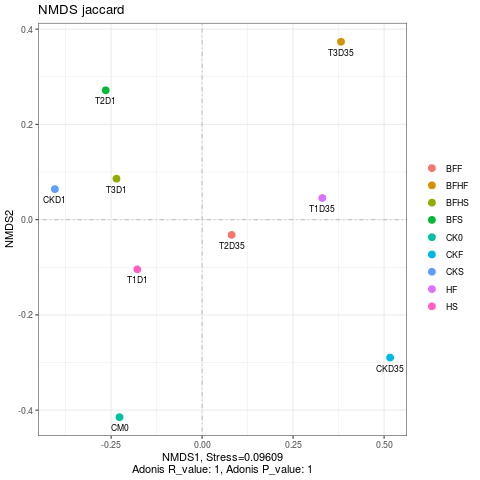

Supplement: Supplementary file 1 [file Data_Sheet_1.zip › 4.Beta_diversity/NMDS/jaccard_NMDS_name.png]

# NMDS jaccard

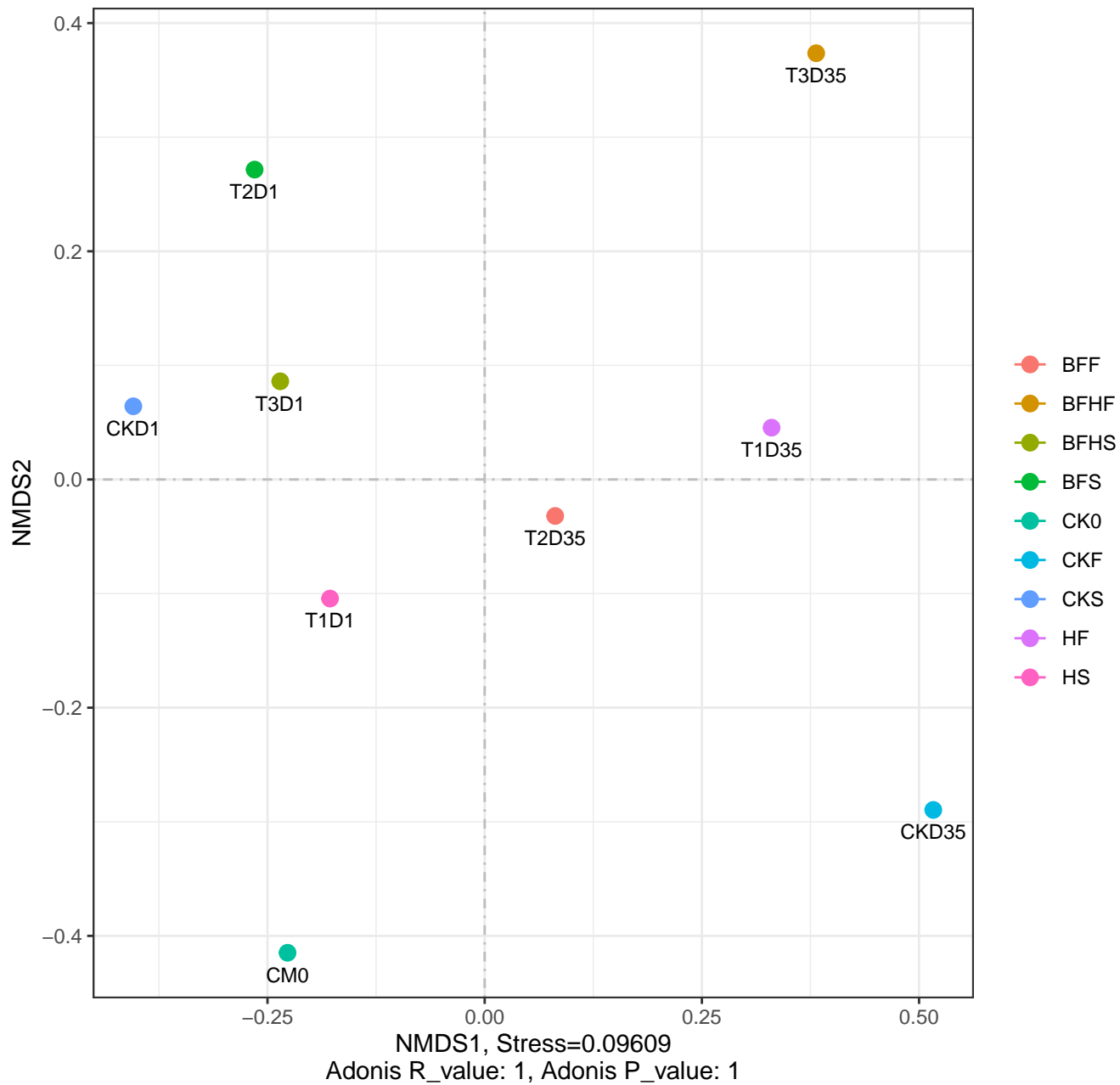

Supplement: Supplementary file 1 [file Data_Sheet_1.zip › 4.Beta_diversity/NMDS/jaccard_NMDS_name_cluster.pdf]

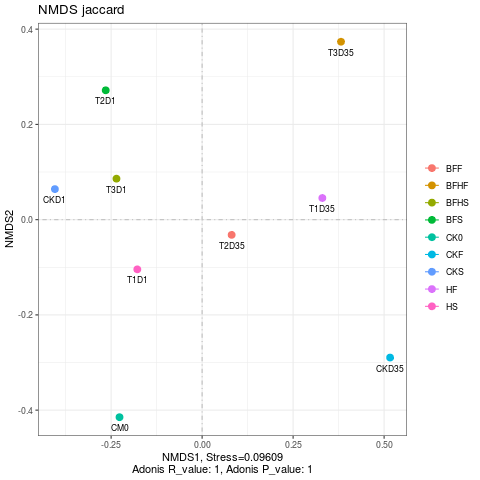

Supplement: Supplementary file 1 [file Data_Sheet_1.zip › 4.Beta_diversity/NMDS/jaccard_NMDS_name_cluster.png]

# NMDS unweighted\_unifrac

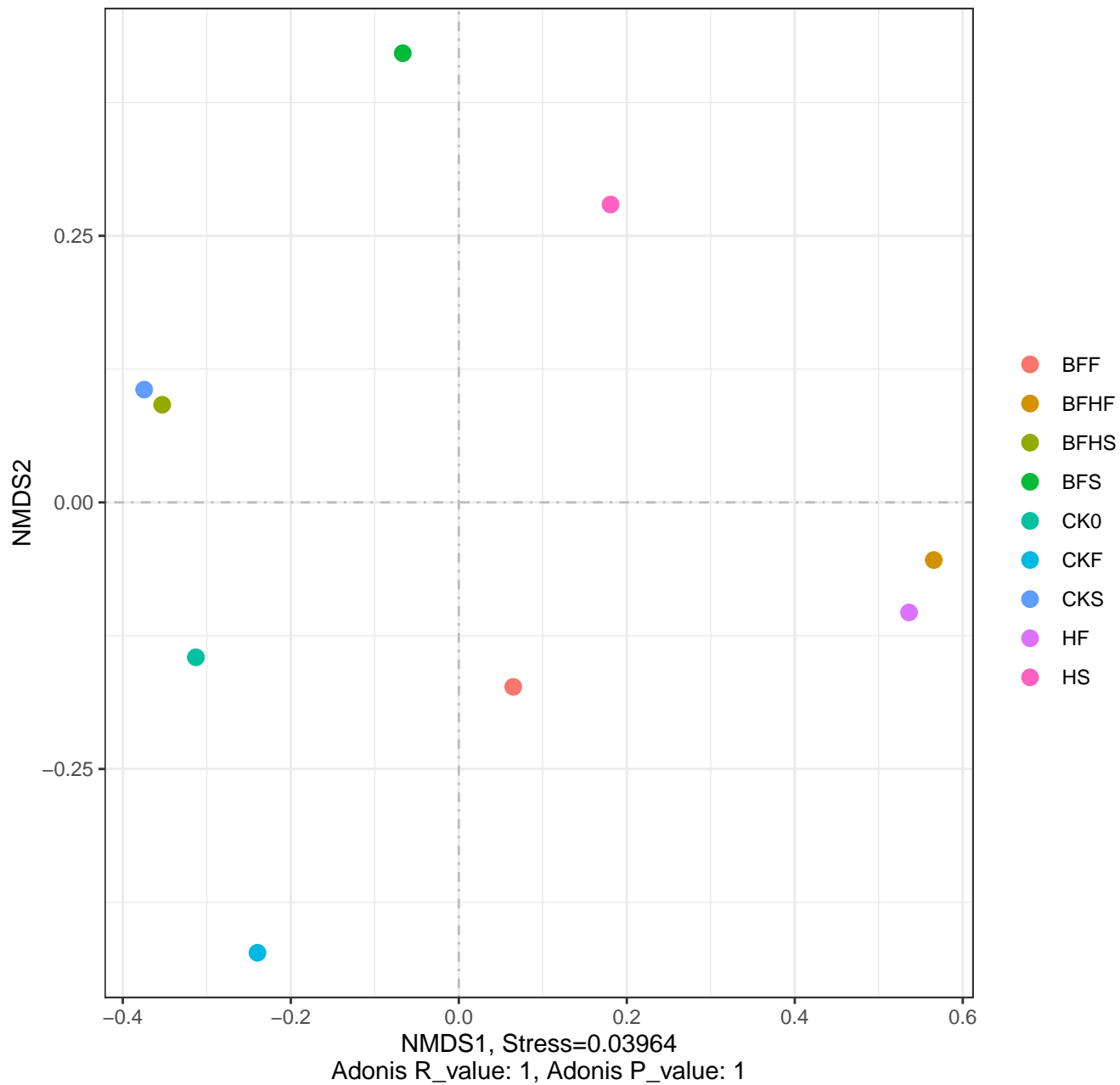

Supplement: Supplementary file 1 [file Data_Sheet_1.zip › 4.Beta_diversity/NMDS/unweighted_unifrac_NMDS.pdf]

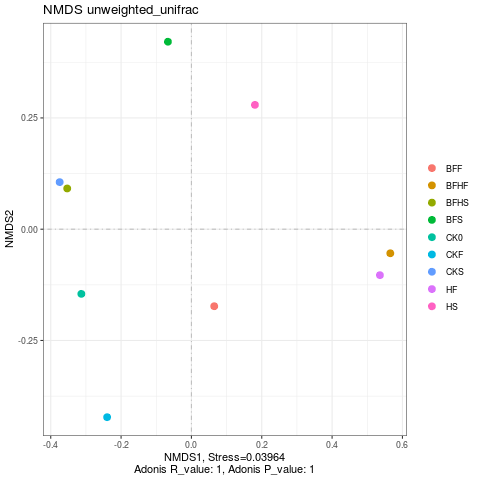

Supplement: Supplementary file 1 [file Data_Sheet_1.zip › 4.Beta_diversity/NMDS/unweighted_unifrac_NMDS.png]

# NMDS unweighted\_unifrac

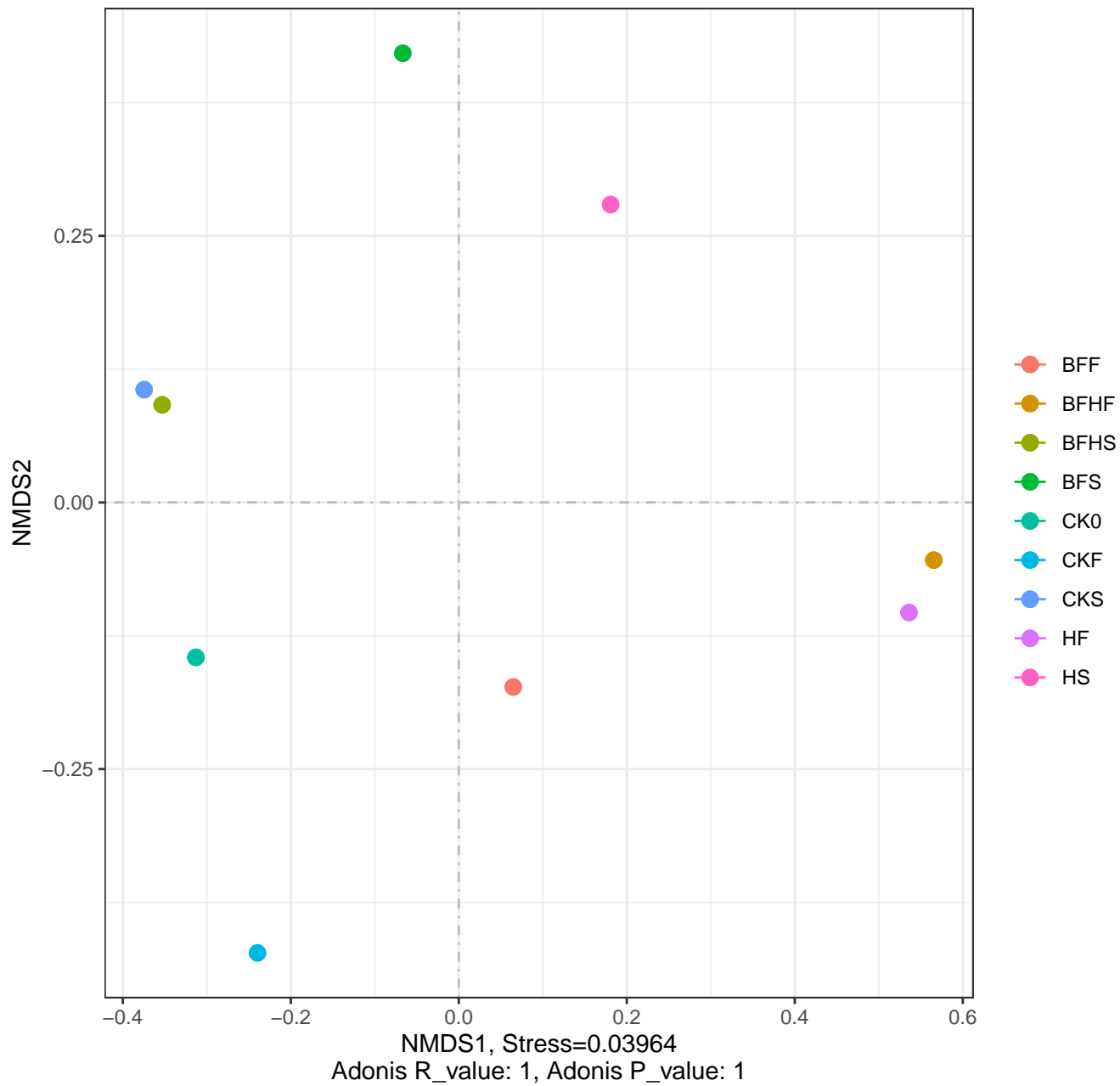

Supplement: Supplementary file 1 [file Data_Sheet_1.zip › 4.Beta_diversity/NMDS/unweighted_unifrac_NMDS_cluster.pdf]

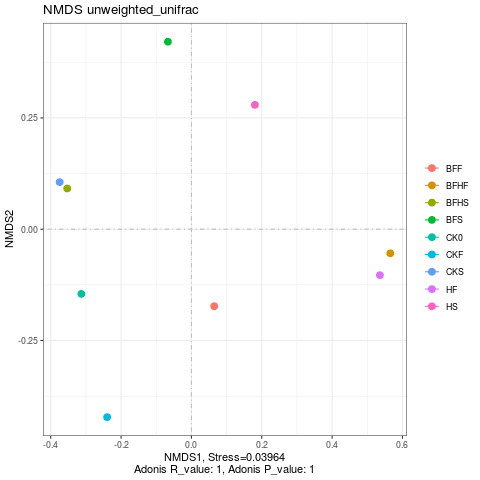

Supplement: Supplementary file 1 [file Data_Sheet_1.zip › 4.Beta_diversity/NMDS/unweighted_unifrac_NMDS_cluster.png]

# NMDS unweighted\_unifrac

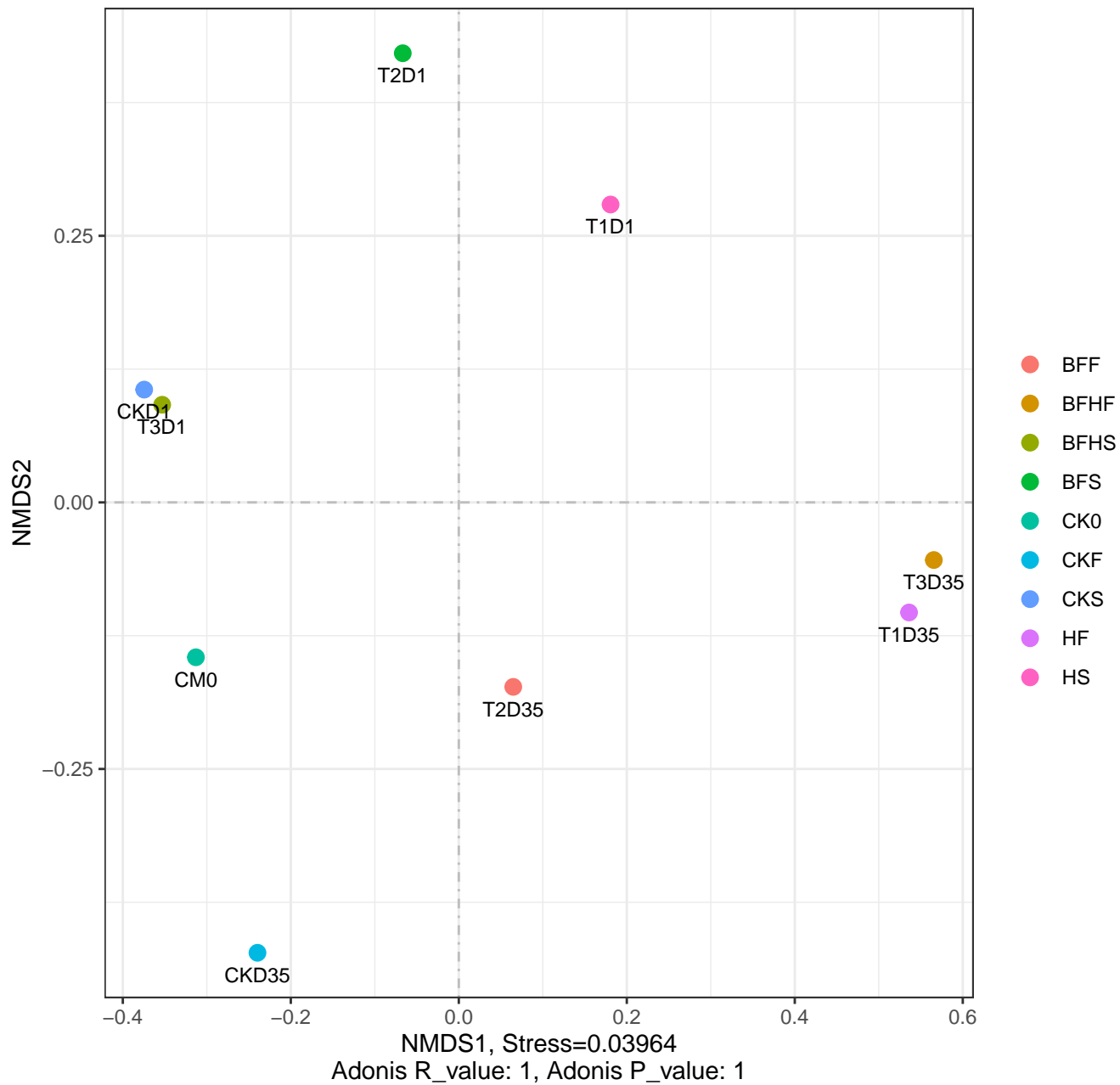

Supplement: Supplementary file 1 [file Data_Sheet_1.zip › 4.Beta_diversity/NMDS/unweighted_unifrac_NMDS_name.pdf]

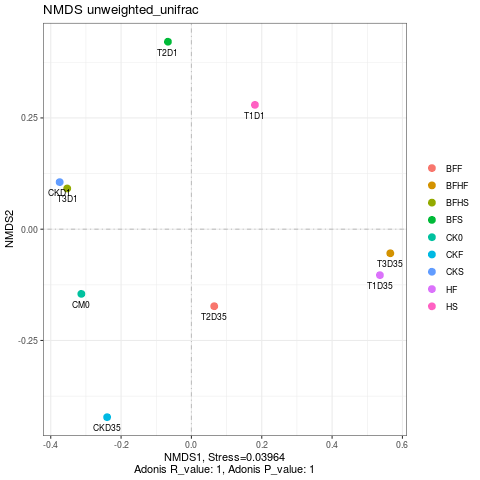

Supplement: Supplementary file 1 [file Data_Sheet_1.zip › 4.Beta_diversity/NMDS/unweighted_unifrac_NMDS_name.png]

# NMDS unweighted\_unifrac

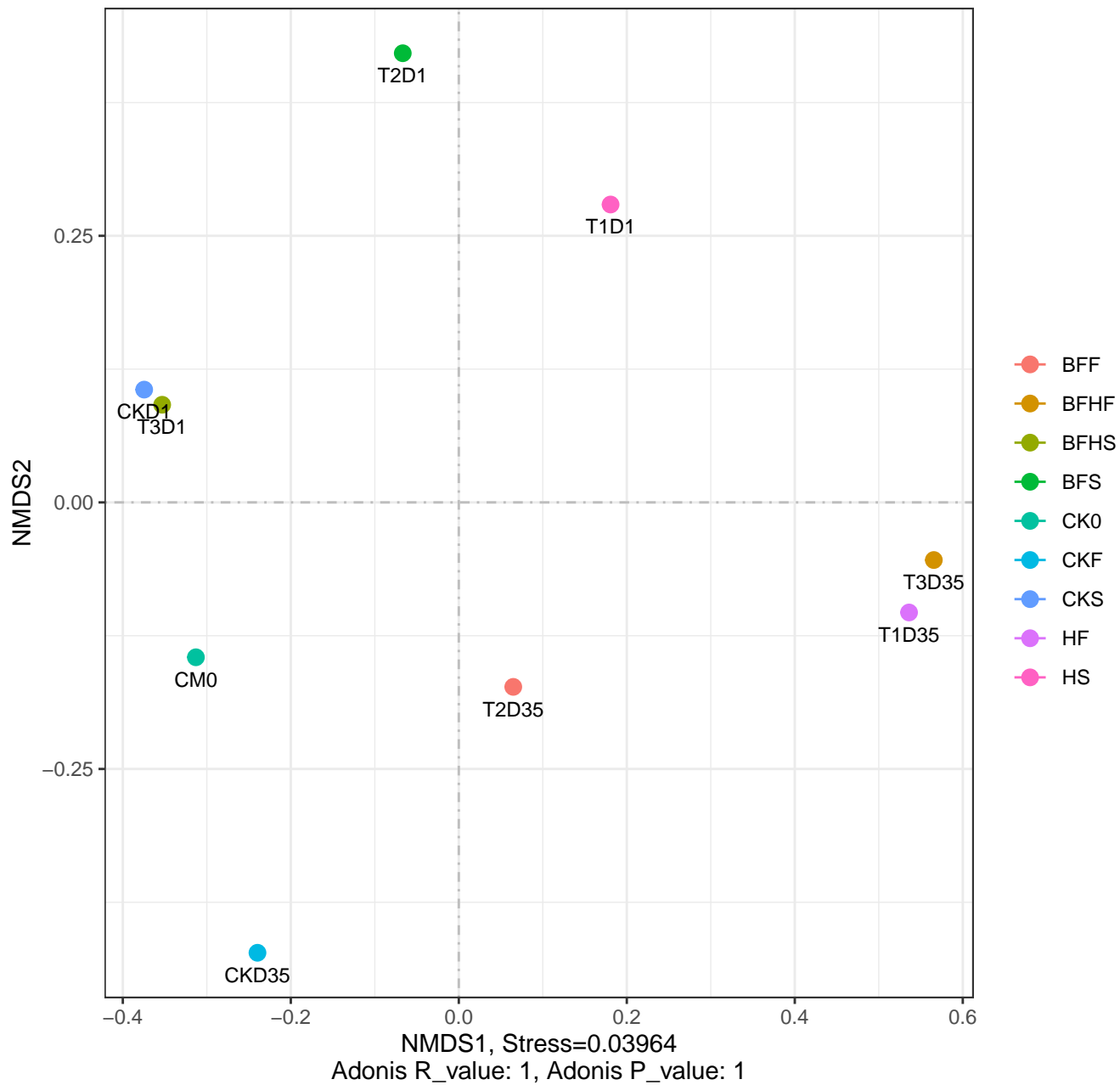

Supplement: Supplementary file 1 [file Data_Sheet_1.zip › 4.Beta_diversity/NMDS/unweighted_unifrac_NMDS_name_cluster.pdf]

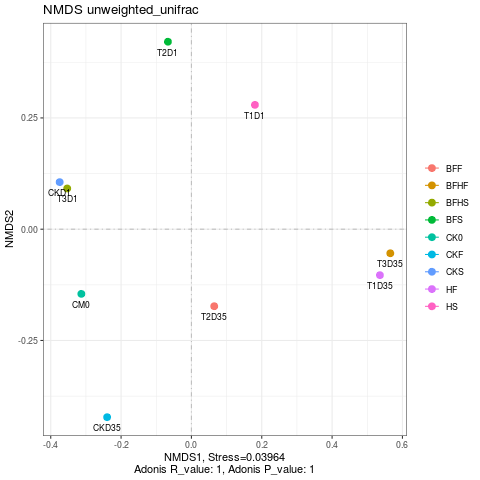

Supplement: Supplementary file 1 [file Data_Sheet_1.zip › 4.Beta_diversity/NMDS/unweighted_unifrac_NMDS_name_cluster.png]

# NMDS weighted\_unifrac

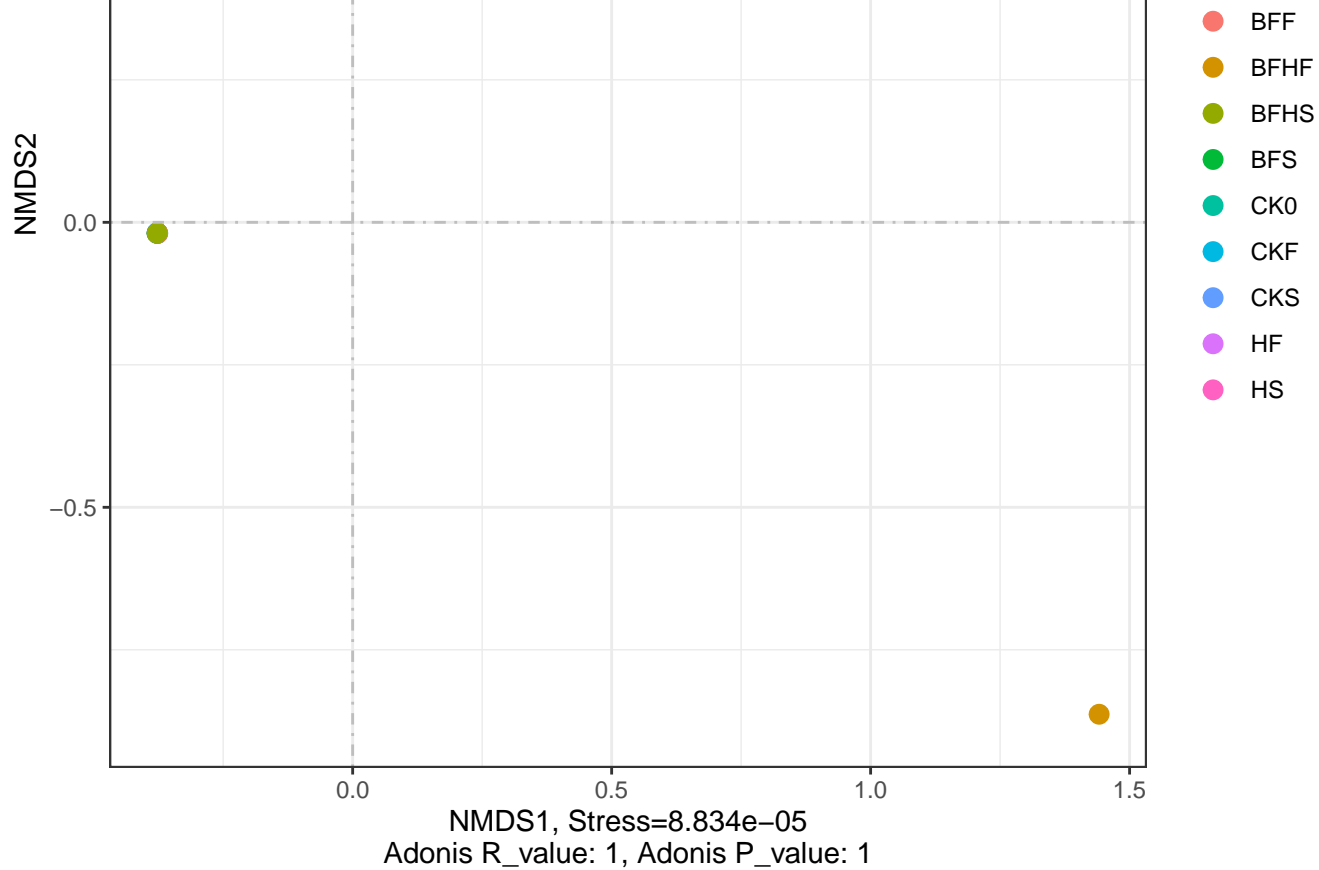

Supplement: Supplementary file 1 [file Data_Sheet_1.zip › 4.Beta_diversity/NMDS/weighted_unifrac_NMDS.pdf]

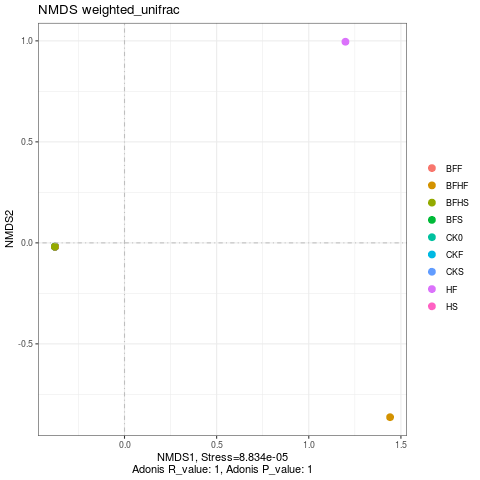

Supplement: Supplementary file 1 [file Data_Sheet_1.zip › 4.Beta_diversity/NMDS/weighted_unifrac_NMDS.png]

# NMDS weighted\_unifrac

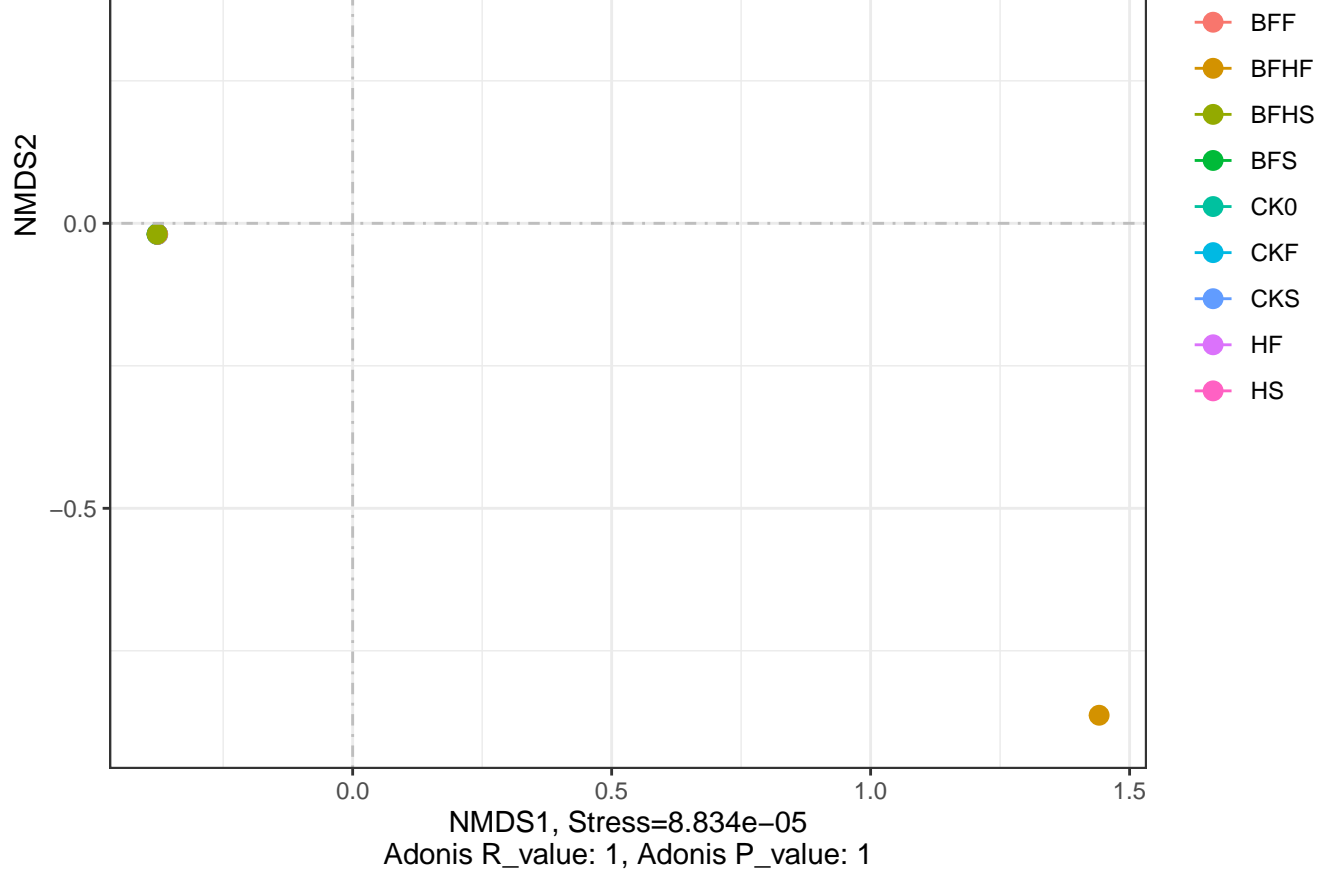

Supplement: Supplementary file 1 [file Data_Sheet_1.zip › 4.Beta_diversity/NMDS/weighted_unifrac_NMDS_cluster.pdf]

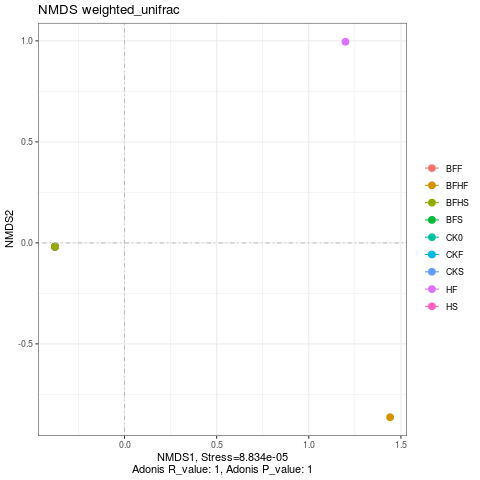

Supplement: Supplementary file 1 [file Data_Sheet_1.zip › 4.Beta_diversity/NMDS/weighted_unifrac_NMDS_cluster.png]

# NMDS weighted\_unifrac

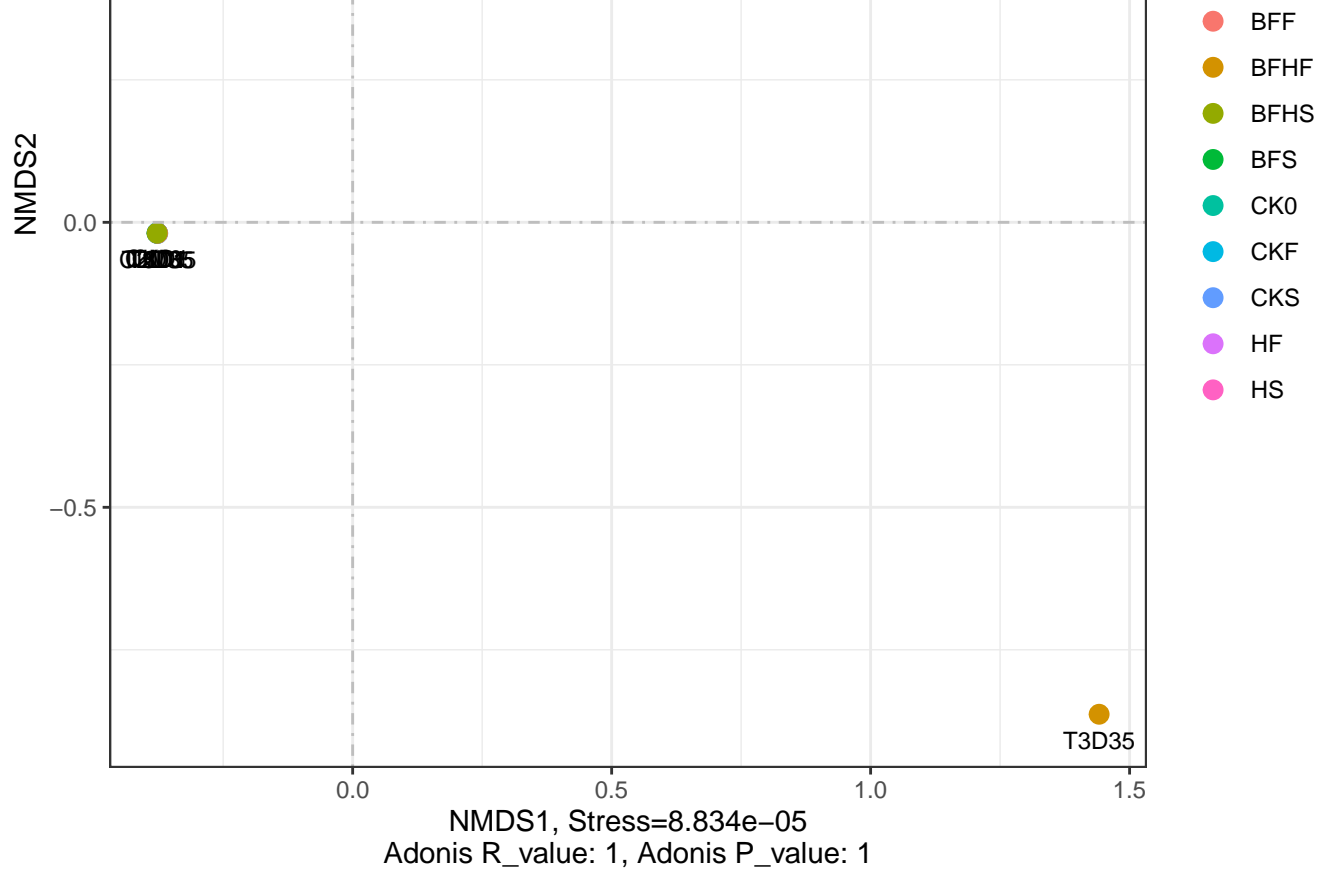

Supplement: Supplementary file 1 [file Data_Sheet_1.zip › 4.Beta_diversity/NMDS/weighted_unifrac_NMDS_name.pdf]

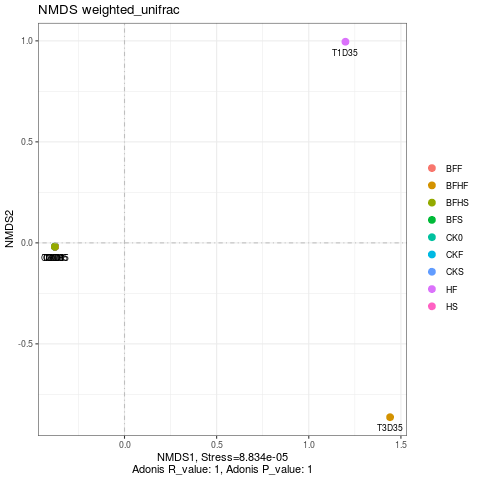

Supplement: Supplementary file 1 [file Data_Sheet_1.zip › 4.Beta_diversity/NMDS/weighted_unifrac_NMDS_name.png]

# NMDS weighted\_unifrac

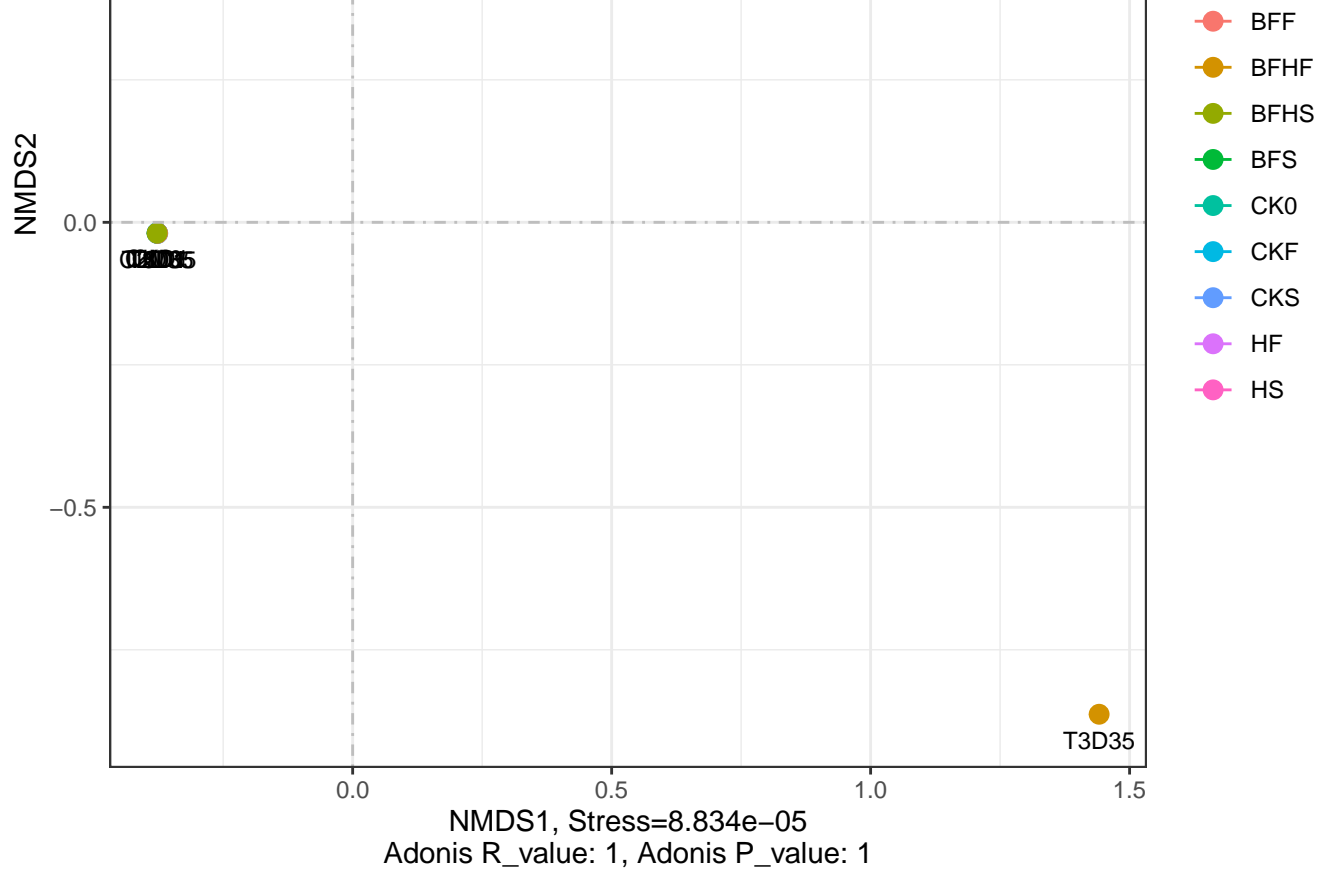

Supplement: Supplementary file 1 [file Data_Sheet_1.zip › 4.Beta_diversity/NMDS/weighted_unifrac_NMDS_name_cluster.pdf]

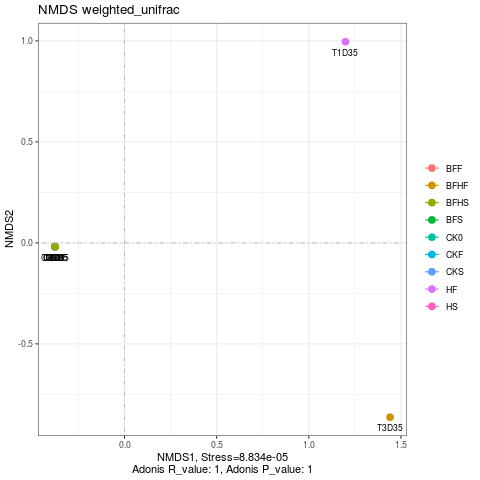

Supplement: Supplementary file 1 [file Data_Sheet_1.zip › 4.Beta_diversity/NMDS/weighted_unifrac_NMDS_name_cluster.png]

# aitchison\_PCoA1

Kruskal-Wallis,  $p = 0.4335$

group

|     |     |      |     |      |
|-----|-----|------|-----|------|
| CK0 | HS  | BFHS | HF  | BFHF |
| CKS | BFS | CKF  | BFF |      |

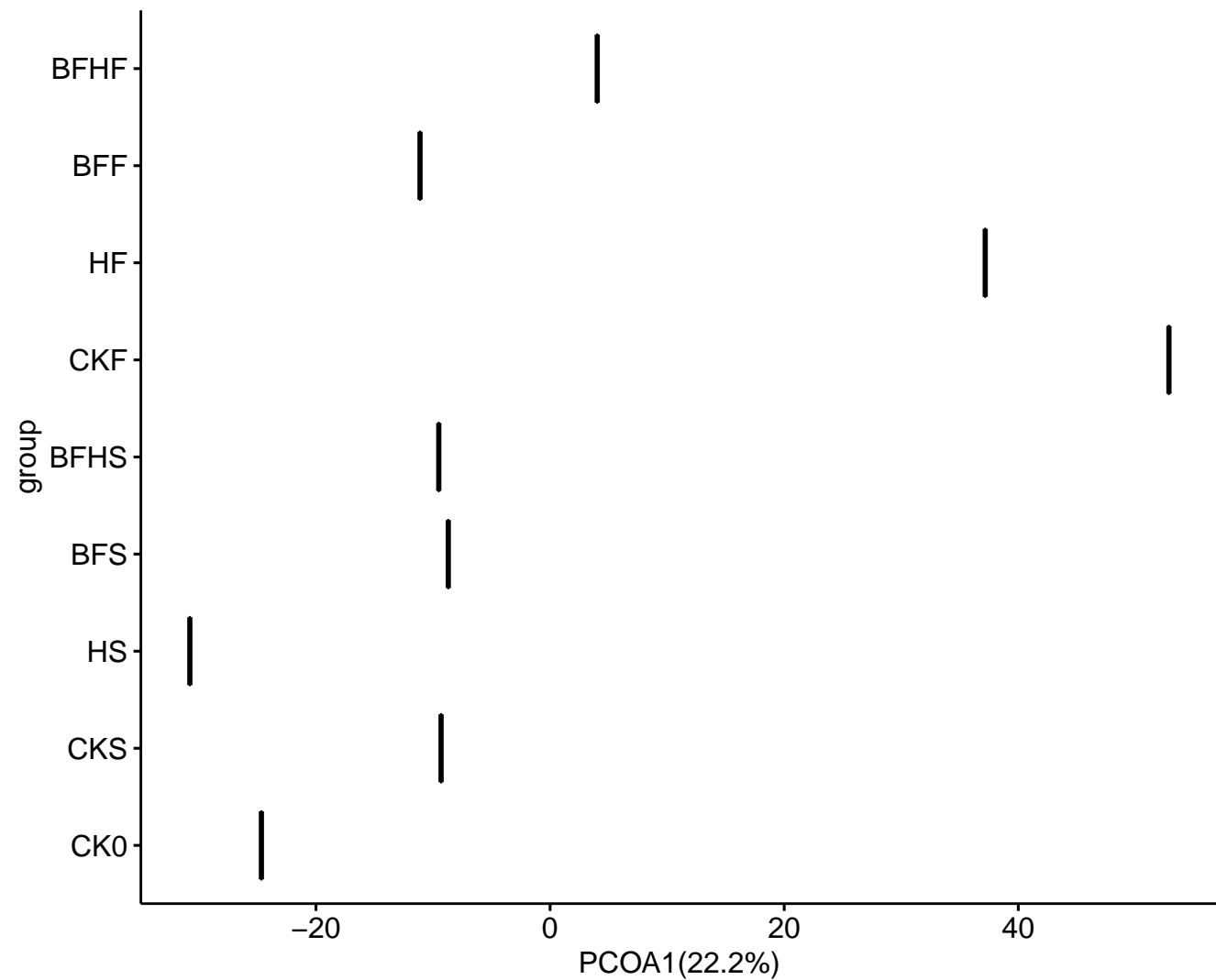

Supplement: Supplementary file 1 [file Data_Sheet_1.zip › 4.Beta_diversity/PCoA/aitchison.PCoA1.boxplot.pdf]

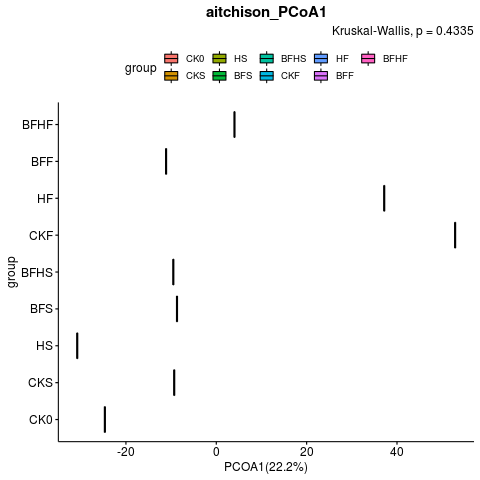

Supplement: Supplementary file 1 [file Data_Sheet_1.zip › 4.Beta_diversity/PCoA/aitchison.PCoA1.boxplot.png]

# aitchison\_PCoA2

Kruskal-Wallis,  $p = 0.4335$

group

|     |     |      |     |      |
|-----|-----|------|-----|------|
| CK0 | HS  | BFHS | HF  | BFHF |
| CKS | BFS | CKF  | BFF |      |

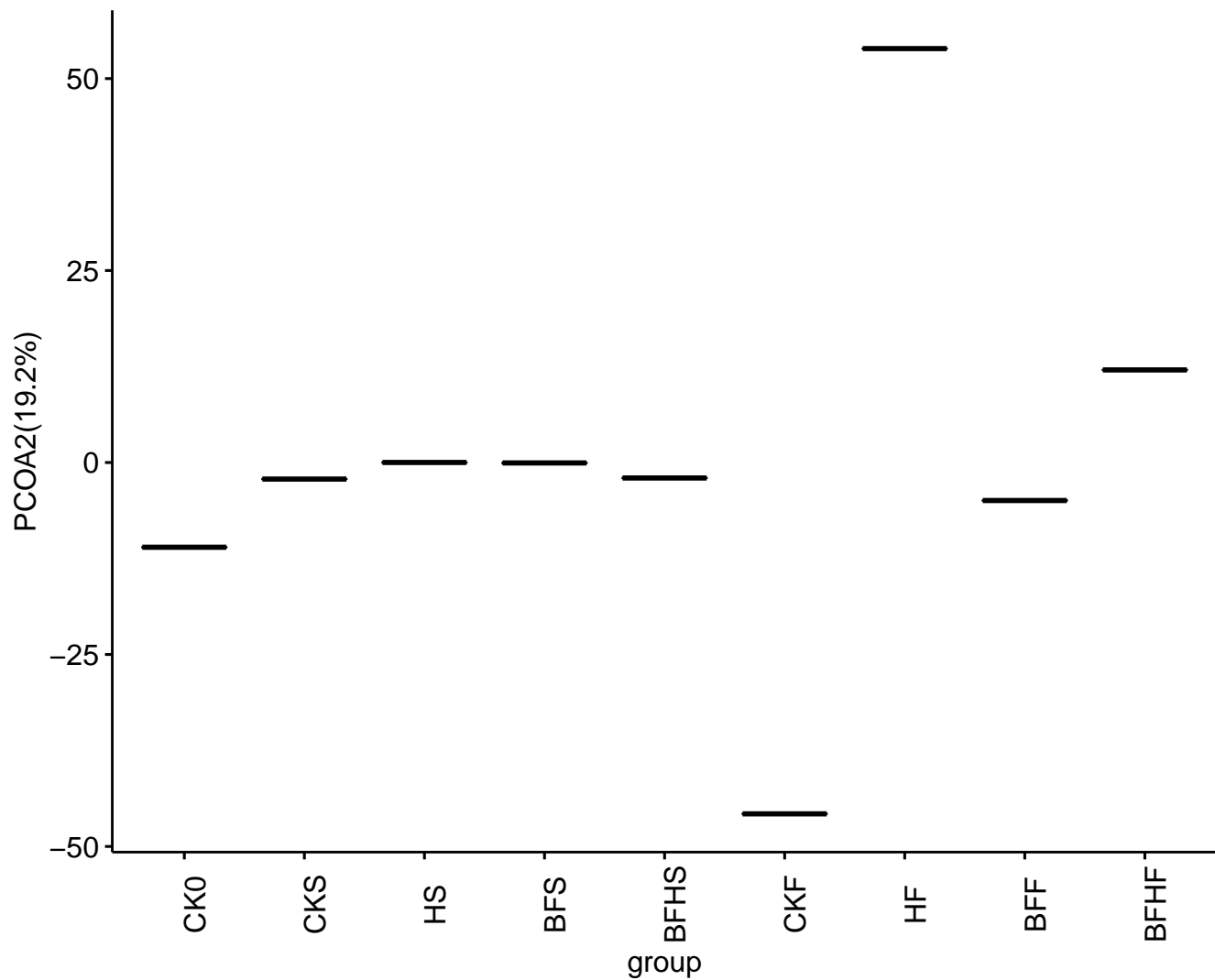

Supplement: Supplementary file 1 [file Data_Sheet_1.zip › 4.Beta_diversity/PCoA/aitchison.PCoA2.boxplot.pdf]

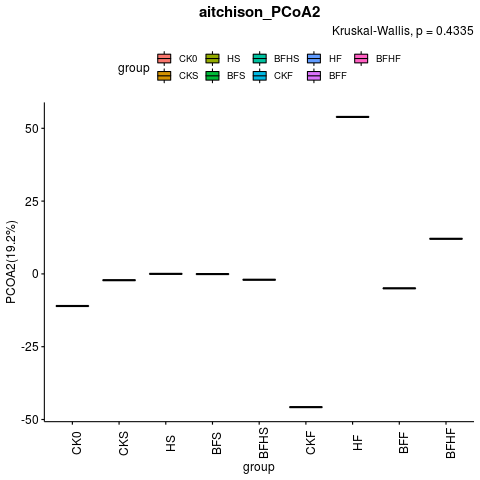

Supplement: Supplementary file 1 [file Data_Sheet_1.zip › 4.Beta_diversity/PCoA/aitchison.PCoA2.boxplot.png]

PCoA aitchison

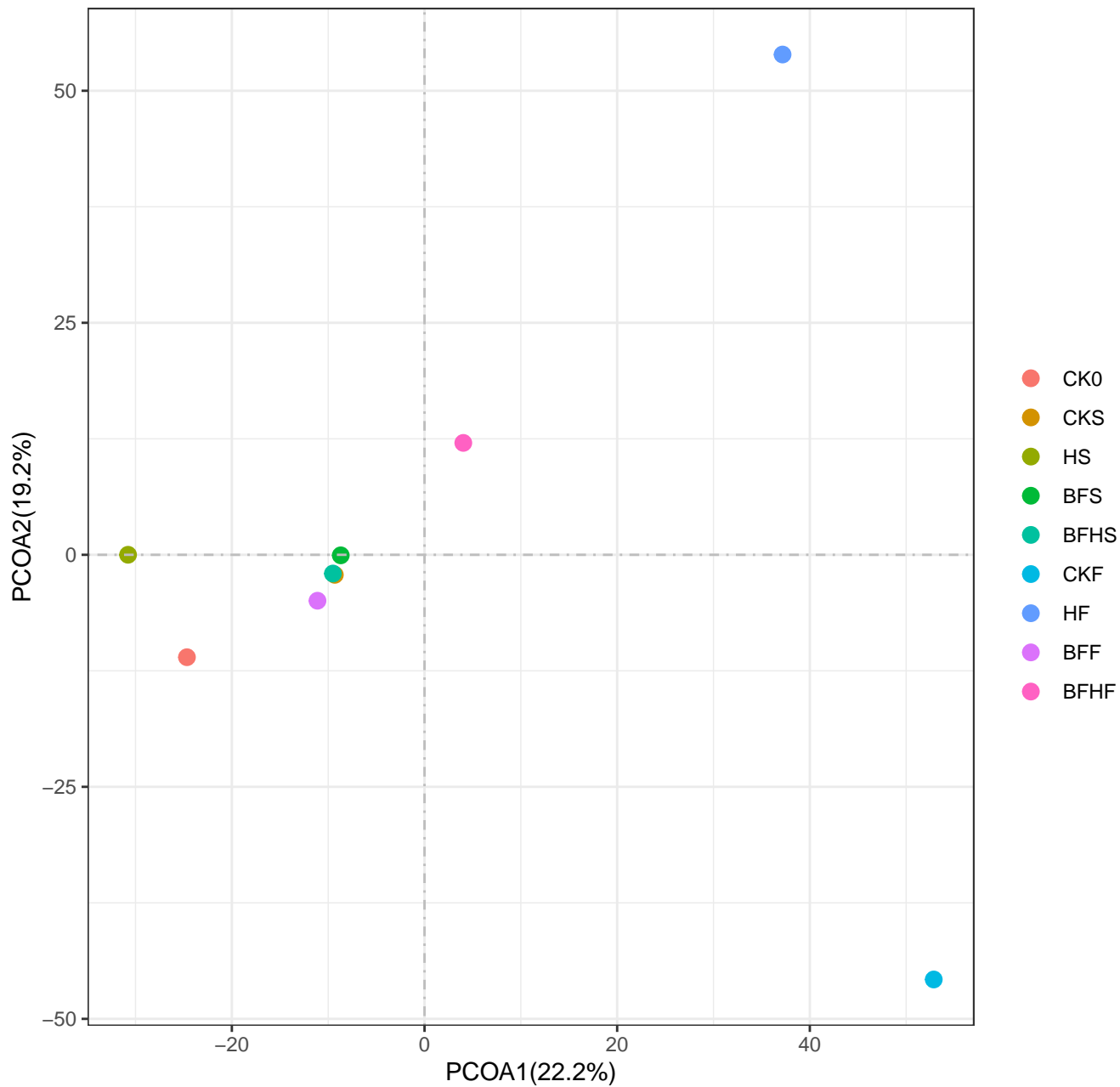

Supplement: Supplementary file 1 [file Data_Sheet_1.zip › 4.Beta_diversity/PCoA/aitchison_PCoA.pdf]

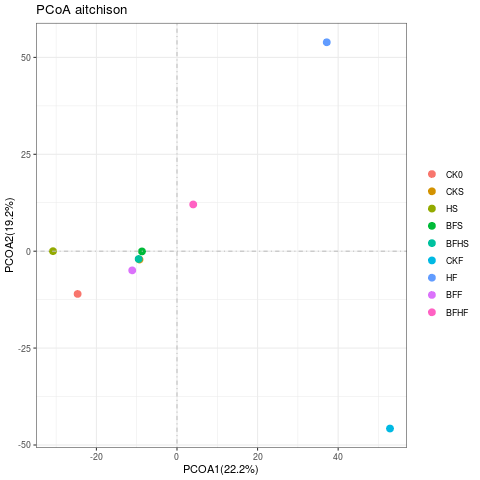

Supplement: Supplementary file 1 [file Data_Sheet_1.zip › 4.Beta_diversity/PCoA/aitchison_PCoA.png]

PCoA aitchison

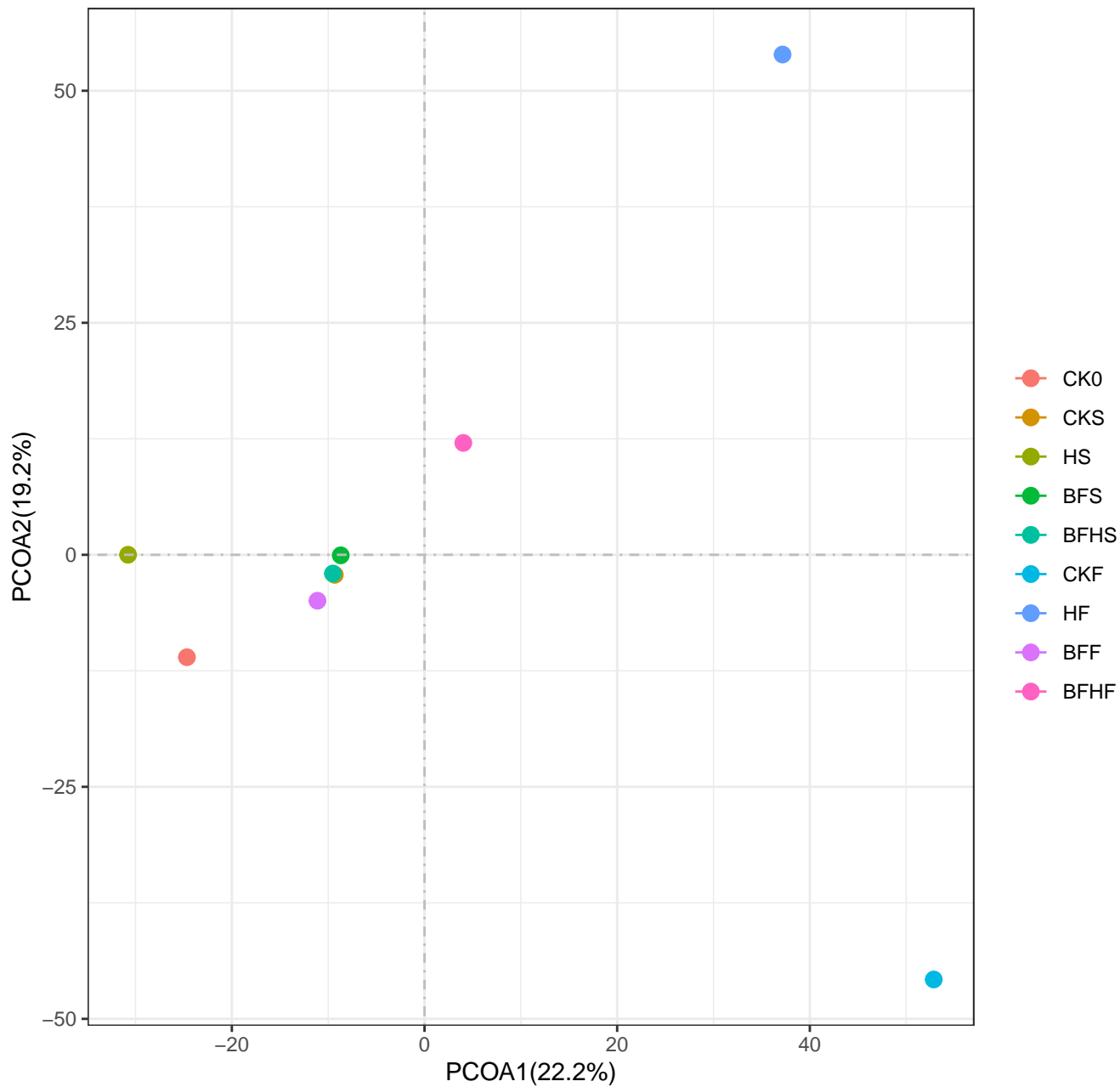

Supplement: Supplementary file 1 [file Data_Sheet_1.zip › 4.Beta_diversity/PCoA/aitchison_PCoA_cluster.pdf]

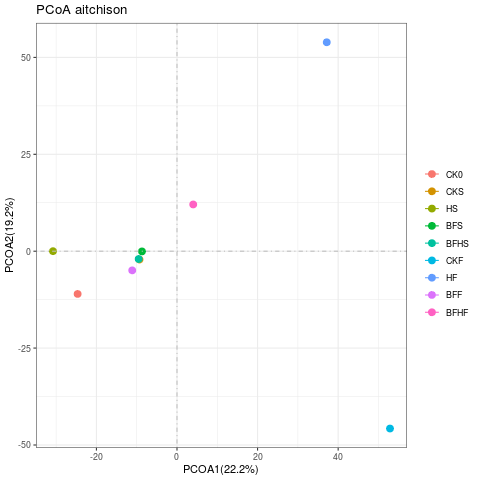

Supplement: Supplementary file 1 [file Data_Sheet_1.zip › 4.Beta_diversity/PCoA/aitchison_PCoA_cluster.png]

PCoA aitchison

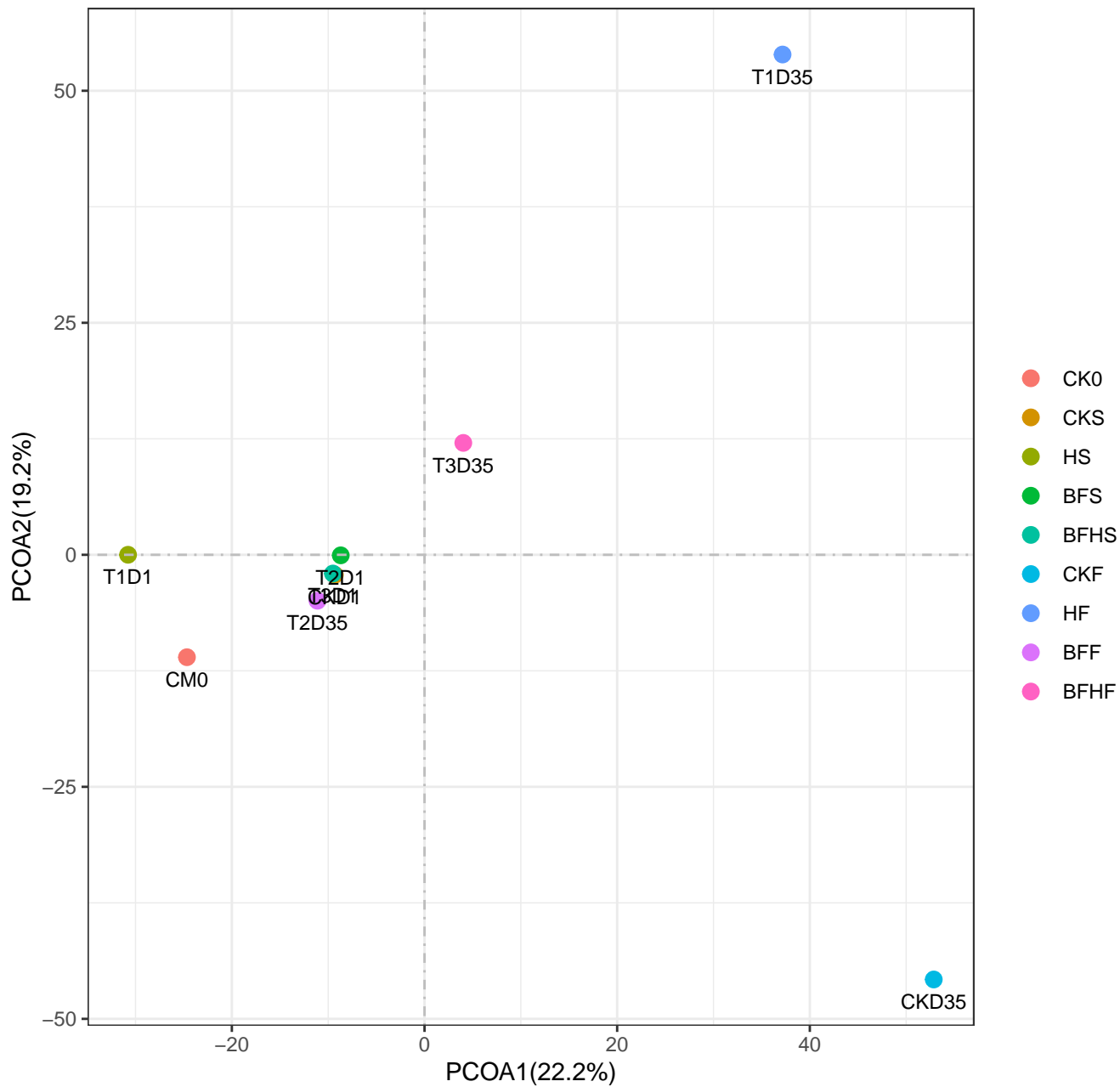

Supplement: Supplementary file 1 [file Data_Sheet_1.zip › 4.Beta_diversity/PCoA/aitchison_PCoA_name.pdf]

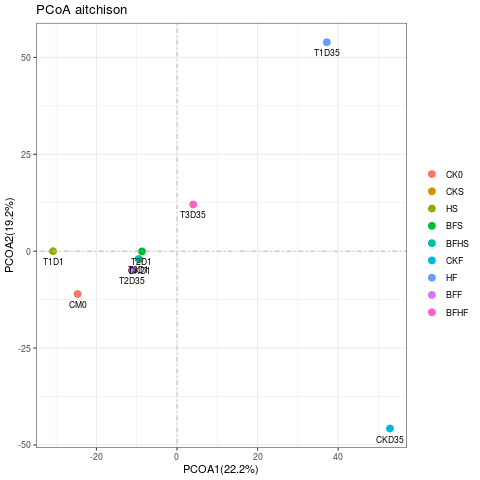

Supplement: Supplementary file 1 [file Data_Sheet_1.zip › 4.Beta_diversity/PCoA/aitchison_PCoA_name.png]

PCoA aitchison

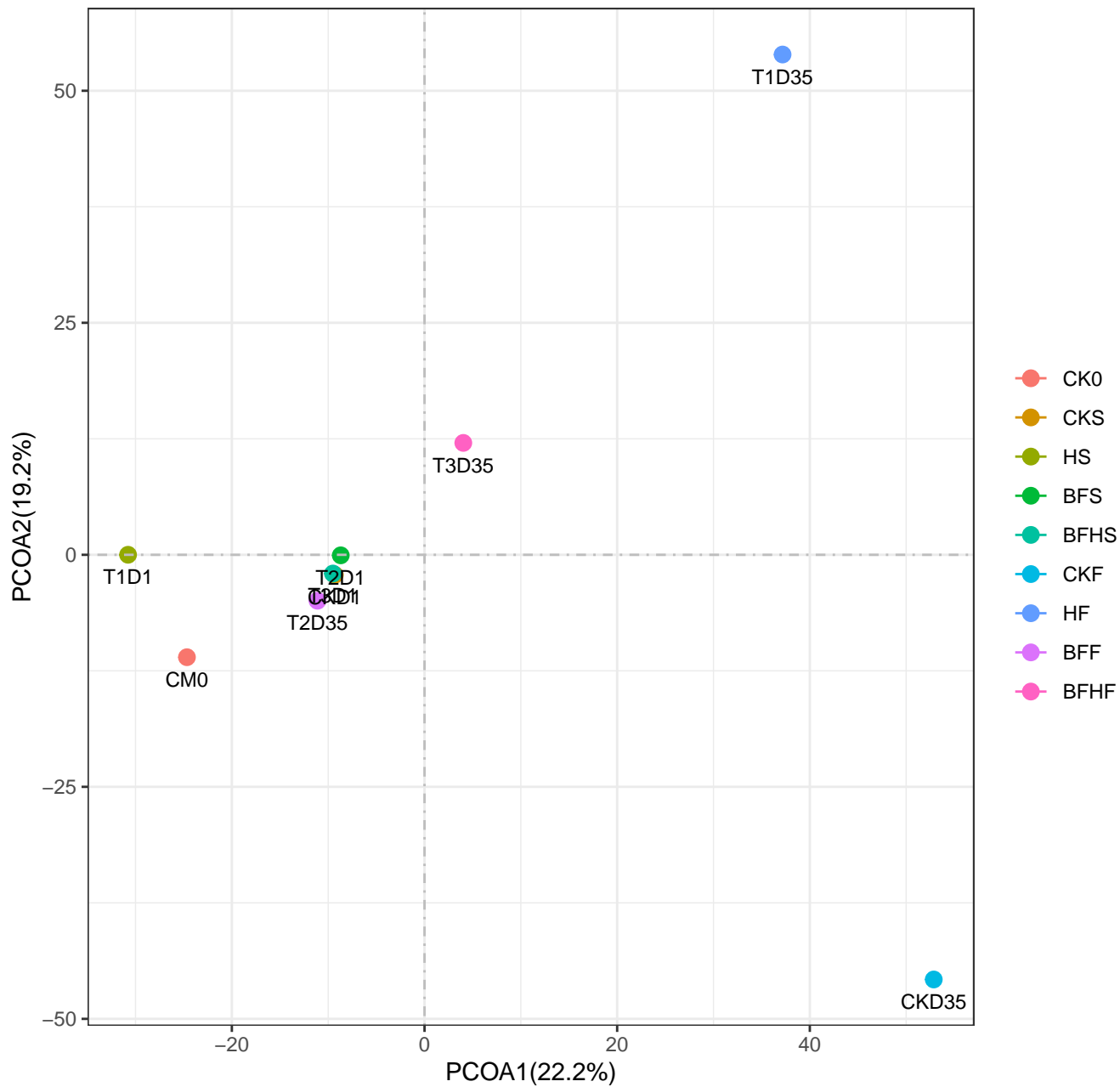

Supplement: Supplementary file 1 [file Data_Sheet_1.zip › 4.Beta_diversity/PCoA/aitchison_PCoA_name_cluster.pdf]

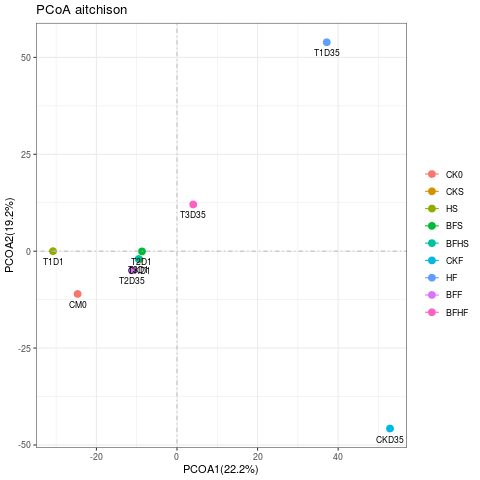

Supplement: Supplementary file 1 [file Data_Sheet_1.zip › 4.Beta_diversity/PCoA/aitchison_PCoA_name_cluster.png]

# bray\_curtis\_PCoA1

Kruskal-Wallis,  $p = 0.4335$

group

|     |     |      |     |      |
|-----|-----|------|-----|------|
| CK0 | HS  | BFHS | HF  | BFHF |
| CKS | BFS | CKF  | BFF |      |

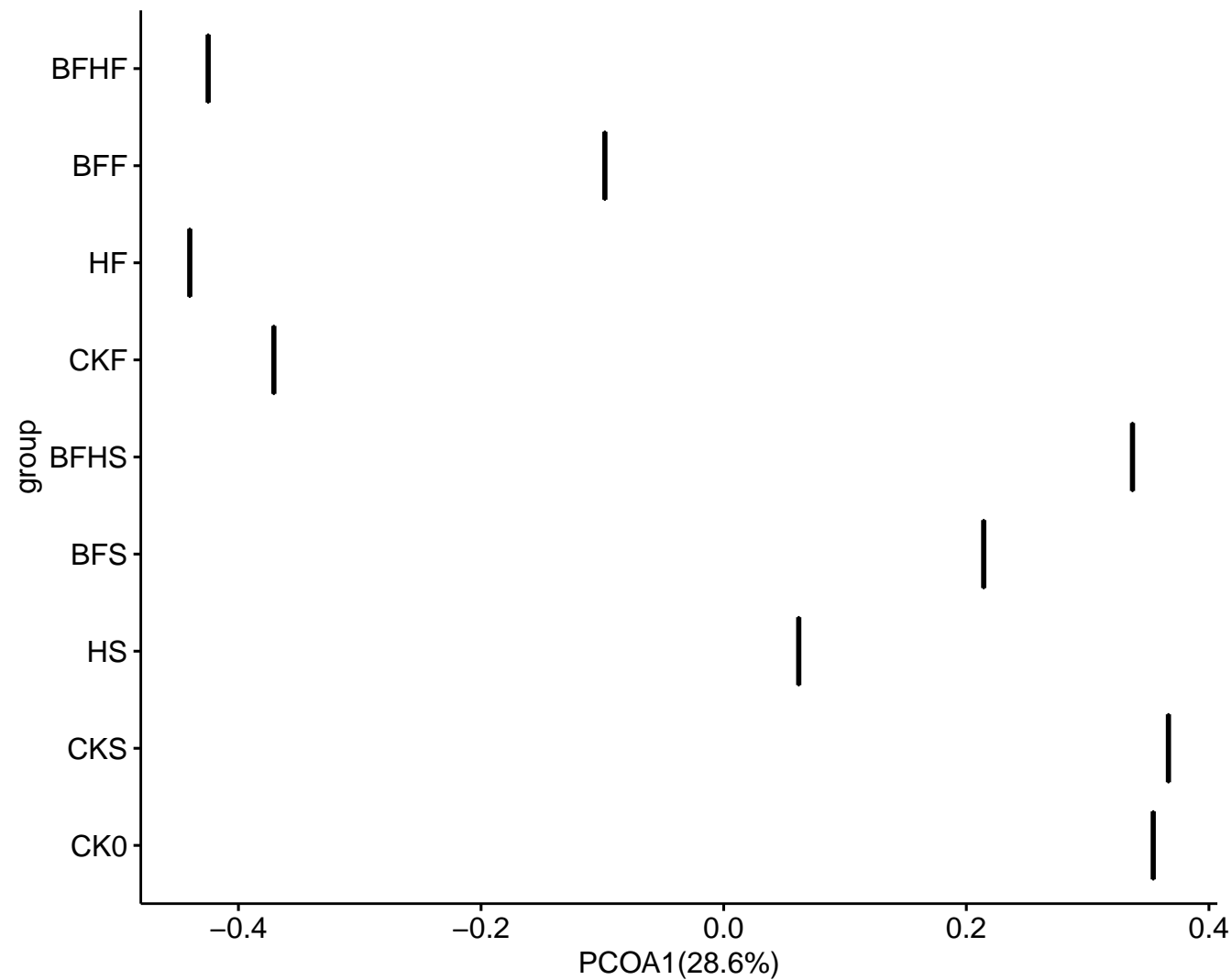

Supplement: Supplementary file 1 [file Data_Sheet_1.zip › 4.Beta_diversity/PCoA/bray_curtis.PCoA1.boxplot.pdf]

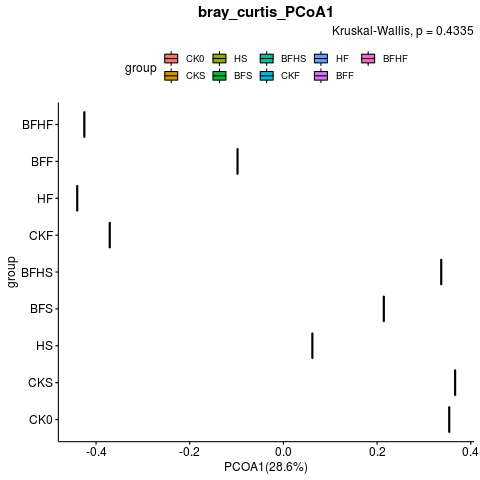

Supplement: Supplementary file 1 [file Data_Sheet_1.zip › 4.Beta_diversity/PCoA/bray_curtis.PCoA1.boxplot.png]

# bray\_curtis\_PCoA2

Kruskal-Wallis,  $p = 0.4335$

group

|     |     |      |     |      |
|-----|-----|------|-----|------|
| CK0 | HS  | BFHS | HF  | BFHF |
| CKS | BFS | CKF  | BFF |      |

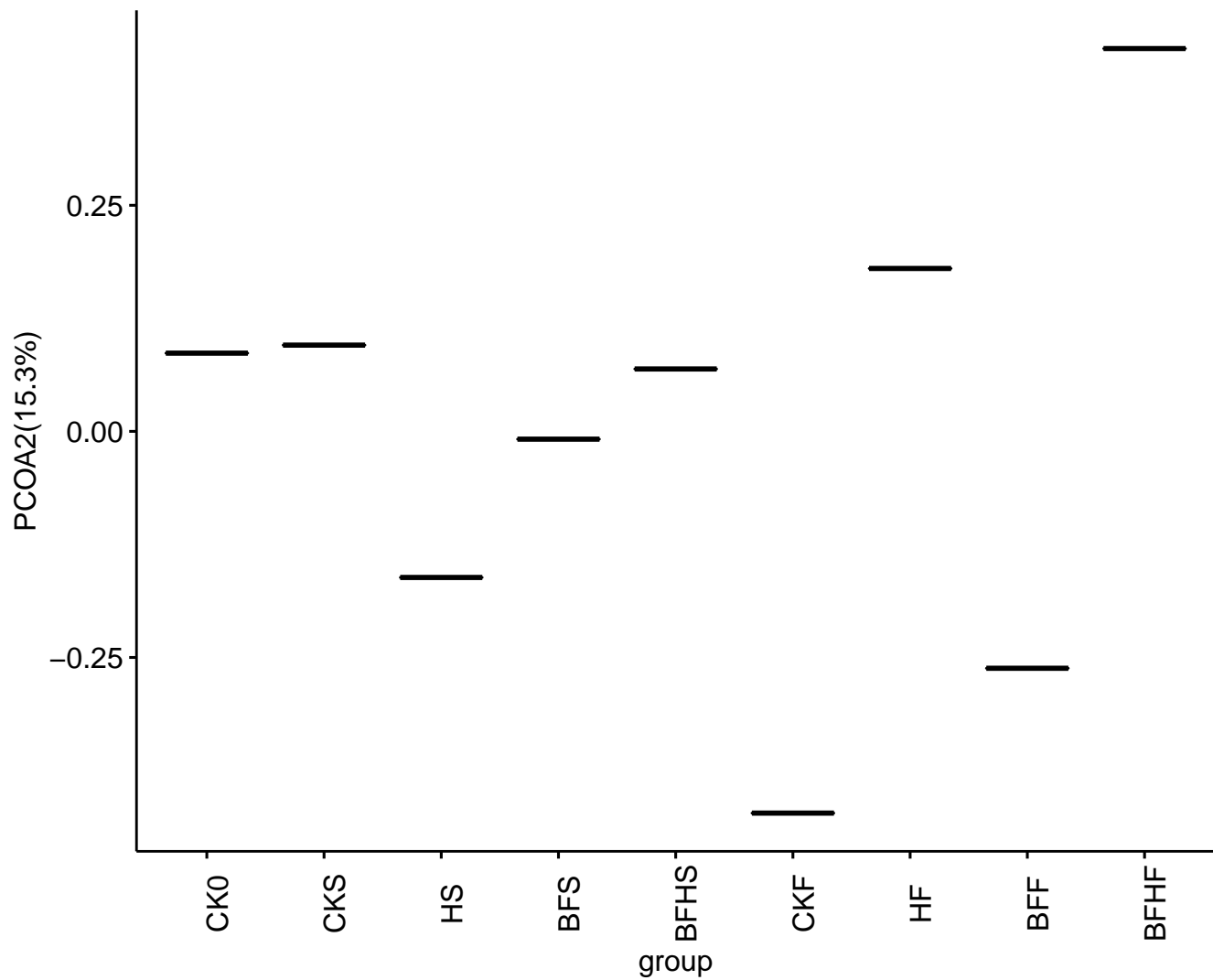

Supplement: Supplementary file 1 [file Data_Sheet_1.zip › 4.Beta_diversity/PCoA/bray_curtis.PCoA2.boxplot.pdf]

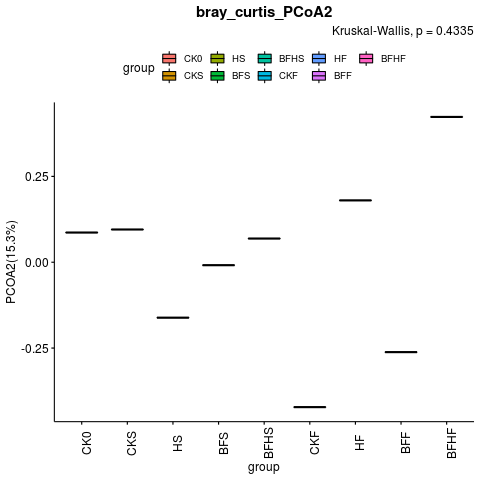

Supplement: Supplementary file 1 [file Data_Sheet_1.zip › 4.Beta_diversity/PCoA/bray_curtis.PCoA2.boxplot.png]

PCoA bray\_curtis

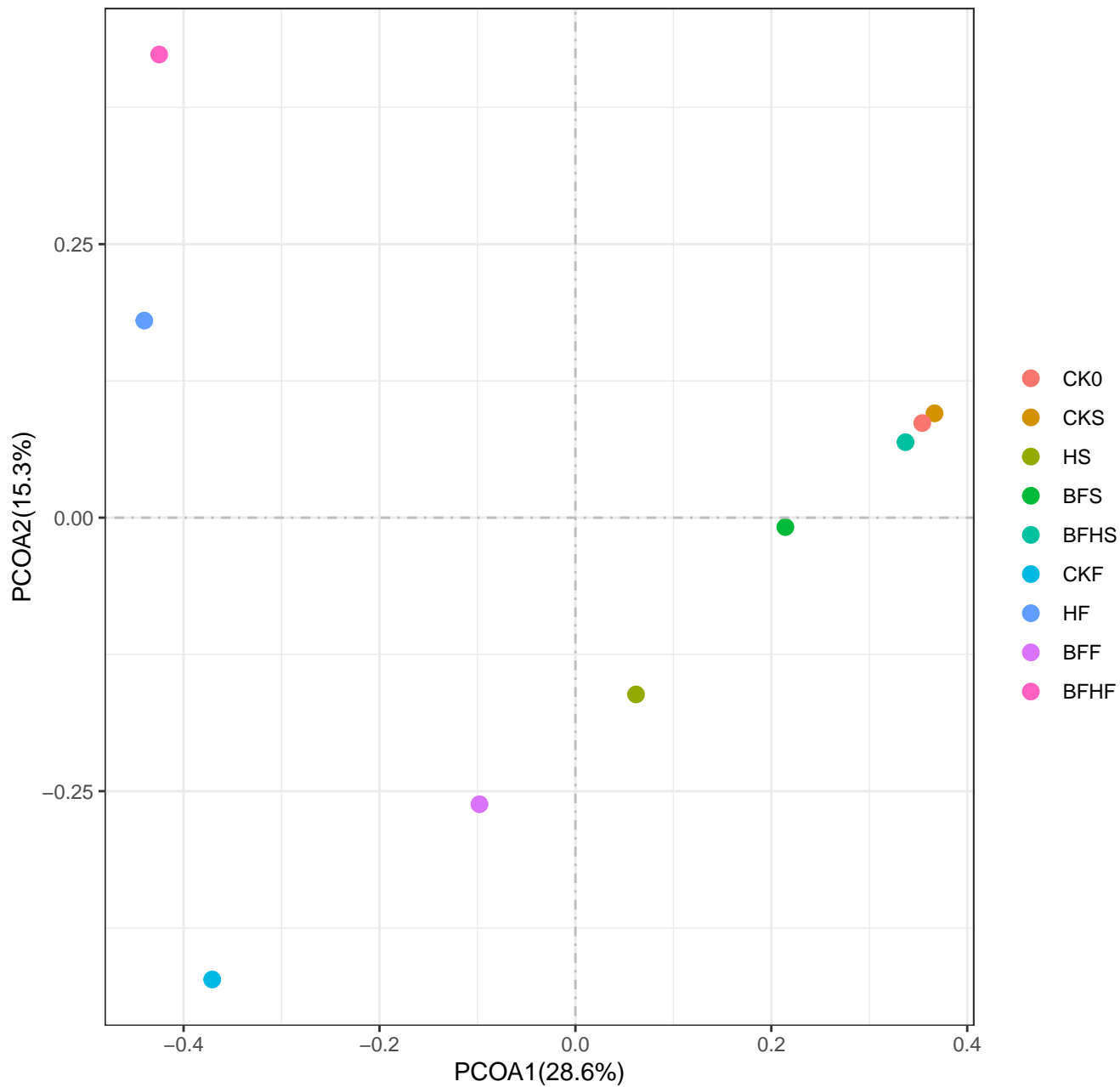

Supplement: Supplementary file 1 [file Data_Sheet_1.zip › 4.Beta_diversity/PCoA/bray_curtis_PCoA.pdf]

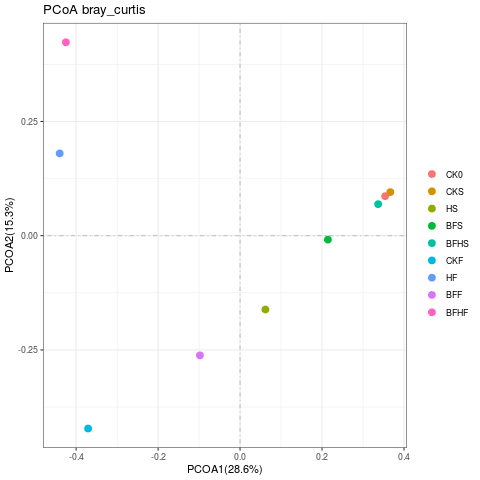

Supplement: Supplementary file 1 [file Data_Sheet_1.zip › 4.Beta_diversity/PCoA/bray_curtis_PCoA.png]

PCoA bray\_curtis

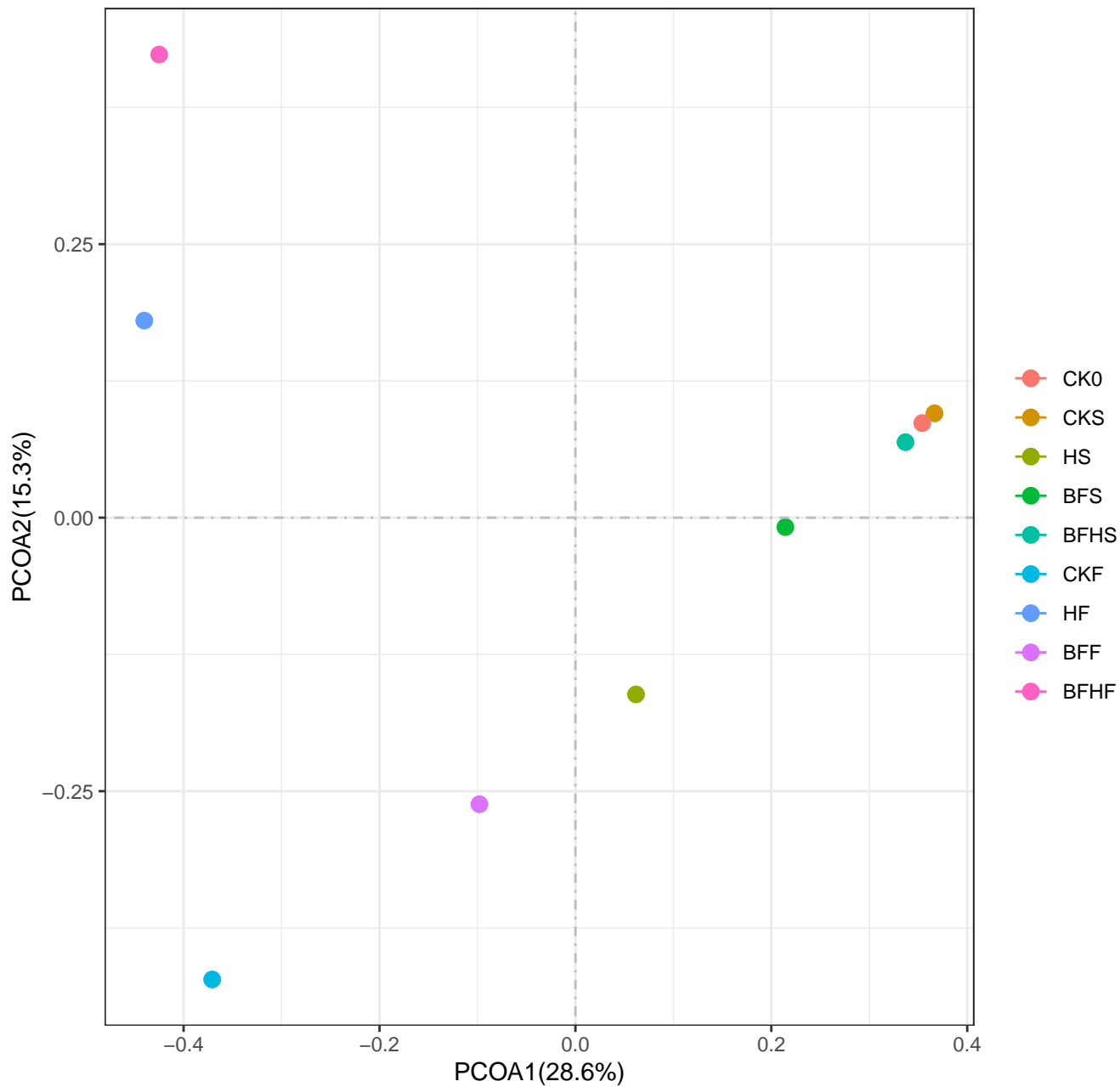

Supplement: Supplementary file 1 [file Data_Sheet_1.zip › 4.Beta_diversity/PCoA/bray_curtis_PCoA_cluster.pdf]

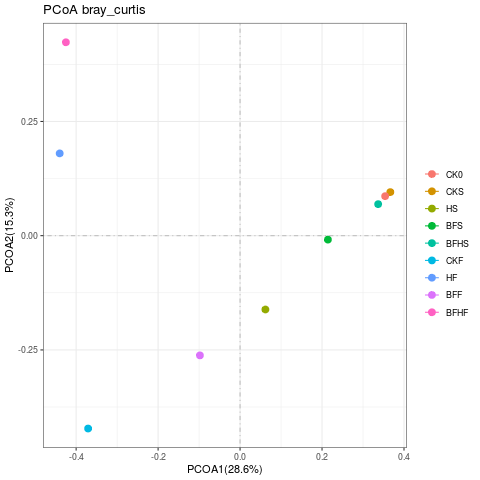

Supplement: Supplementary file 1 [file Data_Sheet_1.zip › 4.Beta_diversity/PCoA/bray_curtis_PCoA_cluster.png]

PCoA bray\_curtis

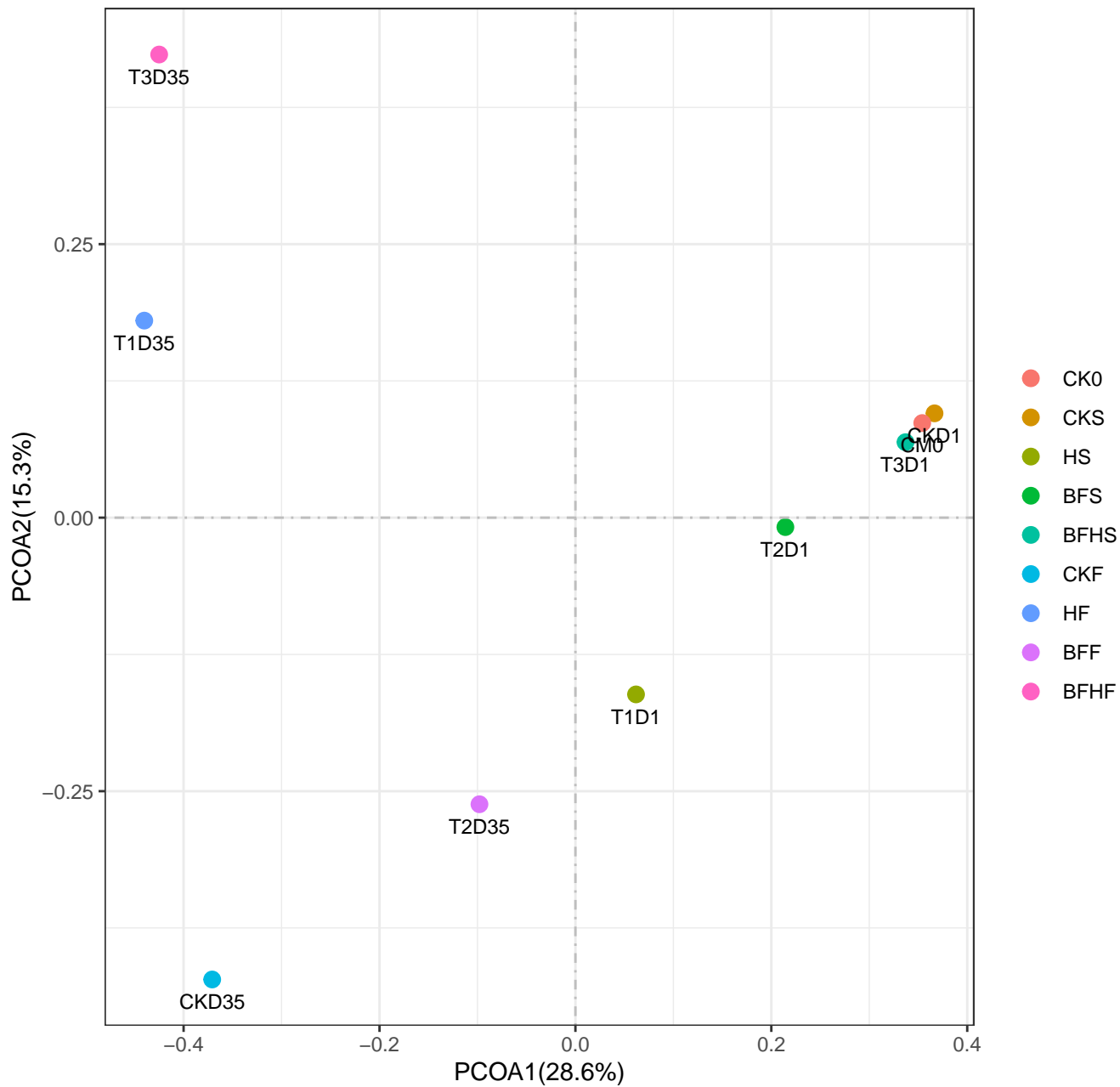

Supplement: Supplementary file 1 [file Data_Sheet_1.zip › 4.Beta_diversity/PCoA/bray_curtis_PCoA_name.pdf]

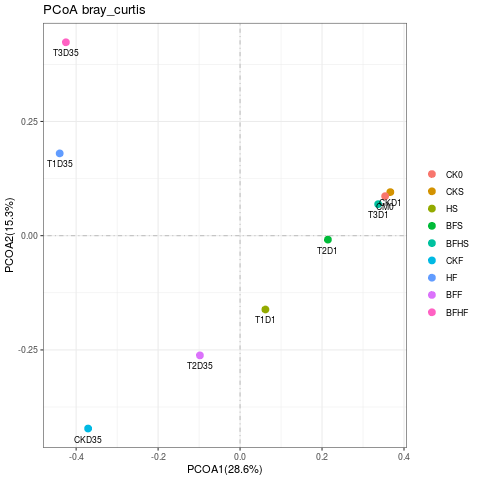

Supplement: Supplementary file 1 [file Data_Sheet_1.zip › 4.Beta_diversity/PCoA/bray_curtis_PCoA_name.png]
